# Controlled reductive C–C coupling of isocyanides promoted by an aluminyl anion†

**DOI:** 10.1039/d3sc01387a

**Published:** 2023-05-12

**Authors:** Matthew J. Evans, Mathew D. Anker, Claire L. McMullin, Martyn P. Coles

**Affiliations:** a School of Chemical and Physical Sciences, Victoria University of Wellington P. O. Box 600 Wellington New Zealand martyn.coles@vuw.ac.nz; b Department of Chemistry, University of Bath Bath BA2 7AY UK

## Abstract

We report the reaction of the potassium aluminyl, K[Al(NON)] ([NON]^2−^ = [O(SiMe_2_NDipp)_2_]^2−^, Dipp = 2,6-iPr_2_C_6_H_3_) with a series of isocyanide substrates (R-NC). In the case of *t*Bu-NC, degradation of the isocyanide was observed generating an isomeric mixture of the corresponding aluminium cyanido-κ*C* and -κ*N* compounds, K[Al(NON)(H)(CN)]/K[Al(NON)(H)(NC)]. The reaction with 2,6-dimethylphenyl isocyanide (Dmp-NC), gave a C_3_-homologation product, which in addition to C–C bond formation showed dearomatisation of one of the aromatic substituents. In contrast, using adamantyl isocyanide Ad-NC allowed both the C_2_- and C_3_-homologation products to be isolated, allowing a degree of control to be exercised over the chain growth process. These data also show that the reaction proceeds through a stepwise addition, supported in this study by the synthesis of the mixed [(Ad-NC)_2_(Dmp-NC)]^2−^ product. Computational analysis of the bonding within the homologised products confirm a high degree of multiple bond character in the exocyclic ketenimine units of the C_2_- and C_3_-products. In addition, the mechanism of chain growth was investigated, identifying different possible pathways leading to the observed products, and highlighting the importance of the potassium cation in formation of the initial C_2_-chain.

## Abstract 2

Controlled addition of isocyanides R-NC to a potassium aluminyl afforded the C2- and C3- homologized products.

## Graphical abstract

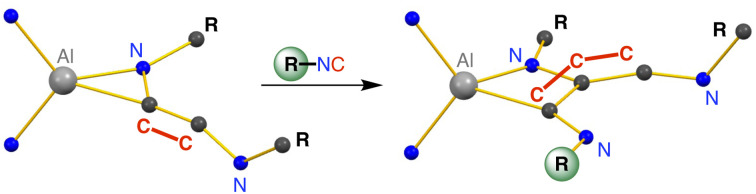

## Introduction

Isocyanides (R-NC) participate in a wide range of chemical transformations, which is undoubtedly associated with their ability to react with nucleophiles, electrophiles and radicals.^1^ As a consequence they are frequently used as reagents in multicomponent reactions,^2^ where they are considered as C_1_-synthons.^3^ They offer an intrinsic advantage over oxygenated C_1_-species (*e.g.* CO and CO_2_), due to the steric and electronic tuning that can be achieved through modification of the pendent *N*-substituent.^4^

**Scheme 1:**
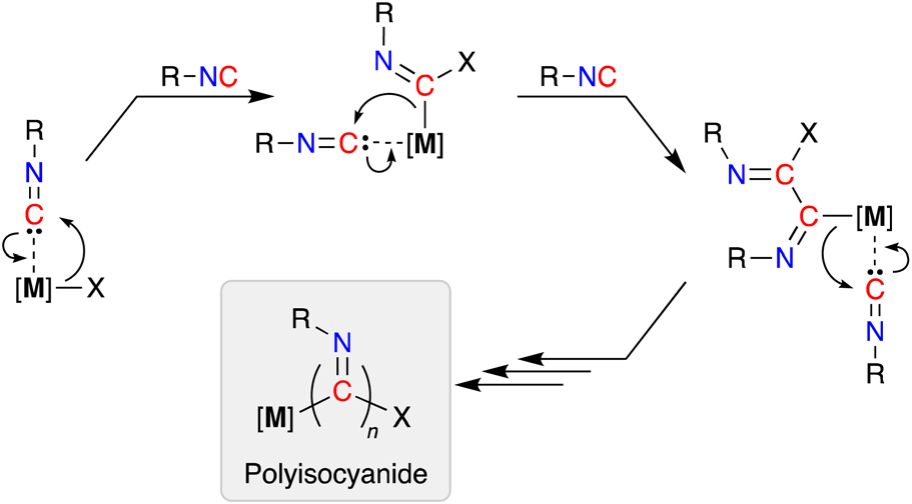
Generic mechanism for the polymerization of isocyanides. Note: an intermolecular process may also initiate the reaction, in which an external nucleophile attacks the carbon atom of a coordinated isonitrile ligand, generating the first propagating iminoacyl species.

In addition to their stoichiometric incorporation into more complex molecules, the ability to homopolymerize isocyanides into high molecular weight materials is an established reaction.^5^ The resulting polyisocyanide materials consist of a saturated carbon backbone with a helical arrangement of pendant imine functionalities^6^ that incorporate the R-group present in the isocyanide monomer.^7^ Transition metals are widely used to promote these polymerizations and have traditionally been based on precious metals (*e.g.* Pd, Rh),^8^ although extension to base metals has attracted recent attention.^9^ The mechanism of chain-growth in these systems follows a sequential insertion of coordinated isocyanides into [M]–C bonds of the propagating iminoacyl intermediate (Scheme 1).^10^

The reductive coupling of isocyanides to low molecular weight oligomers may offer further insight into the mechanism of polymerization. However, molecular species that allow controlled insertion reactions leading to short chain homologues of isocyanides are relatively scarce.^11^ Early work with transition metal complexes demonstrated that controlled dimerisation of isocyanides primarily gave compounds containing C_2_-ethynediamine ligands (RHN–C≡C–NHR), typically achieved through a reductive coupling of two coordinated isocyanide ligands under acidic (Zn^2+^/H_2_O) conditions.^12^ More recently, the direct dimerization by low-valent transition metals^13^ and uranium,^14^ has been reported, whereas the formation of higher homologues is less common and is limited to examples of C_3_-,^13*e*,15^ C_4_-,^16^ and C_6_-products.^16*a*^

**Fig. 1:**
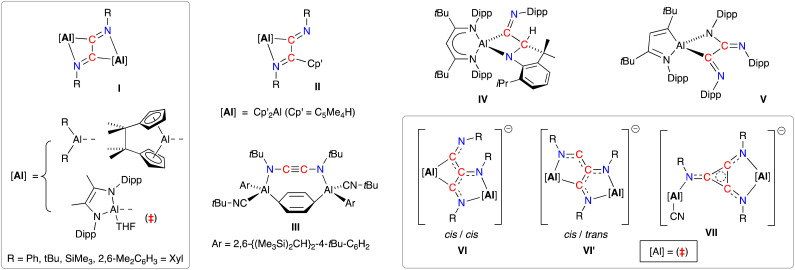
Di- and trimerisation of isocyanides promoted by aluminium reagents.

A resurgence of interest in the chemistry of (low-valent) s- and p-block complexes has established many examples of parallel reactivity to that of d- and f-block metal compounds.^17^ In the context of this study, main-group systems have been shown to access a range of C_2_- and C_3_-coupled isocyanide products exploiting the reducing abilities of the low-valent elements.^18^ The reductive dimerisation to form 1,2-diazabutadiene-2,3-diyl ligands, [RN=C–C=NR]^2−^ (R = Ph, *t*Bu, SiMe_3_, 2,6-Me_2_C_6_H_3_ = Dmp), has been noted in the presence of bimetallic dialanes (I, Fig. 1)^19^ and digallanes,^20^ as well as dimeric Mg(i)^21^ and Ge(i)^22^ compounds. A similar diimine product is obtained from the insertion of two molecules of *t*BuNC into an Al–C bond of AlCp′_3_ (II, Cp′ = [C_5_Me_4_H]^−^).^23^ In contrast, when the masked dialumene reagent [Al(Ar)]_2_(μ-C_6_H_6_) (Ar = 2,6-{(Me_3_Si)_2_CH}_2_-4-*t*BuC_6_H_2_) is reacted with *t*BuNC, the coupled product is best described as the ethynediamide ligand [*t*BuNC≡CN*t*Bu]^2−^, which contains a linear NCCN unit that bridges two Al centres (III).^24^ We also note that, while the reaction of the bulky β-diketiminate aluminium compound Al(_*t*Bu_BDI^Dipp^) (_*t*Bu_BDI^Dipp^ = [HC(C*t*BuNDipp)_2_]^−^, Dipp = 2,6-iPr_2_C_6_H_3_) with DippNC afforded reductively coupled dimers of the isocyanide, additional reactivity afforded products in which either an iPr group of the DippNC substrate (IV) or the _*t*Bu_BDI^Dipp^-ligand (V) was also activated.^25^ This area has recently evolved to include examples of linear- and cyclo-trimerized isocyanides (VI–VII), generated when the dialumane [(THF){L}Al–Al{L}(THF)]_2_ ({L} = [{DippNC(Me)}_2_]^2−^) is reacted with *t*Bu-NC in the presence of sodium as an additional reducing agent.^19*c*^ The crystallographically characterized products show that the linear C_3_-trimers exist as isomeric radical trianionic [(*t*BuNC)_3_]^3˙−^ ligands, whilst the cyclic trimer forms an aromatic C_3_-ring in the [*cyclo*-(*t*BuNC)_3_]^2−^ ligand.

Alkali metal aluminyls^26^ are a new class of anionic Al(i) complex that have been shown to activate a range of small molecules (*e.g.* H_2_,^27^ CO_2_,^28^ C_2_H_4_ (ref. 29)) and E–H bonds (E = C,^27*a*,30^ N,^31^ Si,^31*a*,*b*,32^ O,^31*b*^ P^31*b*^). We have focussed our studies in this field on the [Al(NON)]^−^ anion ([NON]^2−^ = [O(SiMe_2_NDipp)_2_]^2−^), which has been isolated as the full series of alkali metal salts, Li–Cs.^27*b*,30*c*,33^ We have recently established that the contacted dimeric pair [K{Al(NON)}]_2_,^33^ and the monomeric ion pair, (NON)Al–K(TMEDA)_2_,^34^ will promote the homologation of CO to afford linear-[(CO)_4_]^4−^ and -[(CO)_5_]^5−^ ligands.^35^ Analogous CO homologation chemistry was observed using a related three-coordinate aluminyl anion.^36^ Although inherent difficulties associated with gas phase reactions involving CO prevented the acquisition of mechanistic details from experimental evidence, a quantum chemical study on the latter system showed that the key step in the formation of the C_4_-homologue [(CO)_4_]^4−^ was the C=C bond formation between two monometallic carbene units, each consisting of a bent coordinated ketene ligand.^36^ More detailed mechanistic analysis of the CO homologation sequence (from C_1_ → C_2_ → C_3_ → C_4_) promoted by bimetallic Al/TM (TM = Cr, Mo, W, Mn, Re, Co) systems concluded similar metallocarbene complexes of the transition metals for the C_3_- and C_4_-products.^37^

There have been no reports on reductive homocoupling of isocyanides initiated by aluminyl anions. In this contribution we report the reactivity of K[Al(NON)]‡‡K[Al(NON)] is known to exist as the contacted dimeric pair [K{Al(NON)}]_2_ in the solid- and solution-states. to a range of aromatic and aliphatic isocyanides resulting in the selective production of C_1_-, C_2_- or C_3_-homologated products. These studies are complimented by density functional theory (DFT) experiments to provide insight into the mechanism of C–C chain growth.

## Results and discussion

**Scheme 2:**
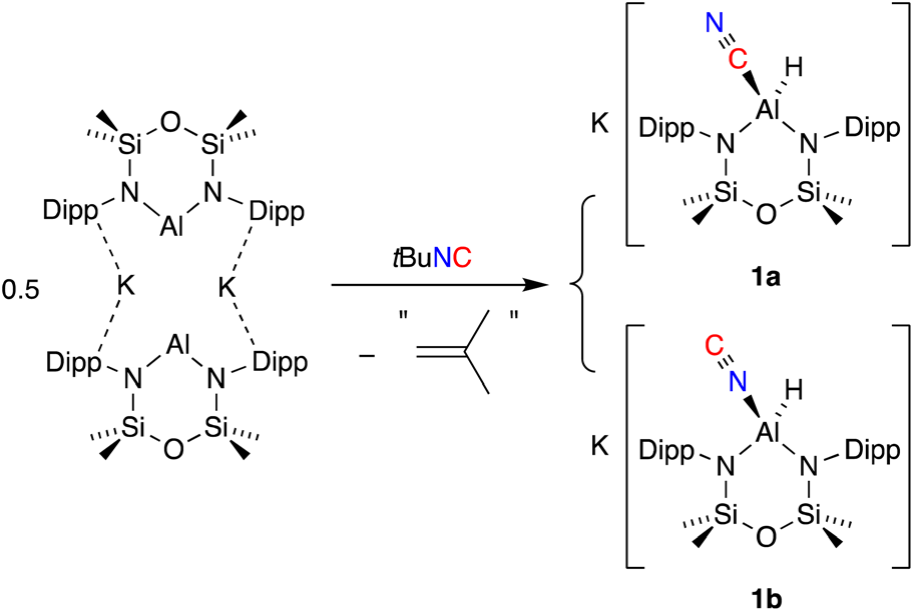
Synthesis of a mixture of K[Al(NON)(CN)] (1a) and K[Al(NON)(NC)] (1b).

The addition of one equivalent of *tert*-butyl isocyanide (*t*Bu-NC) to a yellow solution of K[Al(NON)], resulted in immediate decolorization (Scheme 2). The ^1^H NMR spectrum of the crude product revealed an absence of peaks that could be assigned to a *t*Bu group and indicated the presence of an isomeric mixture with overlapping NON-ligand signals. Based on these observations and previous reports on the reactions of other aluminium-based systems with *t*Bu-NC,^19*c*,38^ we postulated that the *t*Bu moiety was lost as isobutene (not detected), yielding the (hydrido)aluminium product that was assigned as a mixture of the cyanido-κ*C* (cyanide, 1a) and cyanido-κ*N* (isocyanide, 1b) structural isomers. A similar isomerisation of the cyanido ligand has been observed and studied in detail for a magnesium(ii) complex.^39^ This study reported that the activation barrier between the two isomers was small and that the cyanido-κ*N* isomer was favoured, with key ^13^C {^1^H} NMR signatures identified for each isomer (*δ*_C_ Mg–N*C* = 175.9; *δ*_C_ Mg–*C*N = 144.3).

For the mixture of 1a and 1b, we tentatively assign the cyanido-κ*C* isomer 1a as the major product, based on a more intense (broad) signal in the ^13^C {^1^H} NMR spectrum at *δ*_C_ 141.0 compared to the cyanido-κ*N* isomer at *δ*_C_ 174.2 (Fig. S4†). 2D correlation experiments were used to identify the respective Si*Me*_2_ signals in the ^1^H NMR spectrum and the partial separation of these resonances were used to determine the relative ratio of isomers 1a and 1b as 4 : 1 (Fig. S2†). IR data collected on crystals of 1a/1b show a characteristic Al–H stretch at 1755 cm^−1^,^27*b*^ with a weak absorption at 2119 cm^−1^ that is assigned to the CN stretching vibration of the cyanido-κ*C* isomer, 1a. The corresponding *ν*_CN_ for the cyanido-κ*N* derivative is predicted at 70–100 cm^−1^ lower than the Al–CN isomer,^39^ but was not observed.

To further our understanding of the Al–CN/Al–NC isomerisation, a sample in 1 prepared *in situ* was reacted with 1 equivalent of 222-cryptand, affording a mixture of [K(2.2.2)crypt][Al(NON)(H)(CN)] (1a-crypt) and [K(2.2.2)crypt][Al(NON)(H)(NC)] (1b-crypt). ^1^H NMR data show a similar splitting of the Si*Me*_2_ resonances with a ratio of 5 : 3 in favour of the cyanido-κ*C* isomer (Fig. S8†). The AlN*C* resonance of 1b-crypt was observed at *δ*_C_ 179.3 in the ^13^C{^1^H} NMR spectrum, although the corresponding Al*C*N peak was not observed presumably due to broadening caused by the ^27^Al nucleus. The diagnostic absorptions in the IR spectrum of 1a-crypt/1b-crypt (*ν*_CN_ 2106 cm^−1^; *ν*_Al–H_ 1792 cm^−1^) are comparable to those in 1a/1b.

**Fig. 2:**
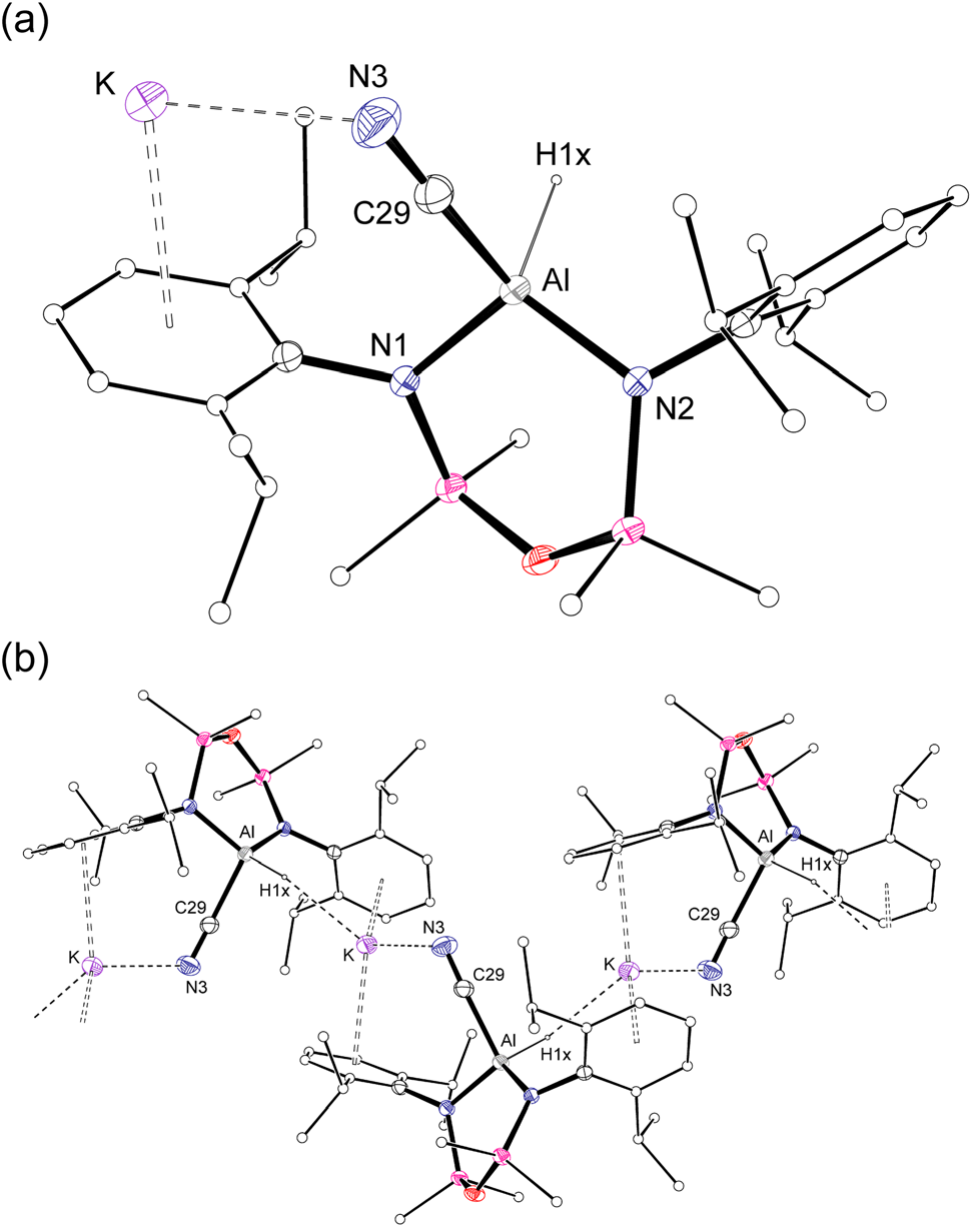
(a) Displacement ellipsoid plot (30%) of the asymmetric unit of K[Al(NON)(H)(CN)] (1a). (b) Section of the 1-D chain formed by intermolecular K⋯H and K⋯N/C interactions.

**Fig. 3:**
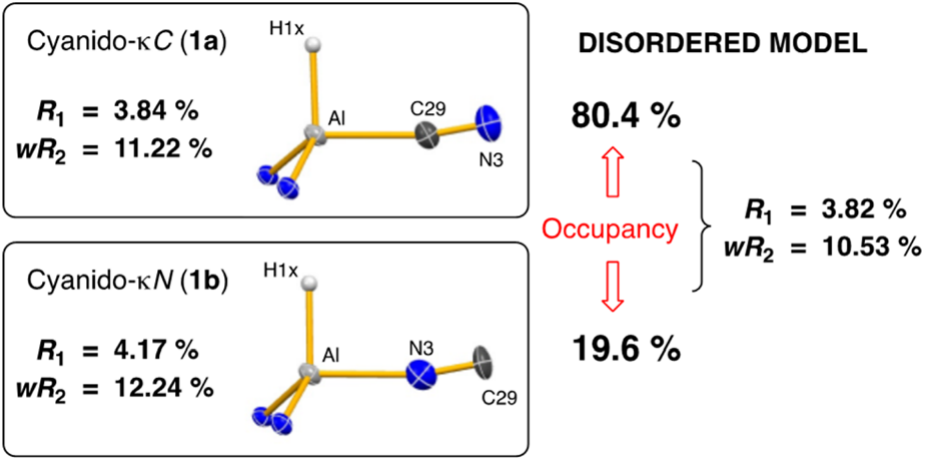
Summary of crystallographic data for the models of K[Al(NON)(H)(CN)] (1a) and K{Al(NON)(H)(NC)] (1b).

Single crystal X-ray diffraction data was collected on 1a/1b and the structure was modelled as (i) Al–CN (Fig. 2a), (ii) Al–NC and (iii) disordered Al–(C/N)(N/C). The most stable refinement was obtained with a disordered model, in which the cyanido-κ*C* isomer 1a was the major contributor (80.4%), which correlates well with the spectroscopic data (Fig. 3). Residual electron density was present near the aluminium centre, consistent with a terminal hydride ligand thereby confirming the overall structure as K[Al(NON)(H)(CN/NC)]. The aluminium adopts a distorted tetrahedral geometry, with the K cation engaging in intramolecular π(arene) interactions to a Dipp substituent with additional K⋯C/N interactions to the cyanido group. Additional intermolecular K⋯π(arene) and K⋯H interactions generate a 1-D chain parallel to the *b*-axis of the unit cell (Fig. 2b). The Al–C/N bond distance of 2.000(2) Å in 1 is within the range of those reported for related aluminium compounds containing the “Al–CN/Al–NC” fragment (1.989(3) Å–2.047(3) Å),^19*c*,38,40^ with a short C≡N bond length (1.153(3) Å) consistent with a triple bond. Unfortunately, analysis of 1a-crypt/1b-crypt by X-ray crystallography revealed additional disorder in the position of the aluminium and in the backbone of the NON-ligand, preventing any additional useful information from being obtained (Fig. S11†).

**Scheme 3:**
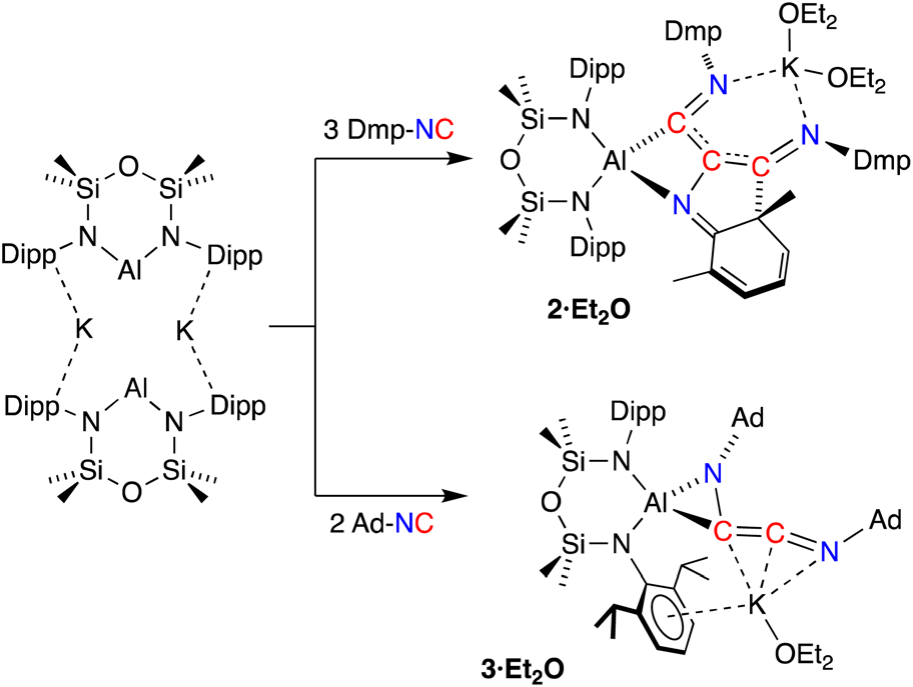
Synthesis of 2·Et_2_O and 3·Et_2_O.

Since the desired homologation reaction did not occur with *tert*-butyl isocyanide, we therefore extended the study to the reactions between K[Al(NON)] and 2,6-dimethylphenyl- (Dmp-) and 1-adamantyl- (Ad-) isocyanides. The addition of Dmp-NC to a yellow diethyl ether solution of K[Al(NON)] resulted in a colour change to dark purple (Scheme 3). The ^1^H NMR spectrum of crystals isolated from the reaction indicated the presence of more than one species in solution. An analytically pure sample of the major product (2) was prepared by washing the crystals with hexane and drying the sample under high vacuum. Attempts to characterize the by-products in the hexane wash were unsuccessful.

The ^1^H NMR spectrum of 2 revealed a highly asymmetric environment for the NON-ligand, best represented by the presence of four singlets between *δ*_H_ 0.38 and −0.15 for the Si*Me*_2_ groups. The Dmp-methyl groups appear as six singlets between *δ*_H_ 2.53 and 0.93 that integrate for 18H, consistent with the incorporation of three Dmp-NC molecules in the structure of 2. However, the presence of low field signals in the ^1^H NMR spectrum (*δ*_H_ 5.79, 1H; 5.71–5.62, 2H) and a loss of symmetry for one of the Dmp-rings in the ^13^C {^1^H} NMR spectrum (*δ*_C_ 156.3, 133.6, 123.5, 122.4, 122.3, 63.4) were reminiscent of data observed for a related germanium complex that underwent dearomatisation of an aromatic ring system to form a metallacycle.^41^ Repeating the reaction at low temperatures (−78 °C) or varying the number of equivalents of Dmp-NC (1–3 equivalents) had no effect on reaction product and all resulted in the isolation of 2.

**Fig. 4:**
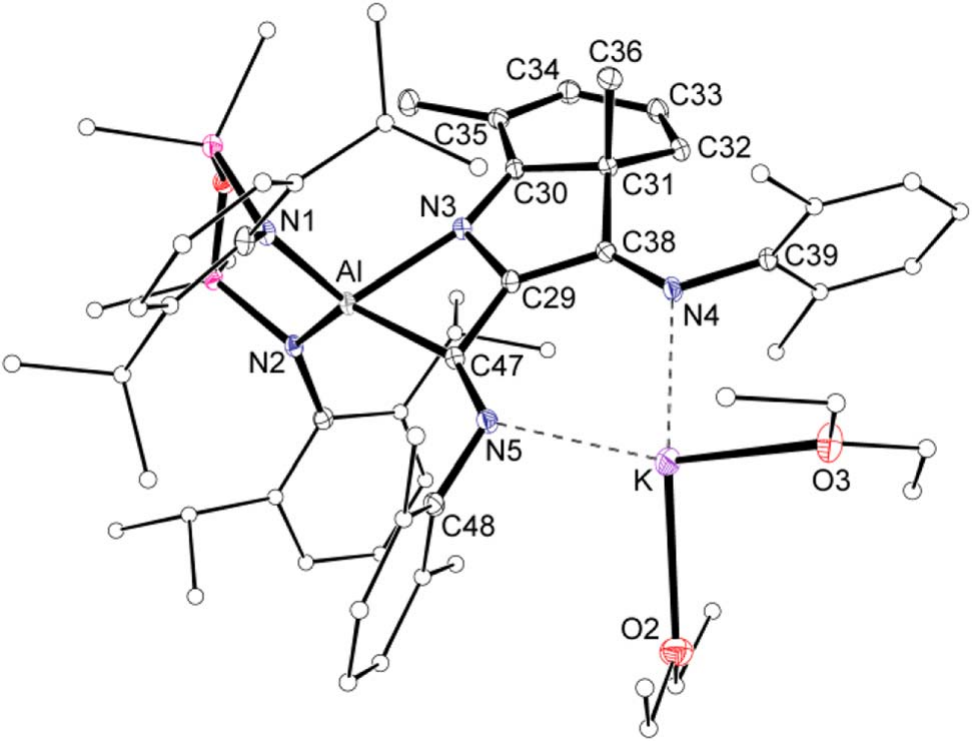
Displacement ellipsoid plot (30%) of 2·Et_2_O (H-atoms omitted, peripheral carbon atoms represented as spheres). Selected bond lengths (Å) and angles (°): Al–N1 1.8497(11), Al–N2 1.8597(11), Al–N3 2.0394(11), Al–C47 2.0226(12), C29–C38 1.4350(16), C29–C47 1.4399(17), C29–N3 1.4058(15), C38–N4 1.3009(17), C47–N5 1.3015(17), C30–C31 1.5172(16), C31–C32 1.5049(16), C32–C33 1.3419(19), C33–C34 1.4461(19), C34–C35 1.3648(18), C35–C30 1.4371(17), N3–C30 1.3200(16). N1–Al–N2 111.86(5), N3–Al–C47 70.30(5), C29–N3–C30 110.93(10), N3–C29–C47 110.53(10), C29–C47–N5 121.23(11).

Compound 2 was crystallized from Et_2_O as the bis-ether adduct, 2·Et_2_O.§§Compound 2 has also been crystallographically characterised as the toluene solvate, 2·toluene. In this structure, the potassium is not solvated by Et_2_O and has intermolecular K⋯O contacts with the NON-ligand of a neighbouring molecule, forming a polymeric 1-D chain. The structure of 2·toluene is included in the supporting information for reference (Fig. S15). The solid-state structure confirms the formation of a C_3_-chain as part of a tricyclic ring system, formed by the reductive coupling of three molecules of Dmp-NC, in addition to dearomatisation of one of the pendent Dmp-groups (Fig. 4). The Al centre adopts a distorted tetrahedral geometry supported by the bidentate NON ligand and a four-membered AlC_2_N-chelate formed from the coupled isocyanides. The potassium cation coordinates to the nitrogen atoms of two of the isocyanide groups, with the two Et_2_O molecules completing the coordination sphere.

Within the [Dmp-NC]_3_ unit of 2·Et_2_O, the C29–C38 (1.4350(16) Å) and C29–C47 distances (1.4399(17) Å) are similar, and intermediate between the idealised bond lengths for C–C single (1.54 Å) and C=C double (1.34 Å) bonds, suggesting delocalisation of electron density within the C_3_-chain. The C–N distances C38–N4 (1.3009(17) Å) and C47–N5 (1.3015(17) Å) confirm C=N double bonds. These atoms form part of a larger planar unit that incorporates the *ipso*-carbon atoms C39 and C48, with the latter atom showing the maximum deviation from the mean plane of 0.0132(8) Å. The formation of a bond between an isocyanide Dmp-*C*N atom and an *ortho*-carbon of an adjacent Dmp-group generates a cyclohexa-1,3-diene ring, with a short N3–C30 bond of 1.3200(16) Å. The ring has a fold angle of 28.48(5)° to the previously defined plane, imposed by the sp^3^-hybridised C31 atom. Analogous dearomatisation of Dmp-groups have been observed during coupling reactions of Dmp-NC initiated by complexes of scandium^42^ and vanadium.^13*e*,43^

To avoid complications due to the presence of the arene-substituent, the reaction of K[Al(NON)] with Ad-NC was investigated. When performed in a 1 : 1 ratio, a complex mixture of products was identified by ^1^H NMR spectroscopy. It was reasoned however that the bulk of the adamantyl group might provide access to the C_2_-coupled product. Therefore, the reaction was repeated with two equivalents of Ad-NC at −78 °C, allowing the isolation of 3·Et_2_O in good yields.

The ^1^H NMR spectrum of 3·Et_2_O shows broad resonances typical for the Ad-group, which overlap with the NON-ligand signals. The Si*Me*_2_ resonances are split into four signals, indicating a low symmetry at the aluminium centre. A low field peak is observed in the ^13^C{^1^H} NMR spectrum at *δ*_C_ 196.1, assigned to the central ‘–C=*C*=N–’ carbon atom of a ketenimine group. The lack of signal for the other carbon of this unit is likely a consequence of bonding to the quadrupolar ^27^Al atom (*I* = 5/2). To determine the structure of 3·Et_2_O, crystals suitable for a single crystal X-ray diffraction experiment were obtained by the slow evaporation of a diethyl ether solution.

**Fig. 5:**
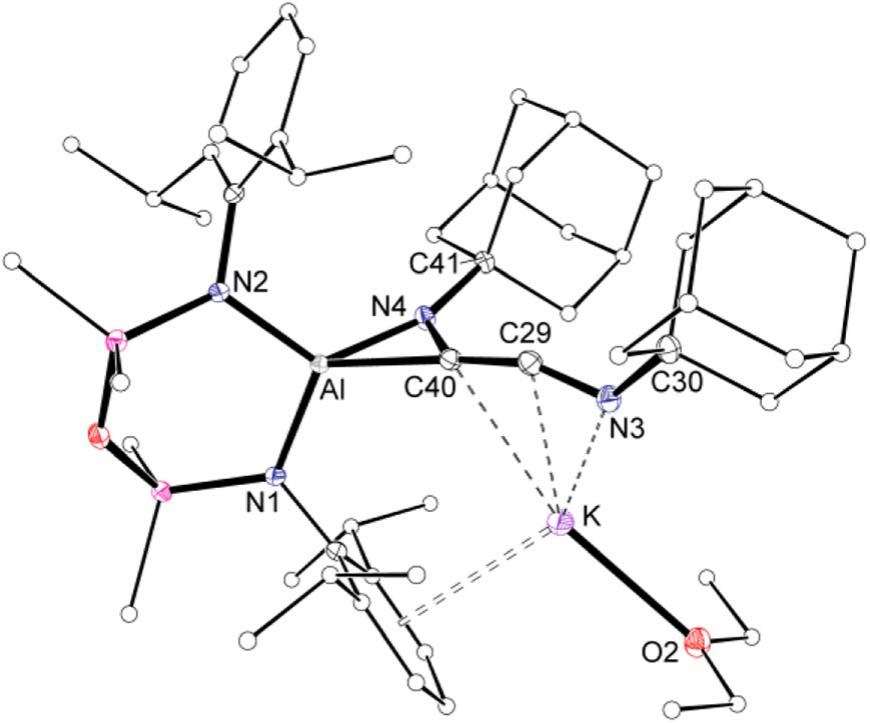
Displacement ellipsoid plot (30%) of 3·Et_2_O (H-atoms omitted, peripheral carbon atoms represented as spheres). Selected bond lengths (Å) and angles (°) presented in Fig. 7.

**Fig. 6:**
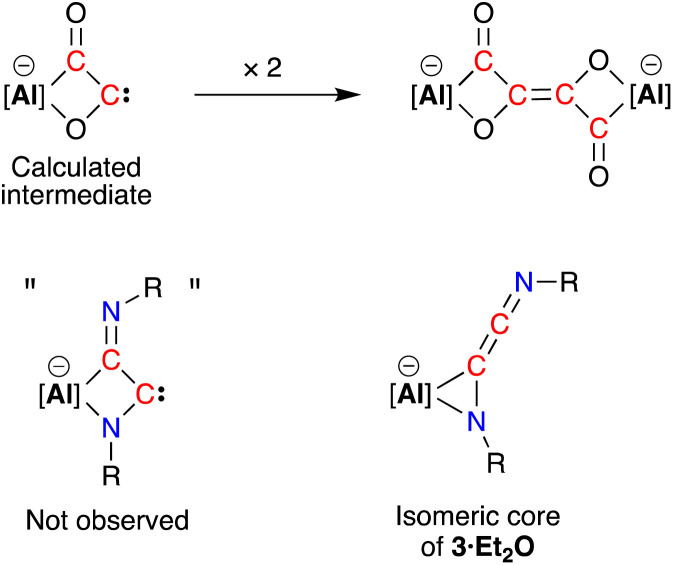
Calculated carbene intermediate in the formation of the [(CO)_4_]^4−^ ligand, with the analogous dimerised isocyanide (not observed) and isomeric core of 3·Et_2_O.

Compound 3·Et_2_O is monomeric in the solid state with a distorted tetrahedral Al centre defined by the NON-ligand, and an η^2^-*C*,*N*-bonded [{Ad-NC}_2_]^2−^ group that forms an aluminaazacyclopropane ring (Fig. 5). The coordination sphere of the K cation is defined by π(arene) interactions with a Dipp substituent, with additional interactions to the C_2_N-chain and a molecule of diethyl ether. The C40–N4 distance of 1.4292(15) Å is longer the corresponding bonds in related AlCN-rings that contain delocalised (range: 1.358(2) Å–1.379(2) Å) or unsaturated (range: 1.239(11) Å–1.322(6) Å) C–N bonds derived from carbodiimides^30*f*,44^ or isocyanides,^19*a*,24,45^ respectively, confirming an aluminaazacyclopropane ring. The C29–C40 bond length (1.3081(18) Å) is much shorter than the corresponding C–C bonds in the C_3_-unit of 2·Et_2_O and is close to the value observed for an idealised double bond. Furthermore, the C29–N3 bond length (1.2693(17) Å) and the bond angle at C29 (157.12(13)°) suggest that the exocyclic C_2_N-unit is best described as a localised ‘C=C=N’ ketenimine, with consecutive double bonds. This structure for the dimerized isocyanide contrasts with the proposed intermediate carbene that forms from the dimerization of CO during the formation of the [(CO)_4_]^4−^ ligand (Fig. 6). It is likely that the bulky R-groups enforce this structural difference, although the influence of the supporting ligands cannot be discounted. Additionally, the presence of the *N*-substituents will hinder dimerization and prevent formation of the analogous [(R-NC)_4_]^4−^ ligand. We note a recent report that describes the reductive coupling of Dmp-NC by a low-valent uranium complex in the presence of Cp*_2_Co, which afforded [U{N(SiMe_3_)_2_}_2_(Dmp-NCCNDmp)]^−^ anion containing a similar η^2^-*N*,*C*-ligand to that in 3·Et_2_O.^14^ Although described as an “acetylene diamide” complex, analysis of the bond lengths and angles by the authors concluded the same contiguous C=C and C=N double bond character as that observed in 3·Et_2_O.

**Fig. 7:**
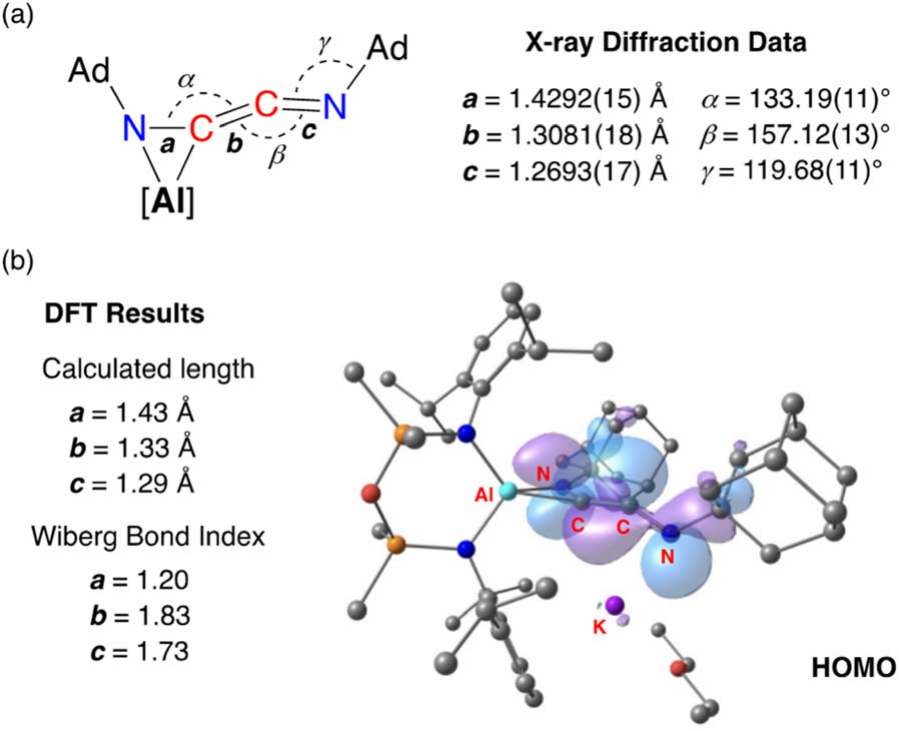
(a) Selected bond lengths and angles in 3·Et_2_O ([Al] = [Al(NON)]^−^) from X-ray diffraction data. (b) Summary of key bond parameters from DFT (BP86/BS2//BP86/BS1) and NBO analysis, with a representation of the HOMO.

The bonding within 3·Et_2_O was examined using DFT (full details in ESI†). In agreement with the proposed structure based on X-ray diffraction data (Fig. 7a), relatively large values for the Wiberg bond indices (WBIs) at C29–C40 (1.83) and C29–N3 (1.73) confirm C=C and C=N double bond character within the ketenimine unit (Fig. 7b). The HOMO shows π-electron density across the C=C bond with p-orbital characteristics at N4. The distal C29–N3 double bond is π* anti-bonding with respect to the C=C bond, with one lobe aligned with the N3–C30 bond and the other showing (sp-hybridized) lone-pair character.

**Scheme 4:**
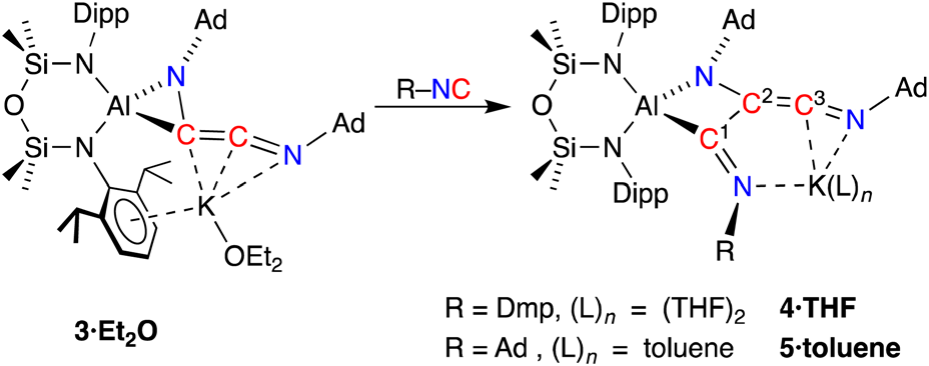
Chain growth of 3·Et_2_O upon addition of R-NC, affording 4·THF (R = Dmp) and 5·toluene (R = Ad). Carbon atom numbering in products according to NMR assignments.

To develop our understanding of the C–C chain growth mechanism from C_2_- to C_3_-products, we exposed a solution of 3·Et_2_O to one equivalent of Dmp-NC or Ad-NC affording new products 4 and 5, which were isolated as the THF (4·THF) and toluene (5·toluene) adducts, respectively (Scheme 4). The resonances in the ^1^H and ^13^C{^1^H} NMR spectra of 4·THF are broad and heating the sample to 333 K only partially resolved the signals, suggesting a rigid structure with restricted rotational freedom in solution. No evidence of dearomatisation was observed in the solution state, even when heated to 333 K. Compound 5·toluene exhibits two low field resonances in the ^13^C{^1^H} NMR spectra (*δ*_C_ 226.6 {C^3^}, 212.6 {C^1^}, Scheme 4) consistent with an additional unsaturated carbon atom in a RN=C^1^–C^2^(NR)=C^3^=NR chain. The low solubility and resulting broadening of the observed resonances for 4·THF means that only one signal is resolved, at *δ*_C_ 209.5, assigned to the C^3^-carbon atom. These data are consistent with the additional isocyanide inserting into the Al–C bond of 3·Et_2_O to form an unsaturated C_3_-chain.

**Fig. 8:**
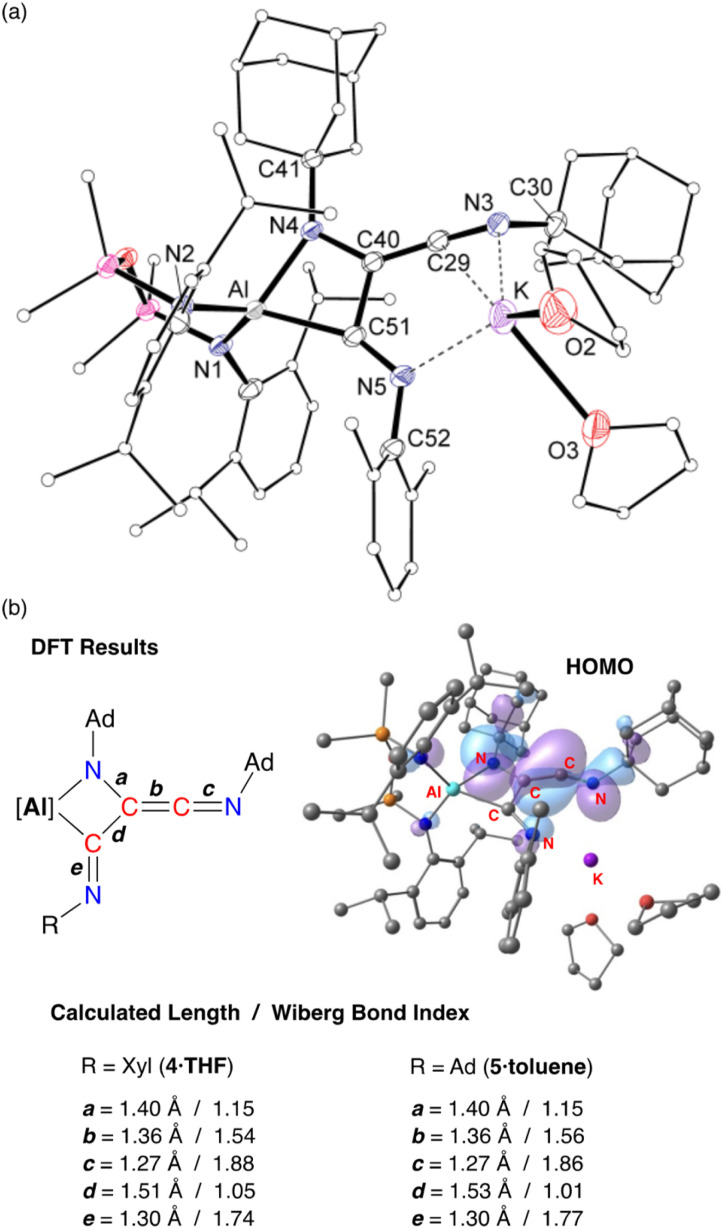
(a) Displacement ellipsoid plot (30%) of 4·THF (H-atoms and disordered atoms omitted, peripheral carbon atoms represented as spheres). Selected bond lengths and angles presented in Table 1. (b) Summary of key bond parameters from DFT (BP86/BS2//BP86/BS1) and NBO analysis of 4·THF and 5·toluene, with a representation of the HOMO of 4·THF.

X-ray crystallographic studies of 4·THF (Fig. 8a) and 5·toluene (Fig. S25†) confirm the formation of the trimerized C_3_-chain (Table 1). The key components of the two derivatives are essentially the same. Structural features are therefore discussed for 4·THF with corresponding bond lengths and angles for 5·toluene given in parentheses.

**Table 1:** Selected bond lengths (Å) and angles (°) for 4·THF and 5·toluene

	4·THF	5·Toluene
Al–N4	1.9143(19)	1.8658(15)
Al–C51	2.063(2)	2.1236(18)
N3–C29	1.246(3)	1.250(3)
C29–C40	1.334(3)	1.332(3)
C40–C51	1.512(3)	1.531(2)
C40–N4	1.400(3)	1.399(2)
C51–N5	1.280(3)	1.282(2)
N4–Al–C51	71.36(8)	71.34(7)
C30–N3–C29	120.6(2)	120.05(16)
N3–C29–C40	162.2(3)	163.75(19)
C29–C40–N4	133.4(2)	133.27(17)
C29–C40–C51	120.74(19)	120.48(16)
N4–C40–C51	105.71(18)	105.64(14)
C40–N4–C41	119.50(18)	121.22(14)
C40–N4–Al	96.13(13)	98.53(11)
C41–N4–Al	144.36(14)	140.21(12)
C40–C51–N5	117.7(2)	116.63(16)
C40–C51–Al	86.79(13)	84.35(10)
N5–C51–Al	155.26(19)	158.43(14)
C51–N5–C52	123.6(2)	122.94(16)

The trimerized isocyanide group bonds to aluminium as a κ^2^-*C*,*N*-chelate, forming a four-membered aluminaazacyclobutane ring with an acute bite angle of 71.36(8)° (71.34(7)°) at aluminium. The solvated potassium atom is contacted by an unsaturated C=N unit of the chain, with an additional contact to the terminal imine nitrogen, N5. The C–C and C–N bond lengths within the metallacycle indicate single bonds, while the exocyclic components of the C_3_-chain are consistent with a reduced bond order. For example, the C29–C40 bond length of 1.334(2) Å (1.332(3) Å) indicated double bond character, although we note that this is longer than the C=C ketenimine bond in 3·Et_2_O. In addition, the C–N distance of 1.246(3) Å (1.250(3) Å) reflects multiple bond character, with the large angle of 162.2(3)° (163.75(19)°) consistent with this model. At the other end of the C_3_-chain, the C51–N5 bond length of 1.280(3) Å (1.282(2) Å) also indicates retention of an unsaturated bond in a terminal imine group.

DFT calculations performed on 4·THF and 5·toluene are consistent with the bonding model suggested from X-ray diffraction data. The WBIs confirm single and multiple C–C bond character within the C_3_-chain, with the C29–C40 bond order of ∼1.5 lower than the corresponding value in the dimer (1.83). The HOMOs of both compounds are similar (Fig. 8b and S26†) with key components the same as that calculated for 3·Et_2_O, consisting of C=C π-bonding component that is π* anti-bonding with respect to the C29–N3 bond, and a non-hybridised p-orbital on N4. The exocyclic C51–N5 imine does not contribute to the HOMO.

**Fig. 9:**
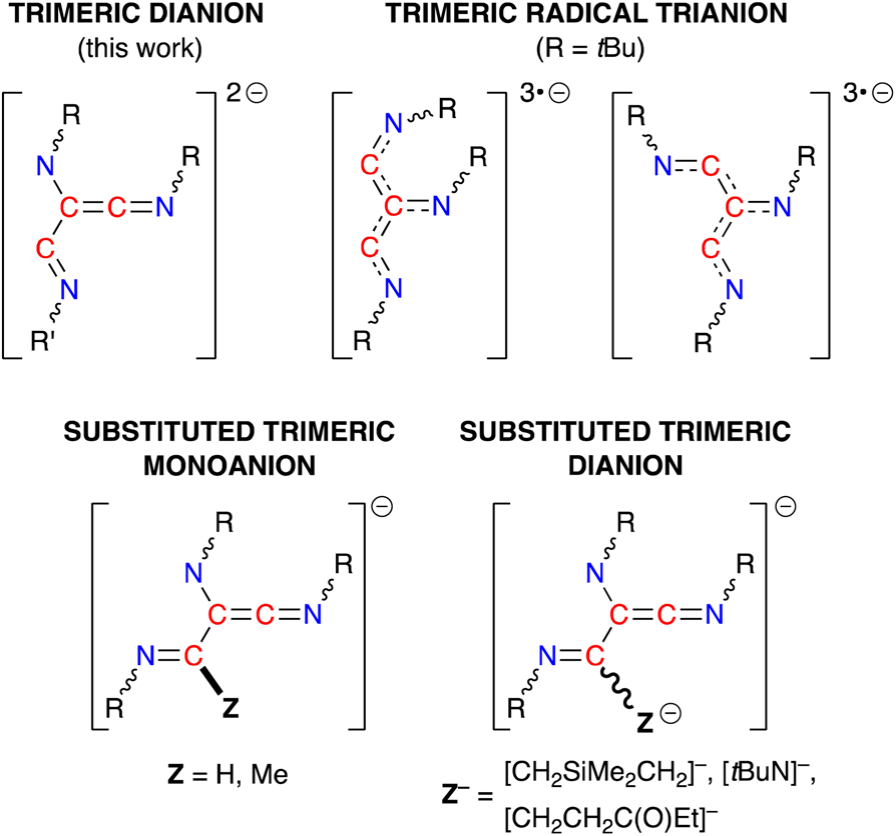
Schematic diagrams of the different ligand classes generated from the trimerization of isocyanides at metal centres.

The (R-NC)_3_ homologues in 4·THF and 5·toluene are unique examples of dianionic trimerized isocyanides, differing from the radical trianionic trimers [(*t*BuNC)_3_]^3˙−^ noted in VI (Fig. 1).^19*c*^ They are, however, structurally related to products formed during the trimerization of isocyanides at group 4 and 5 metals,^43,46^ although these transition metal promoted reactions proceed *via* an initial insertion of isocyanide into a M–H or M–C bond, resulting in carbon-substituted trimers (Fig. 9). Of relevance to this study, we note that the sequential addition of differently substituted isocyanides to the tantalum hydride complex Ta(ODipp)_2_(H)Cl_2_(PMe_2_Ph)_2_ (ref. 46d and f) or the titanium imido (κ^3^-N_2_Npy)Ti(=N*t*Bu)(py) (N_2_Npy = [(2-C_5_H_4_N)C(Me)(CH_2_NSiMe_2_)_2_]^2−^)^46*c*^ afforded non-symmetrical keteneimine ligands, similar to the ligand observed in 5·toluene. This led to two proposed mechanisms for the transition metal promoted oligomerisation that differ in the position at which the second isocyanide bonds to the growing chain. Thus, either an intermediate *N*-bound aminoketeneimine group,^46*d*^ or an imine-substituted iminoacyl intermediate^46*a*^ is postulated, which are formed from a common η^2^-iminoacyl complex derived by insertion of R-NC into a M–H or M–C bond.

We have examined the mechanism of isocyanide dimerisation and trimerisation by K[Al(NON)] leading to 3·Et_2_O, 4·THF and 5·toluene using DFT (BP86-D3BJ(PCM=C_7_H_8_)/BS2//BP86/BS1). We note that comparisons with the transition metal systems described above must be treated with caution as the initial step of the reaction with aluminyls cannot generate the analogous iminoaacyl intermediate. Furthermore the possibility exists in the current study for R-CN coordination at multiple (Al and K) centres.‡

An assessment of the dimerisation of Ad-NC to afford 3·Et_2_O was conducted. Initial work examined whether the reaction is favoured at the contacted dimeric pair (CDP) [K{Al(NON)}]_2_ (A) or *via* an initial dissociation into monomeric ‘K[Al(NON)]’ units (Fig. S27†). Splitting the dimer has an energy cost of 14.5 kcal mol^−1^, with an increase to 17.2 kcal mol^−1^ when Ad-NC is coordinated to the aluminium of the resulting monomeric K[Al(NON)] unit. However, retaining the dimeric form gave lower energy pathways for the initial coordination of Ad-NC at either aluminium or potassium.¶¶See Fig. S28. Coordination of Ad-NC at aluminium affords B (Δ*G*_tol_ = 1.1 kcal mol^−1^) and coordination at potassium generates B′ (Δ*G*_tol_ = 0.7 kcal mol^−1^). An increase in the energy of B to Δ*G*_tol_ = 4.5 kcal mol^−1^ was calculated when a second (non-coordinated) isocyanide was introduced, C. The transition-state for association of the second Ad-NC to a potassium cation of C, TS(C–D), was isolated at 3.8 kcal mol^−1^ to give adduct D (Δ*G*_tol_ = 1.9 kcal mol^−1^). TS(C–D) is higher in ‘raw’ free energy than intermediate C by 0.67 kcal mol^−1^, however, when the full methodology of single point corrections is applied (most specifically dispersion) TS(C–D) has a relative free energy value that lies below C on the reaction surface by 0.7 kcal mol^−1^. Multiple attempts were made to identify a transition state corresponding to the dissociation of “K[Al(NON)]” from D. Unfortunately, this proved unsuccessful due to the flat nature of the reaction energy surface with the productive dissociation imaginary frequency not the only negative frequency located.

**Fig. 10:**
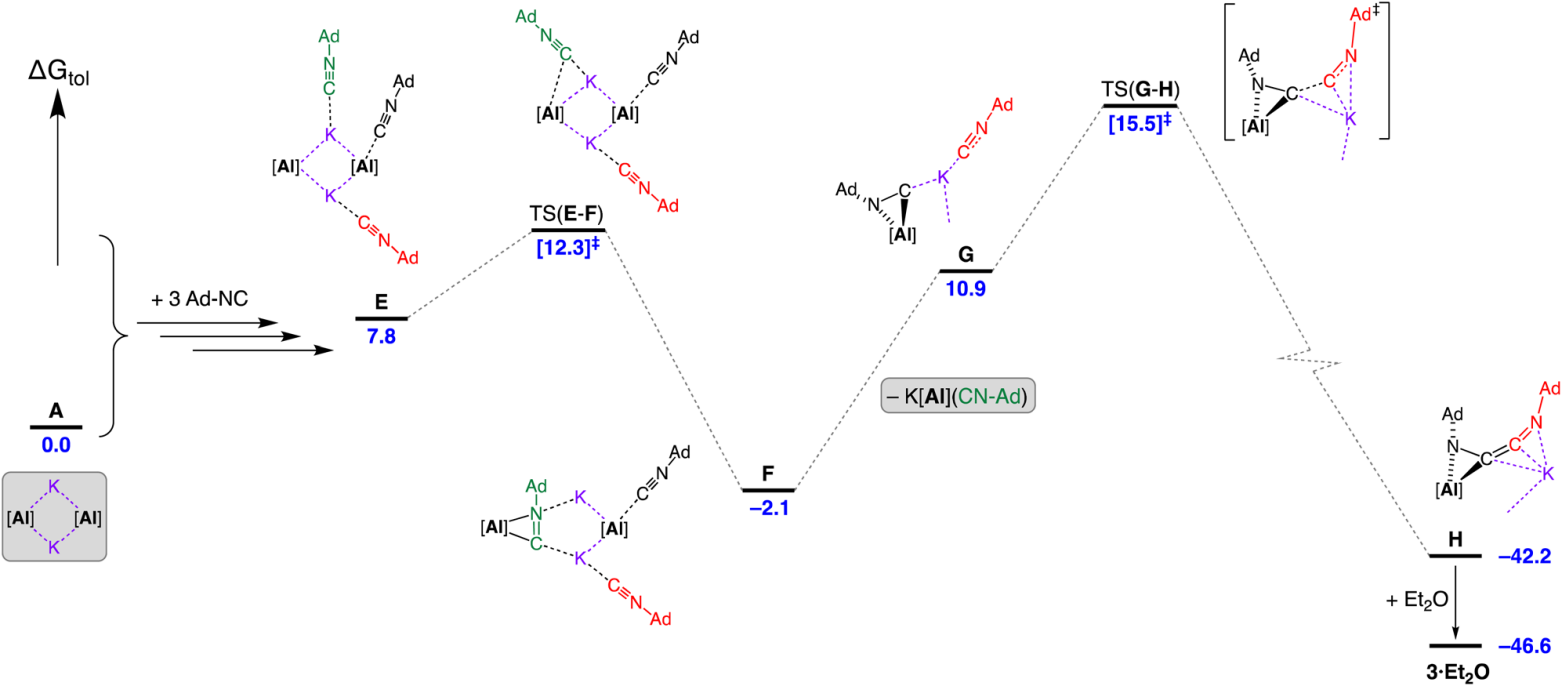
Computed free energy profile (BP86-D3BJ(PCM = C_7_H_8_)/BS2//BP86/BS1 in kcal mol^−1^) for the dimerisation of Ad-NC at [K{Al(NON)}]_2_ leading to formation of 3·Et_2_O. Note: charges and lone pairs on isocyanide groups omitted for clarity. See footnote ¶ and Fig. S28† for details of structures A–E.

Introduction of three equivalents of isocyanide (at one aluminium of the CDP and both potassium atoms) generated structure E (Δ*G*_tol_ = 7.8 kcal mol^−1^) that provided a pathway for C–C bond formation (Fig. 10). Intermediate F (Δ*G*_tol_ = −2.1 kcal mol^−1^), where the new isocyanide substrate forms an aluminaazacyclopropane ring, is accessed *via* transition state TS(E–F) with an energetically accessible barrier of 4.5 kcal mol^−1^. From intermediate F, the disruption to Al⋯K interactions within the Al_2_K_2_-core (present in structures A–E) permits an onward trajectory with loss of “K[Al(NON)(Ad-NC)]” to form G (Δ*G*_tol_ = 10.9 kcal mol^−1^). Intermediate G undergoes the critical C–C bond forming step *via* TS(G–H) to afford H (Δ*G*_tol_ = −42.2 kcal mol^−1^), *via* potassium delivery of Ad-NC to a strained azaaluminacyclopropane. This step mirrors that calculated for the C_1_ → C_2_ growth in a mixed Al/Mn system, in which exogenous CO reacts with a strained oxaaluminacyclopropane ring the give the C–C coupled product.^37*c*^ Compound H can coordinate Et_2_O to form 3·Et_2_O (Δ*G*_tol_ = −46.6 kcal mol^−1^) that is notably more stable by 4.4 kcal mol^−1^.

An alternative ‘ether assisted’ pathway for the C–C coupling step has also been identified as part of this study (Fig. S29†). The coordination of Et_2_O to the potassium cation of G before the C–C bond forming step lowers the energy by 3.0 kcal mol^−1^ to form G·Et_2_O (Δ*G*_tol_ = 7.9 kcal mol^−1^). This pathway leads to the product 3·Et_2_O*via* TS(G·Et_2_O–3·Et_2_O), with a small energy barrier of 2.3 kcal mol^−1^. We note that this is lower than the corresponding barrier for the conversion of G → H (4.6 kcal mol^−1^). However, experimental observations note that a distinct colour change to bright yellow occurs upon mixing the reagents in the reaction solvent of toluene prior to the removal of this solvent and crystallization from Et_2_O. This suggests that under the conditions of the experiment, the pathway in Fig. 10 is most likely operating, although it does highlight the potentially important role that the solvent can play in this reaction.

**Fig. 11:**
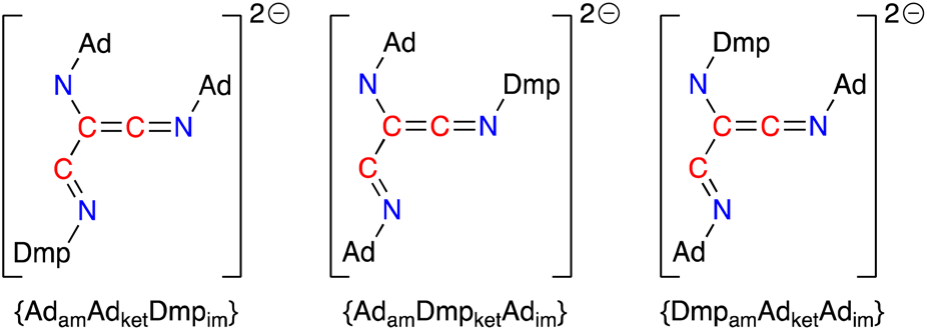
Possible forms of the [(Ad-NC)_2_(Dmp-NC)]^2−^ ligand that differ in the relative position of the amino, ketenimine and imine substituents (am = amido, ket = ketenimine, im = imine).

Based on the crystal structures of 4·THF and 5·toluene and assuming there is no interconversion between tautomers or geometric isomers, the [(Ad-NC)_2_(Dmp-NC)]^2−^ dianion can adopt three different structures that differ in the relative positions of the amido (am), imine (im) and ketenimine (ket) substituents (Fig. 11). The crystal structure of 4·THF adopts the {Ad_am_Ad_ket_Dmp_im_} form (Fig. 8a) and represents the least structural reorganisation when starting from 3·Et_2_O (which may be considered as the {Ad_am_Ad_ket_} form of the dimer). Taking this into consideration, we examined the addition of a third equivalent of R-NC to the non-solvated species H as a route to 4 (R = Dmp) and 5 (R = Ad).

**Fig. 12:**
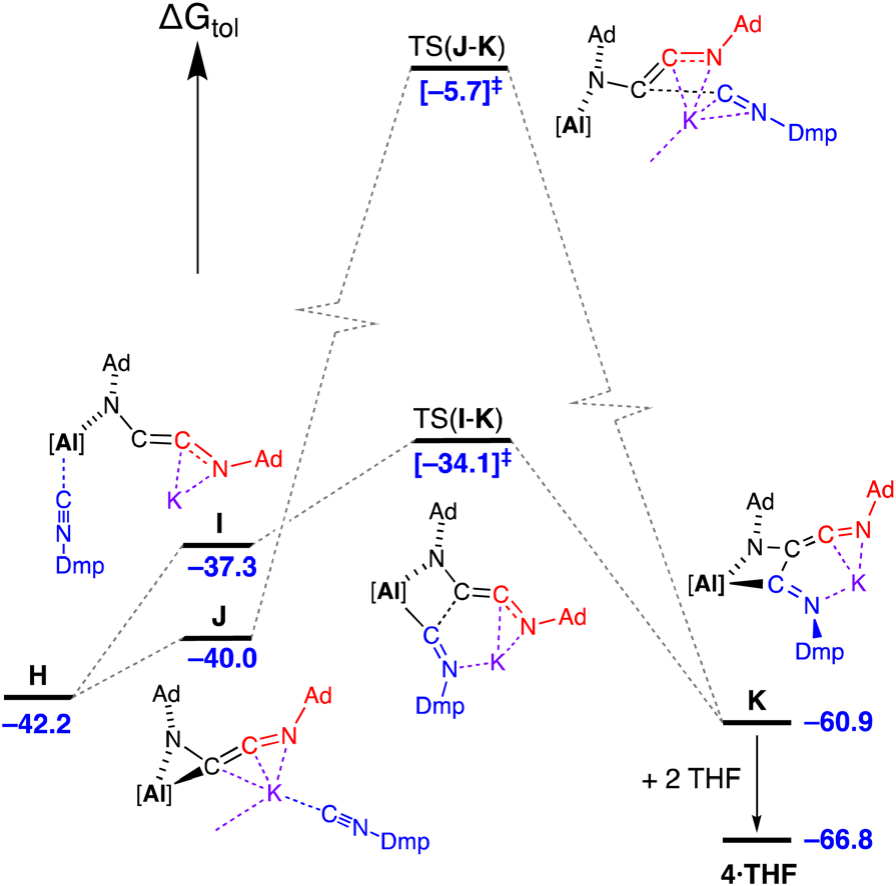
Computed free energy profile (BP86-D3BJ(PCM = C_7_H_8_)/BS2//BP86/BS1 in kcal mol^−1^) for the addition of Dmp-NC to H (third isocyanide, Dmp-NC = blue) leading to formation of 4·THF as the {Ad_am_Ad_ket_Dmp_im_} form. Note: charges and lone pairs on isocyanide groups omitted for clarity. Corresponding energy values for addition of Ad-NC (Δ*G*_tol_/kcal mol^−1^): J′ = −41.3; I′ = −36.2; TS(J′–K′) = −10.6; TS(I′–K′) = −39.4; K′ −61.6; 5·toluene = −62.3.

Three energetically accessible pathways were identified for the addition of isocyanide to the C_2_-chain in H. The addition of Dmp-NC at the potassium affords J at Δ*G*_tol_ = −40.0 kcal mol^−1^, while coordination at the aluminium forms a higher free energy structure I at −37.3 kcal mol^−1^ (Fig. 12) with a change in the coordination of the [AdNC]_2_ unit to the monodentate *N*-bound C_2_-chain. Onward reaction from I occurs *via* C–C coupling in TS(I–K) (Δ*G*_tol_ = −34.1 kcal mol^−1^) with an overall barrier from H of 8.1 kcal mol^−1^. The transition state for the onward pathway from J was also identified, although occurs at a high free energy barrier (34.3 kcal mol^−1^) compared to the barrier for TS(I–K) of only 3.2 kcal mol^−1^. As noted for the formation of 2·Et_2_O, the coordination of solvent during the crystallisation process (in this case two THF molecules associating to the K^+^ cation), stabilises structure K by 5.9 kcal mol^−1^ to give 4·THF.

The third pathway (Fig. S30†) involves a reorganization of the coordinated C_2_-ligand of H to a four-membered AlNC_2_ metallacycle followed by coordination of Dmp-NC at potassium *via* K⋯π(arene) interactions to give intermediate L (Δ*G*_tol_ = −33.8 kcal mol^−1^) and subsequent C–C bond formation with a barrier of 15.8 kcal mol^−1^. However, when Dmp-NC is introduced as the third isocyanide, this results in the {Ad_am_Dmp_ket_Ad_im_} form of the ligand coordinated to aluminium, M, which is inconsistent with the experimentally observed structure of 4·THF. Therefore, although the pathway cannot be ruled out as the possibility exists for interconversion of the different ligand forms, we consider it less likely compared with the other pathways identified that involve considerably less reorganization.

Examination of the corresponding pathways for the conversion of H to 5·toluene (*i.e.* the addition of Ad-NC to H) has also been conducted (Fig. S31†). The pathway with the lowest barrier mirrors that shown in Fig. 12, with an overall value of 6.0 kcal mol^−1^. In this case we note that the Δ*G*_tol_ value of I′ (−36.2 kcal mol^−1^) is higher than TS(I′–K′) (−39.4 kcal mol^−1^) due to the presence of dispersion effects. The pathway with insertion of Ad-NC *via* potassium, TS(J′–K′), remains relatively high with a barrier from H of 31.6 kcal mol^−1^. Finally, we note that for the homologized (AdNC)_3_ product, only one isomer is possible (*i.e.* {Ad_am_Ad_ket_Ad_im_}), legitimizing the third pathway noted previously. However, the overall barrier for the formation of K′ from H through this route remains relatively high (28.2 kcal mol^−1^) compared with the lower energy pathway *via*I′.

## Conclusions

We have demonstrated that the potassium aluminyl K[Al(NON)] is active for the homologation of organic isocyanides (R-NC), which proceeds with a stepwise chain growth mechanism. To prevent unwanted side reactions observed when R = *t*Bu (loss of isobutene and formation of isomeric (hydrido)(cyanido-κ*C*)- and (hydrido)(cyanido-κ*N*)-aluminate anions) and R = Dmp (dearomatization) the 1-adamantyl group was used. Thus, performing the reaction with Ad-NC allowed a high degree of control to be implemented, enabling isolation of the C_2_-homologue. This product was in turn reacted with additional R-NC (R = Dmp, Ad) to give the C_3_-chain growth product. Detailed analysis of the mechanism by DFT showed that formation of the C_2_-homologue involved addition of three equivalents of Ad-NC to the dimeric potassium aluminyl, facilitating loss of “K[Al(NON)(Ad-NC)]”. The potassium cation is important in this process, delivering the second equivalent of R-NC to an azaaluminacyclopropane ring thus enabling onward C–C bond formation. It was also shown that the most energetically favourable pathway leading to the C_3_-products proceeded *via* the coordination of the incoming isocyanide at aluminium and insertion into the Al–C bond of the C_2_-product.

## Data availability

The data supporting this study is available within the ESI.†

## Author contributions

M. P. C. and M. D. A. supervised the work. M. P. C. carried out the X-ray crystallographic analysis. M. J. E carried out the synthetic work. C. L. M. carried out the computational studies. M. P. C. and M. D. A. prepared the manuscript. M. J. E., M. P. C. and C. L. M. prepared the ESI.† All authors read and commented on the manuscript.

## Conflicts of interest

There are no conflicts to declare.

## Supplementary Material

SC-014-D3SC01387A-s001

SC-014-D3SC01387A-s002
