# Supplementary material for: Controlled reductive C–C coupling of isocyanides promoted by an aluminyl anion
Source: Chem Sci. 2023 May 12;14(23):6278–88. doi: 10.1039/d3sc01387a (PMC10266456; doi:10.1039/d3sc01387a)
Supplement: SC-014-D3SC01387A-s001 [file SC-014-D3SC01387A-s001.pdf]

Electronic Supplementary Material (ESI) for Chemical Science.
This journal is © The Royal Society of Chemistry 2023

Controlled Reductive C-C Coupling of Isocyanides Promoted by an Aluminyl Anion

Table of Contents:

S3 General Experimental Procedures

S3 Experimental Details for K[Al(NON)(H)(CN)] (**1a**) / K[Al(NON)(H)(NC)] (**1b**)

S4 **Figure S1** ^{1}H NMR spectrum of K[Al(NON)(H)(CN)] (**1a**) / K[Al(NON)(H)(NC)] (**1b**)

S5 **Figure S2** Expansion of Si*Me₂* region of ^{1}H NMR spectrum of **1a** / **1b**

S6 **Figure S3** ^{13}C{^{1}H} NMR spectrum of K[Al(NON)(H)(CN)] (**1a**) / K[Al(NON)(H)(NC)] (**1b**)

S7 **Figure S4** Expansion of Al–*C*N / Al–N*C* region of ^{13}C{^{1}H} NMR spectrum of **1a** / **1b**

S8 **Figure S5** IR spectrum of **1a** / **1b**

S9 **Figure S6** ORTEP of K[Al(NON)(H)(CN)] (**1a**)

S10 Experimental Details for [K(2.2.2)crypt][Al(NON)(H)(CN)] (**1a·crypt**) / [K(2.2.2)crypt][Al(NON)(H)(NC)] (**1b·crypt**)

S11 **Figure S7** ^{1}H NMR spectrum of [K(2.2.2)crypt][Al(NON)(H)(CN)] (**1a·crypt**) / [K(2.2.2)crypt][Al(NON)(H)(NC)] (**1b·crypt**)

S12 **Figure S8** Expansion of Si*Me₂* region of ^{1}H NMR spectrum of **1a-crypt** / **1b-crypt**

S13 **Figure S9** ^{13}C{^{1}H} NMR spectrum of [K(2.2.2)crypt][Al(NON)(H)(CN)] (**1a·crypt**) / [K(2.2.2)crypt][Al(NON)(H)(NC)] (**1b·crypt**)

S14 **Figure S10** IR spectrum of **1a-crypt** / **1b-crypt**

S15 **Figure S11** ORTEP of [K(2.2.2)crypt][Al(NON)(H)(CN)] (**1a·crypt**)

S16 Experimental Details for [K(Et_2O)$_2$][Al(NON){(DmpNC)$_2$(DmpNC*)] (**2·Et_2O**)

S17 **Figure S12** ^{1}H NMR spectrum of [K(Et_2O)$_2$][Al(NON){(DmpNC)$_2$(DmpNC*)] (**2·Et_2O**)

S18 **Figure S13** ^{13}C{^{1}H} NMR spectrum of [K(Et_2O)$_2$][Al(NON){(DmpNC)$_2$(DmpNC*)] (**2·Et_2O**)

S19 **Figure S14** ORTEP of [K(Et_2O)$_2$][Al(NON){(DmpNC)$_2$(DmpNC*)] (**2·Et_2O**)

S20 **Figure S15** ORTEP of K[Al(NON){(DmpNC)$_2$(DmpNC*)] (**2·toluene**)

S21 Experimental Details for [K(Et_2O)][Al(NON)(AdNC)$_2$] (**3·Et_2O**)

S22 **Figure S16** ^{1}H NMR spectrum of [K(Et_2O)][Al(NON)(AdNC)$_2$] (**3·Et_2O**)

S23 **Figure S17** ^{13}C{^{1}H} NMR spectrum of [K(Et_2O)][Al(NON)(AdNC)$_2$] (**3·Et_2O**)

S24 **Figure S18** ORTEP of [K(Et_2O)][Al(NON)(AdNC)$_2$] (**3·Et_2O**)

S25 Experimental Details for [K(THF)$_2$][Al(NON)(AdNC)$_2$(DmpNC)] (**4·THF**)

S26 **Figure S19** ^{1}H NMR spectrum of [K(THF)$_2$][Al(NON)(AdNC)$_2$(DmpNC)] (**4·THF**)

S27 **Figure S20** ^{1}H NMR spectrum (333 K) of [K(THF)$_2$][Al(NON)(AdNC)$_2$(DmpNC)] (**4·THF**)

S28 **Figure S21** ^{13}C{^{1}H} NMR spectrum of [K(THF)$_2$][Al(NON)(AdNC)$_2$(DmpNC)] (**4·THF**)

S29 **Figure S22** ORTEP of [K(THF)$_2$][Al(NON)(AdNC)$_2$(DmpNC)] (**4·THF**)

S30 Experimental Details for [K(toluene)][Al(NON)(AdNC)$_3$] (**5·toluene**)

S31 **Figure S23** ^{1}H NMR spectrum of [K(toluene)][Al(NON)(AdNC)$_3$] (**5·toluene**)

S32 **Figure S24** ^{13}C{^{1}H} NMR spectrum of [K(toluene)][Al(NON)(AdNC)$_3$] (**5·toluene**)

S33 **Figure S25** ORTEP of [K(toluene)][Al(NON)(AdNC)$_3$] (**5·toluene**)

S34 Crystallographic Details

S35 **Table S1** Crystal structure and refinement data for **1a**, **1-crypt**, **2·Et_2O** and **2·toluene**

S36 **Table S2** Crystal structure and refinement data for **3·Et_2O**, **4·THF** and **5·toluene**

S37 Computational Methodology

S37 **Figure S26** Representation of the HOMO of **5·toluene**

S39 **Table S3** Relative energies for computed structures.

S40 **Figure S27** DFT computed free energy profile for splitting the [K{Al(NON)}]$_2$ (**A**) dimer and coordination of Ad-NC

S40 **Figure S28** DFT computed free energy profile (BP86-D3BJ,(PCM=toluene)/BS2//BP86/BS1, in kcal mol^{-1}) for the coordination of three equivalents of Ad-NC to $[K\{Al(NON)\}]_2$ (**A**)
S41 **Figure S29** DFT computed free energy profile for the Et_2O assisted coupling of two Ad-NC molecules at aluminium.
S42 **Figure S30** DFT computed free energy profile for the formation of the $\{Ad_{am}Dmp_{ket}Ad_{im}\}$ isomer of $K[Al(NON)\{(AdNC)_2(DmpNC)\}]$.
S43 **Figure S31** DFT computed free energy profile for the formation of $[K(toluene)][Al(NON)(AdNC)_3]$ (**5·toluene**).
S44 **Figure S32** DFT computed free energy profile for for the formation of $K[Al(NON)(AdNC)_3]$ via the third pathway.
S45 References
S46 Cartesian coordinates and energies of computed structures.

General Experimental Procedures

All manipulations were performed under dry nitrogen or argon using standard Schlenk-line techniques, or in a conventional nitrogen-filled glovebox. Hexane, toluene, diethyl ether (Et_2O), and tetrahydrofuran (THF) were obtained from a PureSolv MD 5 system and stored over activated 5 Å molecular sieves for 24 hours prior to use. NMR spectra were recorded using a Jeol JNM-ECZ500S 500 MHz spectrometer equipped with a ROYAL digital auto tune probe S, operating at 500.1 (^{1}H) and 125.8 (^{13}C). Spectra were recorded at 294 K (unless stated otherwise) and proton and carbon chemical shifts were referenced internally to residual solvent resonances. Coupling constants are quoted in Hz. Elemental analyses were performed by the Elemental Analysis Service at London Metropolitan University. K[Al(NON)] was prepared according to the literature procedures.[1] All other chemicals were purchased from Sigma-Aldrich and used without further purification.

Experimental details for K[Al(NON)(H)(CN)] (1a) / K[Al(NON)(H)(NC)] (1b)

A solution of *tert*-butyl isocyanide (23 mg, 0.28 mmol) in toluene (~5 mL) was added to a stirred solution of K[Al(NON)] (76 mg, 0.14 mmol) at room temperature. The reaction mixture was allowed to stir for *ca.* 1 hr to give a white suspension. The volatiles were removed *in vacuo* and the residue dissolved in THF. Crystals suitable for single crystal X-ray diffraction experiments were grown at room temperature *via* slow evaporation. Yield 46 mg, 58 %.

An isomeric mixture exists in solution. Overlap in the ^{1}H NMR spectrum causes most of the peaks to be indistinguishable between the two isomers. However, a noticeable shift is observed between the Si*Me*$_2$ signals that has been used to calculate the relative ratio between each isomer as 4:1.

The ^{13}C{^{1}H} NMR spectrum also shows two signals for a the Al–*C*N / Al–N*C* ligands and has also been used to distinguish the two isomers. In the ^{13}C{^{1}H} NMR spectrum, signals associated with the minor isomer are denoted with an asterisk (*).

^{1}H NMR (500 MHz, THF-D$_8$): δ 6.93 (d, J = 7.5, 3H, C$_6$*H*$_3$), 6.79 (t, J = 7.5, 2H, C$_6$*H*$_3$), 4.09 – 4.00 (m, 4H, C*H*Me$_2$), 1.30 (d, J = 6.8, 6H, CH*Me*$_2$), 1.22 – 1.14 (m, 18H, CH*Me*$_2$), 0.11, 0.02 (s, 6H, Si*Me*$_2$).

*SiMe$_2$ ^{1}H NMR signals for each isomer: 0.11 (s, 6.0H, **Major**), 0.11 (s, 1.5H, **Minor**), 0.02 (s, 6.0H, **Major**), 0.01 (s, 1.5H, **Minor**).*

^{13}C{^{1}H} NMR (126 MHz, THF-D$_8$): δ 174.2* (Al–N*C*, **Minor**), 147.9*, 147.7, 147.6*, 146.1*, 146.0 (*C*$_6$H$_3$), 141.0 (Al–*C*N, **Major**), 123.8, 123.6*, 123.5, 123.4*, 122.1, 122.0* (*C*$_6$H$_3$), 28.0, 27.7, 27.5*, 27.1*, (*C*HMe$_2$), 26.9, 26.8, 26.0, 25.9*, 25.7*, 25.6 (CH*Me*$_2$), 3.1*, 3.0, 2.6, 2.6* (Si*Me*$_2$).

IR (solid, cm^{-1}): 2119 (υ_{CN}), 1755 (υ_{Al-H}).

Figure S1 ^{1}H NMR spectrum (500 MHz, THF-D_8) of K[Al(NON)(H)(CN)] (**1a**) / K[Al(NON)(H)(NC)] (**1b**)

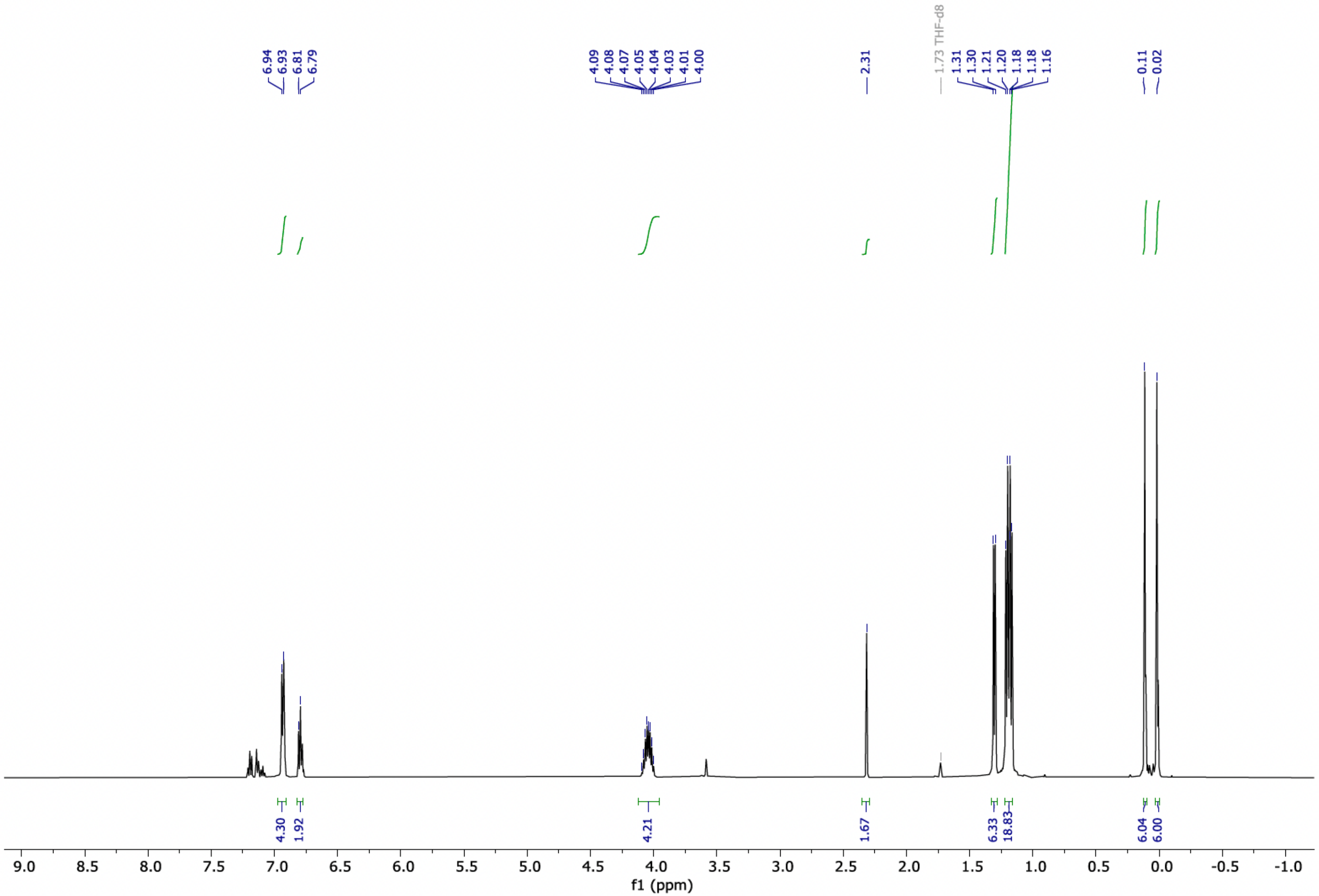

Figure S2 Expansion of Si*Me₂* region of the ^{1}H NMR spectrum (500 MHz, THF-D$_8$) of K[Al(NON)(H)(CN)] (**1a**) / K[Al(NON)(H)(NC)] (**1b**)

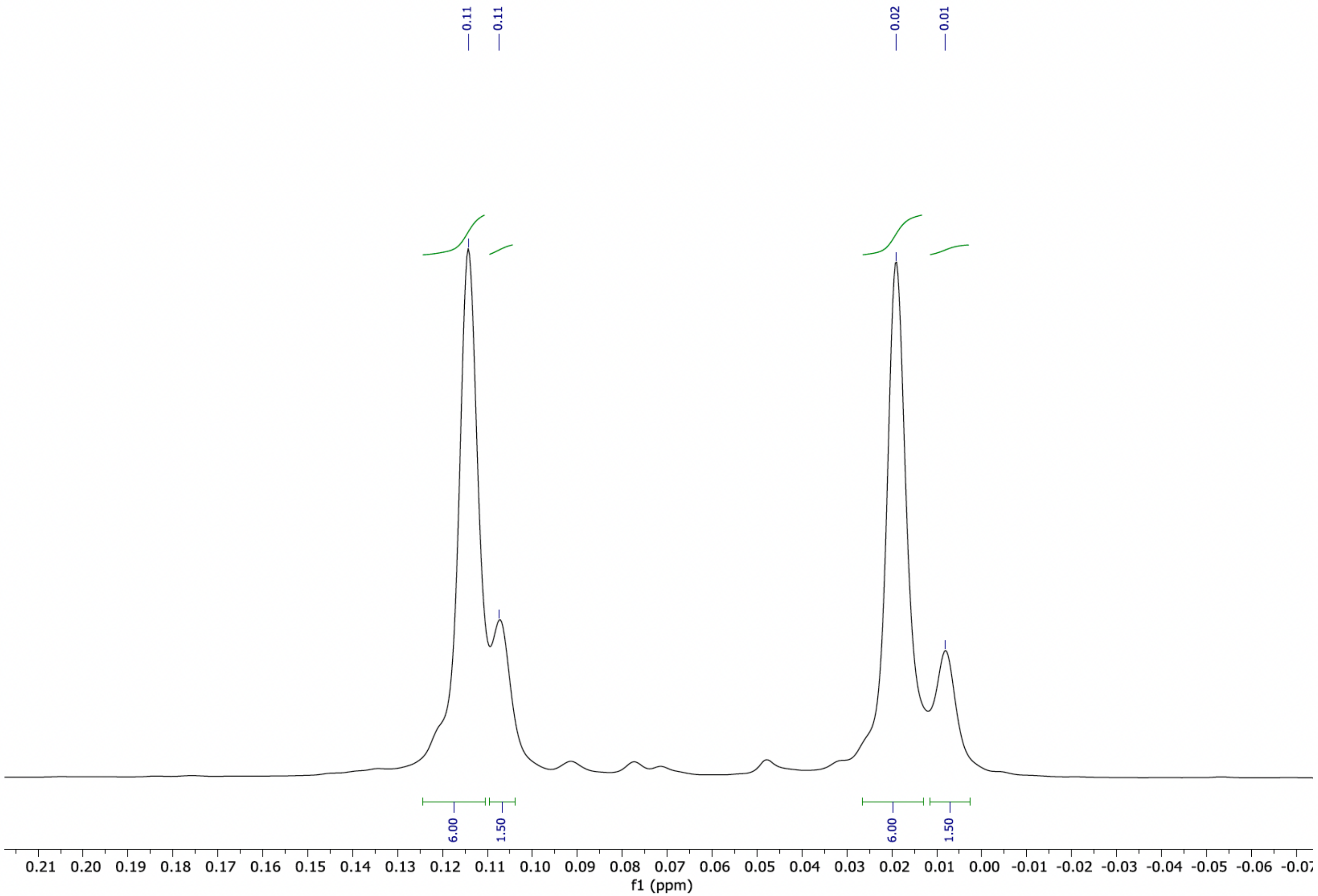

Figure S3 $^{13}C\{^{1}H\}$ NMR spectrum (126 MHz, THF-D$_8$) of K[Al(NON)(H)(CN)] (**1a**) / K[Al(NON)(H)(NC)] (**1b**)

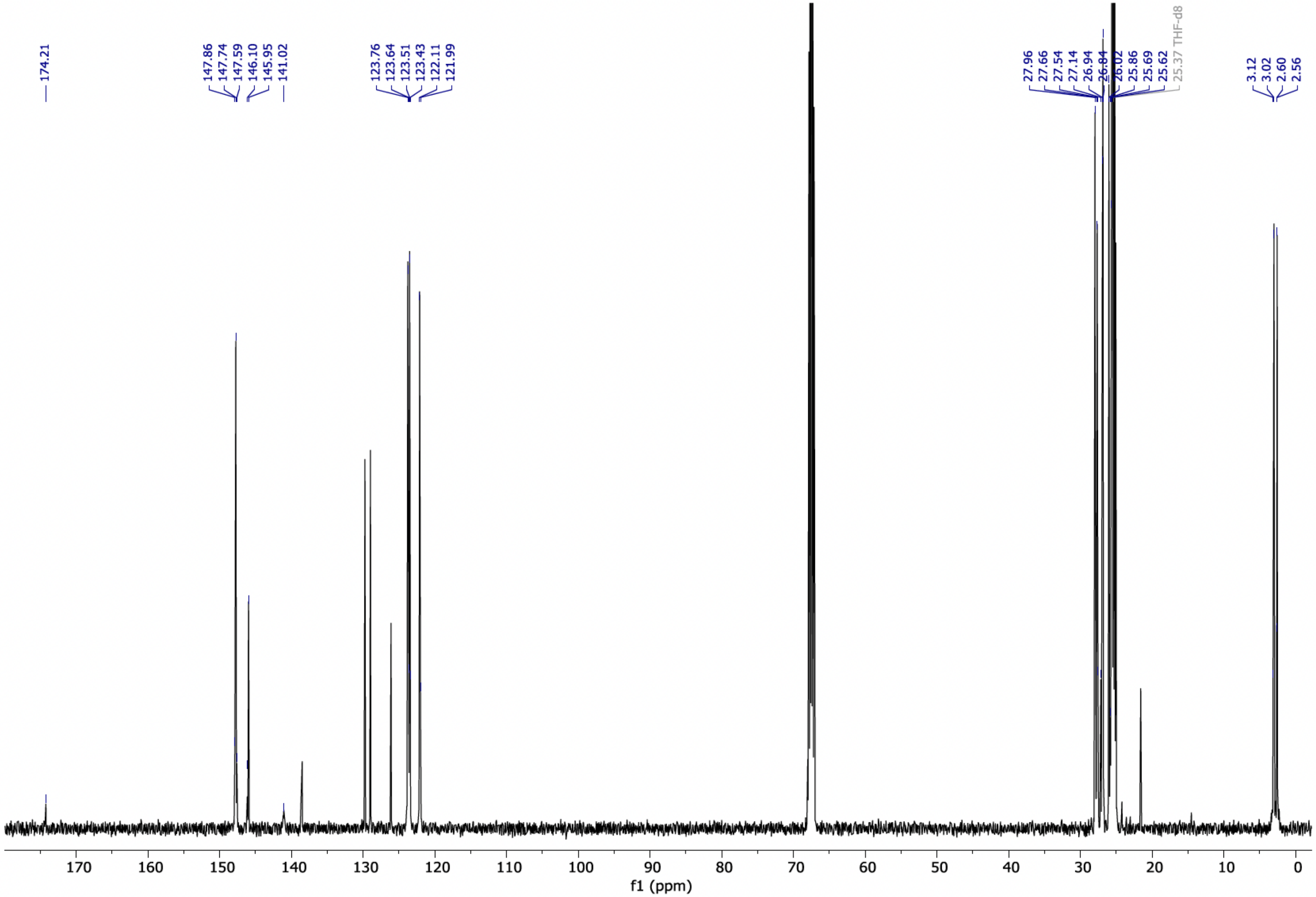

Figure S4 Expansion of Al–*C*N / Al–N*C* region of $^{13}C\{^1H\}$ NMR spectrum (126 MHz, THF-D_8) of K[Al(NON)(H)(CN)] (**1a**) / K[Al(NON)(H)(NC)] (**1b**)

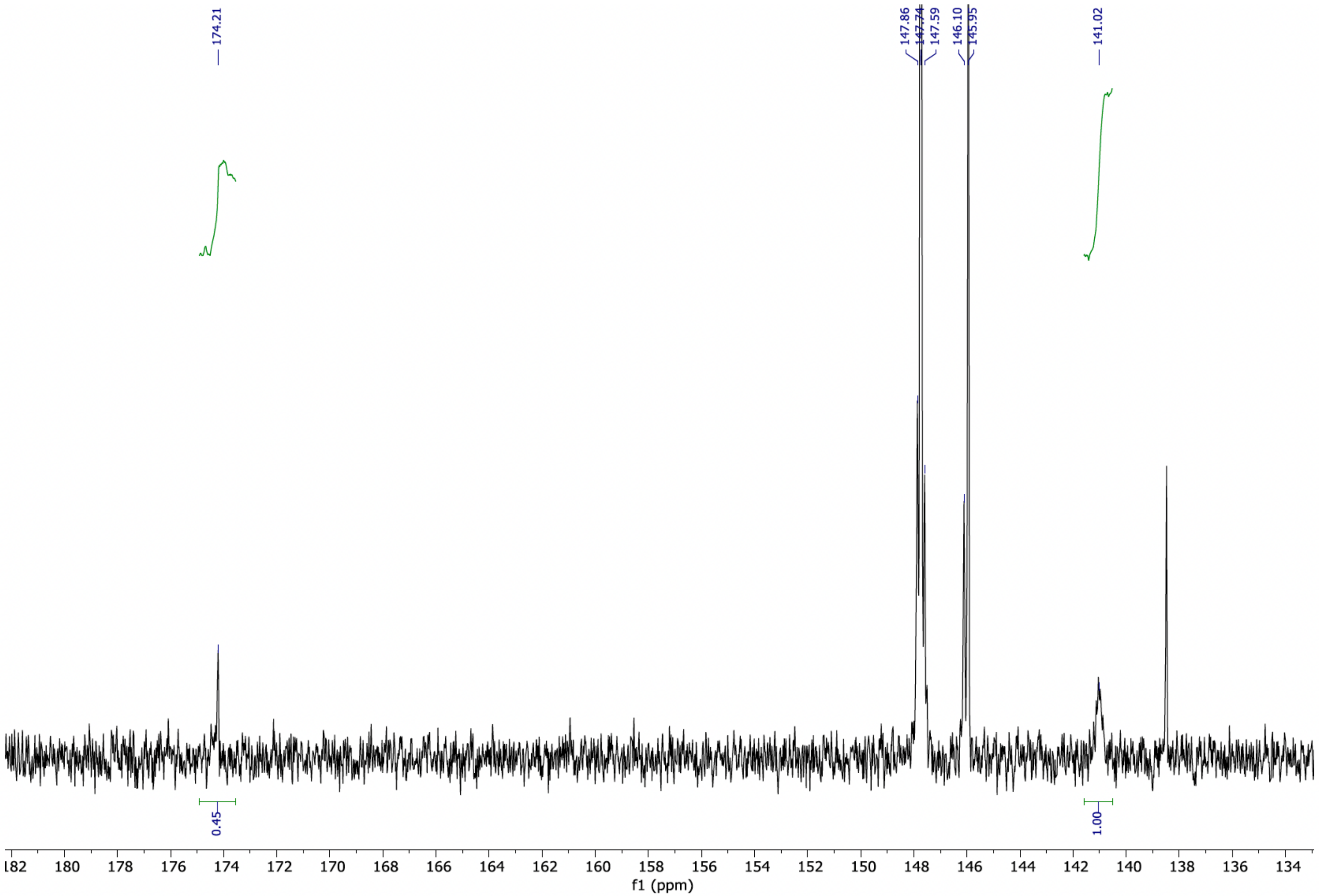

Figure S5 IR spectrum (solid, cm^{-1}) of K[Al(NON)(H)(CN)] (**1a**) / K[Al(NON)(H)(NC)] (**1b**)

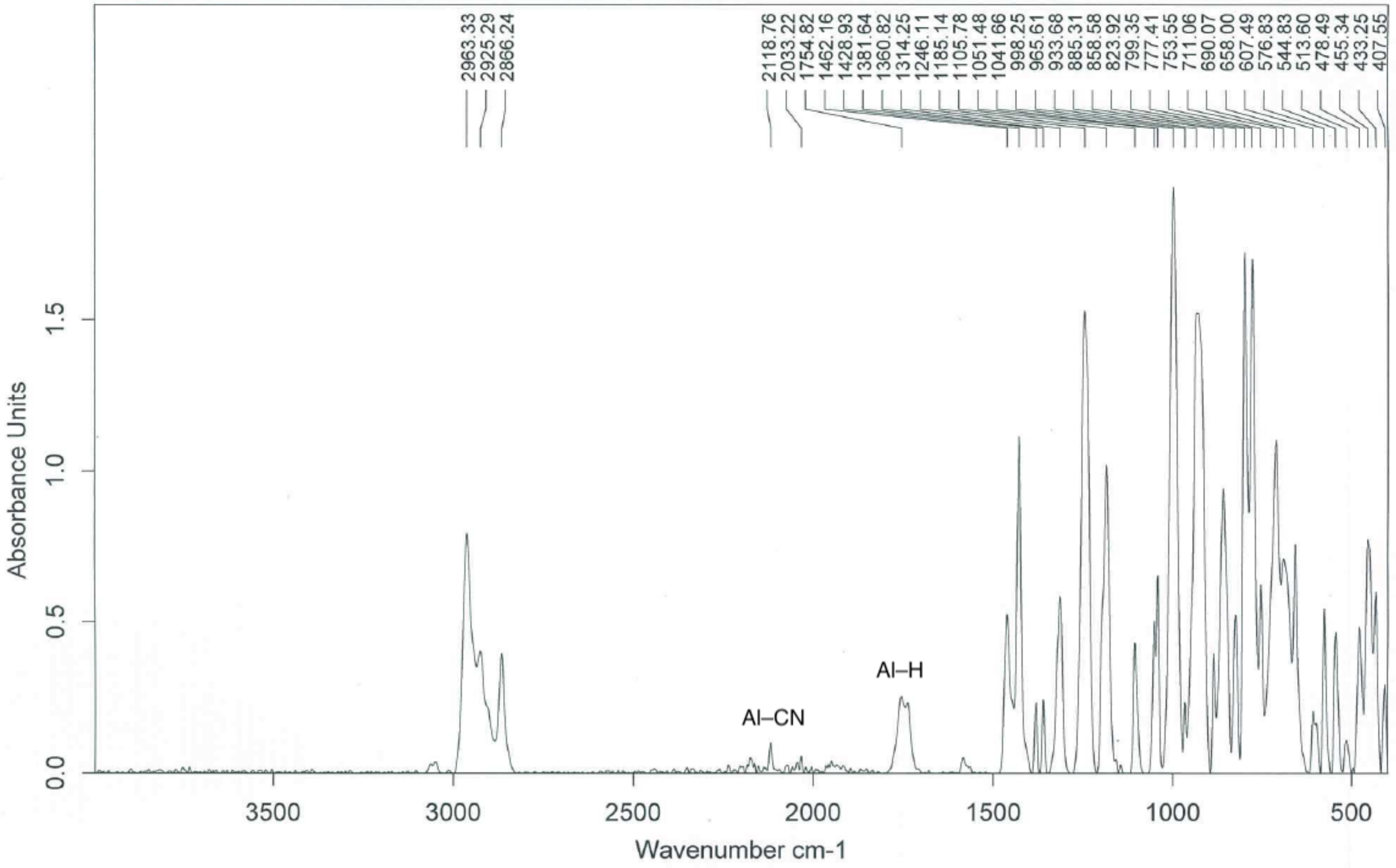

Figure S6 Displacement ellipsoid plot (30 %, H-atoms except Al*H* omitted) of K[Al(NON)(H)(CN)] (**1a**) (*asymmetric unit*)

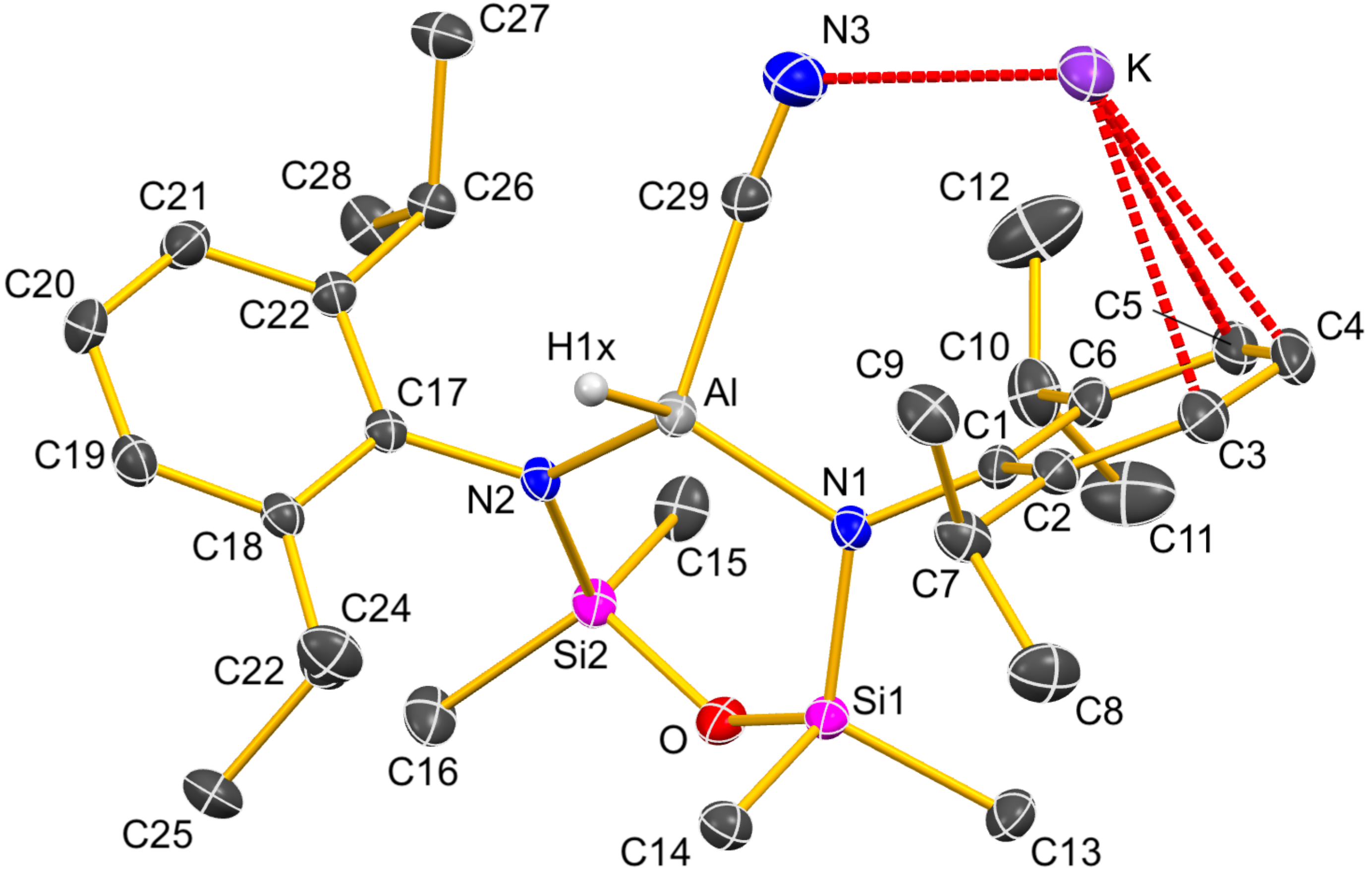

Experimental details for [K(2.2.2)crypt][Al(NON)(H)(CN)] (1a·crypt) / [K(2.2.2)crypt][Al(NON)(H)(NC)] (1b·crypt)

A solution of *tert*-butyl isocyanide (15 mg, 0.19 mmol) in THF (~3 mL) was added to a stirred solution of K{Al(NON)}] (102 mg, 0.19 mmol) in THF (~3 mL) at room temperature. The reaction mixture was allowed to stir for *ca.* 1 hr and 222-cryptand (70 mg, 0.19 mmol) was added. The volatiles were removed *in vacuo* and the residue dissolved in boiling toluene (~3 mL). Crystals suitable for single crystal X-ray diffraction experiments were grown at room temperature *via* slow evaporation. Yield 134 mg, 76 %.

An isomeric mixture exists in solution. Overlap in the ^{1}H NMR spectrum causes most of the peaks to be indistinguishable between the two isomers. However, a noticeable shift is observed between the $SiMe_2$ signals that has been used to calculate the relative ratio between each isomer as 5:3.

Anal. Calcd. for $C_{47}H_{83}AlKN_5O_7Si_2$ (*951.53*): C, 59.27; H, 8.79; N, 7.35 %. Found: C, 58.64; H, 8.91; N, 7.04 %.

^{1}H NMR (500 MHz, THF-D_8): δ 6.97 – 6.85 (m, 4H, C_6H_3), 6.75 (t, J = 7.2, 2H, C_6H_3), 4.14 – 4.10 (m, 4H, C*H*Me$_2$), 3.49 (s, 12H, crypt-CH_2), 3.50 – 3.42 (m, 12H, crypt-CH_2), 2.46 – 2.49 (m, 12H, crypt-CH_2), 1.30 (d, J = 6.8 Hz, 6H, CHMe_2), 1.21 – 1.14 (m, 16H, CHMe_2), 0.10, –0.02 (s, 6H, SiMe_2).

SiMe$_2$ ^{1}H NMR signals for each isomer: 0.11 (s, 6.0H, ***Major****), 0.10 (s, 3.6H,* ***Minor****), –0.02 (s, 6.0H,* ***Major****), –0.03 (s, 3.6H,* ***Minor****).*

^{13}C{^{1}H} NMR (126 MHz, THF-D_8): δ 179.3 (Al–N*C*, **Minor**),* 148.3, 148.2, 147.6, 147.5, 146.5, 146.4, 123.6, 123.5, 123.5, 123.4, 121.9, 121.8 (C_6H$_3$), 71.3, 68.5, 54.8 (crypt-*C*H$_2$), 27.9, 27.7, 27.7, 27.6, 27.5, 27.1, 27.1, 26.0, 26.0, 25.9, 25.9, 25.6 (*C*HMe$_2$ and CHMe_2), 3.3, 3.1, 2.7, 2.6 (SiMe_2).

* The ^{13}C{^{1}H} NMR spectrum only shows a signal for the Al–N*C* ligand (Al–*C*N not observed due to coupling with ^{27}Al nuclei).

IR (solid, cm^{-1}): 2106 (υ_{CN}), 1792 ($\upsilon_{Al\text{-}H}$).

Figure S7 ^{1}H NMR spectrum (500 MHz, THF-D_8) of [K(2.2.2)crypt][Al(NON)(H)(CN)] (**1a·crypt**) / [K(2.2.2)crypt][Al(NON)(H)(NC)] (**1b·crypt**)

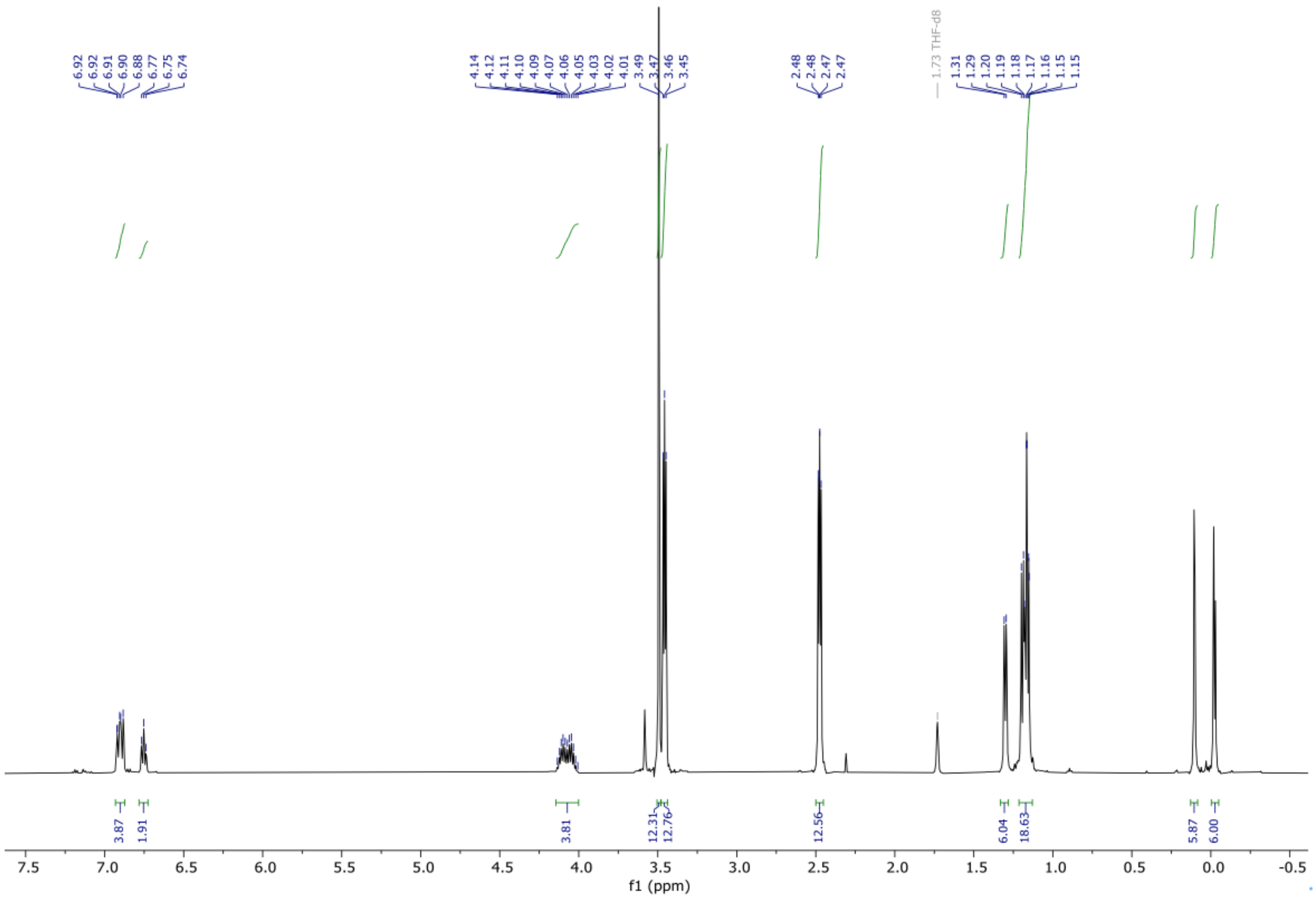

Figure S8 Expansion of Si*Me₂* region of the ^{1}H NMR spectrum (500 MHz, THF-D_8) of [K(2.2.2)crypt][Al(NON)(H)(CN)] (**1a·crypt**) / [K(2.2.2)crypt][Al(NON)(H)(NC)] (**1b·crypt**)

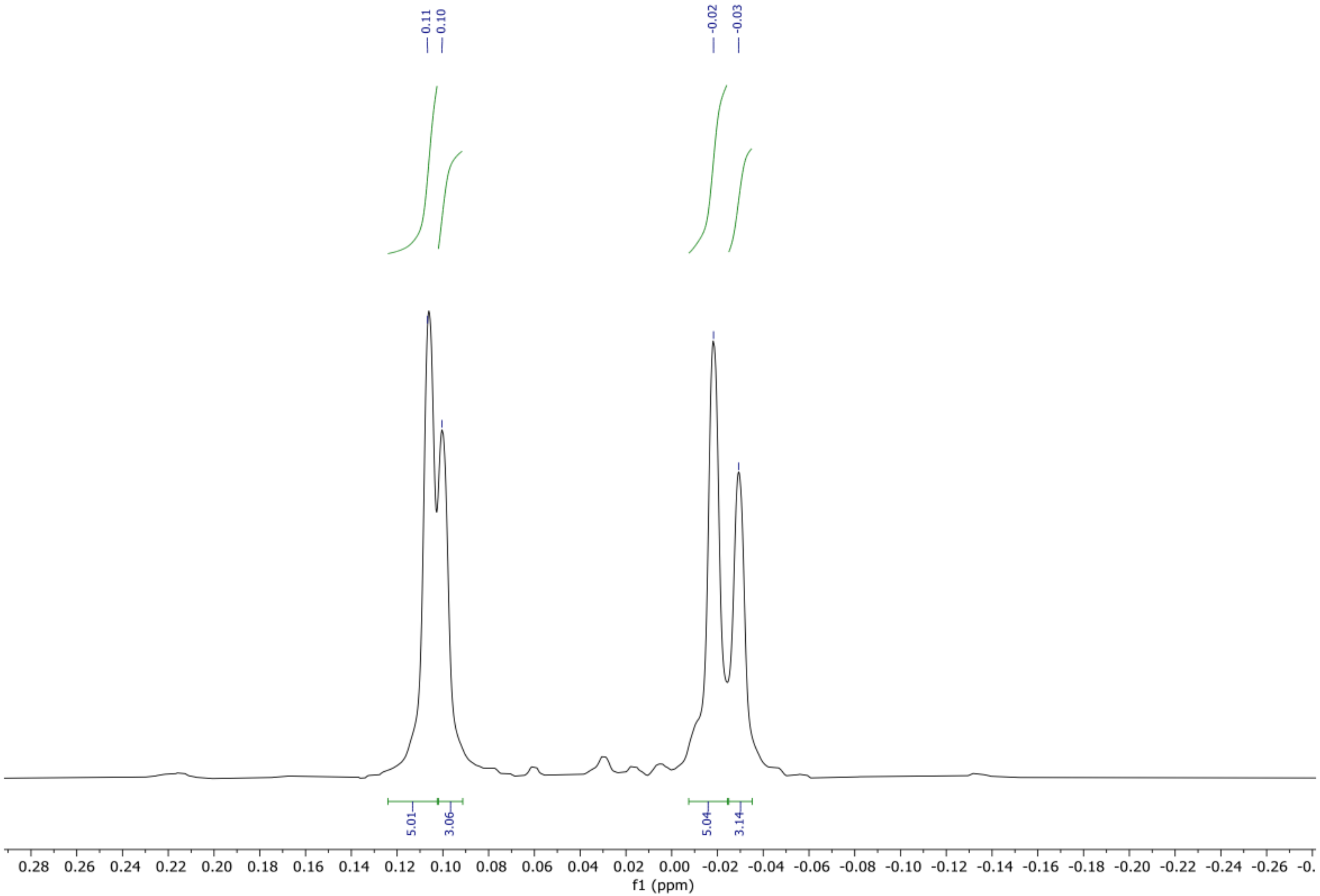

Figure S9 $^{13}C\{^1H\}$ NMR spectrum (500 MHz, THF-D_8) of [K(2.2.2)crypt][Al(NON)(H)(CN)] (**1a·crypt**) / [K(2.2.2)crypt][Al(NON)(H)(NC)] (**1b·crypt**)

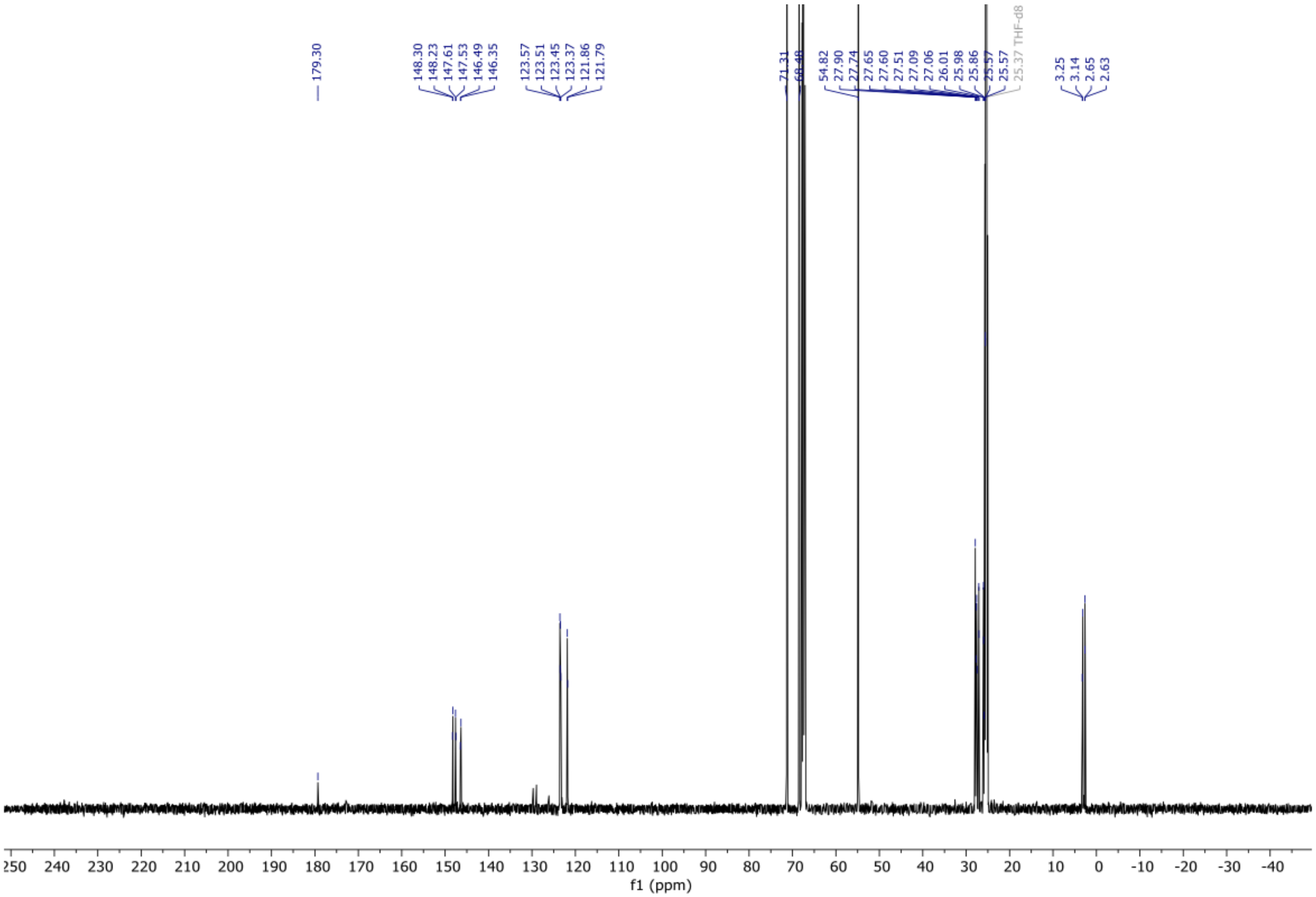

Figure S10 IR spectrum (solid, cm^{-1}) of [K(2.2.2)crypt][Al(NON)(H)(CN)] (**1a·crypt**) / [K(2.2.2)crypt][Al(NON)(H)(NC)] (**1b·crypt**)

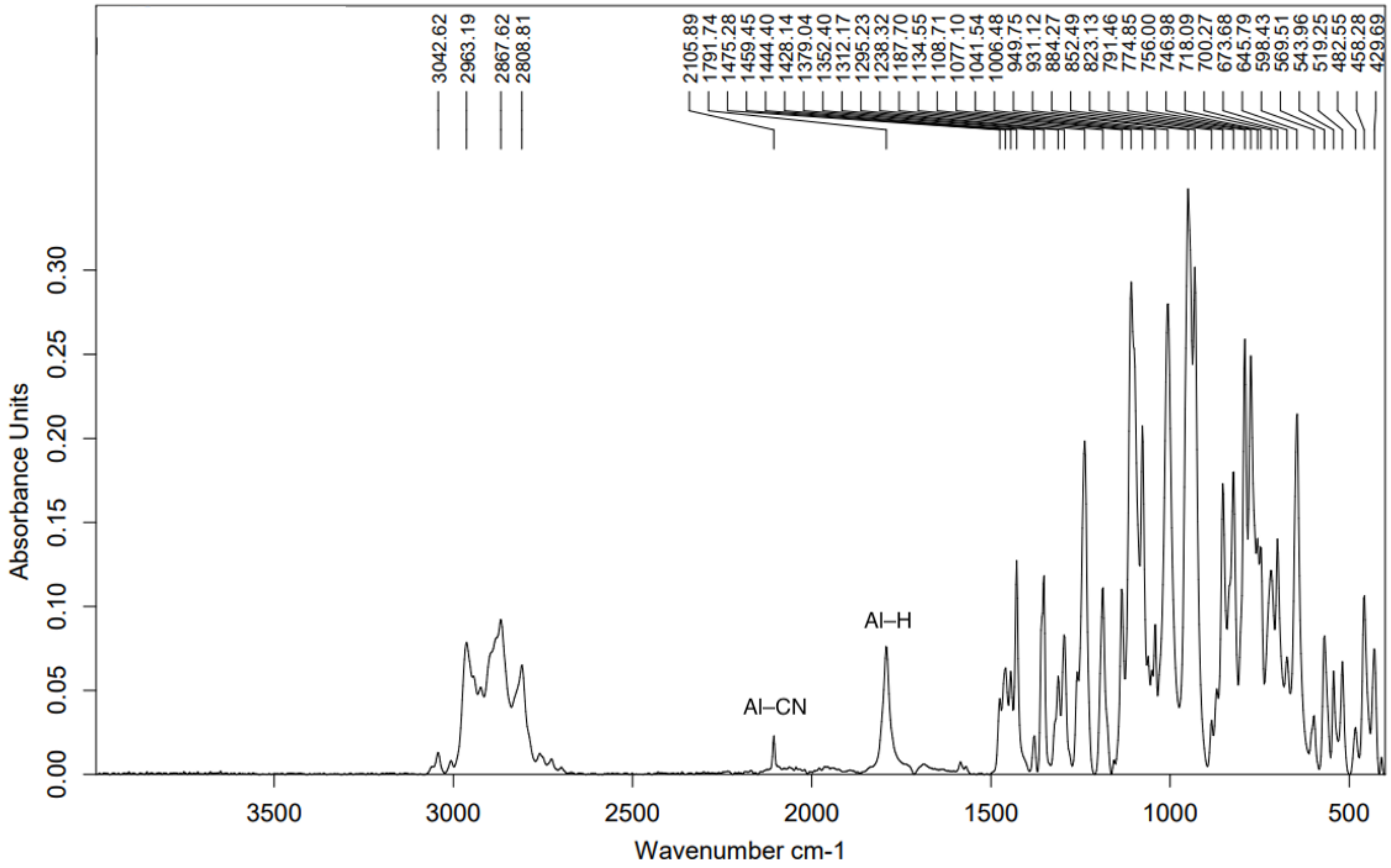

Figure S11 Displacement ellipsoid plot (30 %, H-atoms except Al*H* and disordered atoms omitted) of [K(2.2.2)crypt][Al(NON)(H)(CN)] (**1a·crypt**) (*asymmetric unit*)

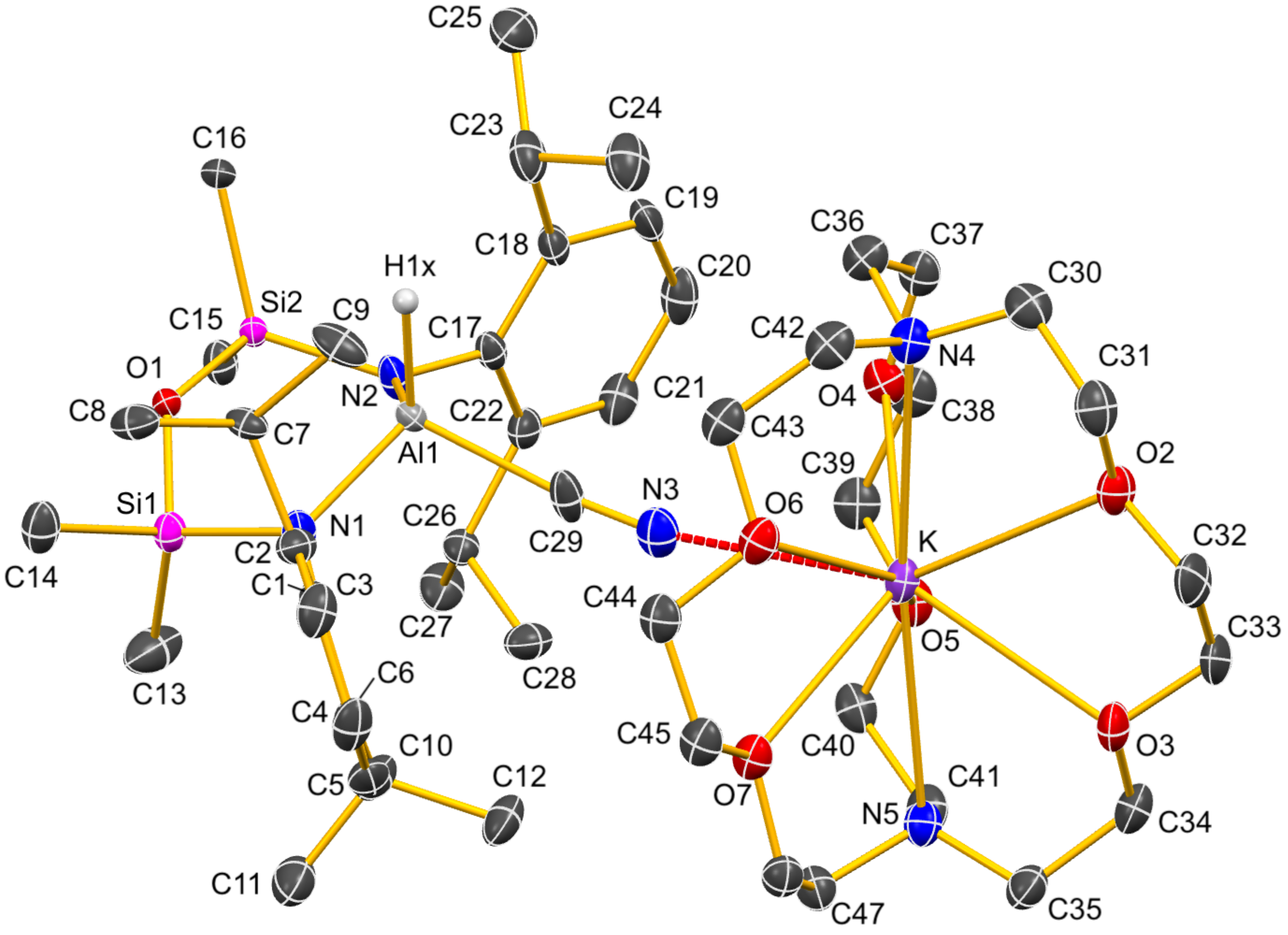

Experimental details for [K(Et_2O)$_2$][Al(NON){(DmpNC)$_2$(DmpNC*)}] (2·Et_2O) (* = ring-activated DmpNC)

A solution of 2,6-dimethylphenyl isocyanide (130 mg, 0.99 mmol) in toluene was added drop wise to a stirred solution of K[Al(NON)] (182 mg, 0.33 mmol) in an ampoule at –78 °C. The resulting dark red/purple mixture was allowed to warm to room temperature and the volatiles removed *in vacuo*. Crystallisation was achieved from a solution of toluene or diethyl ether *via* slow evaporation at room temperature. An analytically pure purple powder was obtained by washing the crystals with hexane (5 x 2 mL). Yield 211 mg, 68 %.

Anal. Calcd. for $C_{63}H_{93}AlKN_5O_3Si_2$ (*1089.63*): C, 69.38; H, 8.60; N, 6.43 %. Found: C, 68.50; H, 8.45; N, 6.50 %.

^{1}H NMR (500 MHz, THF-D_8): δ 6.98 (d, *J* = 7.8, 1H, C_6H_3), 6.95 (d, *J* = 7.7, 1H, C_6H_3), 6.89 (m, 3H, C_6H_3),‡ 6.87 – 6.79 (m, 2H, C_6H_3), 6.76 (t, *J* = 7.5, 1H, C_6H_3), 6.47 (d, *J* = 7.3, 1H, C_6H_3), 6.44 (d, *J* = 7.3, 1H, C_6H_3), 6.33 (t, *J* = 7.4, 1H, C_6H_3), 5.79 (d, *J* = 6.0, 1H, C_6H_3),* 5.71 – 5.62 (m, 2H, C_6H_3),* 4.02 (sept, *J* = 7.3, 1H, C*H*Me$_2$), 3.69 – 3.54 (m, 3H, C*H*Me$_2$), 3.39 (q, *J* = 7.0, 4H, Et_2O),¥ 2.53 (s, 3H, C_6H_3*Me$_2$*), 2.22 (s, 3H, C_6H_3*Me$_2$*), 1.95 (s, 3H, C_6H_3*Me$_2$*), 1.39 (d, *J* = 6.5, 3H, CH*Me$_2$*), 1.36 (s, 3H, C_6H_3*Me$_2$*), 1.24 (d, *J* = 6.8, 3H, CH*Me$_2$*), 1.15 (d, *J* = 6.7, 3H, CH*Me$_2$*), 1.12 (t, *J* = 7.0, 6H, Et_2O),¥ 1.06 (d, *J* = 4.2, 3H, CH*Me$_2$*), 1.05 (d, *J* = 4.2, 3H, CH*Me$_2$*), 0.95 (s, 3H, C_6H_3*Me$_2$*),* 0.93 (s, 3H, C_6H_3*Me$_2$*),* 0.77 (d, *J* = 6.5, 3H, CH*Me$_2$*), 0.72 (d, *J* = 6.8, 3H, CH*Me$_2$*), 0.40 (d, *J* = 6.8, 3H, CH*Me$_2$*), 0.38 (s, 3H, Si*Me$_2$*), 0.26 (s, 3H, Si*Me$_2$*), –0.13 (s, 3H, Si*Me$_2$*), –0.15 (s, 3H, Si*Me$_2$*).

^{13}C{^{1}H} NMR (126 MHz, THF-D_8): δ 167.4 (C=*C*=N), 156.3,* 152.6, 151.3, 148.5, 148.0, 147.9, 147.3, 144.3, 143.2 (*C$_6$*H$_3$), 135.1 (C=*C*=C), 133.6,* 131.3, 129.1, 128.9, 128.8, 128.8, 128.5, 128.4, 128.1, 127.5, 124.7, 124.6, 124.4, 123.8, 123.5,* 123.3, 123.1, 122.4,* 122.3,* 120.5 (*C$_6$*H$_3$), 66.4 (Et_2O),¥ 63.4 (*C$_6$*H$_3$),* 29.8, 28.8 (CH*Me$_2$*), 28.6 (*C*HMe$_2$), 28.6 (C_6H_3*Me$_2$*), 28.2 (CH*Me$_2$*), 28.0 (*C*HMe$_2$), 27.8 (CH*Me$_2$*), 27.6, 27.3 (*C*HMe$_2$), 27.1, 26.3, 25.9, 24.0 (CH*Me$_2$*), 21.1, 20.1, 19.8, 18.2, 18.1 (C_6H_3*Me$_2$*), 15.7 (Et_2O),¥ 5.4, 5.1, 1.4, 1.3 (Si*Me$_2$*).

* activated ring system; ‡ overlapping signals causing observed splitting pattern; ¥ one equivalent of Et_2O present after crystals dried under vacuum.

Figure S12 ^{1}H NMR spectrum (500 MHz, THF-D_8) of $[K(Et_2O)_2][Al(NON)\{(DmpNC)_2(DmpNC^*)\}]$ (**2·Et₂O**) (* = ring-activated DmpNC).

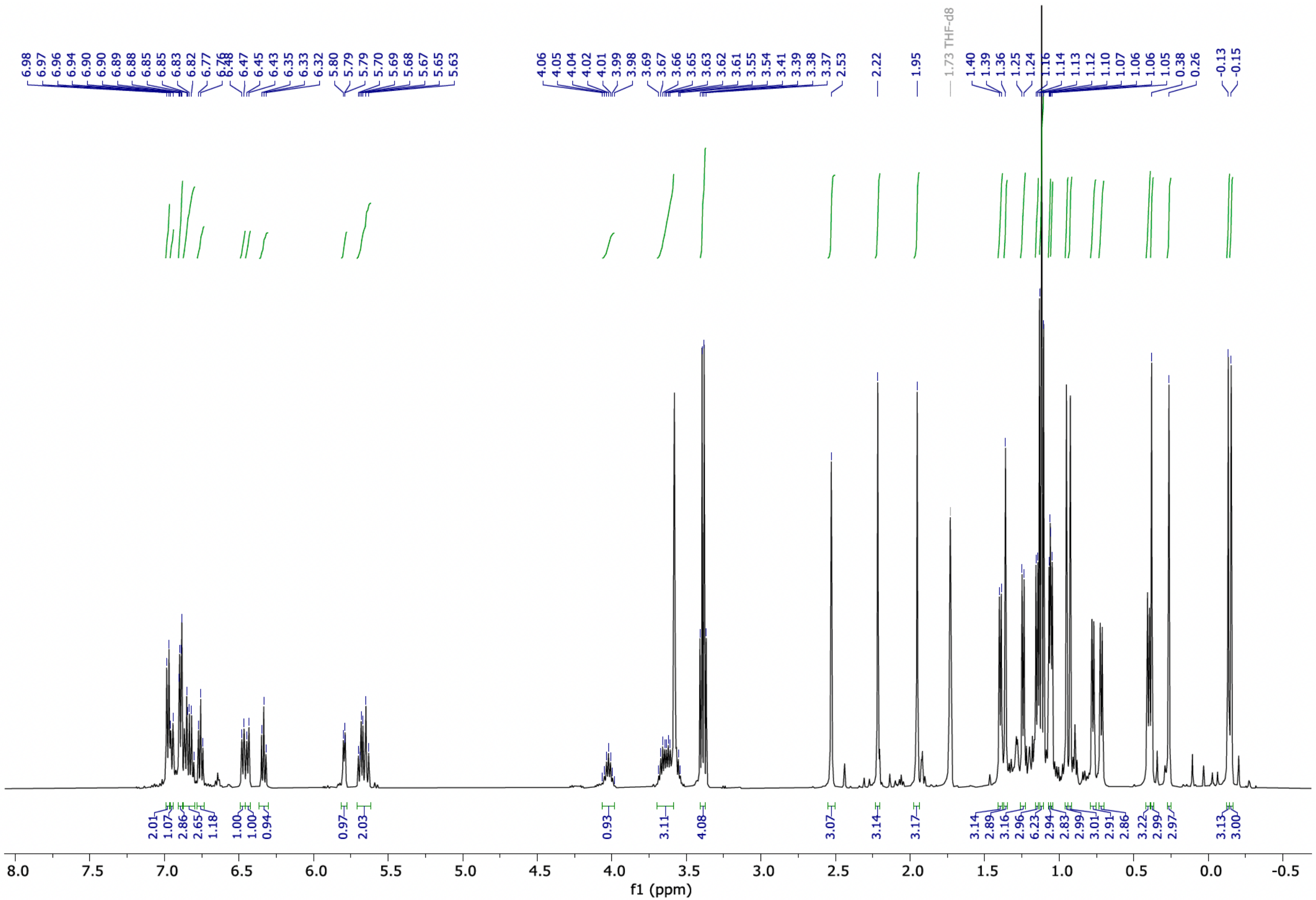

Figure S13 $^{13}C\{^{1}H\}$ NMR spectrum (126 MHz, THF-D$_8$) of $[K(Et_2O)_2][Al(NON)\{(DmpNC)_2(DmpNC^*)\}]$ (**2·Et₂O**) (* = ring-activated DmpNC).

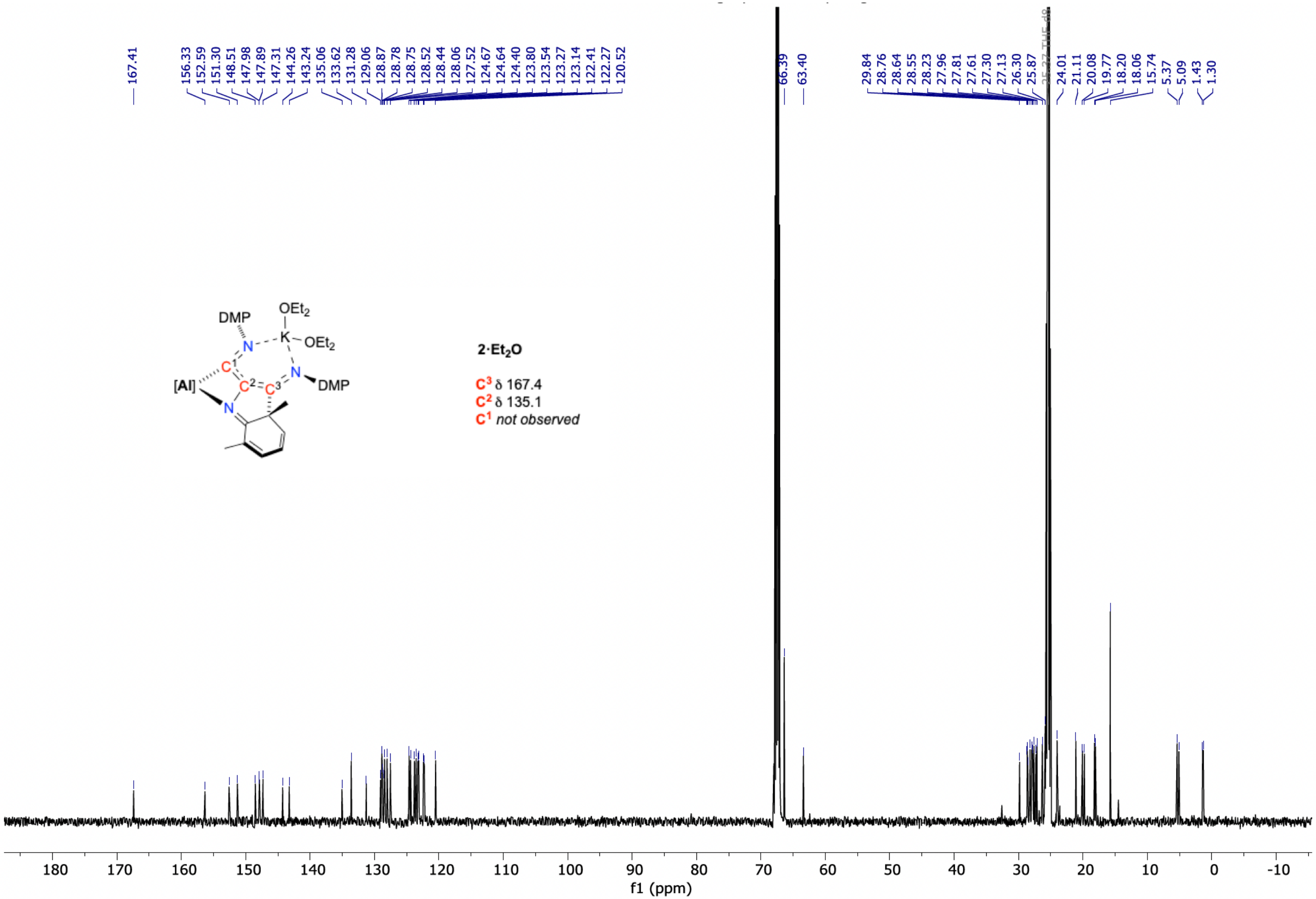

Figure S14 Displacement ellipsoid plot (30 %, H-atoms omitted) of $[K(Et_2O)_2][Al(NON)\{(DmpNC)_2(DmpNC^*)\}]$ (**2·Et$_2$O**) (* = ring-activated DmpNC).

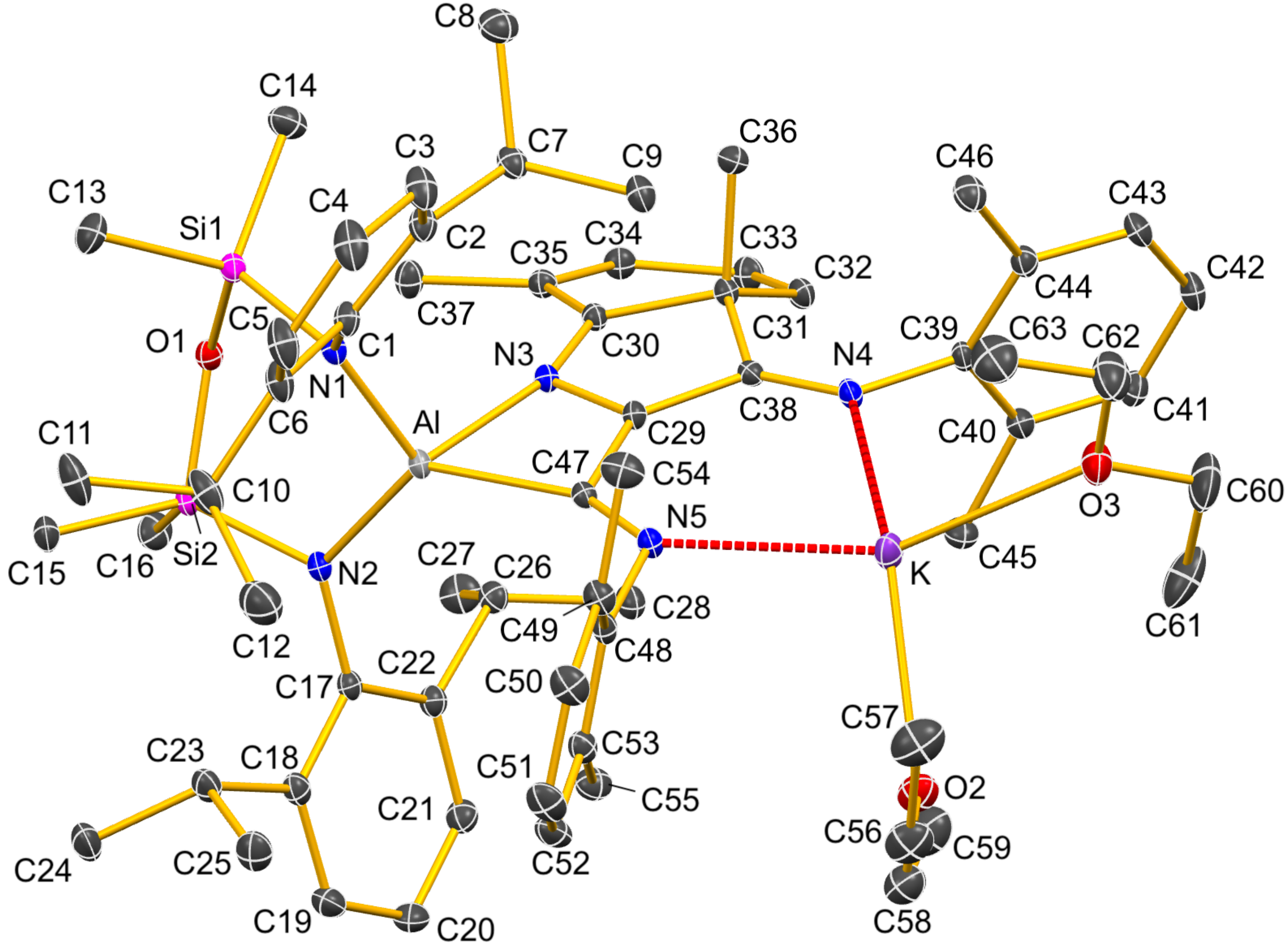

Figure S15 Displacement ellipsoid plot (30 %, H-atoms , disordered atoms and toluene solvate molecules omitted) of K[Al(NON){(DmpNC)$_2$(DmpNC*)}] (**2·toluene**) (* = ring-activated DmpNC).

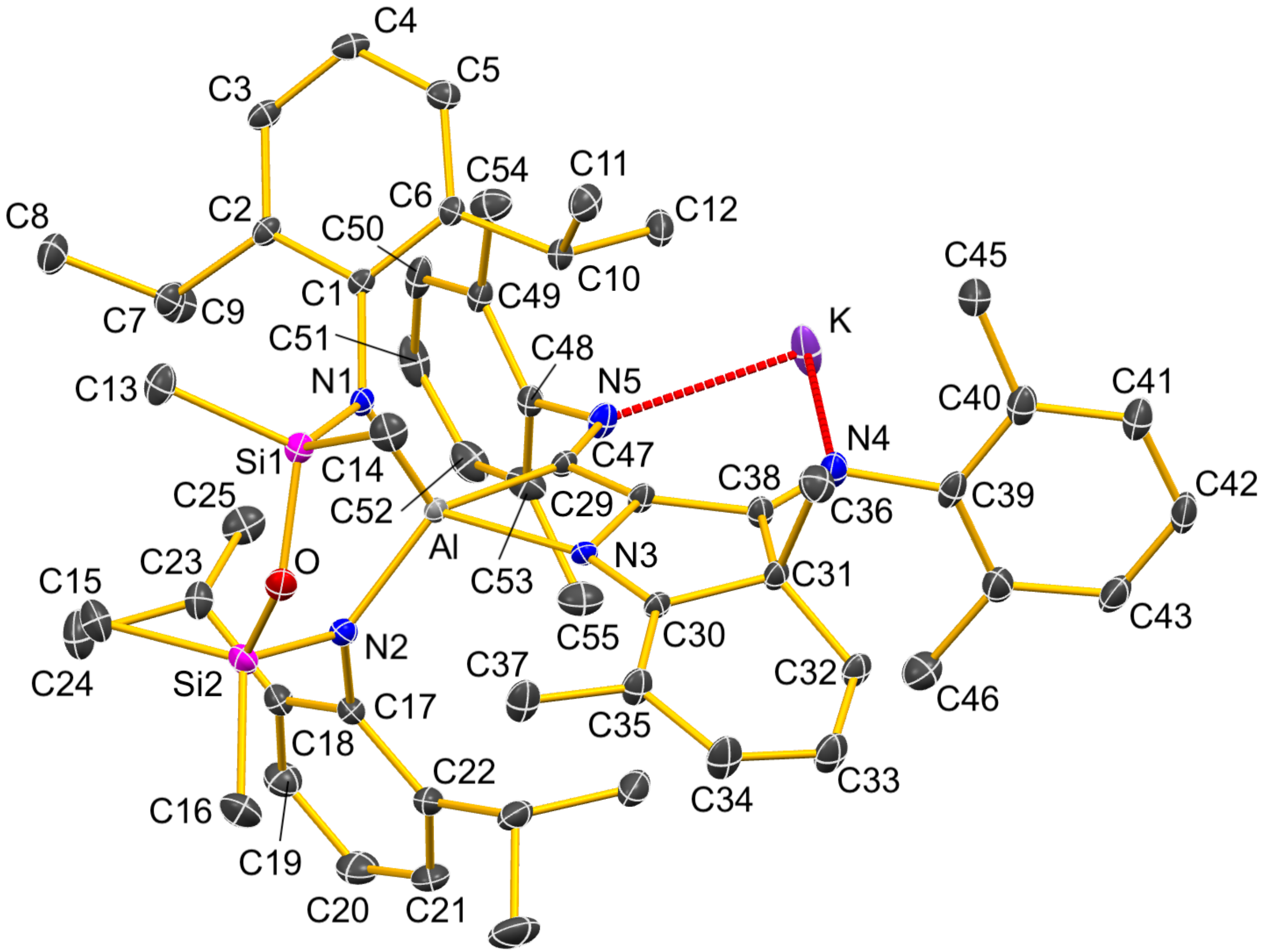

Experimental details for [K(Et_2O)][Al(NON)($AdNC)_2$] (3·Et_2O)

A solution of 1-adamantyl isocyanide (52 mg, 0.32 mmol) in toluene (~5 mL) was added to a stirred solution of K[Al(NON)] (89 mg, 0.16 mmol) at room temperature. The reaction mixture was allowed to stir for *ca.* 1 hr to give a bright yellow solution. The solvent was removed *in vacuo* and the residue dissolved in diethyl ether. Crystallisation was achieved by slow evaporation of a diethyl ether solution stored at room temperature. Yield 120 mg, 85 %.

Anal. Calcd. for $C_{54}H_{86}AlKN_4O_2Si_2$ (*944.57*): C, 68.60; H, 9.18; N, 5.93 %. Found: C, 69.87; H, 8.89; N, 5.49 %.

^{1}H NMR (500 MHz, Toluene-D_8, 373 K*): δ 7.13 – 7.09 (m, 3H, C_6H_3), 6.84 (d, *J* = 7.6, 1H, C_6H_3), 6.78 (d, *J* = 7.6, 1H, C_6H_3), 6.62 (t, *J* = 7.6, 1H, C_6H_3), 4.20 – 3.92 (m, 4H, C*H*Me$_2$), 3.27 (q, *J* = 7.0, 14H, Et_2O), 1.91 (br s, 4H, Ad), 1.81 (br s, 2H, Ad), 1.74 – 1.67 (m, 16H, Ad), 1.61 (d, *J* = 6.8, 3H, CH*Me$_2$*), 1.56 (s, 2H, Ad), 1.54 (d, *J* = 6.8, 3H, CH*Me$_2$*), 1.46 (br d, *J* = 11.0, 2H, Ad), 1.38 (d, *J* = 6.8, 3H, CH*Me$_2$*), 1.36 – 1.30 (m, 2H, Ad), 1.29 (d, *J* = 6.8, 3H, CH*Me$_2$*), 1.23 (d, *J* = 6.8, 3H, CH*Me$_2$*), 1.07 (t, *J* = 7.0, 20H, Et_2O), 0.97 (br d, *J* = 11.0, 2H, Ad), 0.74 (s, 3H, Si*Me$_2$*), 0.65 (s, 3H, Si*Me$_2$*), 0.08 (s, 3H, Si*Me$_2$*), –0.14 (s, 3H, Si*Me$_2$*).

^{13}C{^{1}H} NMR (126 MHz, Toluene-D_8, 373 K*): δ 196.1 (C=*C*=N), 151.1, 149.4, 149.2, 147.3, 146.1, 145.8, 124.0, 123.8, 123.2, 123.1, 122.1 (*C$_6$*H$_3$), 65.9 (Et_2O), 55.3,‡ 45.4,‡ 43.8,‡ 37.7,‡ 31.1, 30.8 (Ad), 28.7,‡ 27.9, 27.4 (*C*HMe$_2$), 27.1, 26.8, 26.3, 25.9, 25.7, 25.6, 25.5, 24.8 (CH*Me$_2$*), 15.5 (Et_2O), 5.2,‡ 1.1, 0.1 (Si*Me$_2$*).

* NMR spectra recorded at 373 K due to poor solubility in toluene-D_8; ‡ overlapping signal correspond to more than one ^{13}C peak; Al–*C* resonance not observed.

Figure S16 ^{1}H NMR spectrum (500 MHz, Toluene-D$_8$, 373 K) of [K(Et$_2$O)][Al(NON)(AdNC)$_2$] (**3·Et$_2$O**).

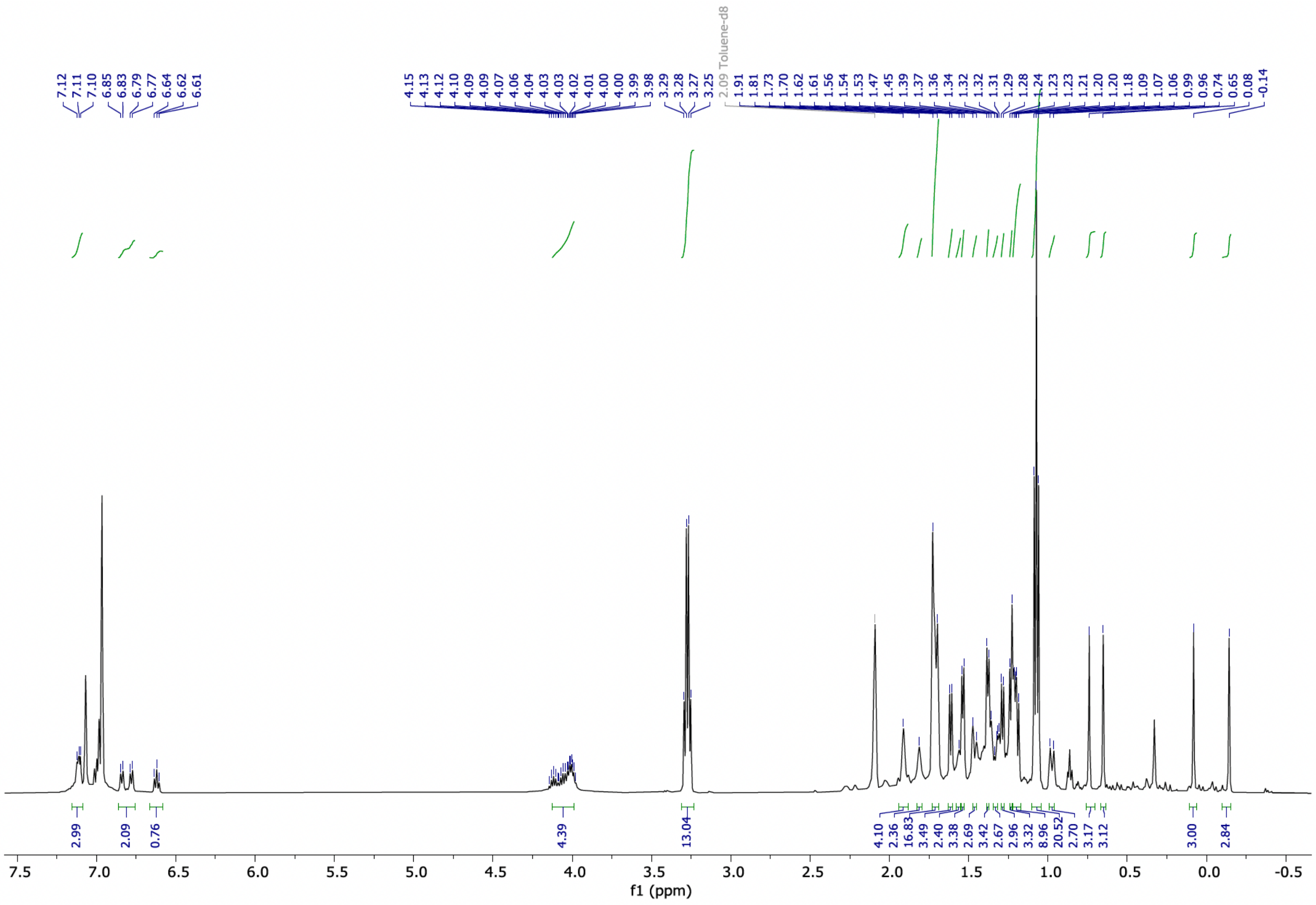

Figure S17 $^{13}C\{^{1}H\}$ NMR spectrum (126 MHz, Toluene-D_8, 373 K) of [K(Et_2O)][Al(NON)($AdNC)_2$] (**3·Et₂O**).

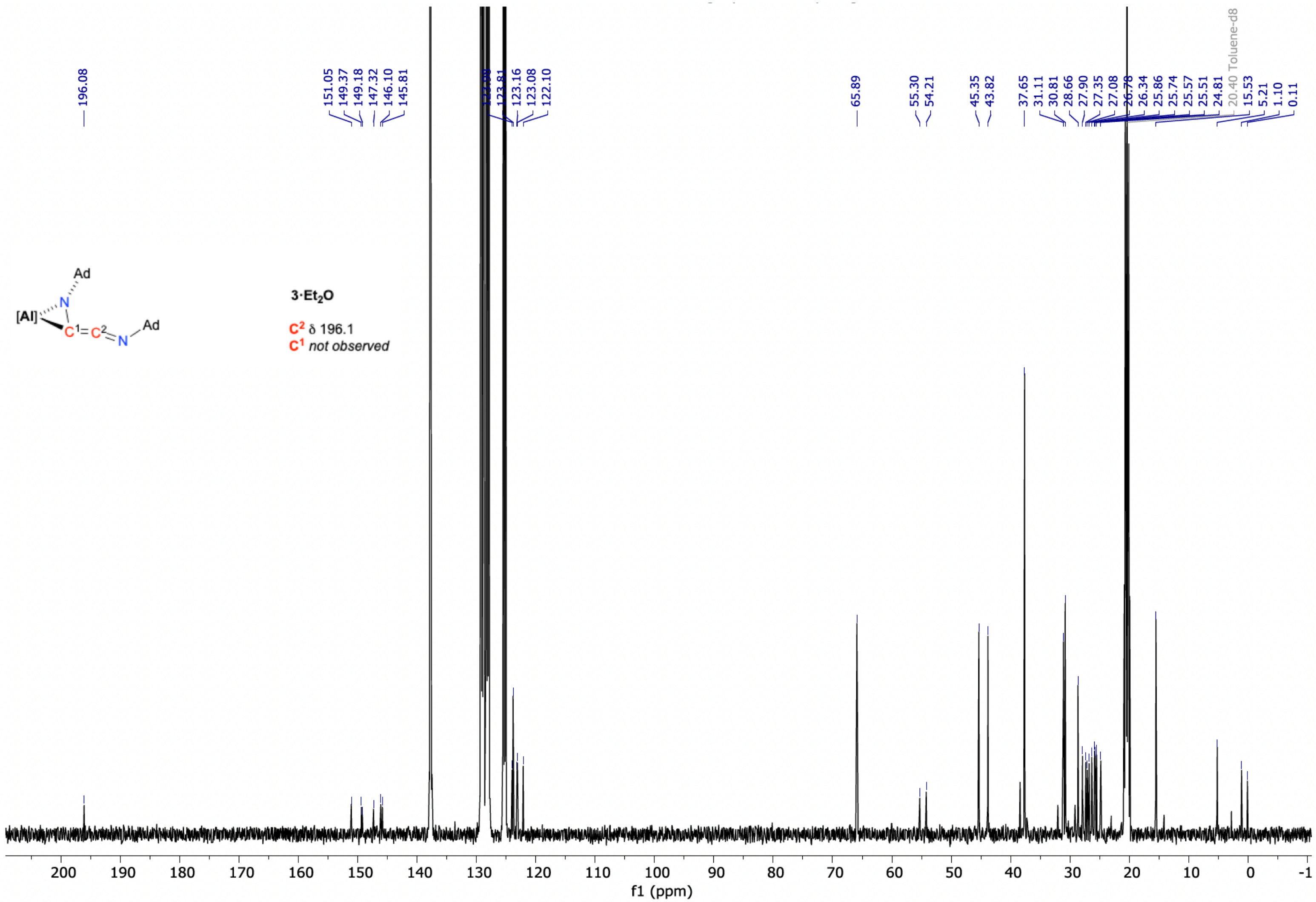

Figure S18 Displacement ellipsoid plot (30 %, H-atoms omitted) of [K(Et_2O)][Al(NON)(AdNC)$_2$] (**3·Et_2O**)

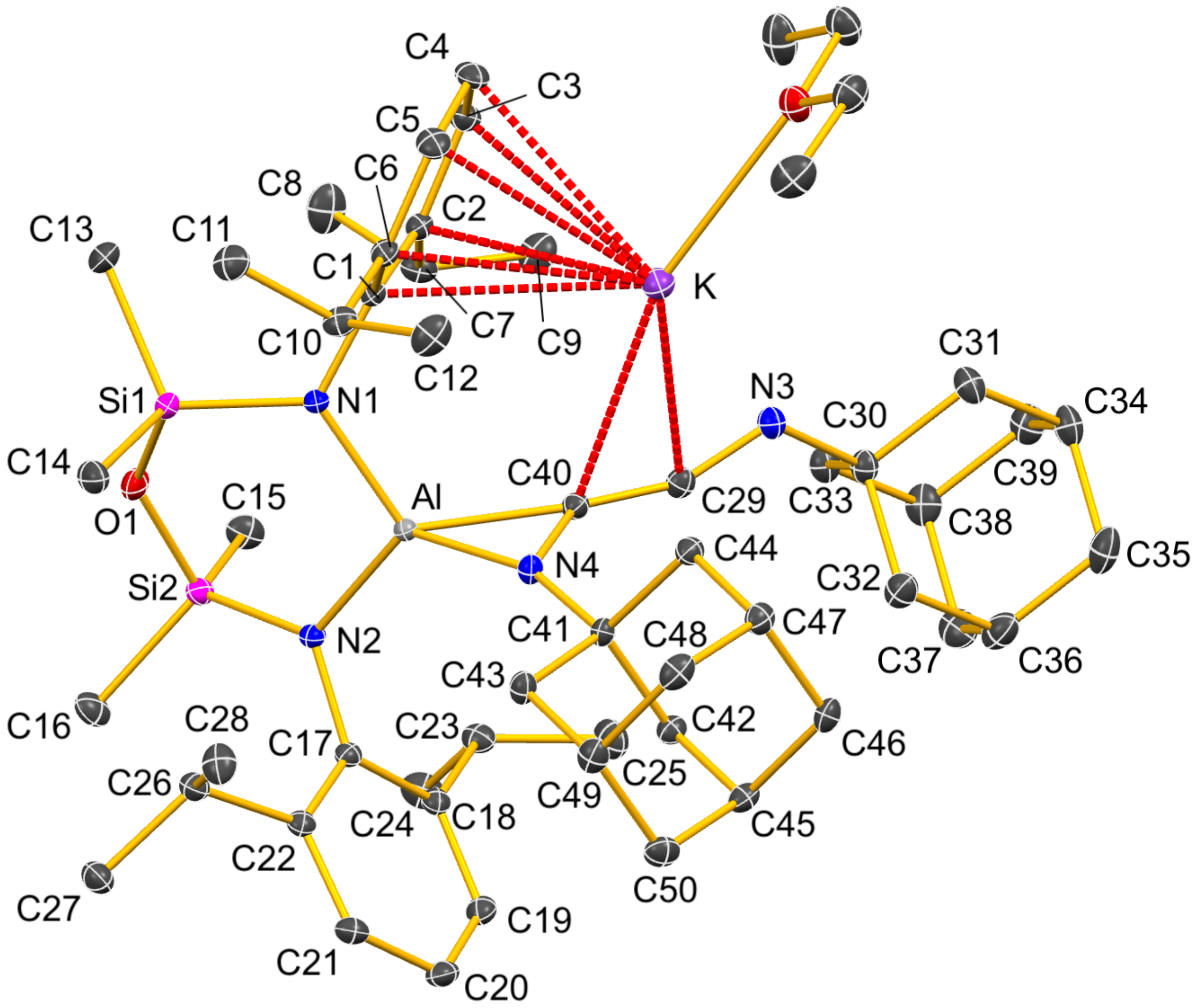

Experimental Details for [K(THF)$_2$][Al(NON)(AdNC)$_2$(DmpNC)] (4·THF)

A solution of [K(Et$_2$O)$_2$][Al(NON)(AdNC)$_2$] (76 mg, 0.09 mmol) in THF-D$_8$ was added to a solution of 2,6-dimethylphenyl isonitrile (12 mg, 0.09 mmol) in a J Youngs fitted NMR tube. The mixture was allowed to react for 18 hours to give a dark red solution. The solution was transferred to a scintillation vial and the solvent removed *in vacuo*. Crystallisation was achieved by slow evaporation from a hexane/diethyl ether mixture stored at room temperature. Yield 70 mg, 75 %.

While the broadness of signals in the NMR spectra thwarted accurate spectroscopic characterisation, the observed signals do agree with the proposed structure determined from single crystal X-ray diffraction experiments.

Anal. Calcd. for $C_{67}H_{101}AlKN_5O_3Si_2$ (*1145.70*): C, 70.18; H, 8.88; N, 6.11 %. Found: C, 70.01; H, 8.74; N, 5.93 %.

^{1}H NMR (500 MHz, THF-D$_8$): δ 6.94 – 6.84 (m, 4H, C$_6$*H$_3$*), 6.76 – 6.67 (m, 2H, C$_6$*H$_3$*), 6.49 (br d, *J* = 7.3, 2H, C$_6$*H$_3$*), 6.32 (t, *J* = 7.3, 1H, C$_6$*H$_3$*), 4.10 (br sept, 4H, CH*Me$_2$*), 1.86 – 1.76 (m, 6H, *Ad*), 1.70 – 1.15 (m, 48H, *Ad*, CH*Me$_2$*, C$_6$H$_3$*Me$_2$*), 1.08 (br d, 6H, CH*Me$_2$*), 0.31 (s, 3H, Si*Me$_2$*), 0.26 (s, 3H, Si*Me$_2$*), 0.10 (s, 3H, Si*Me$_2$*), –0.02 (s, 3H, Si*Me$_2$*).

^{1}H NMR (500 MHz, THF-D$_8$, 333 K): δ 6.91 (dd, *J* = 14.8, 7.4, 4H, C$_6$*H$_3$*), 6.74 (t, *J* = 7.4, 2H, C$_6$*H$_3$*), 6.49 (d, *J* = 7.4, 2H, C$_6$*H$_3$*), 6.34 (t, *J* = 7.4, 1H, C$_6$*H$_3$*), 4.23 (br sept, 2H, C*H*Me$_2$), 4.02 (br sept, 2H, C*H*Me$_2$), 1.83 (br s, 6H, *Ad*), 1.59 – 1.40 (m, 22H, Ad, C$_6$H$_3$*Me$_2$*), 1.33 – 1.05 (m, 32H, Ad, CH*Me$_2$*),* 0.16 (br s, 12H, Si*Me$_2$*).

* overlapping doublets: 1.28 (d, *J* = 6.6), 1.20 (d, *J* = 6.7), 1.12 (d, *J* = 6.6).

^{13}C{^{1}H} NMR (126 MHz, THF-D$_8$): 209.5, 153.0 (N*C*), 148.5, 146.8, 127.0, 124.1, 123.9, 123.4, 121.7, 119.4 (*C$_6$*H$_3$), 59.0, 51.3, 43.6, 43.2, 38.0, 37.6, 31.1, 28.5, 28.3, 27.9, 27.7, 27.5, 18.9 (*Ad*, CH*Me$_2$*, C$_6$H$_3$*Me$_2$*), 4.8, 4.2, 3.5, 2.7 (Si*Me$_2$*).

Figure S19 ^{1}H NMR spectrum (500 MHz, THF-D_8) of [K(THF)$_2$][Al(NON)(AdNC)$_2$(DmpNC)] (**4·THF**).

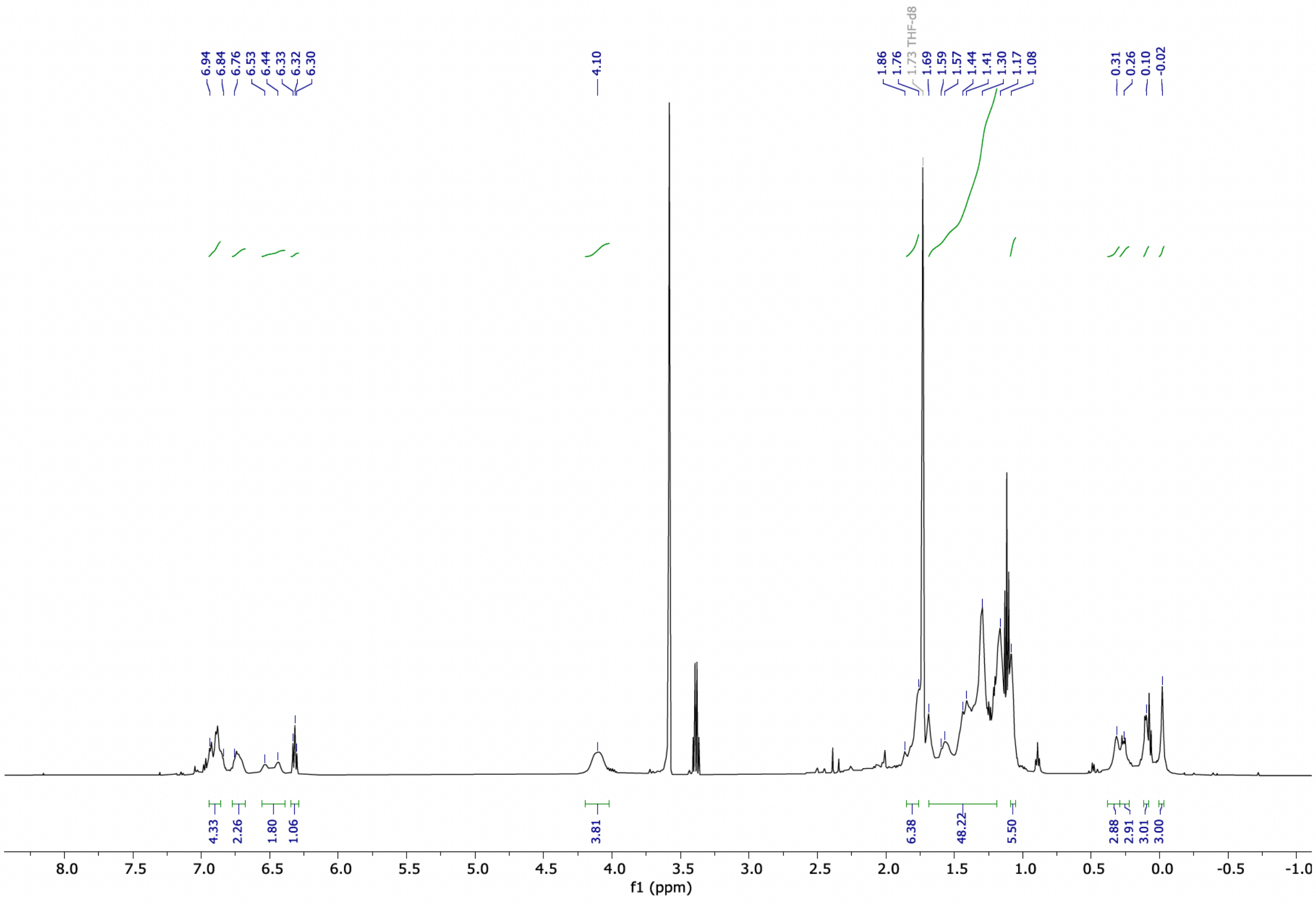

Figure S20 ^{1}H NMR spectrum (500 MHz, THF-D_8, 333 K) of [K(THF)$_2$][Al(NON)(AdNC)$_2$(DmpNC)] (**4·THF**).

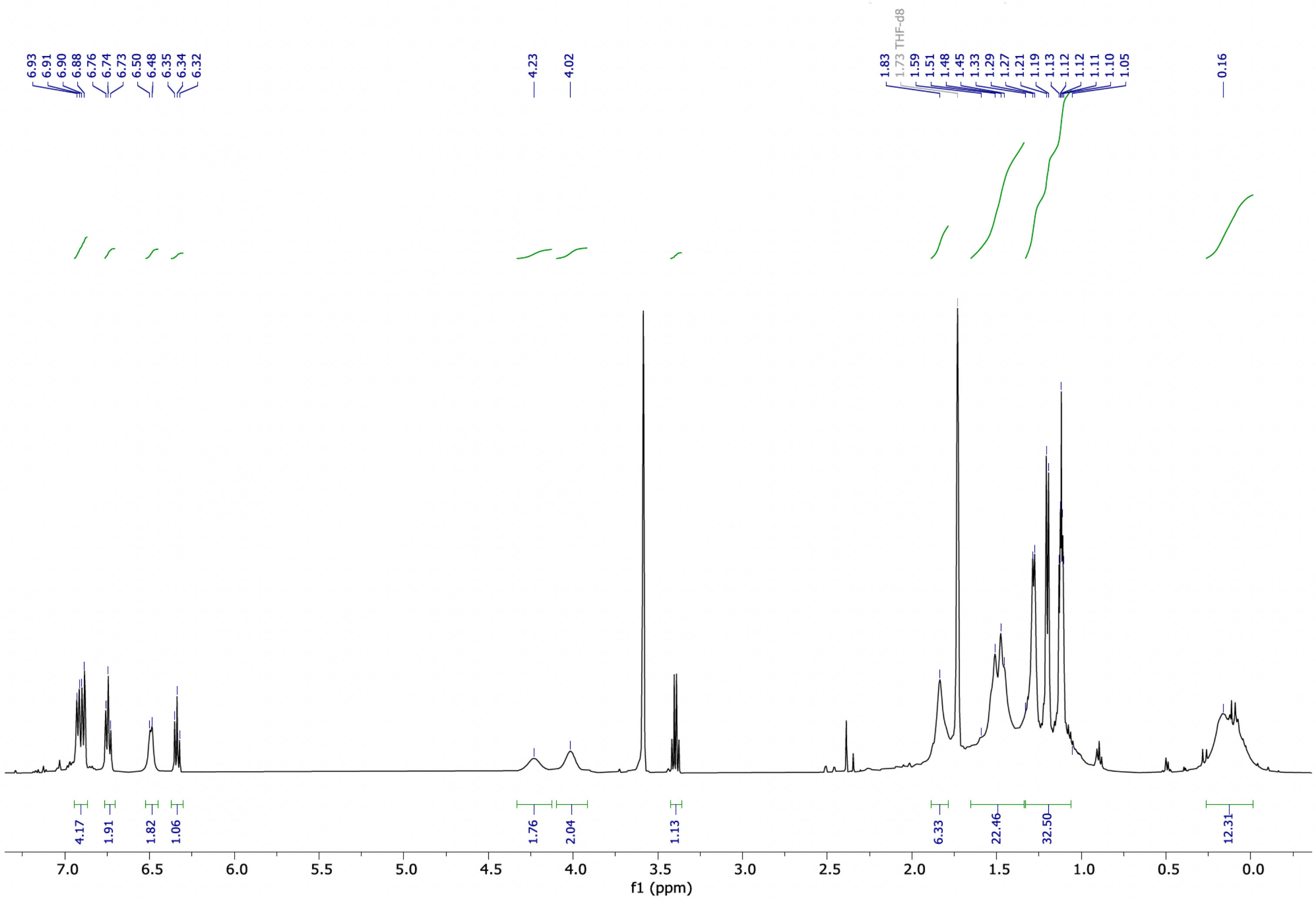

Figure S21 $^{13}C\{^1H\}$ NMR spectrum (126 MHz, THF-D_8, 333 K) of $[K(THF)_2][Al(NON)(AdNC)_2(DmpNC)]$ (**4·THF**).

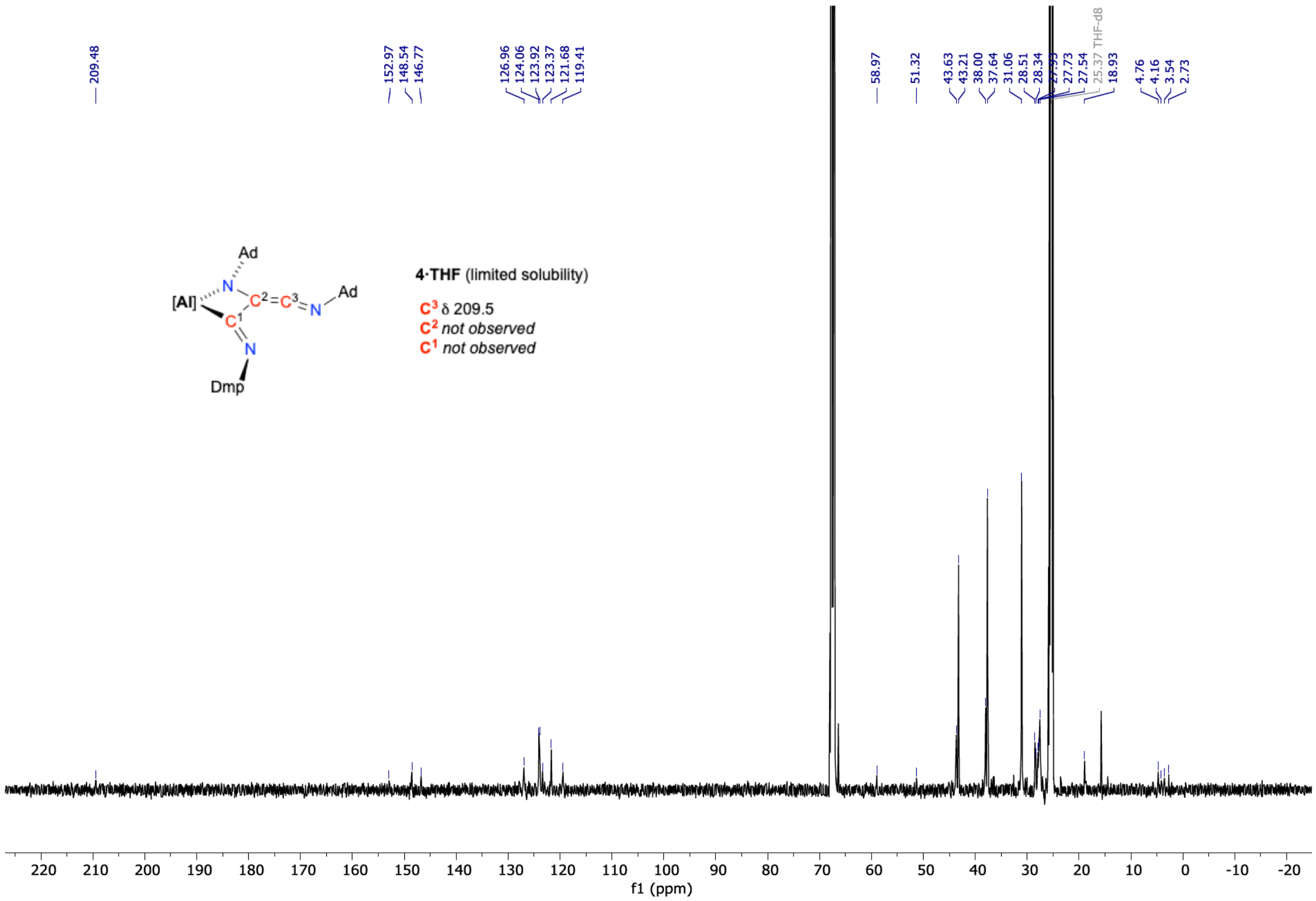

Figure S22 Displacement ellipsoid plot (20 %, H-atoms and disordered components omitted) of [K(THF)$_2$][Al(NON)(AdNC)$_2$(DmpNC)] (**4·THF**).

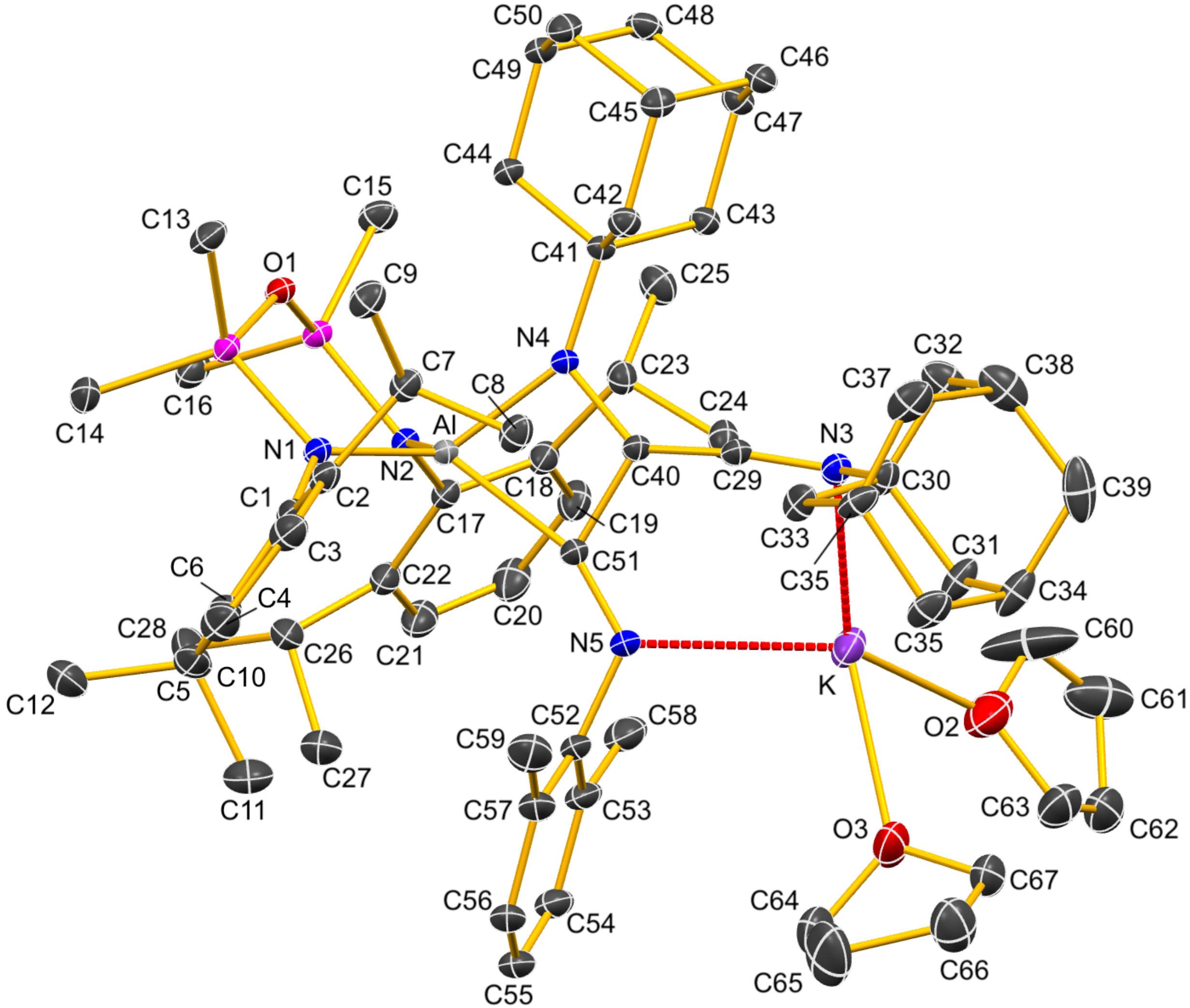

Experimental Details for [K(toluene)][Al(NON)(AdNC)$_3$] (5·toluene)

A solution of 1-adamantyl isocyanide (49 mg, 0.30 mmol) in THF (~5 mL) was added to a stirred solution of [K{Al(NON)}]$_2$ (54 mg, 0.10 mmol) at room temperature. A dark red intermediate was observed upon addition of the reagents. The solution was allowed to stir for *ca.* 1 hour to give a bright orange solution. Crystallisation was achieved from a THF/toluene mixture stored at –30 °C for 18 hours. Yield 66 mg, 61 %.

Anal. Calcd. for $C_{68}H_{99}AlKN_5OSi_2$ (*1123.68*): C, 72.62; H, 8.88; N, 6.23 %. Found: C, 72.54; H, 8.57; N, 6.65 %.

^{1}H NMR (500 MHz, THF-D$_8$): δ 6.98 – 6.94 (m, 2H, C$_6$*H$_3$*), 6.92 – 6.88 (m, 2H, C$_6$*H$_3$*), 6.78 (d, *J* = 7.6, 1H, C$_6$*H$_3$*), 6.75 (d, *J* = 7.6, 1H, C$_6$*H$_3$*), 4.10 (sept, *J* = 6.8, 1H, C*H*Me$_2$), 4.02 – 3.92 (m, 2H, C*H*Me$_2$), 3.82 (sept, *J* = 6.8, 1H, C*H*Me$_2$), 3.62 (s, 4H, THF), 2.16 (br t, 3H, Ad), 2.08 – 1.94 (m, 10H, Ad), 1.85 – 1.71 (m, 18H, Ad, THF), 1.70 – 1.63 (m, 6H, Ad), 1.62 – 1.52 (m, 6H, Ad), 1.34 (d, *J* = 6.8, 3H, CH*Me$_2$*), 1.26 (d, *J* = 6.8, 3H, CH*Me$_2$*), 1.23 (d, *J* = 11.7, 5H, Ad), 1.19 (d, *J* = 6.8, 3H, CH*Me$_2$*), 1.17 (d, *J* = 6.8, 3H, CH*Me$_2$*), 1.15 (d, *J* = 6.8, 3H, CH*Me$_2$*), 1.16 – 1.12 (m, 3H, Ad), 1.10 (d, *J* = 6.8, 3H, CH*Me$_2$*), 1.08 (d, *J* = 6.8, 3H, CH*Me$_2$*), 0.99 (d, *J* = 6.8, 3H, CH*Me$_2$*), 0.78 – 0.71 (m, 3H, Ad), 0.30 (s, 3H, Si*Me$_2$*), 0.23 (s, 3H, Si*Me$_2$*), 0.20 (s, 3H, Si*Me$_2$*), –0.02 (s, 3H, Si*Me$_2$*).

^{13}C{^{1}H} NMR (126 MHz, THF-D$_8$): δ 226.6 (C=*C*=C), 212.6 (C–*C*=N), 148.7, 147.7, 147.4, 147.4, 145.8, 145.0 (*C$_6$*H$_3$), 129.7, 129.0, 124.2, 124.1, 123.9, 123.8, 122.1, 121.9 (*C$_6$*H$_3$), 108.5 (C–*C*=C), 68.3 (THF), 61.0, 58.3, 50.7, 45.2, 45.0, 42.3, 38.0, 37.5*, 31.2*, 30.5 (Ad), 29.9, 29.3, 29.2, 28.3 (CH*Me$_2$*), 27.9, 27.9, 27.5* (*C*HMe$_2$), 27.4 (CH*Me$_2$*), 26.4 (THF), 26.2, 26.0, 25.9 (CH*Me$_2$*), 7.2, 6.7, 5.9, 3.3 (Si*Me$_2$*).

*overlapping ^{13}C signal (appearing as a single signal).

Figure S23 ^{1}H NMR spectrum (500 MHz, THF-D$_8$) of [K(toluene)][Al(NON)(AdNC)$_3$] (**5·toluene**)

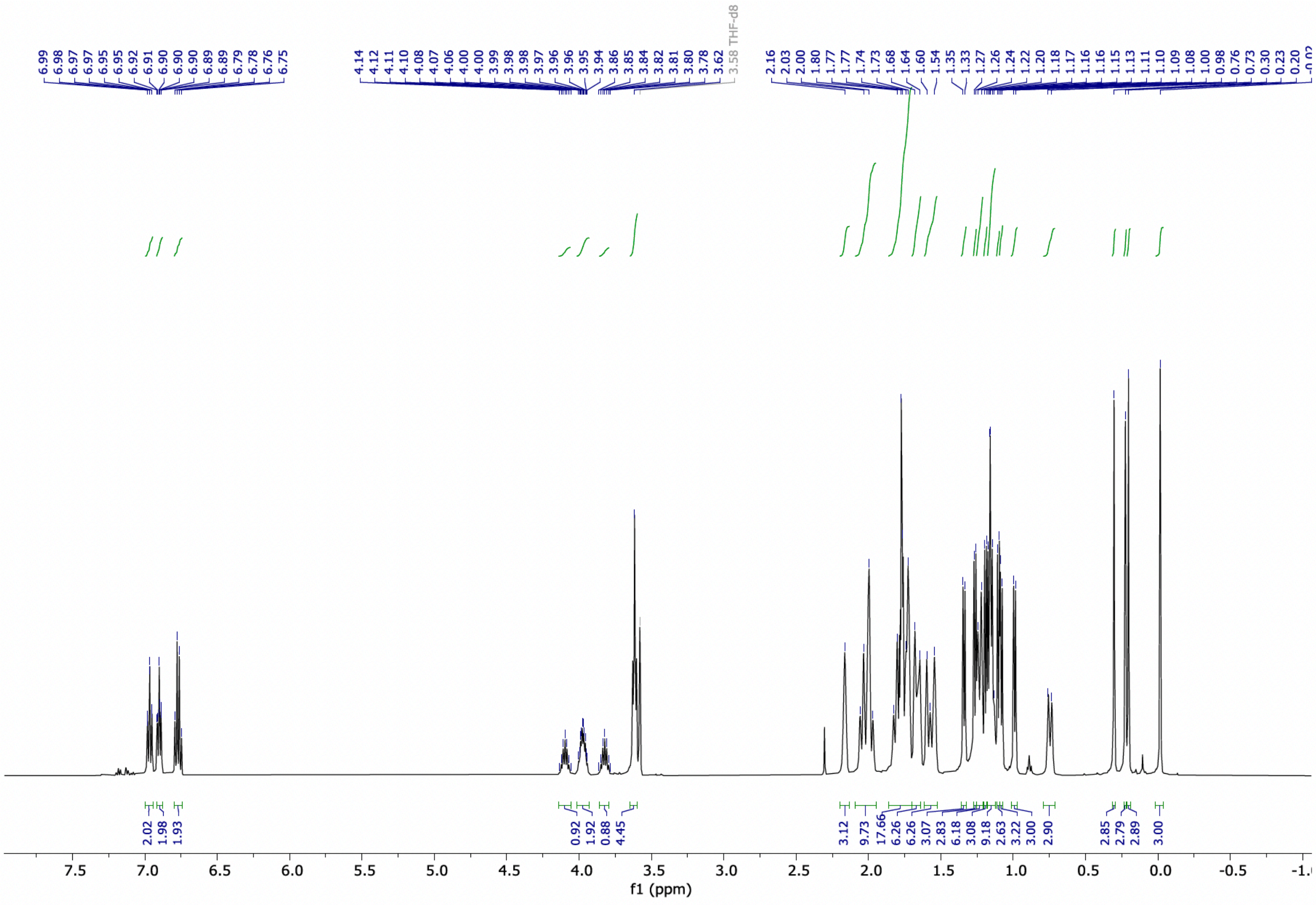

Figure S24 $^{13}C\{^{1}H\}$ NMR spectrum (126 MHz, THF-D_8) of [K(toluene)][Al(NON)(AdNC)$_3$] (**5·toluene**)

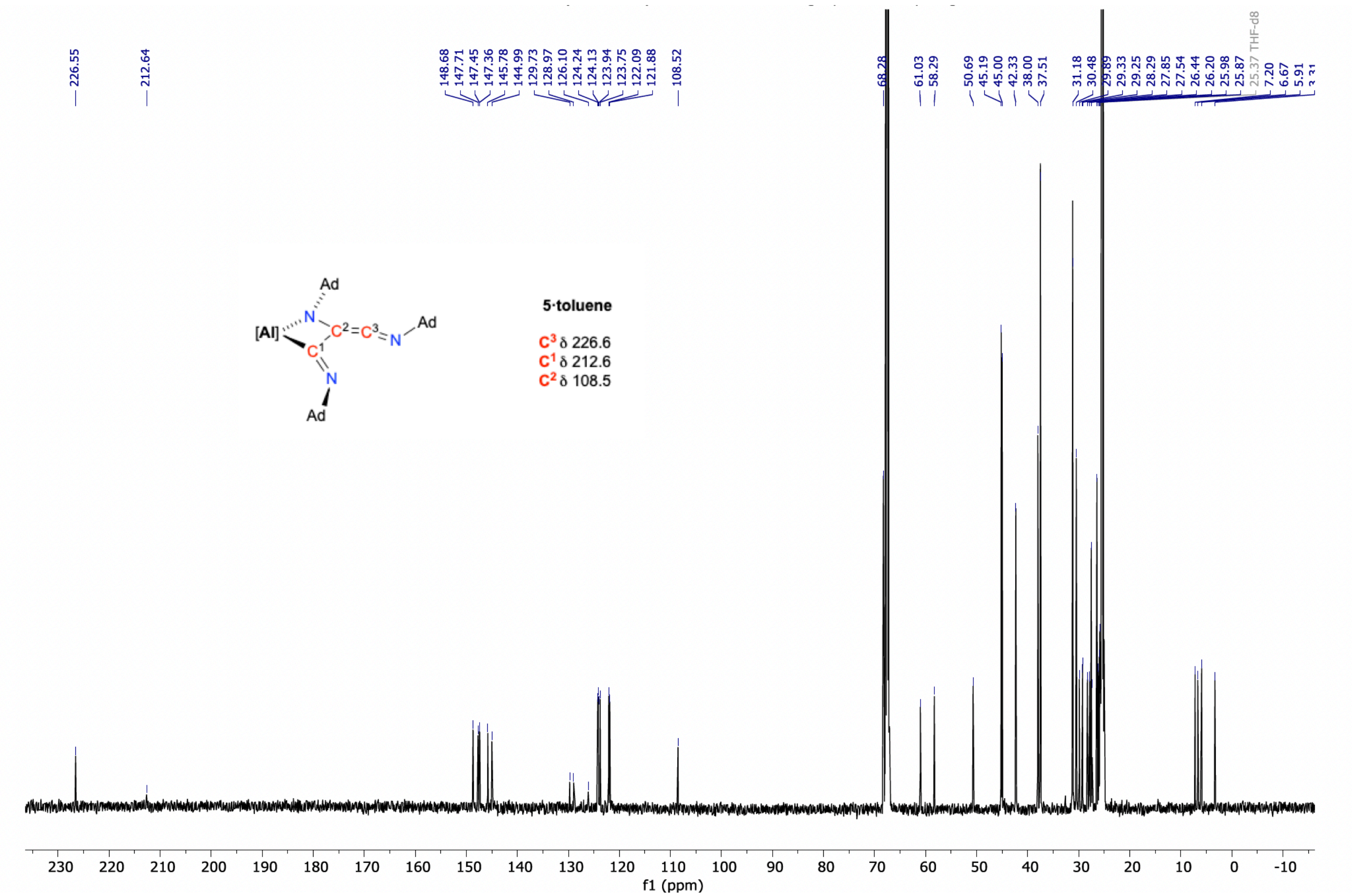

Figure S25 Displacement ellipsoid plot (30 %, H-atoms omitted) of [K(toluene)][Al(NON)(AdNC)$_3$] (**5·toluene**).

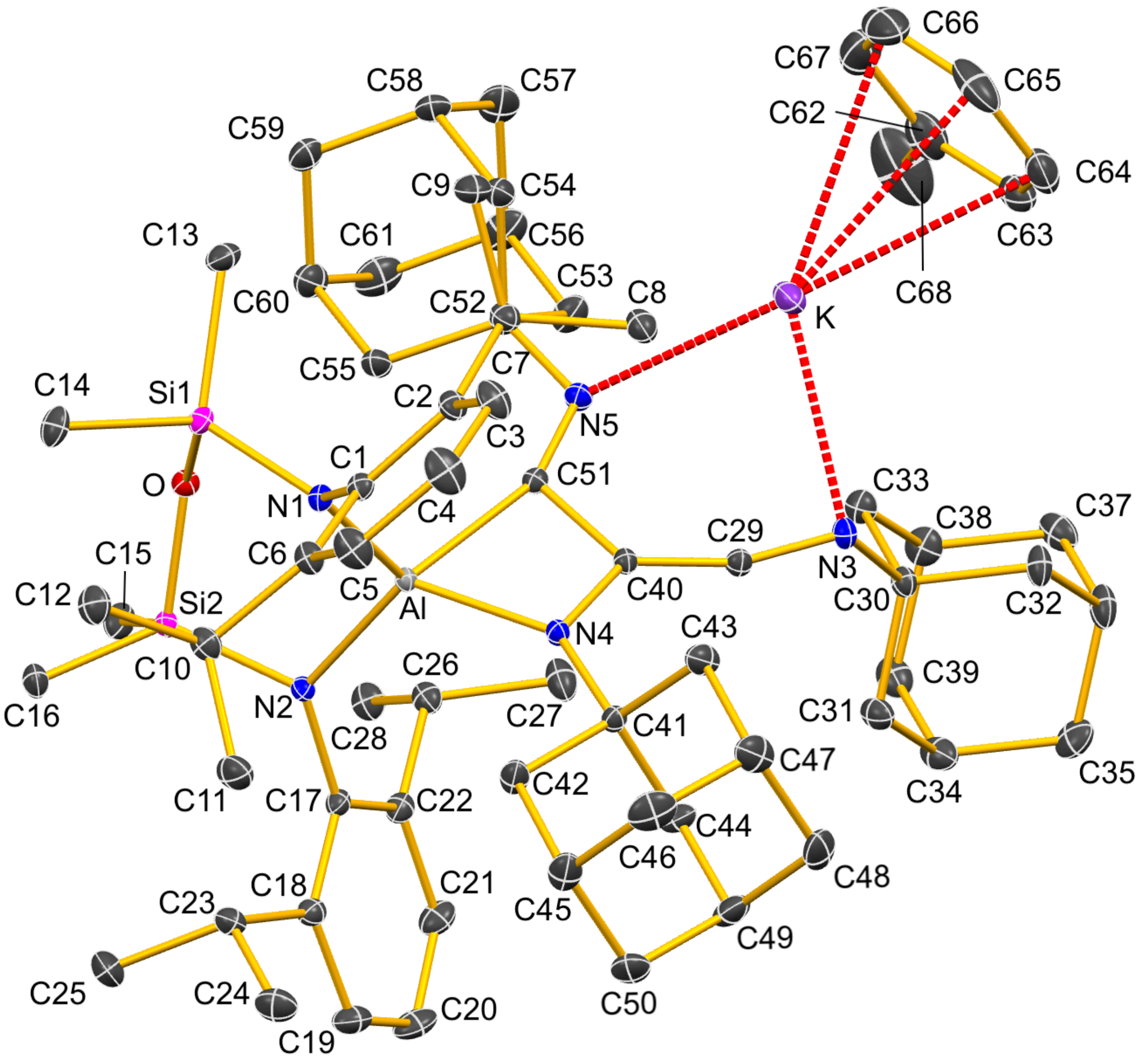

Crystallographic Details

Crystals were covered in inert oil and suitable single crystals were selected under a microscope and mounted on an Agilent SuperNova diffractometer fitted with an EOS S2 detector. Data were collected at 120 K (unless indicated otherwise) using focused microsource Cu Kα radiation at 1.54184 Å. Intensities were corrected for Lorentz and polarisation effects and for absorption using multi-scan methods.[2] Space groups were determined from systematic absences and checked for higher symmetry. All structures were solved using direct methods with SHELXS,[3] refined on F^2 using all data by full matrix least-squares procedures with SHELXL-97,[4] within the WinGX[5] program. Non-hydrogen atoms were refined with anisotropic displacement parameters. Hydrogen atoms were placed in calculated positions or manually assigned from residual electron density where appropriate, unless otherwise stated. The functions minimized were Σ*w*(*F2*o-*F2c*), with *w* = [σ2(F2o) + *a*P2 + *bP*]-1, where *P* = [max(Fo)2 + 2F2c]/3. The isotropic displacement parameters are 1.2 or 1.5 times the isotropic equivalent of their carrier atoms.

Additional Details:

*K[Al(NON)(H)(CN)] (**1a**)*: The unit cell contains a half of a poorly defined THF solvent molecule that lies on a 2-fold rotation access. A suitable model for this could not be developed and so it was treated as a diffuse contribution to the overall scattering without specific atom positions by SQUEEZE/PLATON. Details included in the .cif.

*[K(2.2.2)crypt][Al(NON)(H)(CN)] (**1-crypt**)*: The Al(H) unit is disordered over two positions located above and below the approximate plane of the ligand, with some additional disorder modelled in the iPr and Si-O-Si components of the ligand. Due to this disorder, the hydride ligands could not be located on the Al atoms. They were placed in calculated positions for a tetrahedral metal (HFIX 13) and the Al–H distance contained to be 1.6 A (in accordance with related structures).

K[Al(NON){(DmpNC)$_2$(DmpNC)]·2(C_7H_8) (**2·toluene**)*: A residual electron density peak from the diffraction located between 3 × alkyl groups was assigned to a low occupancy position for the potassium cation. The disordered toluene (about an inversion centre) forms a π-interaction with this low occupancy K^+, and is located between the 1-D chains that are formed when the major occupancy K^+ interacts with the oxygen atom of the ligand scaffold in an adjacent molecule.

*[K(THF)$_2$][Al(NON)(AdNC)$_2$(DmpNC)] (**4·THF**)*: There is significant disorder in the molecule due to the presence of either THF / Et2O at one of the coordination sites of potassium. This was modelled as THF (67.4%) and Et2O (30.6%), leading to the non-integer value for the number of hydrogen atoms in the asymmetric unit. This substitution caused additional disorder in the molecule which was modelled for the major components but ignored for the minor components (leading to the Alert Level B concerning the large Ueq(max)/Ueq(min) or carbon).

Table S1 Crystal structure and refinement data for K[Al(NON)(H)(CN)] (**1a**), [K(2.2.2)crypt][Al(NON)(H)(CN)] (**1-crypt**), [K(Et_2O)$_2$][Al(NON){(DmpNC)$_2$(DmpNC*)] (**2·Et$_2$O**, * = ring activated DmpNC) and K[Al(NON){(DmpNC)$_2$(DmpNC*)]·2(C_7H_8) (**2·toluene**, * = ring activated DmpNC).

	**1a**	**1·crypt**	**2·Et$_2$O**	**2·toluene**
**Empirical formula**	$C_{31}H_{51}AlKN_3O_{1.5}Si_2$	$C_{47}H_{83}AlKN_5O_7Si_2$	$C_{63}H_{93}AlKN_5O_3Si_2$	$C_{131}H_{170}Al_2K_2N_{10}O_2Si_4$
**CCDC Number**	2247345	2247346	2247347	2247348
**M_r**	612.00	952.44	1090.68	2161.28
***T* [K]**	150.0(1)	120.1(2)	120.01(10)	120.01(10)
**Crystal size [mm]**	0.34 × 0.26 × 0.22	0.25 × 0.21 × 0.12	0.24 × 0.14 × 0.09	0.20 × 0.09 × 0.08
**Crystal system**	Monoclinic	Triclinic	Monoclinic	Triclinic
**Space group**	I2/*a* (alternative No.15)	$P\overline{1}$ (No.2)	$P2_1/c$ (No.14)	$P\overline{1}$ (No.2)
***a* [Å]**	18.16775(17)	12.3130(3)	11.59172(14)	11.0552(3)
***b* [Å]**	13.05007(9)	12.5109(3)	22.52352(17)	11.8345(3)
***c* [Å]**	30.7237(2)	19.2634(5)	24.5925(2)	23.9671(7)
**α [°]**	90	104.783(2)	90	82.310(2)
**β [°]**	102.7091(8)	105.037(2)	96.0048(9)	76.873(2)
**γ [°]**	90	99.317(2)	90	80.547(2)
**V [Å^3]**	7105.83(9)	2687.05(13)	6385.53(11)	2997.04
Z	8	2	4	1
**$D_{calc.}$ [mg m^{-3}]**	1.144	1.177	1.135	1.198
**Absorption coefficient [mm^{-1}]**	2.41	1.85	1.57	1.65
**θ range for data collection [°]**	3.694 to 73.296	3.768 to 73.236	3.614 to 73.313	3.806 to 73.303
**Reflections collected**	48752	30839	45551	39077
**Independent reflections**	7138 [R_{int} 0.029]	10624 [R_{int} 0.023]	12745 [R_{int} 0.029]	11958 [R_{int} 0.048]
**Reflections with $I > 2\sigma(I)$**	6775	10030	11417	10186
**Data/restraints/parameters**	7138 / 0 / 351	10624 / 2 / 682	12745 / 0 / 698	11958 / 0 / 725
**Final *R* indices [$I > 2\sigma(I)$]**	R_1 = 0.038, wR_2 = 0.111	R_1 = 0.081, wR_2 = 0.187	R_1 = 0.035, wR_2 = 0.089	R_1 = 0.090 wR_2 = 0.227
**Final *R* indices (all data)**	R_1 = 0.040, wR_2 = 0.112	R_1 = 0.084, wR_2 = 0.189	R_1 = 0.040, wR_2 = 0.092	R_1 = 0.102, wR_2 = 0.234
**GOOF on F^2**	1.027	1.12	1.003	1.101
**Largest diff. peak/hole [e.Å^{-3}]**	0.86 and –0.45	1.00 and –0.81	0.30 and –0.29	0.979 and –0.669

Table S2 Crystal structure and refinement data for $K(Et_2O)][Al(NON)(AdNC)_2]$ (**3·Et₂O**), $[K(THF)_2][Al(NON)(AdNC)_2(DmpNC)]$ (**4·THF**) and $[K(toluene)][Al(NON)(AdNC)_3]$ (**5·toluene**).

	**3·Et$_2$O**	**4·THF**	**5·toluene**
**Empirical formula**	$C_{54}H_{86}AlKN_4O_2Si_2$	$C_{67}H_{101.61}AlKN_5O_3Si_2$	$C_{68}H_{99}AlKN_5OSi_2$
**CCDC Number**	2247349	2247350	2247351
**M_r**	945.52	1147.4	1124.78
**T [K]**	125(7)	150.00(10)	150.00(10)
**Crystal size [mm]**	0.24 × 0.15 × 0.10	0.15 × 0.14 × 0.09	0.24 × 0.09 × 0.06
**Crystal system**	Orthorhombic	Triclinic	Monoclinic
**Space group**	Pbca (No. 61)	$P\overline{1}$ (No.2)	C*c* (No. 9)
**a [Å]**	22.07209(13)	12.7748(6)	36.4496(7)
**b [Å]**	19.95370(13)	14.6769(7)	12.44536(9)
**c [Å]**	24.45220(16)	19.1675(8)	20.2703(4)
**α [°]**	90	84.046(4)	90
**β [°]**	90	88.135(4)	136.513(3)
**γ [°]**	90	65.381(5)	90
**V [Å^3]**	10769.23(12)	3249.3(3)	6328.05(18)
**Z**	8	2	4
**$D_{calc.}$ [mg m^{-3}]**	1.167	1.173	1.181
**Absorption coefficient [mm^{-1}]**	1.176	1.1564	1.575
**θ range for data collection [°]**	3.490 to 73.461	3.806 to 73.238	3.524 to 73.290
**Reflections collected**	137184	43228	20829
**Independent reflections**	10812 [R_{int} 0.063]	12963 [R_{int} 0.042]	8059
**Reflections with $I > 2\sigma(I)$**	10149	10297	7878
**Data/restraints/parameters**	10812 / 0 / 591	12963 / 0 / 864	8059 / 2 / 716
**Final R indices [$I > 2\sigma(I)$]**	$R_1 = 0.035$, w$R_2 = 0.093$	$R_1 = 0.058$, w$R_2 = 0.143$	$R_1 = 0.024$, w$R_2 = 0.060$
**Final R indices (all data)**	$R_1 = 0.037$, w$R_2 = 0.095$	$R_1 = 0.075$, w$R_2 = 0.153$	$R_1 = 0.025$, w$R_2 = 0.061$
**GOOF on F^2**	1.033	1.032	1.011
**Largest diff. peak/hole [e.Å^{-3}]**	0.30 and –0.47	0.57 and –0.67	0.20 and –0.21

Computational Methodology

DFT calculations were run with Gaussian 16 (C.01).[6] The Al, Si and K centres were described with the Stuttgart RECPs and associated basis sets,[7] and the 6-31G** basis set was used for all other atoms (BS1).[8] A polarization function was also added to Al (ζ_d = 0.190), Si (ζ_d = 0.284) and K (ζ_d = 1.000). Initial BP86 optimizations were performed using the 'grid = ultrafine' option,[9] with all stationary points being fully characterized via analytical frequency calculations as minima or transition states (all positive eigenvalues or one imaginary eigenvalue respectively), and with intrinsic reaction coordinate calculations confirming the connectivity of the reaction pathways. All energies were recomputed with a larger basis set featuring 6-311++G** basis sets on all atoms (BS2). Corrections for the effect of toluene (ε = 2.3741) solvent were run using the polarizable continuum model and BS1,[10] using the keyword "scrf=toluene" within Gaussian. Single-point dispersion corrections to the BP86 results employed Grimme's D3 parameter set with Becke-Johnson damping as implemented in Gaussian.[11] Natural Bonding Orbital (NBO7)[12] analyses were performed on the BP86-optmised geometries at the BP86/6-311++G** level, within Gaussian 16 (C.01).

Figure S26 Representation of the HOMO of [K(toluene)][Al(NON)(AdNC)$_3$] (**5·toluene**)

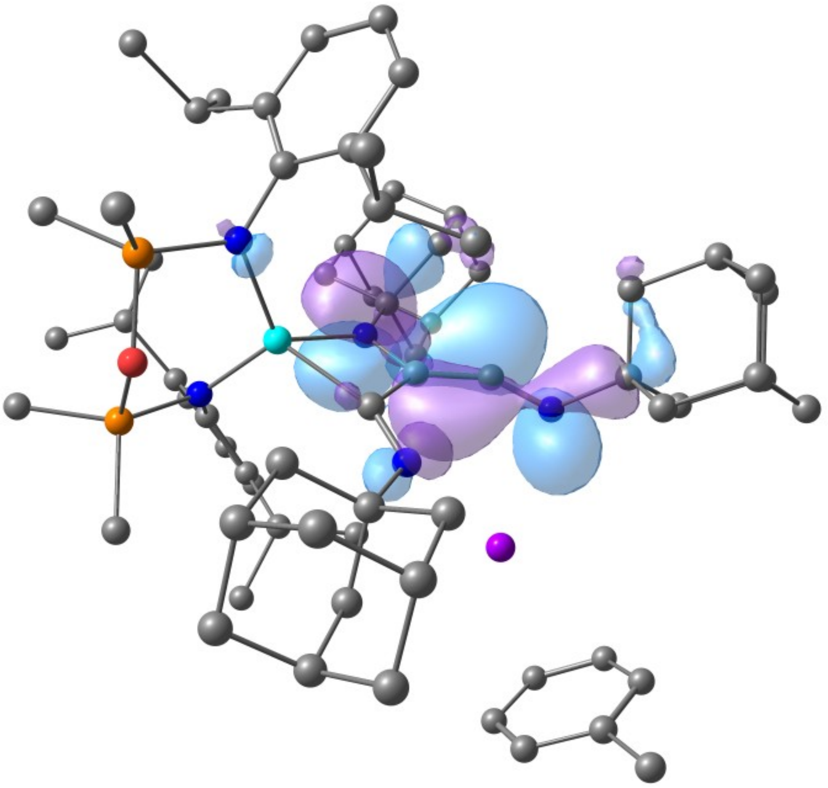

Breakdown of Energy Contributions

The following tables detail the evolution of the relative energies as the successive corrections to the initial SCF energy are included. Terms used are:

$\mathbf{\Delta E_{BSI}}$	SCF energy computed with the BP86 functional with BS1
$\mathbf{\Delta H_{BSI}}$	Enthalpy at 0 K with BS1
$\mathbf{\Delta G_{BSI}}$	Free energy at 298.15 K and 1 atm with BS1
$\mathbf{\Delta G_{BSI/\mathit{tol}}}$	Free energy corrected for toluene solvent with BS1
$\mathbf{\Delta G_{BSI/\mathit{tol}+D3BJ}}$	Free energy corrected for toluene and dispersion effects with BS1
$\mathbf{\Delta E_{BS2}}$	SCF energy computed with the BP86 functional with BS2
$\mathbf{\Delta G_{\mathit{tol}}}$	Free energy corrected for basis set (BS2), dispersion effects and toluene solvent

In each case the final data used in the main article are highlighted in bold. Free energies are quoted in kcal mol^{-1}, and include all three single point corrections (for BS2, solvation and dispersion).

Table S3. Relative energies for computed structures(kcal mol^{-1}). Data in bold is at the same level as free energies used in the text. Free energies are quoted relative to **A**.

	ΔE_{BSI}	ΔH_{BSI}	ΔG_{BSI}	$\Delta G_{BSI/tol}$	$\Delta G_{BSI/tol+D3BJ}$	ΔE_{BS2}	ΔG_{tol}
**A**	0.0	0.0	0.0	0.0	0.0	0.0	**0.0**
**B**	-1.2	-0.4	7.3	7.9	-1.5	1.4	**1.1**
**B′**	-2.4	-2.1	3.2	3.6	-1.5	-0.2	**0.7**
**C**	-2.1	-1.2	11.0	11.9	1.0	1.4	**4.5**
**TS(C-D)**	-2.3	-1.3	11.6	12.5	-0.7	2.2	**3.8**
**D**	-2.6	-1.6	10.7	11.6	-2.8	2.2	**1.9**
**E**	-4.5	-3.3	14.8	15.4	1.0	2.3	**7.8**
**TS(E-F)**	3.5	4.5	23.7	24.6	6.4	9.4	**12.3**
**F**	-5.7	-4.1	15.8	17.4	-7.5	-0.3	**-2.1**
**G**	3.7	5.1	18.0	16.1	8.0	6.6	**10.9**
**TS(G-H)**	10.4	11.4	31.9	32.3	13.8	12.1	**15.5**
**H**	-48.0	-44.7	-22.2	-21.3	-45.1	-45.1	**-42.2**
**G·Et$_2$O**	0.2	1.7	19.0	17.9	4.0	4.0	**7.9**
**TS(G·Et$_2$O-3·Et$_2$O)**	1.0	2.7	33.9	36.3	5.1	6.1	**10.2**
**3·Et$_2$O**	-58.0	-54.4	-21.8	-18.9	-52.0	-52.7	**-46.6**
**I**	-45.3	-40.7	-2.3	0.0	-44.8	-37.9	**-37.3**
**J**	-55.7	-52.0	-18.9	-16.0	-46.5	-49.1	**-40.0**
**TS(I-K)**	-38.7	-35.3	3.9	5.0	-42.8	-31.0	**-34.1**
**TS(J-K)**	-16.0	-11.7	26.8	29.9	-14.3	-7.4	**-5.7**
**K**	-65.8	-59.8	-21.3	-21.7	-70.0	-56.8	**-60.9**
**4·THF**	-86.5	-79.1	-18.5	-14.1	-81.1	-72.3	**-66.8**
**L**	-38.4	-34.5	-3.1	-2.3	-41.9	-30.3	**-33.8**
**TS(L-M)**	-24.5	-20.1	-17.9	19.3	-27.5	-14.9	**-18.0**
**M**	-63.6	-57.6	-18.9	-18.2	-69.6	-55.9	**-59.9**
**I′**	-45.6	-41.1	-4.7	-2.5	-44.0	-37.9	**-36.2**
**J′**	-60.2	-56.4	-25.0	-21.6	-48.5	-53.0	**-41.3**
**TS(I′-K′)**	-39.9	-35.6	2.0	2.8	-47.5	-31.9	**-39.4**
**TS(J′-K′)**	-19.3	-15.7	21.1	24.3	-19.5	-9.9	**-10.6**
**K′**	-66.2	-60.4	-22.3	-21.8	-71.3	-56.5	**-61.6**
**5·toluene**	-73.3	-66.9	-19.4	-16.2	-74.0	-61.5	**-62.3**
**L′**	-46.9	-42.6	-10.3	-8.6	-43.2	-36.9	**-33.3**
**TS(L′-K′)**	-22.4	-18.2	18.7	19.9	-23.0	-13.4	**-14.0**

Figure S27 DFT computed free energy profile (BP86-D3BJ,(PCM=toluene)/BS2//BP86/BS1, in kcal mol^{-1}) for splitting the [K{Al(NON)}]$_2$ (**A**) dimer and coordination of Ad-NC.

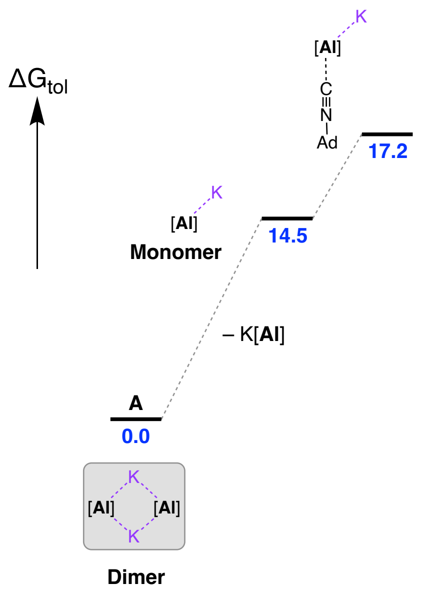

Figure S28 DFT computed free energy profile (BP86-D3BJ,(PCM=toluene)/BS2//BP86/BS1, in kcal mol^{-1}) for the coordination of three equivalents of Ad-NC to [K{Al(NON)}]$_2$ (**A**)

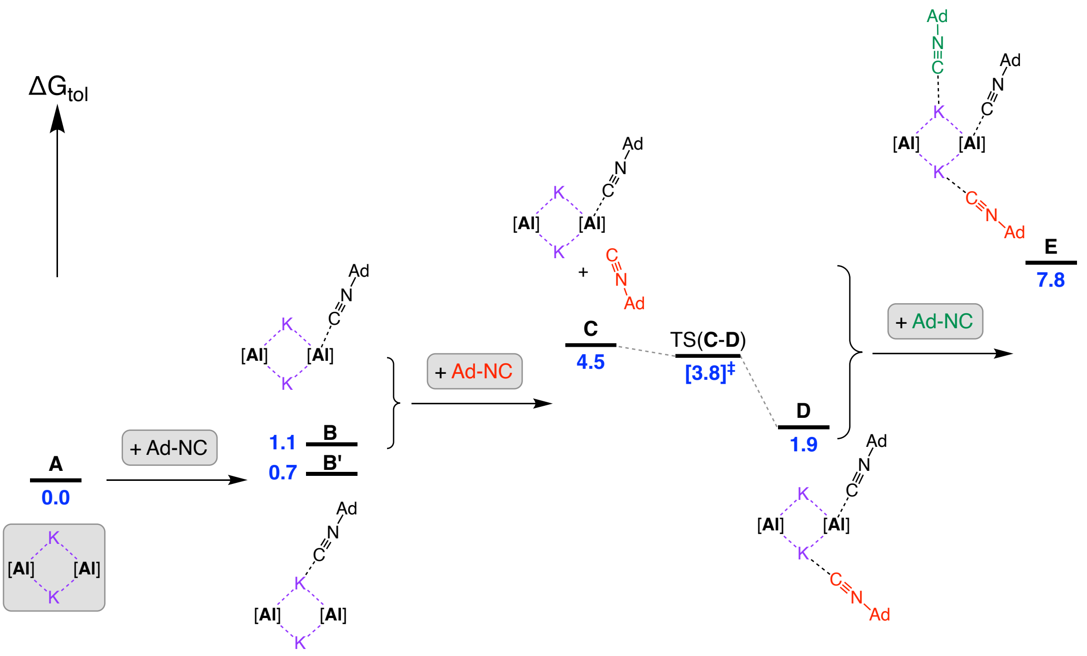

Figure S29 DFT computed free energy profile (BP86-D3BJ,(PCM=toluene)/BS2//BP86/BS1, in kcal mol^{-1}) for the Et_2O assisted coupling of two Ad-NC molecules.

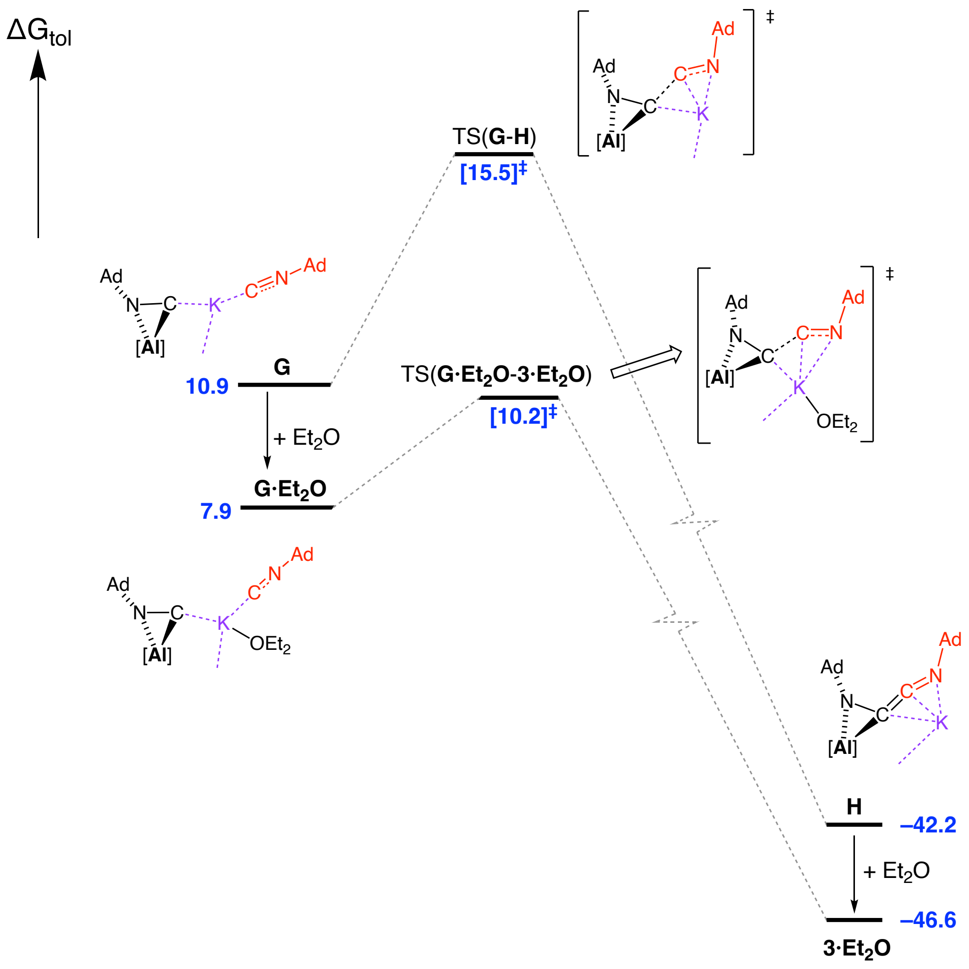

Figure S30 DFT computed free energy profile (BP86-D3BJ,(PCM=toluene)/BS2//BP86/BS1, in kcal mol^{-1}) for the formation of the hypothetical {$Ad_{am}Dmp_{ket}Ad_{im}$} isomer of K[Al(NON)(AdNC)$_2$(DmpNC)].

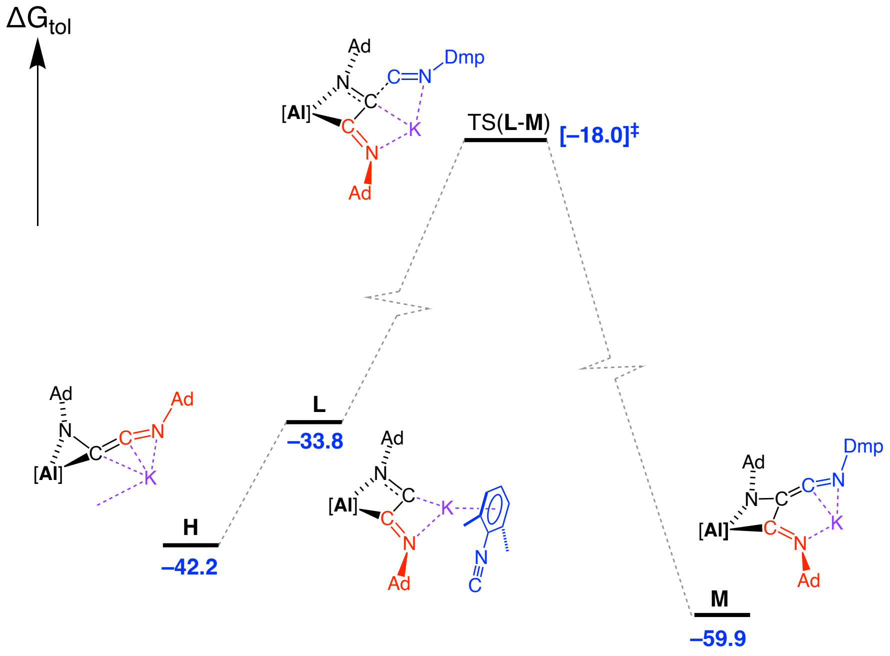

Figure S31 DFT computed free energy profile (BP86-D3BJ,(PCM=toluene)/BS2//BP86/BS1, in kcal mol^{-1}) for the formation of [K(toluene)][Al(NON)(AdNC)$_3$] (**5·toluene**).

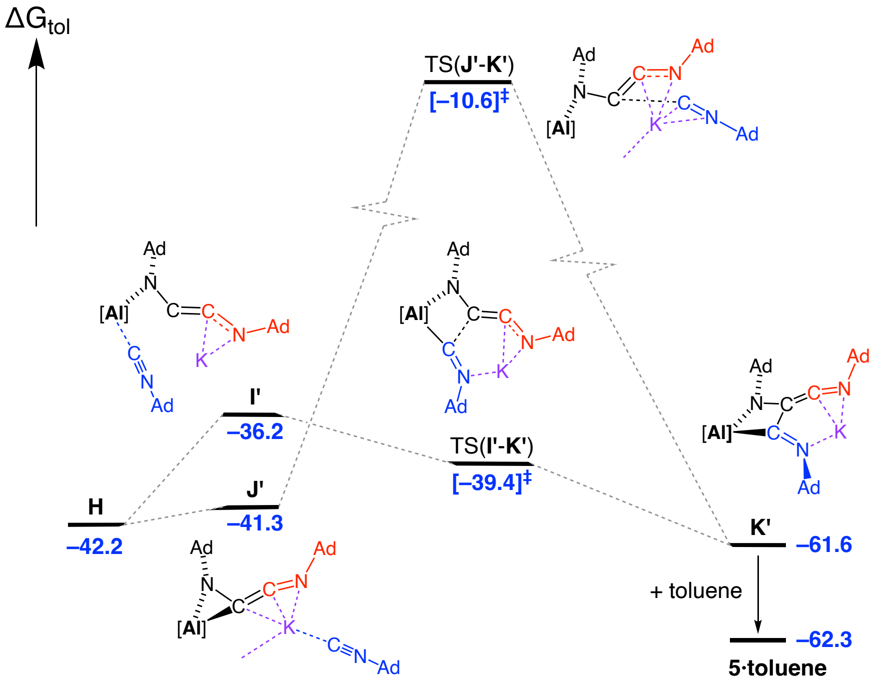

Figure S32 DFT computed free energy profile (BP86-D3BJ,(PCM=toluene)/BS2//BP86/BS1, in kcal mol^{-1}) for the formation of K[Al(NON)(AdNC)$_3$] via the third pathway.

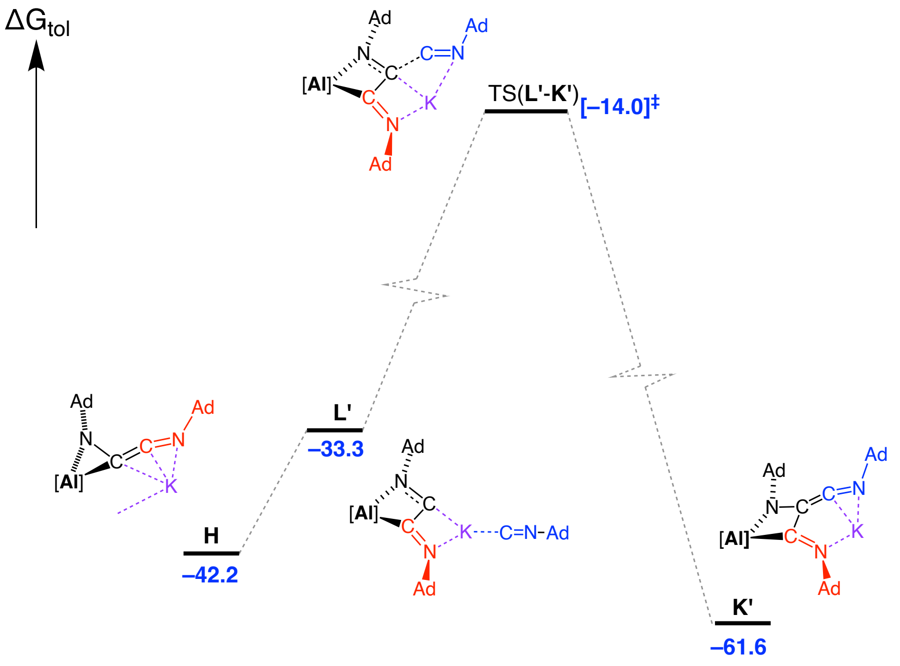

References

[1] R. J. Schwamm, M. D. Anker, M. Lein, M. P. Coles, *Angew. Chem. Int. Ed.* **2019**, *58*, 1489-1493.

[2] R. Blessing, *Acta Cryst.* **1995**, *A51*, 33-38.

[3] G. M. Sheldrick, *Acta Cryst.* **2008**, *A64*, 112-122.

[4] G. M. Sheldrick, University of Gottingen, Germany, **1997**.

[5] L. J. Farrugia, *J. Appl. Cryst.* **1999**, *32*, 837-838.

[6] M. J. Frisch, G. W. Trucks, H. B. Schlegel, G. E. Scuseria, M. A. Robb, J. R. Cheeseman, G. Scalmani, V. Barone, G. A. Petersson, H. Nakatsuji, X. Li, M. Caricato, A. V. Marenich, J. Bloino, B. G. Janesko, R. Gomperts, B. Mennucci, H. P. Hratchian, J. V. Ortiz, A. F. Izmaylov, J. L. Sonnenberg, Williams, F. Ding, F. Lipparini, F. Egidi, J. Goings, B. Peng, A. Petrone, T. Henderson, D. Ranasinghe, V. G. Zakrzewski, J. Gao, N. Rega, G. Zheng, W. Liang, M. Hada, M. Ehara, K. Toyota, R. Fukuda, J. Hasegawa, M. Ishida, T. Nakajima, Y. Honda, O. Kitao, H. Nakai, T. Vreven, K. Throssell, J. A. Montgomery Jr., J. E. Peralta, F. Ogliaro, M. J. Bearpark, J. J. Heyd, E. N. Brothers, K. N. Kudin, V. N. Staroverov, T. A. Keith, R. Kobayashi, J. Normand, K. Raghavachari, A. P. Rendell, J. C. Burant, S. S. Iyengar, J. Tomasi, M. Cossi, J. M. Millam, M. Klene, C. Adamo, R. Cammi, J. W. Ochterski, R. L. Martin, K. Morokuma, O. Farkas, J. B. Foresman, D. J. Fox, Wallingford, CT, **2016**.

[7] D. Andrae, U. Häußermann, M. Dolg, H. Stoll, H. Preuß, *Theor. Chim. Acta* **1990**, *77*, 123-141.

[8] (a) P. C. Hariharan, J. A. Pople, *Theor. Chim. Acta* **1973**, *28*, 213-222; (b) W. J. Hehre, R. Ditchfield, J. A. Pople, *J. Chem. Phys.* **1972**, *56*, 2257-2261.

[9] (a) A. D. Becke, *Phys. Rev. A* **1988**, *38*, 3098-3100; (b) J. P. Perdew, *Phys. Rev. B* **1986**, *33*, 8822-8824.

[10] J. Tomasi, B. Mennucci, R. Cammi, *Chem. Rev.* **2005**, *105*, 2999-3094.

[11] S. Grimme, S. Ehrlich, L. Goerigk, *J. Comp. Chem.* **2011**, *32*, 1456-1465.

[12] E. D. Glendening, J. Badenhoop, K., A. E. Reed, J. E. Carpenter, J. A. Bohmann, C. M. Morales, P. Karafiloglou, C. R. Landis, F. Weinhold, Theoretical Chemistry Institute, University of Wisconsin, Madison, USA, **2003**.

Cartesian Coordinates and Computed Energies [in Hartrees] for Calculated Structures

AdNC
SCF (BP86) Energy = -482.928833186
Enthalpy 0K = -482.694447
Enthalpy 298K = -482.684176
Free Energy 298K = -482.727013
Lowest Frequency = 132.0188 cm^{-1}
Second Frequency = 132.0192 cm^{-1}
SCF (BP86-D3BJ) Energy = -482.983068007
SCF (tol) Energy = -482.931714038
SCF (BS2) Energy = -483.043305574

```
N   0.00000  0.00000  2.49479
C  -0.00000  0.00000  3.68647
C   0.00000  0.00000  1.05755
C  -0.00000  1.46410  0.53700
H  -0.89040  1.98845  0.92948
H   0.89040  1.98845  0.92948
C   1.26795 -0.73205  0.53700
H   1.27685 -1.76533  0.92948
H   2.16725 -0.22312  0.92948
C  -1.26795 -0.73205  0.53700
H  -2.16725 -0.22312  0.92948
H  -1.27685 -1.76533  0.92948
C  -0.00000  1.45998 -1.01050
H  -0.00000  2.50569 -1.37038
C   1.26425  0.72992 -1.52446
H   1.28484  0.74180 -2.63063
H   2.17491  1.25568 -1.17939
C   1.26438 -0.72999 -1.01050
H   2.16999 -1.25284 -1.37038
C  -0.00000 -1.45983 -1.52446
H  -0.00000 -1.48360 -2.63063
H  -0.00000 -2.51137 -1.17939
C  -1.26438 -0.72999 -1.01050
H  -2.16999 -1.25284 -1.37038
C  -1.26425  0.72992 -1.52446
H  -1.28484  0.74180 -2.63063
H  -2.17491  1.25568 -1.17939
```

Ar’NC
SCF (BP86) Energy = -403.086260309
Enthalpy 0K = -402.936922
Enthalpy 298K = -402.926064
Free Energy 298K = -402.971842
Lowest Frequency = 104.0377 cm^{-1}
Second Frequency = 104.4888 cm^{-1}
SCF (BP86-D3BJ) Energy = -403.119812143
SCF (tol) Energy = -403.088797622
SCF (BS2) Energy = -403.186415552

```
N   1.82997 -0.00196  0.00004
C   3.02552 -0.00310 -0.00009
C   0.44090 -0.00048  0.00002
C  -0.23753  1.24578 -0.00005
C  -0.24018 -1.24530  0.00006
C   0.53634  2.54192  0.00004
H  -0.14541  3.40639  0.00088
H   1.19257  2.61861 -0.88474
H   1.19371  2.61783  0.88406
C   0.53093 -2.54308  0.00000
H   1.18816 -2.62039 -0.88400
H  -0.15266 -3.40610 -0.00086
H   1.18696 -2.62116  0.88481
C  -1.64281  1.21799 -0.00003
H  -2.19021  2.16677 -0.00005
C  -2.34285  0.00248 -0.00002
H  -3.43764  0.00365 -0.00004
C  -1.64540 -1.21452  0.00000
H  -2.19482 -2.16212  0.00001
```

Et_2O
SCF (BP86) Energy = -233.654981611
Enthalpy 0K = -233.521943
Enthalpy 298K = -233.514142
Free Energy 298K = -233.552232
Lowest Frequency = 64.8588 cm^{-1}
Second Frequency = 127.4962 cm^{-1}
SCF (BP86-D3BJ) Energy = -233.669091718
SCF (tol) Energy = -233.656181129
SCF (BS2) Energy = -233.719121473

```
O   0.00000 -0.00099  0.84706
C  -1.00272 -0.66248  0.06724
H  -1.41089 -1.45617  0.71844
H  -0.55431 -1.16692 -0.81492
C  -2.12608  0.28358 -0.37343
H  -1.74876  1.08285 -1.03404
H  -2.59416  0.75819  0.50446
H  -2.90411 -0.26972 -0.92834
C   1.00248  0.66219  0.06851
H   1.41052  1.45504  0.72087
H   0.55411  1.16820 -0.81280
C   2.12627 -0.28268 -0.37391
H   1.74946 -1.08078 -1.03627
H   2.59450 -0.75876  0.50310
H   2.90399  0.27234 -0.92749
```

THF
SCF (BP86) Energy = -232.447574761
Enthalpy 0K = -232.333967
Enthalpy 298K = -232.327978
Free Energy 298K = -232.363078
Lowest Frequency = 23.1459 cm^{-1}
Second Frequency = 275.4150 cm^{-1}
SCF (BP86-D3BJ) Energy = -232.461052284
SCF (tol) Energy = -232.448939410
SCF (BS2) Energy = -232.511411715

```
C   1.11953  0.49016  0.18507
O  -0.01624  1.21238 -0.30202
C  -1.14796  0.45256  0.13889
C  -0.75507 -1.04216 -0.02233
C   0.80213 -0.99935 -0.08328
H   2.01008  0.86631 -0.34439
H   1.25822  0.66776  1.27604
H  -1.37417  0.67882  1.20507
H  -2.01389  0.75889 -0.47051
H  -1.12708 -1.64979  0.81904
H  -1.17361 -1.46775 -0.94858
H   1.27911 -1.66696  0.65304
H   1.15943 -1.29360 -1.08365
```

Toluene (C_7H_8)
SCF (BP86) Energy = -271.559258300
Enthalpy 0K = -271.434922
Enthalpy 298K = -271.427572
Free Energy 298K = -271.465923
Lowest Frequency = 28.3294 cm^{-1}
Second Frequency = 202.7397 cm^{-1}
SCF (BP86-D3BJ) Energy = -271.581062958
SCF (tol) Energy = -271.560402015
SCF (BS2) Energy = -271.625271775

```
C  -0.92053  0.00004 -0.01165
C  -0.19617  1.20974 -0.00911
H  -0.74140  2.16116 -0.01831
C   1.20734  1.21260  0.00222
H   1.74901  2.16477  0.00117
C   1.91519 -0.00003  0.00875
H   3.01024 -0.00006  0.01406
C   1.20727 -1.21263  0.00222
H   1.74891 -2.16482  0.00117
C  -0.19622 -1.20972 -0.00911
H  -0.74149 -2.16111 -0.01832
C  -2.43510  0.00002  0.00929
H  -2.84731  0.89355 -0.48855
H  -2.82133 -0.00239  1.04566
H  -2.84735 -0.89122 -0.49263
```

A
SCF (BP86) Energy = -2635.74430418
Enthalpy 0K = -2634.413171
Enthalpy 298K = -2634.318262
Free Energy 298K = -2634.553383
Lowest Frequency = 8.2172 cm-1
Second Frequency = 11.6603 cm-1
SCF (BP86-D3BJ) Energy = -2636.19792467
SCF (tol) Energy = -2635.75267860
SCF (BS2) Energy = -5403.03948350

```
K   0.00010  2.32736  0.00027
Si  5.76066  1.17016 -1.04125
Si  5.76046 -1.17060  1.04091
Al  2.86534 -0.00002 -0.00016
O   6.40873 -0.00047 -0.00039
N   4.05657  1.40607 -0.60774
N   4.05630 -1.40635  0.60757
C   3.38831  2.65502 -0.75757
C   3.28766  3.55616  0.35895
C   2.50960  4.72646  0.24588
H   2.44198  5.40686  1.10299
C   1.83779  5.05128 -0.94244
H   1.25263  5.97413 -1.01679
C   1.95073  4.18821 -2.04192
H   1.44694  4.44659 -2.98142
C   2.71199  3.00127 -1.97778
C   4.00323  3.26550  1.67893
H   4.68539  2.42033  1.48387
C   4.84437  4.46174  2.17399
H   5.55075  4.80556  1.40026
H   5.42643  4.17583  3.06742
H   4.21313  5.32287  2.45790
C   3.00906  2.80971  2.77130
H   2.26120  3.59662  2.98293
H   3.53544  2.58589  3.71629
H   2.46910  1.89519  2.46049
C   2.80467  2.12447 -3.22670
H   3.43430  1.26010 -2.95996
C   3.48454  2.87769 -4.39284
H   2.87802  3.73814 -4.72831
H   3.62069  2.20881 -5.26081
H   4.47464  3.26597 -4.09989
C   1.42875  1.57458 -3.66331
H   0.98017  0.94976 -2.86952
H   1.53031  0.94446 -4.56445
H   0.72103  2.38789 -3.90791
C   6.02957  0.48655 -2.79449
H   7.08831  0.19910 -2.91675
H   5.78759  1.22691 -3.57517
H   5.41314 -0.41138 -2.97006
C   6.76706  2.76462 -0.84592
H   6.81335  3.08400  0.20759
H   6.32838  3.58726 -1.43613
H   7.80013  2.60534 -1.19916
C   6.02967 -0.48662  2.79395
H   7.08841 -0.19904  2.91587
H   5.78798 -1.22689  3.57480
H   5.41320  0.41127  2.96957
C   6.76650 -2.76534  0.84594
H   6.81305 -3.08471 -0.20756
H   6.32736 -3.58787  1.43597
H   7.79949 -2.60637  1.19954
C   3.38790 -2.65517  0.75765
C   2.71172 -3.00120  1.97801
C   1.95037 -4.18806  2.04242
H   1.44670 -4.44626  2.98204
C   1.83717 -5.05126  0.94307
H   1.25194 -5.97405  1.01763
C   2.50882 -4.72665 -0.24539
H   2.44101 -5.40716 -1.10240
C   3.28699 -3.55645 -0.35873
C   2.80460 -2.12418  3.22677
H   3.43439 -1.25998  2.95985
C   3.48432 -2.87727  4.39307
H   2.87765 -3.73753  4.72874
H   3.62060 -2.20822  5.26089
H   4.47436 -3.26580  4.10020
C   1.42877 -1.57395  3.66325
H   0.98032 -0.94919  2.86934
H   1.53043 -0.94369  4.56429
H   0.72090 -2.38709  3.90798
C   4.00245 -3.26606 -1.67884
H   4.68465 -2.42087 -1.48399
C   4.84352 -4.46242 -2.17371
H   5.54996 -4.80608 -1.39997
H   5.42551 -4.17672 -3.06726
H   4.21224 -5.32361 -2.45738
C   3.00822 -2.81047 -2.77123
H   3.53453 -2.58685 -3.71630
H   2.46830 -1.89587 -2.46058
H   2.26032 -3.59740 -2.98265
K  -0.00002 -2.32754  0.00003
Si -5.76039 -1.16992 -1.04146
Si -5.76050  1.17052  1.04094
Al -2.86522 -0.00011  0.00051
O  -6.40873  0.00037 -0.00038
N  -4.05644 -1.40607 -0.60750
N  -4.05634  1.40631  0.60765
C  -3.38822 -2.65501 -0.75751
C  -2.71190 -3.00114 -1.97776
C  -1.95071 -4.18811 -2.04206
H  -1.44693 -4.44638 -2.98160
C  -1.83778 -5.05131 -0.94268
H  -1.25265 -5.97417 -1.01715
C  -2.50958 -4.72661  0.24567
H  -2.44200 -5.40713  1.10269
```

```
C  -3.28763 -3.55632  0.35888
C  -2.80453 -2.12415 -3.22656
H  -3.43409 -1.25977 -2.95969
C  -3.48446 -2.87714 -4.39281
H  -4.47460 -3.26538 -4.09990
H  -3.62056 -2.20812 -5.26068
H  -2.87802 -3.73759 -4.72841
C  -1.42857 -1.57430 -3.66310
H  -0.72091 -2.38762 -3.90783
H  -1.53008 -0.94403 -4.56414
H  -0.97992 -0.94964 -2.86922
C  -4.00332 -3.26587  1.67884
H  -4.68529 -2.42053  1.48390
C  -4.84478 -4.46211  2.17340
H  -4.21375 -5.32346  2.45712
H  -5.42690 -4.17636  3.06685
H  -5.55112 -4.80553  1.39946
C  -3.00925 -2.81061  2.77151
H  -2.46903 -1.89611  2.46107
H  -3.53575 -2.58694  3.71647
H  -2.26159 -3.59773  2.98305
C  -6.76683 -2.76444 -0.84683
H  -7.79974 -2.60521 -1.20056
H  -6.32780 -3.58701 -1.43688
H  -6.81361 -3.08390  0.20664
C  -6.02890 -0.48576 -2.79455
H  -5.41183  0.41175 -2.97004
H  -5.78759 -1.22615 -3.57541
H  -7.08744 -0.19749 -2.91657
C  -6.76651  2.76528  0.84599
H  -7.79948  2.60638  1.19967
H  -6.32729  3.58781  1.43595
H  -6.81312  3.08460 -0.20752
C  -6.02970  0.48641  2.79394
H  -5.41319 -0.41147  2.96946
H  -5.78801  1.22662  3.57485
H  -7.08843  0.19879  2.91586
C  -3.38799  2.65517  0.75760
C  -3.28705  3.55629 -0.35890
C  -2.50891  4.72653 -0.24569
H  -2.44108  5.40692 -1.10280
C  -1.83732  5.05131  0.94275
H  -1.25210  5.97412  1.01721
C  -1.95055  4.18827  2.04222
H  -1.44692  4.44662  2.98183
C  -2.71188  3.00138  1.97794
C  -4.00244  3.26567 -1.67900
H  -4.68466  2.42053 -1.48403
C  -4.84346  4.46195 -2.17415
H  -4.21216  5.32308 -2.45795
H  -5.42540  4.17609 -3.06768
H  -5.54995  4.80577 -1.40051
C  -3.00813  2.80986 -2.77122
H  -2.46825  1.89531 -2.46035
H  -3.53438  2.58605 -3.71629
H  -2.26021  3.59673 -2.98275
C  -2.80488  2.12458  3.22685
H  -3.43452  1.26026  2.95997
C  -3.48493  2.87784  4.39285
H  -4.47497  3.26616  4.09971
H  -3.62127  2.20897  5.26080
H  -2.87844  3.73827  4.72843
C  -1.42909  1.57458  3.66371
H  -1.53087  0.94445  4.56482
H  -0.98040  0.94975  2.86999
H  -0.72135  2.38783  3.90847
```

B

SCF (BP86) Energy = -3118.67687934
Enthalpy 0K = -3117.108814
Enthalpy 298K = -3117.003884
Free Energy 298K = -3117.257168
Lowest Frequency = 7.9080 cm^{-1}
Second Frequency = 11.1199 cm^{-1}
SCF (BP86-D3BJ) Energy = -3119.21463029
SCF (tol) Energy = -3118.68620809
SCF (BS2) Energy = -5886.07823998

```
K  -0.50237 -1.22639  1.94206
Si  4.62619  1.30869  2.74166
Si  4.94471  2.53612 -0.10508
Al  2.36034  0.71102  0.52970
O   5.38003  1.60748  1.24725
N   3.06991  0.55646  2.37422
N   3.20400  2.30827 -0.30364
C   2.35598 -0.22023  3.32748
C   2.52824 -1.65105  3.40202
C   1.75989 -2.41589  4.30460
H   1.91668 -3.50048  4.34650
C   0.81824 -1.82862  5.15847
H   0.24388 -2.43759  5.86461
C   0.64239 -0.44081  5.09953
H  -0.08224  0.03505  5.77120
C   1.38138  0.36993  4.21154
C   3.52553 -2.40282  2.51975
H   4.07899 -1.64281  1.94253
C   4.53857 -3.22556  3.34780
H   5.06241 -2.59868  4.08639
H   5.29764 -3.68013  2.68626
H   4.04589 -4.04938  3.89491
C   2.79135 -3.32503  1.51944
H   2.15670 -4.06086  2.04657
H   3.51284 -3.89451  0.90613
H   2.15280 -2.74303  0.83076
C   1.10108  1.87168  4.23863
H   1.74306  2.33043  3.46774
C   1.45005  2.48971  5.61255
H   0.77439  2.11595  6.40303
H   1.35050  3.58905  5.58322
H   2.48052  2.24694  5.91875
C  -0.37273  2.17786  3.88891
H  -0.62903  1.81786  2.87558
H  -0.56822  3.26346  3.91515
H  -1.06649  1.70119  4.60471
C   4.52775  2.96479  3.67671
H   5.52677  3.43482  3.68972
H   4.21296  2.82304  4.72396
H   3.82364  3.67031  3.20532
C   5.81299  0.19262  3.71561
H   6.13769 -0.66896  3.11096
H   5.34037 -0.18625  4.63791
H   6.71184  0.76347  4.00580
C   6.04317  1.87087 -1.50902
H   7.10337  1.98731 -1.22359
H   5.88522  2.42357 -2.45043
H   5.85620  0.80196 -1.69491
C   5.46601  4.34776  0.14712
H   5.07731  4.77221  1.08574
H   5.11835  4.97952 -0.68861
H   6.56779  4.40895  0.17681
C   2.42107  3.13406 -1.15376
C   2.16764  2.77156 -2.52536
C   1.30398  3.55709 -3.31827
H   1.12801  3.26544 -4.36092
C   0.68668  4.71060 -2.81683
```

H	0.03267	5.31609	-3.45280
C	0.94471	5.08635	-1.49152
H	0.48117	5.99676	-1.09291
C	1.79741	4.33425	-0.65750
C	2.82424	1.55415	-3.17646
H	3.53260	1.14604	-2.43764
C	3.61635	1.94102	-4.44578
H	2.95055	2.29206	-5.25451
H	4.17432	1.07113	-4.83572
H	4.34127	2.74601	-4.23990
C	1.79188	0.44942	-3.49805
H	1.31908	0.07010	-2.57343
H	2.27322	-0.40774	-4.00370
H	1.00086	0.82283	-4.17514
C	2.00828	4.81664	0.77607
H	2.77502	4.15798	1.21745
C	2.50233	6.27825	0.84732
H	3.41404	6.43145	0.24851
H	2.72898	6.55578	1.89200
H	1.73646	6.98558	0.48136
C	0.71880	4.65188	1.60724
H	0.88087	4.96410	2.65429
H	0.38823	3.59944	1.61940
H	-0.10256	5.26958	1.19886
K	-0.81013	2.09137	-1.13921
Si	-6.61809	0.38618	-0.82946
Si	-5.75566	-2.60774	-0.50546
Al	-3.46283	-0.27959	-0.10132
O	-6.80889	-1.29148	-0.67426
N	-4.88339	0.73117	-0.95738
N	-4.25437	-2.02037	0.23498
C	-4.37628	1.86435	-1.65945
C	-4.19685	3.13168	-1.00709
C	-3.56720	4.18916	-1.69701
H	-3.44178	5.15410	-1.19081
C	-3.11547	4.04206	-3.01583
H	-2.64114	4.87944	-3.53812
C	-3.30420	2.81289	-3.66392
H	-2.97119	2.69700	-4.70224
C	-3.93146	1.72726	-3.01982
C	-4.68400	3.37857	0.42063
H	-5.15449	2.44204	0.76046
C	-5.74602	4.50046	0.46919
H	-6.58662	4.29063	-0.21371
H	-6.15157	4.60781	1.49066
H	-5.31920	5.47691	0.17736
C	-3.52261	3.68637	1.39087
H	-2.96338	4.58987	1.08555
H	-3.90351	3.86235	2.41246
H	-2.81623	2.83818	1.44048
C	-4.11964	0.41912	-3.78894
H	-4.77659	-0.21408	-3.16825
C	-4.80310	0.62929	-5.15742
H	-4.16560	1.20219	-5.85460
H	-5.01397	-0.34439	-5.63348
H	-5.75647	1.17320	-5.05241
C	-2.78152	-0.33529	-3.95899
H	-2.33158	-0.57081	-2.97597
H	-2.93004	-1.28678	-4.50038
H	-2.05404	0.26763	-4.53388
C	-7.56592	0.96906	-2.36540
H	-8.64908	0.81259	-2.22494
H	-7.39824	2.04359	-2.55231
H	-7.25498	0.41189	-3.26381
C	-7.46120	1.10198	0.71671
H	-6.90834	0.82889	1.63154
H	-7.54953	2.20050	0.68219
H	-8.47895	0.68256	0.80011
C	-6.61613	-3.90329	0.57847
H	-7.49521	-4.31377	0.05299
H	-5.93766	-4.74073	0.81433
H	-6.96214	-3.46335	1.52757
C	-5.53763	-3.32527	-2.25341
H	-4.98334	-2.63058	-2.90691
H	-5.00192	-4.28924	-2.24576
H	-6.53108	-3.49398	-2.70420
C	-3.47468	-2.82926	1.11009
C	-3.64567	-2.72455	2.53445
C	-2.78349	-3.43340	3.39569
H	-2.92309	-3.34631	4.47957
C	-1.76532	-4.26161	2.90000
H	-1.11615	-4.81618	3.58587
C	-1.60844	-4.38543	1.51208
H	-0.82748	-5.04633	1.11640
C	-2.43996	-3.69087	0.60767
C	-4.74554	-1.85092	3.13995
H	-5.40347	-1.55388	2.30533
C	-5.59815	-2.60793	4.18110
H	-5.01490	-2.87096	5.08185
H	-6.44313	-1.97933	4.51233
H	-6.00898	-3.54237	3.76397
C	-4.16539	-0.55492	3.75109
H	-3.63216	0.04029	2.98567
H	-4.96814	0.07643	4.17208
H	-3.45424	-0.78263	4.56691
C	-2.21884	-3.89153	-0.89179
H	-2.97066	-3.27187	-1.40688
C	-2.44089	-5.36509	-1.30188
H	-3.43581	-5.72555	-0.99027
H	-2.36143	-5.48055	-2.39732
H	-1.68917	-6.03176	-0.84197
C	-0.82711	-3.40331	-1.35111
H	-0.70047	-3.56052	-2.43713
H	-0.69376	-2.32428	-1.15256
H	-0.01374	-3.95060	-0.83984
C	6.92335	-3.74605	-3.54582
H	7.75933	-3.07488	-3.27194
H	7.33771	-4.51956	-4.21942
C	5.81475	-2.94910	-4.27357
H	6.23308	-2.46333	-5.17451
C	6.33313	-4.40874	-2.27818
H	7.12331	-4.97234	-1.74840
C	5.78562	-3.31476	-1.33056
H	6.58747	-2.61879	-1.02441
H	5.37195	-3.76647	-0.41083
C	5.26438	-1.85185	-3.33152
H	4.47827	-1.26139	-3.83586
H	6.06551	-1.15045	-3.03580
C	4.66681	-2.51464	-2.05755
C	3.51890	-3.47952	-2.46921
H	3.08106	-3.93152	-1.56116
H	2.72017	-2.90047	-2.96720
C	4.66860	-3.90671	-4.67877
H	5.04979	-4.68117	-5.37062
H	3.87972	-3.35066	-5.22017
C	5.18624	-5.36712	-2.68024
H	5.57527	-6.16681	-3.33817
H	4.77014	-5.86250	-1.78234
C	4.07860	-4.57153	-3.41203
H	3.25459	-5.25214	-3.69593
C	3.72760	-0.64406	-0.46948
N	4.16095	-1.49438	-1.18285

B′

SCF (BP86) Energy = -3118.68086908

Enthalpy 0K = -3117.114231

Enthalpy 298K = -3117.007957
Free Energy 298K = -3117.270293
Lowest Frequency = 6.0377 cm^{-1}
Second Frequency = 8.1181 cm^{-1}
SCF (BP86-D3BJ) Energy = -3119.20494015
SCF (tol) Energy = -3118.69069946
SCF (BS2) Energy = -5886.08351248

```
K  -0.80777  1.13892  1.07006
Si -3.41514 -4.41065  2.12197
Si -3.15653 -5.13011 -0.89647
Al -1.44739 -2.55427  0.25798
O  -3.72134 -5.28017  0.69583
N  -2.74235 -2.83464  1.67489
N  -1.70201 -4.11297 -0.88203
C  -3.02506 -1.63785  2.39654
C  -4.11001 -0.79224  1.98493
C  -4.28905  0.46138  2.60070
H  -5.11417  1.10429  2.27374
C  -3.44025  0.90457  3.62555
H  -3.60091  1.87980  4.09843
C  -2.40408  0.06573  4.05896
H  -1.76014  0.38921  4.88623
C  -2.18419 -1.20020  3.47462
C  -5.06628 -1.23150  0.87536
H  -4.89943 -2.31248  0.73036
C  -6.55000 -1.02176  1.24393
H  -6.79935 -1.50119  2.20538
H  -7.20218 -1.45492  0.46525
H  -6.81016  0.04916  1.32777
C  -4.73316 -0.54026 -0.46480
H  -4.83192  0.55646 -0.38113
H  -5.40942 -0.88727 -1.26732
H  -3.69353 -0.75599 -0.77513
C  -1.08092 -2.09254  4.04282
H  -1.05034 -2.99632  3.41195
C  -1.41269 -2.52500  5.49012
H  -1.43660 -1.65713  6.17367
H  -0.65257 -3.22982  5.87139
H  -2.39801 -3.01791  5.54808
C   0.31361 -1.43577  3.98017
H   0.59649 -1.19926  2.93864
H   1.08293 -2.11559  4.38680
H   0.35625 -0.50245  4.57036
C  -2.22344 -5.49110  3.13545
H  -2.62925 -6.51403  3.22139
H  -2.07462 -5.09806  4.15526
H  -1.23621 -5.55727  2.64787
C  -5.04664 -4.24800  3.07531
H  -5.83999 -3.81073  2.44811
H  -4.92388 -3.61059  3.96753
H  -5.38716 -5.24241  3.41106
C  -4.59820 -4.38678 -1.88823
H  -5.51025 -4.98565 -1.72058
H  -4.39354 -4.37682 -2.97212
H  -4.80770 -3.35202 -1.56987
C  -2.80652 -6.86998 -1.56626
H  -2.13635 -7.43412 -0.89820
H  -2.34051 -6.82637 -2.56542
H  -3.75077 -7.43401 -1.65525
C  -0.63672 -4.30762 -1.80632
C  -0.58857 -3.59419 -3.05345
C   0.54546 -3.71636 -3.88488
H   0.56769 -3.17158 -4.83669
C   1.63118 -4.53073 -3.53470
H   2.49623 -4.62209 -4.20005
C   1.58126 -5.24502 -2.32780
H   2.41964 -5.89670 -2.05569
C   0.47277 -5.15574 -1.46176
C  -1.74956 -2.71888 -3.52630
H  -2.51925 -2.76349 -2.73803
C  -2.36518 -3.26417 -4.83534
H  -1.64682 -3.21254 -5.67331
H  -3.25379 -2.67463 -5.12246
H  -2.67252 -4.31824 -4.72860
C  -1.34198 -1.23819 -3.69272
H  -0.98817 -0.81812 -2.73398
H  -2.20342 -0.63308 -4.02748
H  -0.54051 -1.11585 -4.44442
C   0.48436 -5.94769 -0.15348
H  -0.54502 -5.91403  0.24192
C   0.88062 -7.42644 -0.34787
H   0.25206 -7.91578 -1.11031
H   0.76403 -7.98009  0.60006
H   1.93454 -7.53678 -0.66076
C   1.39630 -5.27345  0.89648
H   1.39355 -5.83777  1.84619
H   1.05509 -4.24400  1.11717
H   2.44205 -5.22164  0.53984
K   1.90343 -2.00233 -1.21896
Si  6.30547  1.68608  0.18443
Si  4.56156  4.26947  0.03450
Al  3.04302  1.29962  0.02149
O   5.95203  3.31422 -0.12783
N   4.88412  0.72856 -0.26609
N   3.20429  3.19702  0.43191
C   5.00249 -0.55783 -0.85991
C   5.09610 -1.74544 -0.05561
C   5.09740 -3.01247 -0.67644
H   5.17825 -3.91060 -0.05189
C   5.01289 -3.14935 -2.06959
H   5.02474 -4.14133 -2.53324
C   4.93556 -1.99348 -2.86085
H   4.88962 -2.09282 -3.95194
C   4.93910 -0.70406 -2.28991
C   5.20837 -1.67326  1.46704
H   5.19354 -0.60383  1.73259
C   6.53956 -2.27919  1.96606
H   7.40780 -1.79964  1.48307
H   6.63973 -2.15048  3.05828
H   6.59833 -3.36177  1.75277
C   4.00723 -2.33767  2.17407
H   3.92573 -3.41091  1.92307
H   4.11024 -2.26087  3.27086
H   3.05942 -1.84357  1.89475
C   4.88284  0.51484 -3.21274
H   5.06351  1.39785 -2.57575
C   5.97319  0.47488 -4.30591
H   5.80769 -0.34807 -5.02442
H   5.97255  1.41692 -4.88164
H   6.97708  0.34018 -3.87011
C   3.48451  0.68442 -3.84969
H   2.70844  0.81704 -3.07226
H   3.45659  1.57074 -4.50833
H   3.21507 -0.19632 -4.46242
C   7.82387  1.23124 -0.85452
H   8.70870  1.78347 -0.49464
H   8.04529  0.15238 -0.79118
H   7.67029  1.48910 -1.91475
C   6.76612  1.57788  2.02677
H   5.88854  1.75315  2.67163
H   7.19257  0.59558  2.29088
H   7.52072  2.34826  2.26269
C   4.85292  5.57608  1.38141
H   5.63026  6.28766  1.05405
```

```
H   3.93196  6.14867  1.58603
H   5.18794  5.11654  2.32510
C   4.43936  5.17309 -1.63450
H   4.25158  4.46849 -2.46197
H   3.64912  5.94181 -1.64708
H   5.40235  5.67545 -1.83323
C   2.02201  3.70158  1.05517
C   1.86786  3.61941  2.48178
C   0.64379  3.98726  3.07530
H   0.53655  3.91585  4.16424
C  -0.43124  4.45907  2.30893
H  -1.37052  4.75395  2.78978
C  -0.27472  4.57260  0.92051
H  -1.10630  4.95304  0.31682
C   0.92556  4.20581  0.27701
C   3.00647  3.13012  3.37724
H   3.90389  3.08089  2.73744
C   3.29897  4.09547  4.54619
H   2.46033  4.14096  5.26399
H   4.18860  3.75848  5.10691
H   3.48891  5.12021  4.18642
C   2.73926  1.70312  3.90429
H   2.60641  0.98988  3.06977
H   3.57929  1.34844  4.52804
H   1.82433  1.67357  4.52431
C   1.01940  4.35725 -1.24219
H   2.05721  4.11536 -1.51983
C   0.72191  5.80340 -1.69655
H   1.36075  6.53306 -1.17078
H   0.89722  5.91237 -2.78139
H  -0.32897  6.08474 -1.50179
C   0.10627  3.35629 -1.98478
H   0.18728  3.49007 -3.07865
H   0.39787  2.31535 -1.75263
H  -0.95453  3.48940 -1.70515
C  -7.82213  4.53985 -1.99077
H  -8.20273  3.50965 -1.85611
H  -8.65456  5.13935 -2.40421
C  -6.62791  4.54383 -2.97527
H  -6.94166  4.11843 -3.94624
C  -7.37383  5.12603 -0.63044
H  -8.22194  5.11809  0.07869
C  -6.23190  4.25983 -0.04636
H  -6.56927  3.21940  0.11221
H  -5.89944  4.65448  0.93114
C  -5.48291  3.67519 -2.40122
H  -4.61942  3.65619 -3.09083
H  -5.81441  2.63106 -2.25609
C  -5.03279  4.26322 -1.03479
C  -4.52819  5.71909 -1.23531
H  -4.18654  6.12370 -0.26505
H  -3.65896  5.71251 -1.91789
C  -6.12463  5.99357 -3.17593
H  -6.92965  6.61660 -3.60844
H  -5.28324  6.01004 -3.89443
C  -6.87092  6.57607 -0.83036
H  -7.68806  7.20802 -1.22558
H  -6.56699  7.01249  0.14007
C  -5.67696  6.57932 -1.81520
H  -5.30927  7.61253 -1.95473
N  -3.96861  3.45556 -0.49828
C  -3.09817  2.77434 -0.06853
```

C

SCF (BP86) Energy = -3601.60879505
Enthalpy 0K = -3599.805829
Enthalpy 298K = -3599.689167
Free Energy 298K = -3599.972500
Lowest Frequency = 4.4983 cm^{-1}
Second Frequency = 7.5010 cm^{-1}
SCF (BP86-D3BJ) Energy = -3602.20573291
SCF (tol) Energy = -3601.61989371
SCF (BS2) Energy = -6369.12173088

```
K  -0.94662  0.58102  2.19949
Si  0.86740 -4.79071  1.15733
Si -0.06102 -4.97728 -1.81006
Al -0.76526 -2.32259 -0.11774
O   0.04775 -5.44466 -0.18211
N   0.23005 -3.15899  1.38094
N  -0.18708 -3.21578 -1.79822
C   0.33504 -2.45514  2.61209
C  -0.73026 -2.49689  3.58486
C  -0.65092 -1.72660  4.76405
H  -1.47145 -1.78260  5.48964
C   0.45194 -0.90949  5.04118
H   0.50262 -0.33064  5.96964
C   1.49800 -0.86754  4.11115
H   2.37600 -0.24524  4.32181
C   1.46757 -1.61468  2.91395
C  -1.97744 -3.36128  3.39697
H  -1.83567 -3.92689  2.46040
C  -2.16858 -4.36753  4.55473
H  -1.27394 -4.99343  4.69891
H  -3.02344 -5.03635  4.34793
H  -2.37772 -3.85431  5.51067
C  -3.24361 -2.48770  3.24558
H  -3.39468 -1.84584  4.13288
H  -4.14546 -3.11766  3.14074
H  -3.18104 -1.83940  2.35316
C   2.67442 -1.49390  1.98545
H   2.46120 -2.11040  1.09618
C   3.96266 -2.02214  2.65897
H   4.25168 -1.38682  3.51632
H   4.79839 -2.01498  1.93810
H   3.83299 -3.04847  3.04051
C   2.90045 -0.03795  1.51943
H   2.02843  0.34673  0.95901
H   3.78245  0.02287  0.86012
H   3.07733  0.63770  2.37625
C   2.73206 -4.90554  0.79088
H   2.97639 -5.93747  0.48293
H   3.34057 -4.66884  1.67919
H   3.04092 -4.22373 -0.01856
C   0.51268 -5.96013  2.61079
H  -0.56639 -6.15910  2.70854
H   0.87705 -5.53702  3.56258
H   1.02447 -6.92487  2.45117
C  -1.57686 -5.91852 -2.47199
H  -1.41840 -7.00251 -2.33429
H  -1.73433 -5.73514 -3.54822
H  -2.49378 -5.63680 -1.93149
C   1.42874 -5.64268 -2.78776
H   2.39120 -5.34765 -2.34185
H   1.40699 -5.28407 -3.83148
H   1.38746 -6.74559 -2.81142
C  -0.03994 -2.44080 -2.97968
C  -1.18255 -2.05882 -3.77156
C  -1.02006 -1.21417 -4.89012
H  -1.90273 -0.93916 -5.48027
C   0.23942 -0.74065 -5.28028
H   0.34763 -0.10149 -6.16280
C   1.35972 -1.11934 -4.52811
H   2.35226 -0.76306 -4.82861
C   1.25076 -1.95412 -3.39623
```

C	-2.59241	-2.55806	-3.45537
H	-2.49210	-3.27859	-2.62783
C	-3.22377	-3.28917	-4.66192
H	-3.41738	-2.60032	-5.50377
H	-4.19162	-3.74083	-4.37991
H	-2.56867	-4.09375	-5.03519
C	-3.51412	-1.41400	-2.97430
H	-3.13697	-0.97029	-2.03472
H	-4.53772	-1.78558	-2.78300
H	-3.59490	-0.61586	-3.73584
C	2.52653	-2.27270	-2.61843
H	2.25046	-3.01276	-1.84869
C	3.63808	-2.87051	-3.50874
H	3.29042	-3.76097	-4.05665
H	4.50427	-3.16235	-2.88967
H	4.00249	-2.13853	-4.25206
C	3.05083	-1.01557	-1.89169
H	3.97485	-1.22833	-1.32640
H	2.30157	-0.63362	-1.17587
H	3.28317	-0.20974	-2.61354
K	-0.29576	0.83025	-2.27893
Si	-0.67335	6.71840	-0.89876
Si	-2.95369	6.11774	1.15798
Al	-1.14278	3.59571	0.04278
O	-1.93986	7.05733	0.17680
N	-0.69150	4.98026	-1.24065
N	-2.19653	4.52936	1.38286
C	-0.28419	4.43815	-2.49455
C	1.08447	4.08410	-2.74871
C	1.42364	3.42873	-3.95119
H	2.47289	3.16880	-4.13714
C	0.45733	3.11696	-4.91747
H	0.74336	2.61779	-5.84919
C	-0.87785	3.47626	-4.68242
H	-1.63533	3.25257	-5.44303
C	-1.26696	4.13685	-3.49965
C	2.19922	4.41646	-1.75711
H	1.71940	4.90998	-0.89640
C	3.22121	5.40008	-2.37180
H	2.72908	6.31532	-2.74233
H	3.97670	5.69532	-1.62225
H	3.75804	4.94536	-3.22384
C	2.91169	3.15379	-1.22632
H	3.38664	2.57656	-2.04033
H	3.70552	3.42642	-0.50867
H	2.20255	2.49009	-0.70035
C	-2.73646	4.51436	-3.30844
H	-2.77449	5.18092	-2.42970
C	-3.31712	5.27739	-4.51835
H	-3.37523	4.64180	-5.42021
H	-4.34202	5.62306	-4.29659
H	-2.70478	6.15933	-4.76995
C	-3.59927	3.27473	-2.98041
H	-3.24349	2.77793	-2.05779
H	-4.65634	3.55693	-2.82674
H	-3.56519	2.53627	-3.80308
C	-0.93268	7.74718	-2.47062
H	-0.84660	8.82269	-2.23967
H	-0.17786	7.50106	-3.23686
H	-1.93053	7.57057	-2.90360
C	0.89542	7.32879	-0.01452
H	1.11125	6.71416	0.87600
H	1.78295	7.30857	-0.66866
H	0.74463	8.36942	0.32155
C	-3.18245	7.02722	2.80613
H	-3.72665	7.97345	2.64496
H	-3.76122	6.41808	3.52126
H	-2.21092	7.26760	3.26708

C	-4.62578	6.06910	0.25262
H	-4.55491	5.49436	-0.68631
H	-5.42403	5.62243	0.86853
H	-4.93123	7.09858	-0.00336
C	-2.36768	3.76808	2.57483
C	-1.38406	3.83650	3.62180
C	-1.49109	2.98355	4.73899
H	-0.73442	3.04378	5.53008
C	-2.55020	2.07339	4.87134
H	-2.62351	1.42950	5.75421
C	-3.52466	2.01764	3.86471
H	-4.36623	1.32197	3.96954
C	-3.45829	2.84361	2.72229
C	-0.21096	4.81491	3.54653
H	-0.42277	5.49135	2.70064
C	-0.07004	5.66924	4.82500
H	0.21339	5.05878	5.70121
H	0.71710	6.43170	4.69046
H	-1.01176	6.18815	5.06971
C	1.11486	4.08506	3.23164
H	1.05165	3.54381	2.26882
H	1.95280	4.80167	3.16269
H	1.36369	3.35209	4.02142
C	-4.56738	2.73374	1.67572
H	-4.32166	3.45433	0.87882
C	-5.94187	3.11665	2.27017
H	-5.91863	4.11939	2.72937
H	-6.71940	3.11579	1.48585
H	-6.25758	2.40265	3.05227
C	-4.62840	1.33258	1.02805
H	-5.43201	1.29049	0.27130
H	-3.67960	1.08018	0.52052
H	-4.83718	0.54600	1.77647
C	-7.17741	-6.32522	-0.32717
H	-6.55607	-7.20472	-0.58160
H	-8.23095	-6.66273	-0.32792
C	-6.97879	-5.20853	-1.37968
H	-7.24050	-5.59015	-2.38399
C	-6.79434	-5.78779	1.07243
H	-6.92606	-6.58450	1.82789
C	-5.31046	-5.34929	1.06835
H	-4.64738	-6.19835	0.82242
H	-5.01064	-4.97471	2.06384
C	-5.49590	-4.76624	-1.39029
H	-5.32880	-3.97525	-2.14363
H	-4.83543	-5.61359	-1.64920
C	-5.10679	-4.22317	0.01434
C	-6.01460	-3.01027	0.36505
H	-5.72187	-2.61043	1.35234
H	-5.85008	-2.20838	-0.37737
C	-7.87733	-3.99874	-1.02806
H	-8.94129	-4.30167	-1.04026
H	-7.75999	-3.20236	-1.78745
C	-7.69082	-4.57685	1.42639
H	-8.75212	-4.88780	1.45553
H	-7.43850	-4.19695	2.43473
C	-7.49510	-3.46119	0.37167
H	-8.13014	-2.59192	0.62472
C	-2.57917	-3.50805	-0.00658
N	-3.72861	-3.82246	0.00619
C	6.45983	-1.04975	-0.07538
N	7.63476	-1.00090	0.10088
C	9.05511	-0.94688	0.31948
C	9.54195	0.52475	0.21117
C	9.77820	-1.81146	-0.75011
C	9.38118	-1.49681	1.73590
H	9.28538	0.91964	-0.78879
H	9.00606	1.14025	0.95657

C 11.07026 0.57689 0.44907
H   9.52304 -1.43251 -1.75658
H   9.40978 -2.85151 -0.68586
C 11.30566 -1.75143 -0.50927
H   8.84419 -0.89474 2.49134
H   9.00976 -2.53462 1.81652
C 10.90982 -1.43817 1.96871
H 11.40720 1.62714 0.37018
C 11.78793 -0.28469 -0.61779
C 11.39216 0.02854 1.86026
H 11.81128 -2.36892 -1.27465
C 11.62772 -2.29959 0.90190
H 11.13165 -1.83124 2.97817
H 12.88315 -0.23482 -0.46991
H 11.58093 0.11116 -1.63029
H 10.90011 0.65008 2.63243
H 12.48103 0.08359 2.04789
H 11.30543 -3.35507 0.98378
H 12.72037 -2.28188 1.07415

TS(C-D)

SCF (BP86) Energy = -3601.60921104
Enthalpy 0K = -3599.806183
Enthalpy 298K = -3599.690504
Free Energy 298K = -3599.970371
Lowest Frequency = -21.5680 cm^{-1}
Second Frequency = 3.6627 cm^{-1}
SCF (BP86-D3BJ) Energy = -3602.21342579
SCF (tol) Energy = -3601.62041651
SCF (BS2) Energy = -6369.11923836

K -1.62898 1.21350 2.05286
Si -2.51630 -4.50539 2.40706
Si -1.84998 -5.13861 -0.57123
Al -1.73198 -2.07070 0.43301
O -2.78322 -5.12808 0.84668
N -2.24262 -2.77175 2.22168
N -0.98861 -3.59755 -0.60059
C -2.41644 -1.84020 3.28173
C -3.68108 -1.17972 3.49437
C -3.81034 -0.19114 4.49207
H -4.78473 0.29106 4.63624
C -2.73693 0.18095 5.31034
H -2.86119 0.94206 6.08805
C -1.50599 -0.45944 5.12321
H -0.66075 -0.19249 5.76922
C -1.32319 -1.45449 4.13956
C -4.92321 -1.50296 2.66334
H -4.64802 -2.32648 1.98250
C -6.11071 -1.96081 3.54074
H -5.83649 -2.81117 4.18434
H -6.96096 -2.27204 2.90756
H -6.47046 -1.14627 4.19499
C -5.34684 -0.29091 1.80346
H -5.57137 0.58838 2.43436
H -6.25935 -0.52133 1.22412
H -4.55515 -0.01005 1.08697
C   0.05766 -2.10044 4.04371
H   0.01377 -2.82098 3.20995
C   0.41421 -2.86419 5.34023
H   0.53729 -2.17199 6.19287
H   1.36318 -3.41632 5.22013
H -0.37098 -3.58773 5.61406
C   1.15671 -1.06065 3.72961
H   0.96600 -0.55748 2.76377
H   2.14850 -1.54091 3.66581
H   1.21660 -0.28324 4.51266
C -1.06533 -5.47179 3.17634
H -1.25990 -6.55525 3.08921
H -0.94869 -5.23930 4.24830
H -0.10609 -5.25403 2.67836
C -4.06048 -4.95500 3.41663
H -4.98360 -4.66079 2.89282
H -4.04912 -4.46622 4.40587
H -4.09410 -6.04618 3.57924
C -3.10458 -5.41323 -1.97591
H -3.64708 -6.35879 -1.80071
H -2.60464 -5.48775 -2.95659
H -3.84312 -4.59854 -2.02119
C -0.72059 -6.67155 -0.59524
H -0.14254 -6.78770 0.33474
H -0.01128 -6.62816 -1.43988
H -1.34062 -7.57573 -0.72437
C   0.12300 -3.36546 -1.45412
C -0.03650 -2.72761 -2.73528
C   1.09684 -2.40817 -3.51284
H   0.95322 -1.92095 -4.48512
C   2.39495 -2.72070 -3.08735
H   3.26200 -2.47669 -3.71075
C   2.55878 -3.37511 -1.85795
H   3.56855 -3.62684 -1.51680
C   1.45920 -3.70569 -1.04031
C -1.41444 -2.40525 -3.31171
H -2.15376 -2.80932 -2.60120
C -1.63268 -3.09360 -4.67843
H -0.95639 -2.68919 -5.45295
H -2.66728 -2.93650 -5.03228
H -1.45477 -4.18032 -4.61700
C -1.65030 -0.88316 -3.42579
H -1.59476 -0.40093 -2.43335
H -2.64677 -0.66770 -3.85332
H -0.90240 -0.40936 -4.08823
C   1.72673 -4.38114 0.30275
H   0.74740 -4.71114 0.68505
C   2.65336 -5.61003 0.19206
H   2.27579 -6.34187 -0.54041
H   2.73385 -6.11667 1.17026
H   3.67700 -5.32575 -0.11187
C   2.29331 -3.36611 1.31657
H   2.44653 -3.83585 2.30525
H   1.59212 -2.52488 1.45211
H   3.26202 -2.95659 0.97724
K   1.66908 -0.20298 -0.96819
Si 2.62720 5.98257 -0.80662
Si -0.34965 6.58053 -0.12378
Al 0.63084 3.44980 0.07290
O   1.31344 6.89037 -0.23694
N   2.11302 4.28698 -0.88000
N -0.54622 4.94001 0.51414
C   2.83698 3.32328 -1.64697
C   3.92564 2.58143 -1.07570
C   4.52423 1.54078 -1.81651
H   5.34320 0.97006 -1.36582
C   4.08861 1.21032 -3.10740
H   4.57152 0.40207 -3.66757
C   3.04026 1.94573 -3.67821
H   2.70541 1.70473 -4.69420
C   2.41313 2.99784 -2.98151
C   4.45603 2.88134 0.32723
H   3.93407 3.78621 0.67555
C   5.97346 3.17080 0.32007
H   6.23153 3.96710 -0.39858
H   6.31029 3.49033 1.32202
H   6.55823 2.27455 0.04525
C   4.12482 1.74996 1.32667

```
H   4.55183  0.78346  1.00210
H   4.53135  1.98199  2.32772
H   3.03031  1.63228  1.43107
C   1.27553  3.75920 -3.66239
H   1.05949  4.63056 -3.02114
C   1.66068  4.27508 -5.06564
H   1.83277  3.44732 -5.77692
H   0.84821  4.89707 -5.48116
H   2.57740  4.88673 -5.03386
C  -0.01132  2.90738 -3.72960
H  -0.33338  2.59590 -2.71845
H  -0.83780  3.47545 -4.19323
H   0.14759  1.99335 -4.33101
C   3.16770  6.63976 -2.50549
H   3.57856  7.65887 -2.40300
H   3.95175  6.00001 -2.94607
H   2.32317  6.68238 -3.21193
C   4.01405  6.38162  0.43115
H   3.76247  6.03087  1.44588
H   4.98278  5.94196  0.14166
H   4.14043  7.47768  0.47590
C  -1.08152  7.88897  1.03599
H  -1.02353  8.88614  0.56722
H  -2.14052  7.67856  1.26221
H  -0.52772  7.92791  1.98770
C  -1.07669  6.83580 -1.86425
H  -0.73740  6.05417 -2.56484
H  -2.17971  6.82919 -1.85618
H  -0.74814  7.81279 -2.25963
C  -1.60201  4.60391  1.40530
C  -1.38349  4.61354  2.82729
C  -2.40020  4.16035  3.69281
H  -2.22281  4.17132  4.77475
C  -3.63854  3.71594  3.20593
H  -4.41957  3.38053  3.89635
C  -3.86631  3.72814  1.82216
H  -4.83852  3.39976  1.43477
C  -2.87839  4.16291  0.91362
C  -0.06730  5.11108  3.42776
H   0.50192  5.56858  2.60031
C  -0.28602  6.18478  4.51620
H  -0.78830  5.77136  5.40938
H   0.68351  6.59612  4.84761
H  -0.90275  7.01920  4.14288
C   0.78220  3.94442  3.98136
H   1.02370  3.21848  3.18241
H   1.73447  4.31509  4.40090
H   0.24665  3.40766  4.78664
C  -3.20292  4.16663 -0.58006
H  -2.30442  4.54350 -1.09507
C  -4.37978  5.11634 -0.89882
H  -4.18573  6.13773 -0.52981
H  -4.55141  5.17179 -1.98850
H  -5.31912  4.76928 -0.43148
C  -3.48440  2.74903 -1.12373
H  -3.71244  2.78773 -2.20398
H  -2.61186  2.08427 -0.99164
H  -4.34968  2.28318 -0.61745
C  -8.06519 -2.79422 -3.60826
H  -7.90013 -3.88617 -3.53923
H  -8.96839 -2.64255 -4.22885
C  -6.84612 -2.11077 -4.27226
H  -6.67704 -2.54135 -5.27666
C  -8.28540 -2.19747 -2.19768
H  -9.15089 -2.68816 -1.71495
C  -7.02803 -2.44544 -1.33038
H  -6.82468 -3.52676 -1.22830
H  -7.16842 -2.03883 -0.31246
C  -5.58381 -2.35606 -3.41131
H  -4.69758 -1.88374 -3.87237
H  -5.37332 -3.43723 -3.32272
C  -5.79967 -1.75692 -1.99223
C  -6.06311 -0.22970 -2.11663
H  -6.19416  0.19951 -1.10731
H  -5.17899  0.25512 -2.56860
C  -7.10500 -0.58979 -4.39080
H  -7.99352 -0.40537 -5.02364
H  -6.24805 -0.09396 -4.88510
C  -8.54314 -0.67572 -2.31281
H  -9.45547 -0.49089 -2.91068
H  -8.72145 -0.24202 -1.31036
C  -7.32524  0.00709 -2.98044
H  -7.50299  1.09575 -3.05838
C  -3.66921 -2.24367 -0.53421
N  -4.63127 -1.99431 -1.19248
C   5.14862 -1.54557 -0.20584
N   6.25097 -1.98278 -0.14109
C   7.58706 -2.51100 -0.05711
C   8.61060 -1.34752 -0.17477
C   7.81701 -3.52325 -1.21359
C   7.77340 -3.23062  1.30758
H   8.45710 -0.82349 -1.13577
H   8.42597 -0.61762  0.63427
C  10.04550 -1.92003 -0.08346
H   7.65810 -3.01266 -2.18096
H   7.07048 -4.33501 -1.14049
C   9.25500 -4.08753 -1.11861
H   7.58326 -2.51267  2.12607
H   7.02614 -4.03993  1.39701
C   9.21159 -3.79598  1.39289
H  10.76600 -1.08570 -0.16793
C  10.27315 -2.92764 -1.23592
C  10.22981 -2.63609  1.27610
H   9.40915 -4.80616 -1.94456
C   9.43926 -4.80405  0.24080
H   9.33485 -4.30580  2.36616
H  11.30466 -3.32459 -1.19257
H  10.16385 -2.42077 -2.21354
H  10.08939 -1.91930  2.10736
H  11.26060 -3.02847  1.35970
H   8.72961 -5.64876  0.32654
H  10.45752 -5.23079  0.30779
```

D

SCF (BP86) Energy = -3601.61027016
Enthalpy 0K = -3599.807049
Enthalpy 298K = -3599.690553
Free Energy 298K = -3599.973212
Lowest Frequency = 3.4084 cm^{-1}
Second Frequency = 7.2272 cm^{-1}
SCF (BP86-D3BJ) Energy = -3602.21848417
SCF (tol) Energy = -3601.62151749
SCF (BS2) Energy = -6369.11921771

```
K  -1.16803  1.33777  2.07691
Si -3.24823 -4.09809  2.32709
Si -2.84395 -4.79953 -0.67962
Al -1.96485 -1.87188  0.36994
O  -3.70825 -4.59982  0.76867
N  -2.58305 -2.47178  2.15902
N  -1.63451 -3.51393 -0.70528
C  -2.49161 -1.55621  3.24197
C  -3.57036 -0.64738  3.54307
C  -3.42878  0.31180  4.56738
H  -4.26640  0.98682  4.78040
```

C	-2.25589	0.41914	5.32500
H	-2.17012	1.16320	6.12406
C	-1.20257	-0.46114	5.04844
H	-0.28455	-0.40111	5.64574
C	-1.29365	-1.44024	4.03643
C	-4.89071	-0.66360	2.77179
H	-4.84183	-1.51475	2.07150
C	-6.11236	-0.85224	3.69954
H	-6.01237	-1.75195	4.32648
H	-7.03716	-0.95281	3.10351
H	-6.25002	0.01320	4.37255
C	-5.06116	0.62814	1.94093
H	-5.04450	1.52445	2.58737
H	-6.02846	0.62546	1.40621
H	-4.26141	0.72997	1.18660
C	-0.09453	-2.36657	3.84354
H	-0.34459	-3.03666	3.00392
C	0.16147	-3.22652	5.10298
H	0.48106	-2.60277	5.95746
H	0.95897	-3.96697	4.91451
H	-0.74521	-3.77125	5.41304
C	1.18546	-1.58309	3.47477
H	1.05545	-1.03034	2.52601
H	2.04345	-2.26631	3.34938
H	1.45681	-0.85420	4.25994
C	-2.03222	-5.38628	3.02791
H	-2.47102	-6.39553	2.93771
H	-1.82951	-5.20574	4.09723
H	-1.06638	-5.38205	2.49674
C	-4.81768	-4.20899	3.39049
H	-5.66849	-3.69963	2.91130
H	-4.65971	-3.76335	4.38748
H	-5.09231	-5.26836	3.53413
C	-4.16466	-4.72473	-2.04862
H	-4.91250	-5.51880	-1.87751
H	-3.72213	-4.88698	-3.04616
H	-4.68794	-3.75674	-2.05211
C	-2.13846	-6.56598	-0.77006
H	-1.57641	-6.84907	0.13336
H	-1.47321	-6.68390	-1.64284
H	-2.97323	-7.27887	-0.88725
C	-0.52157	-3.52027	-1.59069
C	-0.55795	-2.80457	-2.83941
C	0.60438	-2.70913	-3.63413
H	0.55671	-2.15606	-4.58049
C	1.80374	-3.33041	-3.26241
H	2.69249	-3.25608	-3.89853
C	1.83223	-4.07569	-2.07573
H	2.75952	-4.58172	-1.78418
C	0.70360	-4.18877	-1.24047
C	-1.84439	-2.17382	-3.37182
H	-2.63861	-2.42504	-2.65014
C	-2.23569	-2.76774	-4.74454
H	-1.50225	-2.50366	-5.52756
H	-3.21802	-2.38105	-5.07005
H	-2.29775	-3.86825	-4.70610
C	-1.75106	-0.63503	-3.45385
H	-1.57794	-0.20019	-2.45318
H	-2.68589	-0.20196	-3.85454
H	-0.93126	-0.31546	-4.12347
C	0.82919	-5.00015	0.04752
H	-0.18962	-5.09289	0.45642
C	1.39871	-6.41582	-0.18505
H	0.81907	-6.97207	-0.93920
H	1.38058	-6.99557	0.75502
H	2.44937	-6.38278	-0.52663
C	1.67346	-4.24909	1.09658
H	1.70775	-4.80680	2.05035

H	1.24633	-3.25376	1.30064
H	2.71099	-4.10532	0.74648
K	1.55738	-0.56532	-1.04652
Si	3.63812	5.52981	-0.90482
Si	0.81433	6.49533	-0.05728
Al	1.36060	3.25887	0.04170
O	2.49971	6.58215	-0.21835
N	2.91994	3.90596	-0.93358
N	0.42308	4.88747	0.57200
C	3.52467	2.86901	-1.71394
C	4.56388	2.04139	-1.17016
C	5.06083	0.96175	-1.92688
H	5.85110	0.33451	-1.49958
C	4.56769	0.66744	-3.20478
H	4.97342	-0.17270	-3.77924
C	3.55980	1.47721	-3.74437
H	3.17930	1.26307	-4.75044
C	3.03527	2.57525	-3.03266
C	5.13539	2.28675	0.22720
H	4.74350	3.25957	0.56164
C	6.67770	2.36122	0.22911
H	7.05178	3.09319	-0.50660
H	7.04800	2.65777	1.22626
H	7.13631	1.38534	-0.01350
C	4.64325	1.23058	1.24187
H	4.94571	0.21196	0.94033
H	5.05969	1.42909	2.24622
H	3.54087	1.24992	1.32044
C	1.94307	3.42094	-3.68924
H	1.79903	4.30156	-3.04027
C	2.33989	3.91667	-5.09688
H	2.44136	3.08192	-5.81351
H	1.56631	4.59635	-5.49622
H	3.29760	4.46204	-5.07886
C	0.59802	2.66289	-3.74365
H	0.26526	2.37399	-2.72922
H	-0.19161	3.28904	-4.19656
H	0.68550	1.74242	-4.35019
C	4.10051	6.13675	-2.64540
H	4.62547	7.10526	-2.57895
H	4.77265	5.41999	-3.14791
H	3.21011	6.27452	-3.27957
C	5.17033	5.76709	0.19603
H	4.97498	5.46676	1.23876
H	6.04934	5.21079	-0.16865
H	5.42838	6.84086	0.19910
C	0.29792	7.86992	1.14052
H	0.47871	8.85884	0.68590
H	-0.77302	7.80065	1.39575
H	0.87773	7.81431	2.07594
C	0.08423	6.86200	-1.77628
H	0.29271	6.04177	-2.48405
H	-1.00772	7.01283	-1.74219
H	0.53977	7.78299	-2.17992
C	-0.60281	4.69234	1.53520
C	-0.27795	4.60213	2.93431
C	-1.28901	4.29188	3.86705
H	-1.03006	4.22605	4.93052
C	-2.61990	4.09080	3.47225
H	-3.39251	3.86596	4.21494
C	-2.94745	4.20399	2.11319
H	-3.98930	4.06789	1.79834
C	-1.97073	4.49876	1.13904
C	1.14250	4.85775	3.44225
H	1.72256	5.22778	2.57920
C	1.18047	5.93685	4.54717
H	0.67370	5.60037	5.46969
H	2.22503	6.17420	4.81460

H	0.69062	6.86874	4.21902
C	1.82014	3.55819	3.93331
H	1.89134	2.81612	3.11623
H	2.84379	3.76166	4.29509
H	1.25419	3.10226	4.76714
C	-2.40213	4.62427	-0.32170
H	-1.48841	4.83265	-0.90140
C	-3.38339	5.80211	-0.51865
H	-2.95548	6.75008	-0.15091
H	-3.63115	5.92945	-1.58755
H	-4.33059	5.63423	0.02534
C	-3.00733	3.31523	-0.87203
H	-3.29676	3.43926	-1.93093
H	-2.28417	2.48186	-0.82092
H	-3.91303	3.01764	-0.31281
C	-8.46691	-1.18180	-3.37524
H	-8.49831	-2.28726	-3.33949
H	-9.35850	-0.85138	-3.94073
C	-7.18028	-0.70693	-4.09202
H	-7.14414	-1.12875	-5.11357
C	-8.50314	-0.60010	-1.94181
H	-9.41715	-0.94255	-1.42205
C	-7.26854	-1.09728	-1.15268
H	-7.25926	-2.20015	-1.08536
H	-7.27930	-0.70405	-0.11995
C	-5.93990	-1.20205	-3.31011
H	-5.00791	-0.88099	-3.80972
H	-5.92413	-2.30563	-3.25890
C	-5.97155	-0.62028	-1.86787
C	-5.95986	0.93218	-1.94327
H	-5.95863	1.34579	-0.91909
H	-5.02733	1.26480	-2.43378
C	-7.16562	0.83864	-4.16238
H	-8.03782	1.19955	-4.73939
H	-6.26006	1.18822	-4.69372
C	-8.48664	0.94600	-2.00918
H	-9.37971	1.31039	-2.55101
H	-8.53221	1.37293	-0.98915
C	-7.20100	1.41979	-2.72887
H	-7.18201	2.52456	-2.77214
C	-3.91995	-1.56473	-0.52815
N	-4.82752	-1.09017	-1.13752
C	4.15925	-2.04292	-0.45137
N	5.17111	-2.64217	-0.29467
C	6.39710	-3.36854	-0.09076
C	7.58818	-2.37160	-0.04838
C	6.60335	-4.37460	-1.25693
C	6.31873	-4.14168	1.25517
H	7.62549	-1.80813	-0.99870
H	7.42314	-1.64150	0.76466
C	8.90260	-3.15768	0.17452
H	6.63213	-3.82219	-2.21382
H	5.74181	-5.06579	-1.29811
C	7.92190	-5.15284	-1.03029
H	6.14693	-3.42332	2.07728
H	5.45392	-4.82934	1.23011
C	7.63790	-4.92142	1.47162
H	9.74329	-2.44031	0.20359
C	9.10755	-4.15874	-0.98782
C	8.82325	-3.92720	1.51493
H	8.06017	-5.86536	-1.86427
C	7.84256	-5.92309	0.30974
H	7.57245	-5.46829	2.43022
H	10.05687	-4.70923	-0.84955
H	9.18659	-3.61663	-1.94930
H	8.69704	-3.21854	2.35531
H	9.76838	-4.47343	1.69320
H	7.01057	-6.65212	0.28280
H	8.77216	-6.50102	0.46854

E

SCF (BP86) Energy = -4084.54520627
Enthalpy 0K = -4082.506943
Enthalpy 298K = -4082.379040
Free Energy 298K = -4082.687366
Lowest Frequency = 4.7482 cm^{-1}
Second Frequency = 6.2239 cm^{-1}
SCF (BP86-D3BJ) Energy = -4085.20765592
SCF (tol) Energy = -4084.56011632
SCF (BS2) Energy = -6852.16204623

K	1.91026	2.14452	-0.96144
Si	4.81965	-1.73492	-3.99179
Si	3.34831	-4.27002	-2.96704
Al	2.59781	-1.40477	-1.66407
O	4.68744	-3.27821	-3.29132
N	3.91111	-0.63316	-2.95040
N	2.09171	-3.19695	-2.34188
C	4.16064	0.76650	-2.93262
C	5.04355	1.35327	-1.95321
C	5.19448	2.75354	-1.87673
H	5.86928	3.17209	-1.12045
C	4.52154	3.62002	-2.74661
H	4.66148	4.70448	-2.67932
C	3.68637	3.06350	-3.72327
H	3.17097	3.72734	-4.42750
C	3.49436	1.67010	-3.83807
C	5.86097	0.50714	-0.97663
H	5.65644	-0.54896	-1.22141
C	7.37904	0.75918	-1.12700
H	7.71487	0.60617	-2.16475
H	7.95074	0.07129	-0.47809
H	7.65125	1.78938	-0.83414
C	5.42839	0.75721	0.48504
H	5.54418	1.82070	0.76083
H	6.04801	0.16593	1.18371
H	4.37274	0.47636	0.64574
C	2.57730	1.18039	-4.95746
H	2.51195	0.08375	-4.86255
C	3.15986	1.53005	-6.34732
H	3.16022	2.62229	-6.51544
H	2.55829	1.06988	-7.15076
H	4.20037	1.18179	-6.45361
C	1.15269	1.76204	-4.83118
H	0.67214	1.46527	-3.88278
H	0.50793	1.40222	-5.65128
H	1.15884	2.86572	-4.87639
C	4.20524	-1.88802	-5.78870
H	4.71602	-2.74191	-6.26757
H	4.43115	-0.98806	-6.38353
H	3.11859	-2.06670	-5.84479
C	6.67659	-1.34592	-4.07051
H	7.16238	-1.52894	-3.09887
H	6.85403	-0.29502	-4.35638
H	7.16505	-1.98904	-4.82269
C	4.03859	-5.58120	-1.77142
H	4.87058	-6.11087	-2.26796
H	3.27975	-6.33140	-1.49317
H	4.43077	-5.12080	-0.85113
C	2.82077	-5.21948	-4.52823
H	2.62439	-4.55016	-5.38007
H	1.90791	-5.80988	-4.33688
H	3.61989	-5.92183	-4.82241
C	0.74817	-3.64834	-2.20588

C	0.29149	-4.28038	-0.99599
C	-1.06140	-4.65576	-0.86186
H	-1.39210	-5.12769	0.07017
C	-1.99273	-4.43684	-1.88545
H	-3.03936	-4.73490	-1.76390
C	-1.55473	-3.83583	-3.07328
H	-2.27317	-3.66828	-3.88409
C	-0.21193	-3.44683	-3.25844
C	1.23473	-4.55683	0.17527
H	2.25540	-4.35959	-0.18906
C	1.16505	-6.02528	0.64909
H	0.19015	-6.26349	1.11121
H	1.94229	-6.22488	1.40843
H	1.31666	-6.72914	-0.18634
C	0.96585	-3.59256	1.35303
H	1.16334	-2.54611	1.05573
H	1.61807	-3.82739	2.21506
H	-0.08115	-3.66212	1.69890
C	0.17066	-2.79124	-4.58329
H	1.26697	-2.66662	-4.56577
C	-0.21395	-3.65252	-5.80673
H	0.20665	-4.66868	-5.74055
H	0.16016	-3.18696	-6.73588
H	-1.30984	-3.74802	-5.90908
C	-0.45974	-1.38777	-4.70300
H	-0.17214	-0.90712	-5.65445
H	-0.12611	-0.73279	-3.87955
H	-1.56357	-1.44025	-4.67572
K	-1.42219	-1.32610	-0.69584
Si	-6.74931	2.58393	-1.43311
Si	-4.77332	4.65660	-0.28044
Al	-3.72905	1.52590	-0.71633
O	-6.26789	4.11244	-0.86390
N	-5.67254	1.35316	-0.75259
N	-3.51319	3.47851	-0.71297
C	-6.24921	0.13407	-0.27697
C	-6.45391	-0.98948	-1.14325
C	-7.00653	-2.17844	-0.62594
H	-7.16680	-3.02811	-1.30123
C	-7.37428	-2.29231	0.71930
H	-7.83142	-3.21531	1.09467
C	-7.18011	-1.19522	1.57065
H	-7.49094	-1.26814	2.61990
C	-6.62546	0.01044	1.10316
C	-6.12222	-0.92717	-2.63401
H	-5.68349	0.06767	-2.82069
C	-7.39246	-1.06868	-3.50400
H	-8.14897	-0.30977	-3.24177
H	-7.14964	-0.95466	-4.57586
H	-7.86151	-2.06051	-3.37206
C	-5.07323	-1.98244	-3.04433
H	-5.44201	-3.01095	-2.87795
H	-4.82207	-1.88637	-4.11626
H	-4.14367	-1.86045	-2.46293
C	-6.42342	1.17515	2.07184
H	-6.33900	2.08339	1.45052
C	-7.59650	1.37848	3.05237
H	-7.67862	0.55360	3.78357
H	-7.45251	2.30837	3.63016
H	-8.56105	1.45189	2.52183
C	-5.09111	1.02678	2.84122
H	-4.23516	0.96004	2.14352
H	-4.92240	1.88707	3.51511
H	-5.09150	0.10864	3.45635
C	-8.55097	2.32907	-0.89732
H	-9.18774	3.10974	-1.34778
H	-8.93249	1.34551	-1.21858
H	-8.65566	2.39253	0.19791
C	-6.68070	2.69598	-3.33088
H	-5.63865	2.76358	-3.68549
H	-7.14551	1.81859	-3.81077
H	-7.21706	3.59735	-3.67491
C	-4.44809	6.36642	-1.04140
H	-5.22521	7.07221	-0.70034
H	-3.46591	6.76656	-0.73847
H	-4.48085	6.33330	-2.14215
C	-5.00930	4.90224	1.59259
H	-5.12091	3.93928	2.11721
H	-4.16338	5.44428	2.04774
H	-5.92449	5.49397	1.76911
C	-2.21181	3.98754	-0.99015
C	-1.83696	4.32187	-2.33790
C	-0.54415	4.80803	-2.60572
H	-0.27522	5.07080	-3.63506
C	0.39846	4.99314	-1.58263
H	1.38776	5.41055	-1.80652
C	0.04067	4.66591	-0.26612
H	0.76767	4.81820	0.54171
C	-1.23771	4.15905	0.05008
C	-2.82831	4.14381	-3.48718
H	-3.83065	4.10864	-3.02700
C	-2.80866	5.30176	-4.50598
H	-1.86974	5.33004	-5.08821
H	-3.63248	5.18175	-5.23095
H	-2.92750	6.28111	-4.01207
C	-2.60895	2.78947	-4.19875
H	-2.69125	1.94963	-3.48330
H	-3.35732	2.63788	-4.99777
H	-1.60542	2.74346	-4.65813
C	-1.54742	3.80879	1.50571
H	-2.61646	3.54199	1.54410
C	-1.30902	4.99823	2.46153
H	-1.86081	5.89661	2.13738
H	-1.63651	4.74511	3.48556
H	-0.23828	5.26960	2.51391
C	-0.74927	2.57100	1.97448
H	-1.01074	2.30417	3.01460
H	-0.97776	1.69894	1.33530
H	0.34099	2.76014	1.94361
C	7.07287	-4.84657	3.39421
H	7.45162	-5.47503	2.56611
H	7.57783	-5.18943	4.31695
C	5.54125	-5.01469	3.53461
H	5.29634	-6.07831	3.71185
C	7.40500	-3.35864	3.12994
H	8.49810	-3.23332	3.01928
C	6.71866	-2.90034	1.82096
H	7.06832	-3.49892	0.96053
H	6.95577	-1.84299	1.60494
C	4.84955	-4.55937	2.22742
H	3.75425	-4.68332	2.30018
H	5.19357	-5.16575	1.37005
C	5.17773	-3.06275	1.96093
C	4.66828	-2.20366	3.15294
H	4.88198	-1.13879	2.94989
H	3.57114	-2.31057	3.23248
C	5.03162	-4.15580	4.71666
H	5.50513	-4.49011	5.65905
H	3.93960	-4.28586	4.84010
C	6.89615	-2.49663	4.31043
H	7.40104	-2.80179	5.24639
H	7.14683	-1.43199	4.14090
C	5.36458	-2.66738	4.45487
H	4.99689	-2.04889	5.29495
C	4.00670	-2.28039	-0.25642
N	4.54029	-2.64010	0.74600

```
C   3.09040  3.59795  1.42246
N   3.51321  4.25841  2.31116
C   4.01175  5.07127  3.38930
C   5.47382  4.65866  3.71579
C   3.11939  4.86490  4.64479
C   3.97489  6.56571  2.96388
H   5.49713  3.58863  3.99159
H   6.09799  4.78255  2.81212
C   6.00212  5.53834  4.87460
H   3.12928  3.79613  4.92650
H   2.07606  5.13274  4.39820
C   3.65753  5.74415  5.79928
H   4.58979  6.70086  2.05539
H   2.93709  6.84557  2.70723
C   4.50960  7.43711  4.12573
H   7.04204  5.23845  5.10048
C   5.11365  5.33308  6.12515
C   5.96597  7.02644  4.45074
H   3.01735  5.59154  6.68754
C   3.62053  7.23231  5.37585
H   4.47991  8.49747  3.81464
H   5.49868  5.94039  6.96550
H   5.14874  4.27555  6.44942
H   6.61470  7.18848  3.56904
H   6.36464  7.66047  5.26455
H   2.58064  7.54241  5.16018
H   3.98153  7.86979  6.20442
C  -2.64024 -2.65029  1.70359
N  -3.24219 -3.24896  2.53048
C  -3.98941 -3.97013  3.52528
C  -3.00963 -4.78314  4.41575
C  -4.98328 -4.93678  2.82325
C  -4.78094 -2.96387  4.40481
H  -2.43146 -5.47927  3.78080
H  -2.29007 -4.09145  4.89111
C  -3.81774 -5.55675  5.48501
H  -4.41775 -5.63437  2.17836
H  -5.65559 -4.34936  2.17216
C  -5.78207 -5.71091  3.89971
H  -4.07276 -2.25990  4.87907
H  -5.45353 -2.37501  3.75582
C  -5.58074 -3.74421  5.47518
H  -3.11388 -6.13290  6.11407
C  -4.80595 -6.52150  4.78625
C  -4.60501 -4.55393  6.36270
H  -6.48529 -6.39756  3.39322
C  -6.56958 -4.70919  4.77820
H  -6.13966 -3.02079  6.09706
H  -5.37117 -7.09637  5.54381
H  -4.25165 -7.25614  4.17144
H  -3.90590 -3.87138  6.88266
H  -5.16689 -5.09761  7.14538
H  -7.28480 -4.13816  4.15705
H  -7.16274 -5.25549  5.53563
```

TS(E-F)

SCF (BP86) Energy = -4084.51981865
Enthalpy 0K = -4082.482133
Enthalpy 298K = -4082.355533
Free Energy 298K = -4082.658789
Lowest Frequency = -58.1460 cm^{-1}
Second Frequency = 5.0524 cm^{-1}
SCF (BP86-D3BJ) Energy = -4085.19432419
SCF (tol) Energy = -4084.53403873
SCF (BS2) Energy = -6852.13943289

```
K   1.62862  1.35067 -1.93175
Si  5.54210 -2.76000 -2.50092
Si  4.48659 -4.65585 -0.27565
Al  2.99649 -1.82900 -0.74693
O   5.62390 -3.68110 -1.07492
N   4.29232 -1.53918 -2.22908
N   2.96047 -3.76621 -0.34750
C   4.23945 -0.32982 -2.97532
C   4.85771  0.87716 -2.48129
C   4.70482  2.09384 -3.17891
H   5.18375  2.99616 -2.78037
C   3.97233  2.17802 -4.36930
H   3.87442  3.12994 -4.90252
C   3.39198  1.00867 -4.87628
H   2.83743  1.05357 -5.82114
C   3.51118 -0.23294 -4.21644
C   5.70072  0.90300 -1.20566
H   5.75621 -0.13592 -0.83864
C   7.13791  1.40691 -1.47148
H   7.63264  0.82231 -2.26295
H   7.75008  1.32765 -0.55507
H   7.14694  2.46747 -1.78147
C   5.03454  1.76668 -0.11128
H   4.87535  2.80321 -0.45908
H   5.66951  1.81568  0.79185
H   4.05612  1.35113  0.18848
C   2.86606 -1.44623 -4.88394
H   3.01394 -2.30232 -4.20466
C   3.54355 -1.75797 -6.23952
H   3.34335 -0.95932 -6.97656
H   3.16013 -2.70382 -6.66078
H   4.63779 -1.84442 -6.13913
C   1.34942 -1.25209 -5.09606
H   0.82264 -1.08274 -4.14084
H   0.90296 -2.14271 -5.57062
H   1.14002 -0.38975 -5.75387
C   5.22252 -3.97374 -3.93343
H   5.95010 -4.80177 -3.86840
H   5.34928 -3.49553 -4.91830
H   4.20926 -4.40680 -3.89547
C   7.27529 -2.03168 -2.76404
H   7.64977 -1.55167 -1.84595
H   7.27662 -1.28284 -3.57449
H   7.98130 -2.83296 -3.04227
C   5.24802 -4.91909  1.44824
H   6.23602 -5.39781  1.33053
H   4.62832 -5.57632  2.08053
H   5.39474 -3.96313  1.97475
C   4.39738 -6.37868 -1.07407
H   4.18923 -6.33209 -2.15415
H   3.61237 -6.99108 -0.59747
H   5.36025 -6.89992 -0.93405
C   1.72936 -4.38770  0.00660
C   1.24267 -4.36308  1.36150
C  -0.01161 -4.92885  1.67294
H  -0.36465 -4.90310  2.71071
C  -0.80891 -5.53790  0.69495
H  -1.77259 -5.98398  0.95954
C  -0.34036 -5.57552 -0.62530
H  -0.95161 -6.05893 -1.39679
C   0.90587 -5.02358 -0.98820
C   2.04715 -3.73497  2.49944
H   3.03977 -3.49317  2.08772
C   2.23575 -4.70699  3.68546
H   1.28172 -4.91745  4.20102
H   2.92211 -4.27471  4.43526
H   2.65522 -5.67233  3.35659
C   1.40435 -2.41485  2.98100
H   1.38168 -1.66373  2.17026
```

H	1.97243	-1.98123	3.82476
H	0.37010	-2.57791	3.33442
C	1.32999	-5.10901	-2.45298
H	2.36316	-4.72540	-2.50345
C	1.30825	-6.55551	-2.99514
H	1.91187	-7.23462	-2.37229
H	1.71060	-6.58543	-4.02325
H	0.28108	-6.96015	-3.03577
C	0.44892	-4.19985	-3.33577
H	0.76368	-4.25367	-4.39274
H	0.52273	-3.14520	-3.01767
H	-0.61315	-4.50371	-3.28974
K	-0.93506	-2.20175	-0.07108
Si	-7.12824	-0.63135	-1.81994
Si	-5.68783	1.99592	-2.47203
Al	-4.14464	-0.33789	-0.71382
O	-6.95931	0.87878	-2.58703
N	-6.03779	-0.67348	-0.41852
N	-4.19804	1.13689	-1.99586
C	-6.53076	-1.29240	0.77915
C	-6.29601	-2.67704	1.05925
C	-6.77366	-3.23961	2.26035
H	-6.58179	-4.30058	2.46303
C	-7.49330	-2.48284	3.18945
H	-7.86165	-2.93864	4.11510
C	-7.74851	-1.13411	2.90872
H	-8.32583	-0.53792	3.62497
C	-7.28905	-0.52503	1.72667
C	-5.53496	-3.58026	0.09087
H	-5.29202	-2.96936	-0.79662
C	-6.37991	-4.78604	-0.37579
H	-7.32693	-4.45879	-0.83752
H	-5.82663	-5.38807	-1.11912
H	-6.63700	-5.45305	0.46667
C	-4.19839	-4.05322	0.70120
H	-4.36375	-4.71944	1.56798
H	-3.60420	-4.61394	-0.04368
H	-3.60937	-3.18875	1.05612
C	-7.58568	0.95652	1.49322
H	-7.42049	1.14909	0.41997
C	-9.03616	1.35768	1.83449
H	-9.23640	1.30376	2.91987
H	-9.22725	2.39954	1.52205
H	-9.77023	0.70792	1.32903
C	-6.58902	1.84219	2.27357
H	-5.54774	1.58945	2.01126
H	-6.75733	2.91432	2.06273
H	-6.70877	1.69321	3.36281
C	-8.95210	-0.84318	-1.35220
H	-9.55281	-0.93114	-2.27396
H	-9.11079	-1.75284	-0.74997
H	-9.32998	0.01865	-0.78086
C	-6.70688	-1.94825	-3.12777
H	-5.63566	-1.93025	-3.38865
H	-6.94730	-2.96140	-2.76217
H	-7.28722	-1.77261	-4.05028
C	-5.52611	2.81632	-4.17654
H	-6.45810	3.36377	-4.40083
H	-4.69074	3.53547	-4.20805
H	-5.37137	2.07312	-4.97461
C	-6.21658	3.33909	-1.23665
H	-6.18373	2.97060	-0.19980
H	-5.56311	4.22533	-1.30535
H	-7.24911	3.66194	-1.45583
C	-2.99471	1.56268	-2.63105
C	-2.56402	0.96338	-3.86601
C	-1.38518	1.41442	-4.48819
H	-1.07753	0.96301	-5.43762

C	-0.61375	2.45112	-3.94170
H	0.27414	2.82049	-4.47041
C	-1.02023	3.02956	-2.72964
H	-0.43058	3.84910	-2.29876
C	-2.18636	2.60246	-2.06097
C	-3.37032	-0.15823	-4.52076
H	-4.38874	-0.09798	-4.10017
C	-3.47762	-0.03042	-6.05467
H	-2.51035	-0.21068	-6.55761
H	-4.18839	-0.77835	-6.44664
H	-3.83213	0.96944	-6.35730
C	-2.80068	-1.54283	-4.13496
H	-2.83354	-1.67906	-3.03576
H	-3.38849	-2.35578	-4.59884
H	-1.75157	-1.64596	-4.46681
C	-2.56171	3.27909	-0.74293
H	-3.54157	2.86990	-0.44564
C	-2.70165	4.81047	-0.88974
H	-3.40734	5.07957	-1.69359
H	-3.06699	5.25820	0.05146
H	-1.73226	5.28585	-1.12940
C	-1.55331	2.93285	0.37565
H	-1.84482	3.41587	1.32561
H	-1.51507	1.84501	0.55549
H	-0.53662	3.28816	0.12053
C	7.38105	-0.90966	5.35658
H	7.96341	-1.77955	4.99837
H	7.83530	-0.58863	6.31278
C	5.90376	-1.31125	5.58068
H	5.84788	-2.14124	6.30900
C	7.45061	0.24082	4.32433
H	8.50498	0.52656	4.15287
C	6.84160	-0.23189	2.98273
H	7.39399	-1.10088	2.58191
H	6.89474	0.57068	2.22510
C	5.28863	-1.78680	4.24269
H	4.23556	-2.09039	4.38206
H	5.83554	-2.66276	3.84946
C	5.35450	-0.63181	3.20339
C	4.55870	0.59098	3.74169
H	4.58688	1.40185	2.99158
H	3.50038	0.30287	3.87734
C	5.10986	-0.09377	6.11174
H	5.52993	0.23869	7.07961
H	4.05620	-0.37615	6.29739
C	6.65662	1.46018	4.85216
H	7.10136	1.81952	5.79918
H	6.71712	2.29644	4.12961
C	5.17963	1.05776	5.08038
H	4.60777	1.92812	5.45259
C	4.33396	-1.44283	0.92302
N	4.79075	-1.07332	1.95816
C	2.06648	4.17452	-0.98345
N	2.19904	5.31358	-0.68552
C	2.34429	6.70041	-0.32720
C	3.72979	6.91869	0.34175
C	1.21721	7.09582	0.66698
C	2.24061	7.57435	-1.60826
H	3.80752	6.27463	1.23662
H	4.52623	6.61094	-0.36014
C	3.87846	8.41159	0.72260
H	1.28114	6.45342	1.56403
H	0.23444	6.91068	0.19700
C	1.37675	8.58811	1.04531
H	3.02823	7.26996	-2.32155
H	1.26440	7.39401	-2.09382
C	2.39581	9.06429	-1.21908
H	4.86594	8.55830	1.19764

```
C   2.75612  8.80619  1.71260
C   3.77571  9.28225 -0.55275
H   0.57143  8.86089  1.75167
C   1.27284  9.45875 -0.22990
H   2.32097  9.67815 -2.13536
H   2.86950  9.86576  2.00852
H   2.83279  8.20350  2.63752
H   4.58678  9.02240 -1.25932
H   3.90513 10.34934 -0.29264
H   0.28196  9.32566 -0.70367
H   1.36195 10.52875  0.03516
C  -2.60572 -0.59191  1.51380
N  -3.28105  0.00858  2.35356
C  -3.08158  0.19967  3.80510
C  -1.73533 -0.38570  4.30538
C  -4.24398 -0.50290  4.55346
C  -3.11559  1.71766  4.11850
H  -1.70304 -1.46558  4.06758
H  -0.90173  0.09179  3.75635
C  -1.59050 -0.15911  5.82927
H  -4.22522 -1.58248  4.31670
H  -5.20843 -0.11656  4.18113
C  -4.10226 -0.27369  6.07736
H  -2.29292  2.21763  3.57298
H  -4.06350  2.14480  3.74612
C  -2.97441  1.94608  5.64278
H  -0.62572 -0.58248  6.17027
C  -2.75660 -0.85982  6.56731
C  -1.62878  1.35826  6.13076
H  -4.93961 -0.77768  6.59569
C  -4.14131  1.24388  6.37797
H  -3.00376  3.03300  5.84960
H  -2.65339 -0.71903  7.66073
H  -2.72567 -1.94989  6.37732
H  -0.78571  1.86802  5.62490
H  -1.50789  1.53341  7.21735
H  -5.10799  1.67128  6.05019
H  -4.06309  1.41891  7.46844
```

F

SCF (BP86) Energy = -4084.54901522
Enthalpy 0K = -4082.509629
Enthalpy 298K = -4082.383383
Free Energy 298K = -4082.684081
Lowest Frequency = 3.2482 cm^{-1}
Second Frequency = 5.8851 cm^{-1}
SCF (BP86-D3BJ) Energy = -4085.24473313
SCF (tol) Energy = -4084.56081159
SCF (BS2) Energy = -6852.17032528

```
K  -0.00040  0.50262 -1.41383
Si  5.78884 -2.31320 -2.23870
Si  5.41377 -3.76123  0.48476
Al  3.15083 -1.65242 -0.51697
O   6.19040 -2.76749 -0.64713
N   4.24784 -1.45504 -2.14573
N   3.69456 -3.36571  0.37490
C   3.85991 -0.53253 -3.16836
C   4.12369  0.87603 -3.03333
C   3.67341  1.78175 -4.01448
H   3.88725  2.85019 -3.88691
C   2.98438  1.35420 -5.15406
H   2.65717  2.07151 -5.91500
C   2.74854 -0.01526 -5.31188
H   2.23044 -0.36846 -6.21186
C   3.16801 -0.96050 -4.35400
C   4.90643  1.45297 -1.85378
H   5.25361  0.59707 -1.25003
C   6.14471  2.25819 -2.31021
H   6.79901  1.65996 -2.96393
H   6.73946  2.58260 -1.43670
H   5.85742  3.16848 -2.86696
C   4.00313  2.33455 -0.96454
H   3.56735  3.16739 -1.54539
H   4.57835  2.77560 -0.12939
H   3.17122  1.74852 -0.53667
C   2.87291 -2.43035 -4.64720
H   3.25017 -3.01193 -3.78944
C   3.59046 -2.90680 -5.93197
H   3.17425 -2.41073 -6.82740
H   3.47025 -3.99612 -6.06980
H   4.66891 -2.68013 -5.90559
C   1.35700 -2.69174 -4.77500
H   0.82857 -2.40090 -3.85109
H   1.15145 -3.75956 -4.96559
H   0.92145 -2.11666 -5.61230
C   5.80545 -3.91232 -3.27610
H   6.74370 -4.45762 -3.07179
H   5.76815 -3.70625 -4.35797
H   4.96284 -4.58014 -3.03080
C   7.22328 -1.23446 -2.85790
H   7.48173 -0.45947 -2.11880
H   6.96620 -0.73650 -3.80819
H   8.11911 -1.85562 -3.02961
C   6.29888 -3.34951  2.11937
H   7.37804 -3.54661  1.99345
H   5.93960 -3.96668  2.95937
H   6.17773 -2.28792  2.38590
C   5.77769 -5.59726  0.14526
H   5.52413 -5.89102 -0.88508
H   5.21024 -6.24341  0.83766
H   6.85151 -5.79850  0.30279
C   2.70243 -4.18401  0.97415
C   2.28146 -3.98255  2.33865
C   1.22033 -4.74337  2.87472
H   0.91873 -4.57529  3.91582
C   0.54985 -5.71340  2.11629
H  -0.26327 -6.30247  2.55414
C   0.96664 -5.93594  0.79620
H   0.46667 -6.70866  0.19965
C   2.02831 -5.20965  0.21781
C   2.94513 -2.94506  3.24455
H   3.79988 -2.53674  2.68233
C   3.47197 -3.57269  4.55423
H   2.64816 -3.92587  5.20024
H   4.04912 -2.83158  5.13520
H   4.12860 -4.43551  4.35380
C   1.98919 -1.77120  3.55697
H   1.71779 -1.22310  2.63634
H   2.46145 -1.04679  4.24535
H   1.06210 -2.12504  4.04559
C   2.42561 -5.53891 -1.21925
H   3.31555 -4.92680 -1.44439
C   2.78596 -7.03049 -1.40054
H   3.56625 -7.35090 -0.69188
H   3.15748 -7.21314 -2.42441
H   1.90687 -7.68293 -1.25082
C   1.31735 -5.13526 -2.21343
H   1.61564 -5.37133 -3.24963
H   1.11645 -4.05089 -2.16221
H   0.37472 -5.67549 -2.00704
K  -0.20062 -2.70607  0.73442
Si -6.75305 -2.37869 -0.44550
Si -6.32645 -0.04603 -2.46648
Al -4.18217 -0.57197 -0.13259
```

O	-7.03736	-1.45892	-1.84541
N	-5.67691	-1.44520	0.61565
N	-4.66136	0.08230	-1.84841
C	-5.89619	-1.47726	2.03672
C	-5.25452	-2.45347	2.86234
C	-5.45834	-2.43079	4.25678
H	-4.95982	-3.18273	4.88068
C	-6.28556	-1.47866	4.86014
H	-6.43030	-1.47442	5.94591
C	-6.94103	-0.54215	4.05174
H	-7.60604	0.19470	4.51662
C	-6.77553	-0.52753	2.65378
C	-4.35764	-3.54502	2.27804
H	-4.32214	-3.38219	1.18699
C	-4.93286	-4.95550	2.53812
H	-5.96105	-5.04955	2.14993
H	-4.31155	-5.73015	2.05305
H	-4.96727	-5.18304	3.61857
C	-2.91344	-3.44348	2.81600
H	-2.87726	-3.58134	3.91156
H	-2.27481	-4.23171	2.37182
H	-2.48632	-2.45018	2.58892
C	-7.53505	0.51375	1.83114
H	-7.49333	0.17788	0.78150
C	-9.02043	0.63819	2.23336
H	-9.14148	1.08271	3.23741
H	-9.55408	1.29330	1.52250
H	-9.52445	-0.34277	2.24135
C	-6.84637	1.89555	1.89798
H	-5.81130	1.84004	1.51964
H	-7.39412	2.64214	1.29443
H	-6.80863	2.26661	2.93842
C	-8.41262	-2.75496	0.38267
H	-9.00532	-3.41620	-0.27225
H	-8.27359	-3.26479	1.35041
H	-8.99557	-1.83747	0.55822
C	-5.99616	-4.01406	-1.05250
H	-4.98075	-3.86422	-1.45622
H	-5.93154	-4.75555	-0.23836
H	-6.62025	-4.44312	-1.85536
C	-6.34433	-0.22010	-4.35372
H	-7.38077	-0.13128	-4.72164
H	-5.73586	0.55186	-4.85299
H	-5.96184	-1.20748	-4.65777
C	-7.45190	1.39822	-1.96313
H	-7.34527	1.64447	-0.89494
H	-7.22672	2.30709	-2.54534
H	-8.50555	1.12761	-2.14904
C	-3.58364	0.41305	-2.73206
C	-2.82041	-0.61717	-3.38059
C	-1.69495	-0.26650	-4.15536
H	-1.11438	-1.05887	-4.64034
C	-1.31930	1.07170	-4.34793
H	-0.44082	1.31928	-4.95408
C	-2.10627	2.08150	-3.77637
H	-1.84884	3.13088	-3.96138
C	-3.23338	1.78254	-2.98306
C	-3.22211	-2.09170	-3.30281
H	-4.21509	-2.12213	-2.82179
C	-3.35060	-2.73531	-4.70166
H	-2.37126	-2.82045	-5.20512
H	-3.76495	-3.75498	-4.61491
H	-4.01436	-2.15000	-5.35935
C	-2.25494	-2.91337	-2.42048
H	-2.22647	-2.47860	-1.40528
H	-2.58751	-3.96537	-2.35218
H	-1.23524	-2.91505	-2.84796
C	-4.10156	2.93377	-2.47447

H	-4.80602	2.50048	-1.74384
C	-4.91689	3.54784	-3.63840
H	-5.52820	2.79161	-4.15724
H	-5.59271	4.34001	-3.26898
H	-4.24539	4.00142	-4.38986
C	-3.30087	4.04523	-1.76557
H	-3.98815	4.80463	-1.35196
H	-2.69600	3.64443	-0.93765
H	-2.61911	4.56475	-2.46359
C	7.34209	1.92390	4.67864
H	8.12633	1.16956	4.47837
H	7.73291	2.59458	5.46691
C	6.04732	1.23148	5.16670
H	6.25899	0.64186	6.07783
C	7.04067	2.73374	3.39511
H	7.96471	3.22403	3.03657
C	6.52566	1.77975	2.29100
H	7.27908	1.00783	2.05165
H	6.31600	2.33564	1.35923
C	5.52881	0.27329	4.06737
H	4.61010	-0.24277	4.39971
H	6.28020	-0.50427	3.83965
C	5.22129	1.08390	2.77642
C	4.14257	2.15891	3.08969
H	3.90712	2.71647	2.16551
H	3.21412	1.65587	3.41688
C	4.97138	2.30065	5.47270
H	5.32678	2.97626	6.27344
H	4.04763	1.81790	5.84443
C	5.96381	3.80334	3.69772
H	6.33402	4.50521	4.46865
H	5.75535	4.40128	2.78999
C	4.66974	3.11140	4.18982
H	3.89572	3.87224	4.40210
C	4.37981	-0.52591	0.87944
N	4.74693	0.19857	1.75057
C	0.41940	3.44714	-0.99282
N	0.60083	4.61804	-0.98053
C	0.81422	6.04134	-0.99153
C	2.13508	6.37352	-0.24375
C	-0.37553	6.74834	-0.28510
C	0.91172	6.53153	-2.46320
H	2.06749	6.00106	0.79465
H	2.97344	5.84469	-0.73242
C	2.35917	7.90502	-0.26558
H	-0.45536	6.37655	0.75260
H	-1.31506	6.48567	-0.80389
C	-0.14126	8.27817	-0.30609
H	1.74192	6.00493	-2.96799
H	-0.02056	6.26900	-2.99557
C	1.14020	8.06205	-2.47497
H	3.30066	8.13245	0.26768
C	1.17451	8.60970	0.43831
C	2.45658	8.39231	-1.73124
H	-0.99165	8.77286	0.19818
C	-0.04505	8.76614	-1.77166
H	1.20808	8.40137	-3.52495
H	1.33777	9.70360	0.44840
H	1.10864	8.28397	1.49386
H	3.31344	7.90957	-2.23836
H	2.64070	9.48264	-1.75515
H	-0.98896	8.55259	-2.30803
H	0.09847	9.86256	-1.79618
C	-2.26323	-0.64368	0.45876
N	-2.97053	0.28988	1.09978
C	-2.45954	1.17499	2.21786
C	-0.96993	0.94587	2.53584
C	-3.29600	0.87034	3.48503

C -2.66278 2.65357 1.81820
H -0.80670 -0.11645 2.79936
H -0.35437 1.13800 1.63559
C -0.52741 1.87059 3.69563
H -3.16225 -0.19112 3.76503
H -4.36880 1.00097 3.26069
C -2.86417 1.79604 4.64645
H -2.05617 2.86876 0.91713
H -3.72219 2.82100 1.54772
C -2.23590 3.58631 2.97706
H 0.54524 1.69545 3.91118
C -1.36737 1.56228 4.95825
C -0.73901 3.34917 3.28954
H -3.47365 1.56510 5.54048
C -3.08073 3.27199 4.23486
H -2.39300 4.64054 2.67617
H -1.04313 2.20822 5.79743
H -1.20561 0.51409 5.27573
H -0.12415 3.58946 2.40022
H -0.40783 4.01910 4.10705
H -4.15237 3.45438 4.02769
H -2.79187 3.94691 5.06396

G

SCF (BP86) Energy = -2283.73993319
Enthalpy 0K = -2282.603044
Enthalpy 298K = -2282.535053
Free Energy 298K = -2282.709227
Lowest Frequency = 7.3255 cm^{-1}
Second Frequency = 8.0790 cm^{-1}
SCF (BP86-D3BJ) Energy = -
2284.09478616
SCF (tol) Energy = -2283.74921651
SCF (BS2) Energy = -3667.61187611

K -2.03970 -1.15189 0.52449
Si 3.70639 -2.78152 0.86733
Si 4.24543 -1.55615 -1.93409
Al 1.87651 -0.39100 -0.13234
O 4.35652 -2.72847 -0.70433
N 2.11417 -2.00103 0.84888
N 3.35096 -0.16240 -1.28784
N -6.06431 -0.54096 -0.13993
N 1.03217 1.18996 0.58625
C 0.93537 -2.67789 1.27891
C 0.19050 -3.52637 0.38617
C -0.99012 -4.15539 0.83703
H -1.53686 -4.81246 0.14972
C -1.46527 -3.98143 2.14563
H -2.37060 -4.49781 2.48418
C -0.74123 -3.15982 3.02137
H -1.09369 -3.03393 4.05173
C 0.44385 -2.50791 2.61892
C 0.62498 -3.75479 -1.06166
H 1.64786 -3.35387 -1.15352
C 0.66501 -5.24740 -1.45381
H -0.34393 -5.69768 -1.47801
H 1.27647 -5.83659 -0.75034
H 1.09911 -5.36235 -2.46194
C -0.27687 -2.96185 -2.03847
H -0.22763 -1.87729 -1.82730
H -1.32881 -3.30125 -1.96714
H 0.04149 -3.11439 -3.08498
C 1.20528 -1.66386 3.64005
H 1.92792 -1.05562 3.06862
C 2.00005 -2.56651 4.61361
H 2.69906 -3.22783 4.07604
H 1.31911 -3.20878 5.20131
H 2.58524 -1.95568 5.32379
C 0.28966 -0.71024 4.43604
H -0.40829 -1.25735 5.09573
H -0.30627 -0.06821 3.76495
H 0.89434 -0.05149 5.08316
C 3.58621 -4.61231 1.33859
H 4.59854 -5.04952 1.37367
H 2.99932 -5.17731 0.59695
H 3.11717 -4.75639 2.32606
C 4.90848 -1.88323 2.02598
H 4.93402 -0.80341 1.80615
H 5.93142 -2.28048 1.90993
H 4.60946 -2.00626 3.08079
C 3.40314 -2.42360 -3.40263
H 2.33972 -2.63901 -3.21304
H 3.91564 -3.37935 -3.60952
H 3.46372 -1.80350 -4.31298
C 5.99773 -1.11254 -2.50611
H 6.39923 -1.94306 -3.11187
H 6.67970 -0.95020 -1.65750
H 5.99723 -0.20388 -3.13102
C 3.59164 1.15122 -1.83298
C 2.77125 1.67660 -2.88223
C 2.98348 2.99519 -3.33298
H 2.34208 3.39305 -4.12851
C 3.99264 3.80086 -2.79805
H 4.13981 4.82409 -3.16082
C 4.82289 3.27434 -1.80154
H 5.62657 3.89517 -1.38912
C 4.65097 1.96540 -1.31176
C 1.68208 0.84882 -3.56914
H 1.69382 -0.15283 -3.10589
C 1.98099 0.67954 -5.07734
H 2.99003 0.26793 -5.24977
H 1.92522 1.64638 -5.60910
H 1.24699 0.00011 -5.54666
C 0.26990 1.44140 -3.36502
H 0.20206 2.46587 -3.77425
H -0.00376 1.47421 -2.29613
H -0.48359 0.82509 -3.88991
C 5.59356 1.46515 -0.21503
H 5.45475 0.37339 -0.14750
C 7.08091 1.74380 -0.52302
H 7.72464 1.25878 0.23228
H 7.31047 2.82401 -0.49676
H 7.37164 1.36453 -1.51628
C 5.21690 2.05547 1.16096
H 4.17966 1.79273 1.42547
H 5.29510 3.15786 1.15446
H 5.88500 1.67286 1.95392
C -4.95182 -0.86222 0.10895
C -7.41671 -0.15043 -0.44247
C -7.76699 -0.59535 -1.89000
H -7.05516 -0.13027 -2.59610
H -7.64972 -1.69128 -1.97306
C -8.38773 -0.83010 0.56209
H -8.11679 -0.53054 1.59098
H -8.27366 -1.92744 0.49413
C -7.54180 1.39456 -0.32680
H -6.82895 1.87128 -1.02382
H -7.26497 1.70680 0.69665
C -9.22012 -0.17064 -2.21201
H -9.46095 -0.49033 -3.24253
C -10.18848 -0.84795 -1.21257
H -11.23196 -0.56917 -1.45008
H -10.12132 -1.94898 -1.30147
C -9.83859 -0.40519 0.22865
H -10.52291 -0.89270 0.94721

C	-9.96489	1.13309	0.34464
H	-11.00474	1.44342	0.13147
H	-9.73719	1.45866	1.37744
C	-8.99631	1.80956	-0.65531
H	-9.07715	2.90873	-0.57017
C	-9.34595	1.36769	-2.09686
H	-10.37590	1.68191	-2.34872
H	-8.67211	1.86176	-2.82225
C	0.07892	0.46832	-0.00074
C	0.79158	2.52375	1.26405
C	1.40557	3.62272	0.36189
H	2.47374	3.40227	0.18381
H	0.90805	3.59644	-0.62552
C	1.49328	2.54291	2.63953
H	2.56553	2.30730	2.51279
H	1.06213	1.75336	3.28274
C	-0.70843	2.81701	1.46300
H	-1.22458	2.75677	0.48723
H	-1.15740	2.02529	2.09709
C	1.23149	5.01087	1.02202
H	1.67747	5.78498	0.36860
C	-0.27633	5.29733	1.21783
H	-0.78948	5.31203	0.23700
H	-0.42182	6.29578	1.67497
C	-0.89414	4.20395	2.12309
H	-1.97568	4.40402	2.25980
C	-0.18714	4.22012	3.49993
H	-0.63744	3.45923	4.16707
H	-0.32724	5.20329	3.99035
C	1.32072	3.93012	3.30618
H	1.83002	3.93131	4.28936
C	1.94001	5.01971	2.39790
H	1.83717	6.01406	2.87461
H	3.02322	4.83381	2.26728

TS(G-H)

SCF (BP86) Energy = -2283.71332952
Enthalpy 0K = -2282.577277
Enthalpy 298K = -2282.510246
Free Energy 298K = -2282.679901
Lowest Frequency = -39.3457 cm^{-1}
Second Frequency = 7.9820 cm^{-1}
SCF (BP86-D3BJ) Energy = -2284.07819768
SCF (tol) Energy = -2283.72258117
SCF (BS2) Energy = -3667.58704751

K	-2.28696	-1.33691	1.27266
Si	3.38006	-2.83323	0.24649
Si	3.36874	-1.28639	-2.44701
Al	1.36372	-0.39854	-0.16193
O	3.75937	-2.56104	-1.38592
N	1.80262	-2.08183	0.59539
N	2.63092	0.01114	-1.48216
N	-3.89015	0.46808	-0.17496
N	0.54484	1.09046	0.78960
C	0.76607	-2.81327	1.24579
C	-0.16176	-3.61888	0.49533
C	-1.21100	-4.28511	1.16564
H	-1.89975	-4.91079	0.58479
C	-1.37717	-4.18789	2.55637
H	-2.18452	-4.73110	3.06092
C	-0.46133	-3.42327	3.29433
H	-0.56117	-3.37155	4.38507
C	0.61042	-2.74737	2.67291
C	-0.05470	-3.76981	-1.02263
H	0.92507	-3.35461	-1.31263
C	-0.10581	-5.24231	-1.48476
H	-1.10119	-5.69365	-1.32123
H	0.63319	-5.86361	-0.95234
H	0.10758	-5.30706	-2.56550
C	-1.14317	-2.94153	-1.74693
H	-1.05974	-1.86764	-1.50201
H	-2.15587	-3.29785	-1.47292
H	-1.05152	-3.04407	-2.84271
C	1.62170	-2.00752	3.54796
H	2.27594	-1.44191	2.86273
C	2.49752	-3.01351	4.33251
H	3.00652	-3.72281	3.65882
H	1.88739	-3.60563	5.03836
H	3.26933	-2.48337	4.91791
C	0.96418	-1.00464	4.51827
H	0.31428	-1.50739	5.25765
H	0.35713	-0.26084	3.97560
H	1.73701	-0.45683	5.08491
C	3.31743	-4.70986	0.49411
H	4.32900	-5.12886	0.35818
H	2.65229	-5.18982	-0.24099
H	2.96818	-4.98269	1.50392
C	4.77464	-2.10531	1.30392
H	4.76577	-1.00410	1.27932
H	5.75492	-2.44846	0.93107
H	4.68052	-2.42228	2.35650
C	2.20941	-2.03953	-3.75183
H	1.22238	-2.29312	-3.33211
H	2.65600	-2.96341	-4.15876
H	2.04818	-1.34120	-4.59031
C	4.94473	-0.72761	-3.33384
H	5.22477	-1.48199	-4.08877
H	5.78779	-0.61493	-2.63530
H	4.79155	0.23394	-3.85170
C	2.82677	1.39864	-1.82819
C	1.89543	2.08163	-2.67281
C	2.05355	3.46388	-2.89825
H	1.32871	3.98341	-3.53611
C	3.11057	4.18326	-2.33145
H	3.20997	5.25953	-2.50960
C	4.05522	3.50198	-1.55452
H	4.90502	4.05392	-1.13606
C	3.94895	2.12008	-1.30494
C	0.75787	1.35419	-3.39281
H	0.78716	0.30066	-3.06598
C	0.98233	1.38237	-4.92378
H	1.97304	0.98127	-5.19888
H	0.92553	2.41351	-5.31640
H	0.21156	0.78496	-5.44317
C	-0.63896	1.91168	-3.04420
H	-0.72671	2.98086	-3.30865
H	-0.86789	1.80428	-1.97173
H	-1.41698	1.36833	-3.61074
C	5.06055	1.43449	-0.50587
H	4.89302	0.34786	-0.59430
C	6.46362	1.75158	-1.07243
H	7.22971	1.13963	-0.56366
H	6.73666	2.81035	-0.91604
H	6.52366	1.54941	-2.15422
C	5.01349	1.78500	0.99708
H	4.06769	1.45636	1.45713
H	5.10325	2.87510	1.15409
H	5.84519	1.29926	1.53911
C	-3.06207	1.37428	-0.16305
C	-5.30579	0.29892	-0.54419
C	-6.05719	-0.41515	0.60952
H	-5.57442	-1.39318	0.81429
H	-5.97945	0.19461	1.52982
C	-5.98517	1.66628	-0.82048

H -5.43363 2.18250 -1.62715
H -5.90411 2.29925 0.08247
C -5.39098 -0.57964 -1.81980
H -4.89251 -1.55043 -1.63214
H -4.83642 -0.08286 -2.63640
C -7.54044 -0.63212 0.22214
H -8.06070 -1.14024 1.05571
C -8.20507 0.73873 -0.05193
H -9.27130 0.59469 -0.31009
H -8.17586 1.36557 0.85973
C -7.46619 1.44916 -1.21128
H -7.93676 2.43110 -1.40617
C -7.53944 0.57343 -2.48528
H -8.59405 0.42746 -2.78694
H -7.03068 1.08191 -3.32603
C -6.87307 -0.79639 -2.21073
H -6.91660 -1.42264 -3.12131
C -7.61324 -1.50707 -1.05231
H -8.66964 -1.68484 -1.32863
H -7.16026 -2.49936 -0.86095
C -0.43625 0.34414 0.30751
C   0.32599 2.36114 1.58509
C   0.06981 3.51580 0.58238
H   0.92271 3.59241 -0.11678
H -0.83051 3.26971 -0.00863
C   1.58274 2.67840 2.41615
H   2.44903 2.76612 1.73734
H   1.79180 1.84412 3.11448
C -0.90067 2.23747 2.51770
H -1.78424 1.99499 1.89996
H -0.73923 1.40265 3.23098
C -0.12826 4.84393 1.35224
H -0.30195 5.66161 0.62674
C -1.35066 4.71299 2.29145
H -2.26275 4.51085 1.69904
H -1.51730 5.66158 2.83836
C -1.10590 3.56042 3.29525
H -1.98217 3.45952 3.96542
C   0.15751 3.86759 4.13505
H   0.33031 3.05938 4.87245
H   0.01489 4.80329 4.70986
C   1.38072 4.00088 3.19638
H   2.28739 4.21511 3.79417
C   1.13541 5.15188 2.19043
H   1.01077 6.10943 2.73275
H   2.01218 5.26631 1.52509

H

SCF (BP86) Energy = -2283.80631075
Enthalpy 0K = -2282.666755
Enthalpy 298K = -2282.600451
Free Energy 298K = -2282.766139
Lowest Frequency = 18.6330 cm^{-1}
Second Frequency = 19.4366 cm^{-1}
SCF (BP86-D3BJ) Energy = -2284.17952190
SCF (tol) Energy = -2283.81480132
SCF (BS2) Energy = -3667.67820374

K   1.53634 2.46995 1.89833
Si -3.77220 1.95409 -0.61077
Si -2.92133 -0.04972 -2.81629
Al -1.15874 0.14964 -0.20090
O -3.84399 1.20751 -2.12904
N -2.11378 1.76982 0.03880
N -2.04890 -0.86202 -1.49754
N   3.17059 1.01438 0.35571
N -0.06142 -0.63284 1.09273
C -1.49768 2.89060 0.67400
C -0.69866 3.82248 -0.07961
C -0.06624 4.89582 0.58423
H   0.53346 5.60130 -0.00278
C -0.21415 5.10336 1.96496
H   0.26140 5.96126 2.45398
C -1.02038 4.21917 2.69751
H -1.17592 4.39430 3.76913
C -1.66945 3.12560 2.08229
C -0.52506 3.69962 -1.59363
H -1.23927 2.92884 -1.92907
C -0.85325 5.01649 -2.33293
H -0.10364 5.80130 -2.12539
H -1.84076 5.41546 -2.04872
H -0.85228 4.84671 -3.42332
C   0.89599 3.22188 -1.97073
H   1.13437 2.24832 -1.51291
H   1.65717 3.95660 -1.64724
H   0.98922 3.11528 -3.06601
C -2.57770 2.24484 2.94045
H -2.97583 1.46018 2.27630
C -3.76706 3.05350 3.50778
H -4.33426 3.55803 2.70772
H -3.42672 3.83236 4.21375
H -4.46017 2.39000 4.05384
C -1.80707 1.54415 4.07936
H -1.34962 2.27226 4.77497
H -1.01294 0.89582 3.67164
H -2.48490 0.90579 4.67248
C -4.21200 3.77981 -0.85251
H -5.27739 3.85789 -1.12903
H -3.61990 4.23403 -1.66196
H -4.05495 4.36930 0.06616
C -5.10786 1.16571 0.48313
H -4.82785 0.15549 0.81958
H -6.04963 1.08718 -0.08695
H -5.30536 1.78043 1.37752
C -1.78544 0.78501 -4.08996
H -0.99068 1.37538 -3.60418
H -2.37570 1.46609 -4.72711
H -1.29967 0.04145 -4.74349
C -4.10040 -1.22394 -3.71463
H -4.48806 -0.73312 -4.62347
H -4.95863 -1.49121 -3.07876
H -3.59175 -2.15436 -4.01677
C -1.91061 -2.29923 -1.47589
C -0.80806 -2.94213 -2.12273
C -0.64540 -4.33560 -1.98709
H   0.20604 -4.82071 -2.47865
C -1.54375 -5.11125 -1.24656
H -1.39185 -6.19101 -1.14202
C -2.65882 -4.49156 -0.67104
H -3.39086 -5.09987 -0.12686
C -2.87717 -3.10493 -0.79071
C   0.16634 -2.17971 -3.02235
H -0.06358 -1.10610 -2.91674
C -0.05135 -2.57578 -4.50287
H -1.09791 -2.42416 -4.81841
H   0.18983 -3.64128 -4.66751
H   0.59844 -1.97876 -5.16783
C   1.64487 -2.37566 -2.62812
H   1.94298 -3.43895 -2.65990
H   1.83738 -1.98522 -1.61601
H   2.29940 -1.83024 -3.33186
C -4.17516 -2.52098 -0.22751
H -4.23135 -1.47667 -0.57801
C -5.41917 -3.26921 -0.76053
H -6.34239 -2.74716 -0.45225

H -5.47679 -4.29840 -0.36379
H -5.41325 -3.33885 -1.86071
C -4.20261 -2.49779 1.31648
H -3.39910 -1.86610 1.72858
H -4.07409 -3.51370 1.73154
H -5.16882 -2.10763 1.68481
C   2.10519 0.32266 0.14135
C   4.43655 0.69574 -0.36251
C   5.53739 1.63787 0.18083
H   5.22217 2.68934 0.02787
H   5.63998 1.48420 1.27351
C   4.87798 -0.77057 -0.12688
H   4.08533 -1.44518 -0.50176
H   4.97352 -0.95079 0.96206
C   4.28689 0.93336 -1.88658
H   3.96201 1.97757 -2.06126
H   3.48588 0.27409 -2.27041
C   6.88318 1.36780 -0.53307
H   7.65681 2.05054 -0.13240
C   7.30765 -0.10058 -0.28816
H   8.27836 -0.30243 -0.78081
H   7.45247 -0.27882 0.79503
C   6.22050 -1.05259 -0.84279
H   6.51969 -2.10321 -0.66404
C   6.05183 -0.81107 -2.36232
H   7.00161 -1.02604 -2.88917
H   5.29040 -1.50087 -2.77351
C   5.62699 0.65653 -2.60952
H   5.50191 0.82978 -3.69569
C   6.71473 1.60703 -2.05328
H   7.67620 1.43252 -2.57370
H   6.43197 2.66067 -2.24317
C   0.78952 0.17195 0.28769
C   0.45193 -1.64836 2.02975
C   1.26212 -2.74788 1.28840
H   0.61568 -3.20470 0.51735
H   2.11715 -2.28117 0.76490
C -0.74317 -2.32564 2.73647
H -1.38708 -2.78888 1.96783
H -1.34751 -1.55654 3.25608
C   1.37704 -1.01177 3.11033
H   2.24228 -0.53333 2.61172
H   0.81053 -0.22616 3.65271
C   1.76477 -3.82226 2.28297
H   2.33813 -4.59086 1.73027
C   2.67391 -3.16372 3.34732
H   3.55877 -2.70760 2.86297
H   3.04997 -3.92740 4.05538
C   1.87259 -2.08409 4.11222
H   2.52147 -1.60617 4.87192
C   0.65331 -2.73763 4.80514
H   0.08339 -1.97516 5.37140
H   0.99475 -3.49416 5.53779
C -0.25304 -3.39800 3.73906
H -1.12894 -3.86103 4.23227
C   0.55006 -4.48054 2.97927
H   0.88974 -5.26622 3.68203
H -0.09514 -4.97159 2.22667

G·Et_2O

SCF (BP86) Energy = -2517.40625032
Enthalpy 0K = -2516.135929
Enthalpy 298K = -2516.058701
Free Energy 298K = -2516.258254
Lowest Frequency = 4.0838 cm^{-1}
Second Frequency = 6.6383 cm^{-1}
SCF (BP86-D3BJ) Energy = -2517.79355149
SCF (tol) Energy = -2517.41426954
SCF (BS2) Energy = -3901.33928076

K   1.51818 2.41472 0.08900
Si -4.02142 1.09475 -2.26899
Si -3.03289 -1.78974 -2.85752
Al -1.71691 -0.13241 -0.46147
O -3.84456 -0.33067 -3.18308
O   2.88081 4.66277 1.24592
N -2.54799 1.34403 -1.31747
N -2.42889 -1.72681 -1.18686
N   5.00662 0.27148 -0.47097
N -1.17922 -0.15166 1.39307
C -1.79242 2.55203 -1.39587
C -0.77713 2.73435 -2.39768
C -0.02724 3.92959 -2.42591
H   0.73102 4.06234 -3.20756
C -0.25447 4.96316 -1.50546
H   0.31580 5.89721 -1.56270
C -1.24804 4.79378 -0.53219
H -1.44541 5.60449 0.17890
C -2.01702 3.61430 -0.45625
C -0.47594 1.66286 -3.44557
H -1.28693 0.91872 -3.37813
C -0.46275 2.21641 -4.88691
H   0.38601 2.90282 -5.05967
H -1.38933 2.76700 -5.11986
H -0.36672 1.38843 -5.61054
C   0.85361 0.93581 -3.13360
H   0.81357 0.45022 -2.14127
H   1.70522 1.64294 -3.15709
H   1.06182 0.15252 -3.88443
C -3.10643 3.51291 0.61021
H -3.42257 2.45562 0.63700
C -4.33383 4.37500 0.23145
H -4.74675 4.08631 -0.74916
H -4.06322 5.44515 0.17628
H -5.13514 4.26795 0.98422
C -2.60113 3.90243 2.01539
H -2.33519 4.97361 2.07773
H -1.71359 3.31110 2.29848
H -3.38519 3.71958 2.77086
C -4.30319 2.49916 -3.50881
H -5.25587 2.33667 -4.04095
H -3.49760 2.53314 -4.25964
H -4.35007 3.48371 -3.01446
C -5.54688 0.88217 -1.16255
H -5.36654 0.12021 -0.38668
H -6.41919 0.56388 -1.75873
H -5.80427 1.82715 -0.65450
C -1.67135 -1.94063 -4.17800
H -0.86289 -1.20629 -4.03503
H -2.10765 -1.78020 -5.17939
H -1.21938 -2.94706 -4.16316
C -4.23384 -3.22594 -3.15925
H -4.36657 -3.36035 -4.24659
H -5.22355 -3.03346 -2.71772
H -3.84487 -4.17048 -2.74374
C -2.27046 -2.95234 -0.44584
C -1.01059 -3.63130 -0.40324
C -0.86939 -4.78331 0.39637
H   0.10335 -5.28885 0.43168
C -1.93778 -5.29935 1.13543
H -1.80747 -6.19461 1.75324
C -3.18231 -4.66264 1.06063
H -4.02891 -5.07214 1.62374
C -3.37607 -3.50637 0.28075
C   0.19589 -3.17247 -1.22531

H -0.12434 -2.29219 -1.80842
C 0.64175 -4.26797 -2.22159
H -0.19342 -4.60067 -2.86107
H 1.02728 -5.15958 -1.69484
H 1.44855 -3.89431 -2.87782
C 1.38190 -2.73526 -0.33708
H 1.72654 -3.56550 0.30671
H 1.11045 -1.88124 0.30757
H 2.23567 -2.42332 -0.96835
C -4.76557 -2.86604 0.25442
H -4.77020 -2.14609 -0.58073
C -5.89754 -3.88900 0.01546
H -6.86117 -3.36649 -0.12035
H -6.01998 -4.57408 0.87334
H -5.71157 -4.50432 -0.87997
C -5.03892 -2.06375 1.54471
H -4.28114 -1.27470 1.67866
H -5.00612 -2.71935 2.43383
H -6.03519 -1.58639 1.51193
C 4.18405 1.12326 -0.42380
C 5.97287 -0.79393 -0.51680
C 5.90085 -1.49310 -1.90286
H 4.87864 -1.88218 -2.05982
H 6.09987 -0.75026 -2.69692
C 7.39676 -0.21068 -0.30066
H 7.43355 0.30767 0.67501
H 7.60662 0.54027 -1.08432
C 5.65521 -1.82382 0.60323
H 4.63097 -2.21222 0.46012
H 5.68257 -1.31453 1.58388
C 6.94013 -2.63880 -1.94704
H 6.88261 -3.13022 -2.93568
C 8.35858 -2.05806 -1.73203
H 9.11036 -2.86757 -1.78564
H 8.60286 -1.33778 -2.53601
C 8.42968 -1.36213 -0.35136
H 9.43950 -0.93906 -0.19670
C 8.11426 -2.38826 0.76374
H 8.86233 -3.20288 0.74914
H 8.18268 -1.90573 1.75733
C 6.69563 -2.96820 0.54780
H 6.46271 -3.69581 1.34676
C 6.62424 -3.66495 -0.83232
H 7.34835 -4.50014 -0.87189
H 5.61965 -4.10091 -0.98816
C -0.13153 0.26963 0.68501
C -1.16799 -0.34029 2.89757
C -1.17086 -1.86419 3.17533
H -2.04126 -2.32962 2.67861
H -0.26876 -2.31359 2.71976
C -2.43382 0.29183 3.51588
H -3.33365 -0.13080 3.03333
H -2.43855 1.37888 3.31306
C 0.07618 0.28750 3.55529
H 0.98570 -0.12560 3.08108
H 0.08898 1.37665 3.34806
C -1.19974 -2.12804 4.69917
H -1.20501 -3.21980 4.88163
C 0.05251 -1.49496 5.35053
H 0.96937 -1.95644 4.93554
H 0.05774 -1.68721 6.44144
C 0.05763 0.02929 5.08040
H 0.95736 0.48319 5.54211
C -1.21428 0.66152 5.69616
H -1.21097 1.75703 5.53313
H -1.23118 0.49778 6.79154
C -2.46789 0.03225 5.04255
H -3.38088 0.48801 5.47246
C -2.47310 -1.49266 5.30750
H -2.51523 -1.68809 6.39691
H -3.37559 -1.95207 4.86113
C 2.78775 5.09315 2.61186
H 2.48995 6.16379 2.64532
H 3.78601 5.01505 3.09538
C 1.76860 4.23133 3.34499
H 2.06414 3.16860 3.33685
H 0.76831 4.32481 2.88976
H 1.69055 4.54995 4.39758
C 3.82398 5.45275 0.50750
H 4.82491 5.38291 0.98673
H 3.51914 6.52159 0.53860
C 3.88416 4.95213 -0.93024
H 2.89657 5.03611 -1.41544
H 4.21219 3.89935 -0.96595
H 4.60025 5.55600 -1.51204

TS(G·Et$_2$O-3·Et$_2$O)
SCF (BP86) Energy = -2517.38320301
Enthalpy 0K = -2516.113147
Enthalpy 298K = -2516.037128
Free Energy 298K = -2516.228873
Lowest Frequency = -58.3867 cm-1
Second Frequency = 9.9053 cm-1
SCF (BP86-D3BJ) Energy = -
2517.78224754
SCF (tol) Energy = -2517.39060952
SCF (BS2) Energy = -3901.3157921

K 2.21251 1.42051 0.89821
Si -3.38817 2.95620 -0.85089
Si -3.44861 0.59935 -2.86786
Al -1.59644 0.36118 -0.31973
O -3.75960 2.18043 -2.31510
O 4.72910 1.80253 2.15097
N -1.90523 2.22726 -0.18331
N -2.88352 -0.35997 -1.48283
N 3.12966 -1.07038 -0.45223
N -0.94208 -0.80281 1.09444
C -0.83707 3.05686 0.27214
C 0.18777 3.51169 -0.62993
C 1.25041 4.29961 -0.13742
H 2.01387 4.65949 -0.83789
C 1.33567 4.65928 1.21655
H 2.15261 5.29635 1.57444
C 0.32992 4.23288 2.09493
H 0.37151 4.53658 3.14769
C -0.75753 3.45257 1.65041
C 0.16175 3.17090 -2.12092
H -0.83657 2.75144 -2.33120
C 0.36056 4.40862 -3.02308
H 1.38518 4.81567 -2.94617
H -0.33995 5.22010 -2.76561
H 0.19689 4.13690 -4.08012
C 1.20605 2.08397 -2.47156
H 1.02862 1.15323 -1.90433
H 2.23263 2.44421 -2.26071
H 1.16696 1.83533 -3.54718
C -1.86357 3.10182 2.64498
H -2.54451 2.40559 2.12655
C -2.66716 4.36545 3.03431
H -3.07842 4.87645 2.14771
H -2.03055 5.09263 3.57012
H -3.50867 4.10227 3.69924
C -1.33248 2.39720 3.91044
H -0.65782 3.04975 4.49435
H -0.78267 1.47678 3.65333

```
H  -2.16886  2.11585  4.57385
C  -3.13643  4.79253 -1.24153
H  -4.09801  5.23146 -1.55802
H  -2.41428  4.93555 -2.06050
H  -2.77745  5.35447 -0.36313
C  -4.88115  2.77668  0.30401
H  -4.98147  1.74718  0.68126
H  -5.80933  3.03786 -0.23274
H  -4.79034  3.44937  1.17369
C  -2.15979  0.77851 -4.25396
H  -1.16804  1.05518 -3.86025
H  -2.47760  1.56189 -4.96368
H  -2.04688 -0.16470 -4.81471
C  -5.03523 -0.09503 -3.63083
H  -5.21737  0.39518 -4.60242
H  -5.90870  0.08981 -2.98711
H  -4.95564 -1.18111 -3.80449
C  -3.26005 -1.74524 -1.34127
C  -2.43633 -2.78621 -1.87510
C  -2.77386 -4.13198 -1.62758
H  -2.13059 -4.92322 -2.03064
C  -3.90760 -4.47877 -0.88558
H  -4.14718 -5.53024 -0.69327
C  -4.74772 -3.46155 -0.41749
H  -5.65832 -3.72769  0.13201
C  -4.46231 -2.10155 -0.64705
C  -1.22299 -2.49246 -2.75866
H  -1.10377 -1.39616 -2.79344
C  -1.47038 -2.99022 -4.20271
H  -2.39674 -2.56619 -4.62707
H  -1.56859 -4.09025 -4.23429
H  -0.62940 -2.71094 -4.86276
C   0.08826 -3.08521 -2.20155
H   0.03085 -4.18480 -2.10923
H   0.33048 -2.66883 -1.21115
H   0.92868 -2.85311 -2.88006
C  -5.47651 -1.05563 -0.17683
H  -5.16720 -0.09199 -0.61533
C  -6.90605 -1.37137 -0.67458
H  -7.58691 -0.53333 -0.44164
H  -7.31825 -2.27172 -0.18514
H  -6.93192 -1.54461 -1.76279
C  -5.48635 -0.88285  1.35763
H  -4.50965 -0.53551  1.73098
H  -5.71923 -1.83730  1.86343
H  -6.25227 -0.14724  1.66373
C   2.48832 -1.59093  0.45667
C   4.39299 -1.33524 -1.15466
C   5.20757 -0.01862 -1.24077
H   4.59549  0.74808 -1.75601
H   5.40906  0.34827 -0.21546
C   5.24368 -2.41122 -0.42887
H   4.65261 -3.34177 -0.34868
H   5.45268 -2.07215  0.60346
C   4.07675 -1.82271 -2.59290
H   3.45321 -1.06610 -3.10418
H   3.47983 -2.75088 -2.53391
C   6.52850 -0.25880 -2.01016
H   7.09819  0.68870 -2.05982
C   7.36516 -1.33424 -1.27584
H   8.32104 -1.49863 -1.80879
H   7.62040 -0.98715 -0.25570
C   6.56133 -2.65476 -1.20206
H   7.15667 -3.42373 -0.67409
C   6.23442 -3.14089 -2.63467
H   7.16997 -3.33740 -3.19247
H   5.67639 -4.09552 -2.59346
C   5.39549 -2.06563 -3.36652
H   5.15375 -2.41284 -4.38853
C   6.20174 -0.74690 -3.44211
H   7.13778 -0.90534 -4.01075
H   5.62154  0.02435 -3.98419
C   0.12119 -0.31938  0.47088
C  -0.88286 -1.76791  2.25728
C  -0.66265 -3.19527  1.69209
H  -1.47218 -3.43857  0.97960
H   0.29282 -3.20440  1.13741
C  -2.21399 -1.73043  3.03120
H  -3.03821 -1.97819  2.33924
H  -2.39695 -0.70663  3.41299
C   0.28968 -1.43019  3.20701
H   1.22557 -1.45443  2.62168
H   0.15655 -0.40408  3.60634
C  -0.62829 -4.21992  2.85181
H  -0.47884 -5.23456  2.43530
C   0.53763 -3.87205  3.80735
H   1.50016 -3.92516  3.26470
H   0.58578 -4.60597  4.63562
C   0.32838 -2.44701  4.37413
H   1.16477 -2.19226  5.05433
C  -1.00774 -2.39380  5.15361
H  -1.15824 -1.38378  5.58258
H  -0.98278 -3.10445  6.00265
C  -2.17518 -2.74511  4.20024
H  -3.13328 -2.70298  4.75308
C  -1.96498 -4.16879  3.62978
H  -1.95707 -4.91023  4.45249
H  -2.80361 -4.43489  2.95874
C   5.16838  0.92151  3.20340
H   5.47016  1.52665  4.08494
H   6.06668  0.36395  2.86162
C   4.04545 -0.03924  3.56852
H   3.74042 -0.65831  2.70637
H   3.16448  0.50379  3.95204
H   4.38341 -0.72269  4.36496
C   5.73238  2.78181  1.83672
H   6.68137  2.26867  1.57037
H   5.93502  3.40532  2.73396
C   5.25142  3.64640  0.67877
H   4.30173  4.14859  0.92865
H   5.10554  3.04194 -0.23238
H   5.99829  4.42493  0.45166
```

3·Et$_2$O

SCF (BP86) Energy = -2517.47722829
Enthalpy 0K = -2516.204046
Enthalpy 298K = -2516.128606
Free Energy 298K = -2516.317747
Lowest Frequency = 12.8768 cm^{-1}
Second Frequency = 17.2252 cm^{-1}
SCF (BP86-D3BJ) Energy = -2517.8793527
SCF (tol) Energy = -2517.48364423
SCF (BS2) Energy = -3901.40941287

```
K   2.35656  1.94054  0.42898
Si -3.37868  2.66502 -1.21849
Si -3.77411 -0.16755 -2.40329
Al -1.47203  0.26472 -0.27886
O  -4.05769  1.50532 -2.25021
O   4.70951  3.00327  1.48550
N  -1.76942  2.08970 -0.68995
N  -2.92765 -0.71285 -0.93812
N   2.90339 -0.55255 -0.53559
N  -0.41657 -0.34555  1.13539
C  -0.69320  3.02906 -0.64252
C   0.15910  3.23661 -1.78365
```

C	1.22916	4.15250	-1.69670
H	1.86327	4.30962	-2.57716
C	1.47671	4.89313	-0.53062
H	2.29431	5.62186	-0.49527
C	0.63323	4.71335	0.57509
H	0.79652	5.31188	1.47961
C	-0.44734	3.80532	0.54165
C	-0.07061	2.50939	-3.10880
H	-1.05184	2.01222	-3.02753
C	-0.11885	3.47563	-4.31399
H	0.87047	3.92170	-4.52222
H	-0.82984	4.30259	-4.15325
H	-0.42628	2.93004	-5.22275
C	0.99334	1.41477	-3.35177
H	1.00748	0.67336	-2.53697
H	2.00242	1.86025	-3.43655
H	0.79014	0.88102	-4.29720
C	-1.35445	3.71135	1.76872
H	-2.13081	2.96528	1.53213
C	-2.04820	5.06146	2.06111
H	-2.59800	5.43509	1.18099
H	-1.31711	5.83859	2.34849
H	-2.76505	4.95578	2.89414
C	-0.59501	3.21047	3.01522
H	0.22529	3.89706	3.29697
H	-0.17344	2.20724	2.83462
H	-1.27420	3.13685	3.88272
C	-3.24234	4.28259	-2.19359
H	-4.25683	4.67183	-2.38585
H	-2.75142	4.12579	-3.16646
H	-2.68047	5.05363	-1.64061
C	-4.59905	2.94299	0.20902
H	-4.59301	2.11130	0.93037
H	-5.62148	3.03693	-0.19592
H	-4.36684	3.87147	0.75740
C	-2.76115	-0.37593	-3.99704
H	-1.72612	-0.01664	-3.87199
H	-3.22677	0.20128	-4.81448
H	-2.71500	-1.43280	-4.30858
C	-5.44480	-1.02821	-2.61642
H	-5.86106	-0.78344	-3.60842
H	-6.16500	-0.69584	-1.85279
H	-5.34761	-2.12419	-2.54524
C	-3.27815	-1.96474	-0.31124
C	-2.65120	-3.18778	-0.70935
C	-2.93649	-4.37288	-0.00182
H	-2.44526	-5.30438	-0.30699
C	-3.83070	-4.38855	1.07396
H	-4.02825	-5.31733	1.62013
C	-4.49632	-3.20642	1.41802
H	-5.23276	-3.22088	2.23018
C	-4.26046	-1.99896	0.73154
C	-1.72968	-3.27084	-1.92725
H	-1.56238	-2.23933	-2.27994
C	-2.41428	-4.06465	-3.06598
H	-3.39503	-3.63478	-3.33282
H	-2.58579	-5.11523	-2.77024
H	-1.78344	-4.07101	-3.97310
C	-0.34823	-3.87684	-1.60597
H	-0.43134	-4.89706	-1.19033
H	0.19774	-3.24638	-0.88610
H	0.25911	-3.94517	-2.52665
C	-5.11212	-0.77879	1.09046
H	-4.89943	-0.00939	0.32922
C	-6.62404	-1.09922	1.03396
H	-7.21700	-0.17550	1.15657
H	-6.92437	-1.79100	1.84102
H	-6.90707	-1.56625	0.07630

C	-4.75800	-0.18237	2.47026
H	-3.71647	0.17586	2.50389
H	-4.87828	-0.93445	3.27079
H	-5.42131	0.66836	2.71060
C	1.64955	-0.74100	-0.31555
C	3.72299	-1.63057	-1.16103
C	5.19299	-1.14964	-1.19853
H	5.24785	-0.20225	-1.77127
H	5.52885	-0.92709	-0.16642
C	3.65590	-2.94822	-0.34861
H	2.60397	-3.28766	-0.30808
H	3.97569	-2.74855	0.69321
C	3.25779	-1.91364	-2.61186
H	3.29665	-0.97309	-3.19527
H	2.20084	-2.23836	-2.58762
C	6.10065	-2.22186	-1.84730
H	7.14634	-1.85874	-1.86521
C	6.01839	-3.53000	-1.02378
H	6.67617	-4.30116	-1.46935
H	6.38081	-3.35345	0.00756
C	4.55402	-4.03011	-0.99415
H	4.49155	-4.96297	-0.40149
C	4.07299	-4.29958	-2.44033
H	4.69756	-5.08479	-2.90875
H	3.03286	-4.67714	-2.42919
C	4.15434	-2.99268	-3.26564
H	3.80721	-3.18338	-4.29940
C	5.61946	-2.49391	-3.29325
H	6.27081	-3.24903	-3.77433
H	5.69497	-1.57001	-3.89910
C	0.43678	-0.29445	-0.00425
C	-0.06830	-1.09041	2.35813
C	0.10238	-2.60853	2.07238
H	-0.83141	-2.99167	1.62276
H	0.90762	-2.75017	1.32795
C	-1.20708	-0.92195	3.38870
H	-2.14056	-1.31127	2.94488
H	-1.36120	0.15530	3.59412
C	1.25588	-0.56498	2.98853
H	2.07989	-0.68841	2.25932
H	1.14584	0.51791	3.20468
C	0.43448	-3.37914	3.37270
H	0.55108	-4.45499	3.14008
C	1.74938	-2.83514	3.97893
H	2.58613	-2.98533	3.26971
H	2.00411	-3.38953	4.90308
C	1.58585	-1.32944	4.29462
H	2.52756	-0.93525	4.72495
C	0.43076	-1.13509	5.30544
H	0.31827	-0.06131	5.55309
H	0.66192	-1.66179	6.25156
C	-0.88272	-1.68194	4.69739
H	-1.71208	-1.53781	5.41603
C	-0.71795	-3.18893	4.38717
H	-0.50605	-3.74912	5.31888
H	-1.65849	-3.59369	3.96847
C	5.05666	3.15015	2.87177
H	5.35485	4.20228	3.06981
H	5.93362	2.50895	3.10471
C	3.86474	2.75971	3.73568
H	3.57988	1.70657	3.57160
H	2.99322	3.40305	3.52612
H	4.11740	2.87476	4.80253
C	5.80667	3.35522	0.62588
H	6.68515	2.71929	0.86642
H	6.10000	4.41052	0.81384
C	5.39089	3.16205	-0.82687
H	4.52496	3.79813	-1.07994

H   5.13764 2.10702 -1.02921
H   6.22003 3.44195 -1.49739

I

SCF (BP86) Energy = -2686.88830900
Enthalpy 0K = -2685.597203
Enthalpy 298K = -2685.520367
Free Energy 298K = -2685.706205
Lowest Frequency = 15.1340 cm^{-1}
Second Frequency = 22.1038 cm^{-1}
SCF (BP86-D3BJ) Energy = -2687.32843993
SCF (tol) Energy = -2686.89721173
SCF (BS2) Energy = -4070.85312648

```
K   2.09444 -1.24804 -2.86457
Si -2.74172 -2.89128 -0.03456
Si -2.06777 -1.74016  2.75110
Al -0.74059 -0.39477  0.23128
O  -3.10688 -2.15066  1.44893
N  -1.25801 -2.09071 -0.60522
N  -1.04211 -0.43978  2.13329
N   3.69770 -0.17118 -1.05343
N   0.21334  0.91915 -0.71829
C  -0.54367 -2.72847 -1.66437
C   0.51804 -3.66828 -1.39518
C   1.20316 -4.28441 -2.46479
H   1.99965 -5.00186 -2.23525
C   0.87662 -4.02730 -3.80444
H   1.40197 -4.54071 -4.61777
C  -0.16022 -3.12425 -4.07783
H  -0.44151 -2.92885 -5.11967
C  -0.87461 -2.47667 -3.04502
C   0.94473 -4.03618  0.02435
H   0.21518 -3.55894  0.69908
C   0.93445 -5.56304  0.26858
H   1.73714 -6.06874 -0.29801
H  -0.02201 -6.02760 -0.01796
H   1.11274 -5.77542  1.33736
C   2.33892 -3.46541  0.36205
H   2.35447 -2.36821  0.27732
H   3.11292 -3.88673 -0.30682
H   2.62109 -3.73052  1.39591
C  -1.97841 -1.50073 -3.45390
H  -2.50805 -1.21587 -2.53032
C  -2.99320 -2.12660 -4.43608
H  -3.39262 -3.08252 -4.06009
H  -2.54028 -2.32199 -5.42473
H  -3.84355 -1.44156 -4.59801
C  -1.37291 -0.21409 -4.05726
H  -0.76674 -0.43985 -4.95569
H  -0.74218  0.30131 -3.31281
H  -2.16465  0.48960 -4.37177
C  -2.55355 -4.76951  0.16530
H  -3.53750 -5.20082  0.41895
H  -1.84654 -5.04044  0.96311
H  -2.21458 -5.23794 -0.77452
C  -4.31826 -2.67356 -1.07815
H  -4.54571 -1.62887 -1.33900
H  -5.17043 -3.07200 -0.49943
H  -4.25839 -3.25525 -2.01297
C  -1.21961 -3.34984  3.31717
H  -0.45821 -3.72198  2.61418
H  -1.99183 -4.13044  3.43600
H  -0.73182 -3.21327  4.29582
C  -3.17332 -1.22850  4.20196
H  -3.65348 -2.12505  4.63018
H  -3.96448 -0.52803  3.89587
H  -2.57793 -0.74992  4.99754
C  -0.45972  0.51147  3.05324
C   0.79402  0.24849  3.69801
C   1.38525  1.23405  4.51313
H   2.35150  1.01886  4.98426
C   0.77276  2.46836  4.74150
H   1.25568  3.22644  5.36762
C  -0.48446  2.70228  4.17860
H  -0.99714  3.64931  4.38554
C  -1.12374  1.74792  3.36218
C   1.50844 -1.09857  3.60078
H   0.93405 -1.72371  2.89741
C   1.54113 -1.79763  4.98155
H   0.53840 -1.86321  5.43691
H   2.18256 -1.24222  5.68919
H   1.94972 -2.82055  4.89521
C   2.93983 -0.96494  3.04609
H   3.57119 -0.33733  3.70113
H   2.91757 -0.51196  2.04177
H   3.42346 -1.95611  2.97608
C  -2.53921  2.08045  2.88607
H  -2.90388  1.20113  2.32877
C  -3.49511  2.34386  4.07457
H  -4.53057  2.49449  3.71647
H  -3.20752  3.25900  4.62182
H  -3.49737  1.51317  4.79727
C  -2.56675  3.29800  1.93861
H  -1.89712  3.14642  1.07879
H  -2.23529  4.21395  2.46008
H  -3.58772  3.48305  1.55790
C   2.58966  0.12028 -0.45418
C   5.02809  0.16004 -0.48197
C   5.95398  0.54532 -1.66587
H   5.96140 -0.28469 -2.40076
H   5.53231  1.43071 -2.18098
C   5.01077  1.33361  0.52662
H   4.33608  1.08903  1.36625
H   4.58862  2.22897  0.02952
C   5.62530 -1.09478  0.21047
H   5.63406 -1.93491 -0.51197
H   4.96729 -1.39279  1.04653
C   7.38857  0.84091 -1.16806
H   8.02838  1.11506 -2.02902
C   7.34796  2.01084 -0.15542
H   8.37119  2.24189  0.19940
H   6.96249  2.92521 -0.64664
C   6.44150  1.62772  1.03872
H   6.40781  2.46456  1.76257
C   7.01195  0.36643  1.73159
H   8.02809  0.57288  2.12039
H   6.38235  0.09328  2.59969
C   7.05726 -0.80483  0.72081
H   7.46159 -1.70868  1.21618
C   7.96091 -0.41860 -0.47429
H   8.99343 -0.22385 -0.12497
H   8.01722 -1.25755 -1.19514
C   1.26490  0.03490 -0.37099
C   0.48126  2.29277 -1.19911
C   1.11576  3.15535 -0.07149
H   0.43717  3.15485  0.80094
H   2.05692  2.67901  0.25810
C  -0.84013  2.95669 -1.65011
H  -1.55877  2.94644 -0.81194
H  -1.28721  2.35543 -2.46427
C   1.44882  2.30470 -2.42300
H   2.40630  1.83163 -2.14297
H   0.99550  1.70868 -3.24395
C   1.37212  4.60065 -0.55964
```

```
H   1.82124  5.19190  0.26115
C   2.33533  4.57766 -1.76953
H   3.30617  4.13575 -1.47401
H   2.54109  5.60905 -2.11637
C   1.70450  3.75147 -2.91553
H   2.39630  3.72628 -3.78000
C   0.36518  4.39622 -3.34026
H  -0.08273  3.82768 -4.17894
H   0.53586  5.42796 -3.70409
C  -0.59788  4.40931 -2.13025
H  -1.56380  4.86095 -2.43078
C   0.02916  5.24090 -0.98545
H   0.18895  6.28440 -1.32050
H  -0.66146  5.28071 -0.12182
C  -2.75125  0.44930 -0.20161
N  -3.85106  0.84504 -0.41372
C  -5.16878  1.23113 -0.62677
C  -5.47465  2.00980 -1.77659
C  -6.16041  0.81466  0.30436
C  -6.81738  2.36725 -1.97948
C  -7.48742  1.19998  0.04363
C  -7.81776  1.96745 -1.08145
H  -7.07546  2.96606 -2.85935
H  -8.26945  0.88789  0.74390
H  -8.85898  2.25384 -1.26102
C  -4.39597  2.43494 -2.74066
H  -3.66397  3.10552 -2.25839
H  -3.82637  1.56789 -3.11756
H  -4.83003  2.96403 -3.60303
C  -5.80514  0.01767  1.53321
H  -5.08188 -0.78934  1.32593
H  -5.33746  0.67009  2.29343
H  -6.70902 -0.42128  1.98388
```

J

SCF (BP86) Energy = -2686.90477079
Enthalpy 0K = -2685.615210
Enthalpy 298K = -2685.536929
Free Energy 298K = -2685.732722
Lowest Frequency = 7.7229 cm^{-1}
Second Frequency = 9.8385 cm^{-1}
SCF (BP86-D3BJ) Energy = -2687.32230264
SCF (tol) Energy = -2686.91256738
SCF (BS2) Energy = -4070.87107112

```
K  -1.91452 -0.34326 -2.06907
Si  3.65258 -2.39683 -1.98172
Si  4.93930  0.10414 -0.68899
Al  1.84913 -0.42463 -0.18522
O   4.89801 -1.29289 -1.66551
N   2.08136 -1.61279 -1.64348
N   3.55920  0.02866  0.43177
N   0.34046 -0.28717  0.90868
C   1.01185 -1.87051 -2.55923
C   0.82713 -1.03372 -3.71490
C  -0.23754 -1.29705 -4.60511
H  -0.34503 -0.67418 -5.50045
C  -1.12417 -2.36550 -4.39658
H  -1.92861 -2.56963 -5.11222
C  -0.93282 -3.19579 -3.28410
H  -1.60469 -4.04687 -3.12510
C   0.12040 -2.97919 -2.36923
C   1.77801  0.12300 -4.01160
H   2.68034 -0.05363 -3.40277
C   2.20695  0.19312 -5.49264
H   1.37377  0.49653 -6.15249
H   2.58857 -0.77521 -5.85827
H   3.00511  0.94495 -5.61727
C   1.18179  1.47375 -3.56087
H   0.94061  1.48583 -2.48719
H   0.25703  1.69628 -4.12664
H   1.89392  2.29341 -3.76317
C   0.30273 -3.97239 -1.22019
H   1.22668 -3.67994 -0.69431
C   0.47881 -5.41734 -1.73750
H   1.29737 -5.48982 -2.47329
H  -0.43912 -5.79259 -2.22505
H   0.70876 -6.09927 -0.90002
C  -0.85388 -3.89257 -0.20118
H  -1.82782 -4.11397 -0.67369
H  -0.90600 -2.88654  0.24844
H  -0.70157 -4.61990  0.61645
C   3.78333 -2.89909 -3.80391
H   4.73181 -3.44430 -3.95008
H   3.79065 -2.02357 -4.47206
H   2.95839 -3.56313 -4.11044
C   3.98587 -3.94153 -0.93017
H   3.84245 -3.74912  0.14426
H   5.02921 -4.26932 -1.08216
H   3.32500 -4.77605 -1.21922
C   4.88715  1.57480 -1.89149
H   3.88214  1.71105 -2.32425
H   5.59514  1.39828 -2.71983
H   5.17051  2.51699 -1.39346
C   6.58759  0.11521  0.23791
H   7.41800  0.27026 -0.47298
H   6.75612 -0.83952  0.76124
H   6.61782  0.92686  0.98456
C   3.73110  0.41906  1.81027
C   3.55205  1.78010  2.21915
C   3.61946  2.10428  3.58841
H   3.47141  3.14666  3.89491
C   3.87157  1.13099  4.56199
H   3.90352  1.40076  5.62323
C   4.11843 -0.18498  4.15510
H   4.36238 -0.94412  4.90774
C   4.08400 -0.55787  2.79611
C   3.35072  2.91301  1.21062
H   3.24837  2.44695  0.21612
C   4.58880  3.84171  1.18486
H   5.51775  3.28182  0.98048
H   4.72207  4.35615  2.15369
H   4.47830  4.61851  0.40678
C   2.06909  3.73355  1.46399
H   2.05636  4.17994  2.47466
H   1.17351  3.10283  1.34376
H   2.00003  4.56252  0.73607
C   4.48843 -1.98732  2.42121
H   4.55536 -2.02056  1.32028
C   5.87836 -2.35331  2.99655
H   6.22286 -3.31897  2.58562
H   5.84736 -2.45727  4.09571
H   6.63655 -1.58763  2.76004
C   3.45166 -3.04428  2.85898
H   2.49061 -2.90965  2.33649
H   3.25377 -2.98086  3.94416
H   3.81702 -4.06466  2.64539
C  -0.67173  1.66093 -0.40217
C  -1.89063  3.64971 -1.00292
C  -3.15879  3.94332 -1.83919
H  -3.00487  3.57340 -2.87256
H  -4.01140  3.37663 -1.41505
C  -2.12575  4.15132  0.44452
H  -1.22412  3.93280  1.04722
H  -2.96468  3.58429  0.89391
```

C -0.70127 4.43443 -1.61564
H -0.52242 4.07589 -2.64802
H 0.20923 4.20955 -1.02965
C -3.46856 5.45811 -1.84788
H -4.37676 5.64620 -2.45247
C -3.70117 5.93957 -0.39529
H -3.94113 7.02054 -0.38742
H -4.57023 5.41304 0.04505
C -2.43241 5.66810 0.44915
H -2.59836 6.00796 1.48958
C -1.23506 6.43445 -0.16340
H -1.43461 7.52365 -0.15167
H -0.32647 6.26500 0.44499
C -0.99955 5.95283 -1.61550
H -0.14048 6.49721 -2.05301
C -2.26942 6.22422 -2.45797
H -2.48586 7.30987 -2.48311
H -2.10680 5.90261 -3.50499
C 0.14544 0.64835 -0.13969
C -0.46928 -0.29564 2.13964
C -0.26323 1.00712 2.96388
H 0.81217 1.11531 3.19350
H -0.55697 1.87744 2.34782
C -0.03674 -1.49062 3.01912
H 1.03782 -1.38478 3.25446
H -0.16137 -2.42991 2.44607
C -1.98770 -0.43314 1.82384
H -2.30902 0.42455 1.20253
H -2.15652 -1.35517 1.23257
C -1.09366 0.96938 4.26937
H -0.92846 1.90714 4.83431
C -2.59565 0.82707 3.92853
H -2.93457 1.69766 3.33410
H -3.19922 0.81260 4.85696
C -2.82002 -0.47923 3.12903
H -3.89398 -0.58152 2.87654
C -2.36802 -1.68858 3.98187
H -2.54115 -2.63182 3.42675
H -2.96894 -1.74518 4.91033
C -0.86618 -1.54375 4.32480
H -0.53743 -2.41161 4.92844
C -0.64367 -0.23840 5.12608
H -1.21434 -0.27116 6.07483
H 0.42588 -0.13498 5.39001
N -1.65665 2.17590 -1.05319
N -5.07312 -2.19682 -0.54726
C -4.05799 -2.04697 -1.14953
C -6.30702 -2.26777 0.08929
C -6.61892 -3.43352 0.83606
C -7.19137 -1.15732 -0.03373
C -7.87191 -3.47084 1.47411
C -8.42790 -1.25223 0.62812
C -8.76771 -2.39419 1.37239
H -8.14106 -4.35736 2.05964
H -9.13050 -0.41402 0.55616
C -6.80487 0.06649 -0.83664
H -5.88155 0.53152 -0.44537
H -6.60818 -0.18784 -1.89259
H -7.60648 0.82144 -0.81205
C -5.63169 -4.57317 0.93624
H -5.37661 -4.97656 -0.06058
H -4.68323 -4.24466 1.39694
H -6.04139 -5.39505 1.54483
H -9.73807 -2.44369 1.87851

TS(I-K)
SCF (BP86) Energy = -2686.87934290
Enthalpy 0K = -2685.588606
Enthalpy 298K = -2685.513267
Free Energy 298K = -2685.696304
Lowest Frequency = -166.8351 cm^{-1}
Second Frequency = 8.4061 cm^{-1}
SCF (BP86-D3BJ) Energy = -2687.32426080
SCF (tol) Energy = -2686.89018664
SCF (BS2) Energy = -4070.84210828

Si -2.63802 1.88657 2.40803
Si -3.40543 2.29057 -0.68475
Al -1.19209 0.71272 0.23657
O -2.46839 2.45049 0.77578
N -1.74243 0.41964 2.09358
N -2.51187 0.88922 -1.22386
N 3.46881 -0.54744 -1.46768
N 0.55249 1.51988 -0.13286
N 0.17223 -2.20225 -0.59680
C -1.47402 -0.54931 3.11913
C -0.29323 -0.45699 3.94405
C -0.08741 -1.38706 4.97151
H 0.82290 -1.29941 5.58907
C -0.99797 -2.41075 5.24526
H -0.81454 -3.11971 6.06045
C -2.13244 -2.51561 4.43849
H -2.85091 -3.31962 4.63637
C -2.39423 -1.61785 3.39357
C 0.76594 0.62287 3.74568
H 0.39700 1.29631 2.95079
C 2.09525 -0.00234 3.25961
H 2.45199 -0.76551 3.96895
H 2.88676 0.77298 3.17621
H 1.98776 -0.46886 2.27220
C 1.00222 1.45222 5.02590
H 0.06483 1.87032 5.42680
H 1.69542 2.29542 4.81122
H 1.46517 0.85230 5.82522
C -3.65686 -1.86155 2.56973
H -3.71421 -1.07204 1.79935
C -3.61031 -3.23288 1.87169
H -2.75053 -3.30861 1.17735
H -4.52969 -3.41848 1.30540
H -3.50096 -4.05077 2.60780
C -4.94039 -1.78230 3.43962
H -4.96873 -2.59591 4.18614
H -5.83933 -1.87815 2.81609
H -5.00706 -0.82988 3.99699
C -1.88347 3.19103 3.55500
H -0.82281 3.39622 3.33849
H -1.95246 2.85595 4.59908
H -2.45328 4.14264 3.46698
C -4.44441 1.73897 2.96997
H -4.95081 2.71764 2.91949
H -4.45886 1.41179 4.02686
H -5.03093 1.00784 2.38772
C -3.23647 3.89719 -1.67798
H -3.65449 4.74354 -1.10313
H -3.82735 3.81788 -2.61545
H -2.20286 4.13152 -1.95455
C -5.25656 2.16346 -0.29044
H -5.54891 1.21401 0.17350
H -5.84294 2.27465 -1.22620
H -5.56229 2.99140 0.37773
C -2.67324 0.27815 -2.50899
C -1.83283 0.63409 -3.62564
C -1.99084 -0.01620 -4.87062
H -1.35430 0.28532 -5.70457
C -2.99560 -0.96513 -5.08542

```
H  -3.12736 -1.43170 -6.07111
C  -3.84156 -1.28758 -4.02498
H  -4.64994 -2.01160 -4.17600
C  -3.70355 -0.69865 -2.74932
C  -0.81605  1.76963 -3.57850
H  -0.80814  2.16115 -2.54753
C   0.62769  1.30542 -3.90937
H   1.07246  0.78864 -3.04064
H   1.29104  2.16575 -4.12875
H   0.63828  0.65386 -4.80017
C  -1.22405  2.91514 -4.54142
H  -1.15798  2.59433 -5.59572
H  -0.56362  3.79136 -4.41356
H  -2.26374  3.25083 -4.35525
C  -4.70071 -1.14119 -1.67108
H  -4.40761 -0.63651 -0.73419
C  -4.66408 -2.66344 -1.44657
H  -4.95118 -3.20703 -2.35821
H  -5.39119 -2.94640 -0.66243
H  -3.66438 -3.01994 -1.13093
C  -6.14274 -0.71100 -2.04984
H  -6.20642  0.36054 -2.27547
H  -6.84011 -0.93118 -1.21728
H  -6.49637 -1.26359 -2.93973
C   2.55053  0.03256 -0.77221
C   4.72269 -1.01422 -0.81600
C   1.33026  0.45202 -0.50663
C   1.22154  2.85755 -0.02741
C   2.30033  2.84874  1.09422
H   1.79655  2.60019  2.05477
H   3.03506  2.04535  0.90092
C   1.93091  3.25053 -1.36664
H   2.65641  2.46681 -1.63976
H   1.16125  3.28932 -2.15692
C   0.20212  3.96558  0.30080
H  -0.32712  3.71884  1.23739
H  -0.57111  3.99247 -0.48650
C   2.99585  4.21921  1.21421
H   3.76039  4.17697  2.02353
C   3.69507  4.56718 -0.12218
H   4.21601  5.54028 -0.04402
H   4.46005  3.80460 -0.35641
C   2.62686  4.62128 -1.24788
H   3.12287  4.86122 -2.20867
C   1.58087  5.70563 -0.91722
H   2.06357  6.69846 -0.84170
H   0.83208  5.76686 -1.73078
C   0.88196  5.34545  0.42621
H   0.11903  6.09998  0.65975
C   1.94828  5.30258  1.54557
H   1.45860  5.07393  2.52209
H   2.43339  6.29149  1.64928
C  -0.15906 -1.04430 -0.27144
C  -0.35083 -3.51565 -0.50186
C  -0.98586 -4.11680 -1.62515
C  -0.07062 -4.25682  0.68048
C  -1.21877 -3.36865 -2.92200
H  -2.14081 -3.72637 -3.41843
H  -0.40170 -3.54198 -3.65524
H  -1.34746 -2.28553 -2.77866
C   0.64187 -3.62378  1.84709
H   1.55988 -3.10431  1.51371
H   0.90709 -4.37797  2.60034
H   0.01378 -2.86342  2.34587
C   5.18565 -2.31444 -1.53862
H   4.41573 -3.10341 -1.39782
H   5.27004 -2.11348 -2.62192
C   5.81226  0.06663 -1.02325
H   5.47147  0.99825 -0.52743
H   5.89810  0.29008 -2.09956
C   4.57915 -1.31538  0.68976
H   3.79726 -2.08712  0.84929
H   4.23049 -0.40366  1.21516
C   6.53993 -2.80098 -0.96832
H   6.84840 -3.72578 -1.48998
C   6.38592 -3.08379  0.54997
H   7.34500 -3.44503  0.95983
H   5.63994 -3.88839  0.70737
C   5.93206 -1.79442  1.26969
H   5.81699 -1.99720  2.35748
C   7.00118 -0.69201  1.06856
H   7.96896 -1.01275  1.49246
H   6.70399  0.23263  1.59651
C   7.16832 -0.40830 -0.44893
H   7.92810  0.38427 -0.59834
C   7.60815 -1.69804 -1.17960
H   7.74179 -1.50036 -2.25699
H   8.58630 -2.03902 -0.78783
C  -1.37225 -5.47427 -1.52438
H  -1.87568 -5.93427 -2.37610
C  -1.12717 -6.21460 -0.36376
H  -1.43568 -7.26098 -0.30402
C  -0.47915 -5.61211  0.72164
H  -0.27367 -6.17981  1.62816
K   1.64886 -1.74954 -2.95785
```

TS(J-K)

SCF (BP86) Energy = -2686.84163907
Enthalpy 0K = -2685.551100
Enthalpy 298K = -2685.475156
Free Energy 298K = -2685.659864
Lowest Frequency = -381.6236 cm^{-1}
Second Frequency = 10.6051 cm^{-1}
SCF (BP86-D3BJ) Energy = -2687.28088518
SCF (tol) Energy = -2686.84921080
SCF (BS2) Energy = -4070.80452885

```
K   2.36658  1.61846 -1.40297
Si -3.20373  2.95598 -0.97536
Si -3.87878  0.21874 -2.12397
Al -1.42871  0.47639 -0.05209
O  -4.07199  1.90420 -1.98360
N  -1.56970  2.25610 -0.70382
N  -3.00924 -0.36980 -0.67409
N  -0.38537 -0.24212  1.26552
C  -0.47764  3.16206 -0.91435
C   0.06359  3.35982 -2.23409
C   1.16150  4.23091 -2.41377
H   1.55708  4.39021 -3.42433
C   1.74032  4.91422 -1.33354
H   2.59084  5.58673 -1.49124
C   1.17430  4.76943 -0.05981
H   1.58899  5.33955  0.77902
C   0.05809  3.93832  0.16885
C  -0.51104  2.65262 -3.46654
H  -1.49752  2.25575 -3.17700
C  -0.70534  3.60089 -4.67045
H   0.25800  3.91286 -5.11283
H  -1.25176  4.51498 -4.38779
H  -1.27665  3.09069 -5.46477
C   0.35369  1.44546 -3.90393
H   0.40257  0.66245 -3.12449
H   1.38077  1.76750 -4.16858
H  -0.06633  0.96730 -4.80539
C  -0.61501  3.98429  1.54215
```

H	-1.39394	3.20356	1.54442
C	-1.30175	5.35832	1.74255
H	-2.00663	5.59663	0.92831
H	-0.55359	6.17044	1.78004
H	-1.85973	5.37395	2.69528
C	0.34097	3.69632	2.71613
H	1.12648	4.46954	2.80389
H	0.82765	2.71559	2.57667
H	-0.22094	3.69155	3.66678
C	-3.12963	4.61770	-1.88060
H	-4.14263	5.05623	-1.87227
H	-2.82682	4.49760	-2.93170
H	-2.44419	5.33408	-1.40004
C	-4.16594	3.21654	0.63779
H	-4.02080	2.39603	1.35614
H	-5.24526	3.29844	0.42297
H	-3.84588	4.15207	1.12613
C	-2.95571	-0.10927	-3.75328
H	-1.86717	0.01387	-3.65142
H	-3.31468	0.58610	-4.53168
H	-3.14483	-1.13732	-4.10430
C	-5.61250	-0.51675	-2.30296
H	-5.97158	-0.31889	-3.32779
H	-6.31890	-0.04745	-1.60223
H	-5.62756	-1.60596	-2.13775
C	-3.53508	-1.58253	-0.07862
C	-3.08223	-2.86828	-0.51776
C	-3.53921	-4.02877	0.13864
H	-3.17557	-5.00681	-0.19724
C	-4.45187	-3.96207	1.19570
H	-4.78670	-4.87369	1.70196
C	-4.96555	-2.71415	1.56371
H	-5.72418	-2.65811	2.35277
C	-4.55311	-1.52356	0.93207
C	-2.19032	-3.04734	-1.74793
H	-1.88922	-2.04104	-2.08488
C	-2.98826	-3.72060	-2.89154
H	-3.91717	-3.17277	-3.12366
H	-3.27567	-4.75175	-2.61913
H	-2.38075	-3.77541	-3.81277
C	-0.90454	-3.84819	-1.46315
H	-1.12537	-4.84969	-1.05312
H	-0.25617	-3.31794	-0.74796
H	-0.33636	-3.99555	-2.39885
C	-5.29050	-0.23191	1.30406
H	-4.96812	0.54136	0.58729
C	-6.82434	-0.39948	1.16877
H	-7.32433	0.58075	1.26272
H	-7.23068	-1.04987	1.96356
H	-7.11072	-0.84286	0.20191
C	-4.97281	0.27818	2.72786
H	-3.91509	0.55929	2.84461
H	-5.19486	-0.49667	3.48344
H	-5.58989	1.16285	2.96562
C	1.59439	-0.94916	-0.20249
C	3.24777	-2.16377	-1.52717
C	4.74657	-1.93343	-1.84484
H	4.86774	-0.95033	-2.34296
H	5.31386	-1.88718	-0.89647
C	3.10346	-3.54672	-0.83627
H	2.03779	-3.71325	-0.59506
H	3.65509	-3.52510	0.12230
C	2.46393	-2.18921	-2.86527
H	2.56880	-1.20614	-3.37098
H	1.38933	-2.33116	-2.64950
C	5.29316	-3.05282	-2.76203
H	6.36289	-2.86246	-2.97187
C	5.13614	-4.41987	-2.05478
H	5.53851	-5.22766	-2.69593
H	5.72136	-4.43076	-1.11545
C	3.64005	-4.67176	-1.75320
H	3.52162	-5.64635	-1.24258
C	2.84321	-4.68011	-3.07994
H	3.21048	-5.48951	-3.73989
H	1.77528	-4.88723	-2.87957
C	2.99878	-3.31235	-3.78801
H	2.42340	-3.31479	-4.73384
C	4.49578	-3.06171	-4.08858
H	4.88521	-3.84869	-4.76253
H	4.62294	-2.09556	-4.61568
C	0.75434	-0.07289	0.43667
C	-0.37635	-1.14835	2.44813
C	-0.27496	-2.64071	2.03705
H	-1.11550	-2.88282	1.36321
H	0.66377	-2.80096	1.47235
C	-1.69556	-0.94567	3.21999
H	-2.54161	-1.17542	2.54864
H	-1.77453	0.11916	3.51554
C	0.79541	-0.82917	3.40781
H	1.75334	-0.97359	2.87650
H	0.74116	0.23483	3.69970
C	-0.31651	-3.56031	3.28350
H	-0.24315	-4.61664	2.96003
C	0.86221	-3.21935	4.22346
H	1.82576	-3.39072	3.70571
H	0.84888	-3.88194	5.11088
C	0.75175	-1.74013	4.65980
H	1.60074	-1.48385	5.32356
C	-0.58015	-1.51754	5.41271
H	-0.65349	-0.46587	5.75142
H	-0.61911	-2.15343	6.31859
C	-1.75643	-1.85788	4.46916
H	-2.71742	-1.69069	4.99251
C	-1.65257	-3.33851	4.03170
H	-1.70754	-4.00018	4.91845
H	-2.50221	-3.59908	3.37353
N	2.78880	-1.08367	-0.62463
N	3.26125	1.03804	1.23633
C	2.03335	1.07024	1.40800
C	4.46958	1.49425	1.79105
C	4.60646	1.67009	3.20282
C	5.57568	1.75325	0.93137
C	5.84617	2.09730	3.70866
C	6.79240	2.18522	1.48849
C	6.93765	2.35844	2.86906
H	5.94737	2.22946	4.79190
H	7.63760	2.37880	0.81800
C	5.46826	1.57079	-0.56313
H	4.87247	0.66952	-0.79278
H	5.00754	2.45366	-1.05358
H	6.46522	1.45484	-1.01865
C	3.46599	1.40441	4.15193
H	2.58130	2.01614	3.90915
H	3.13992	0.35190	4.10545
H	3.76694	1.62569	5.18842
H	7.89277	2.68910	3.28937

K

SCF (BP86) Energy = -2686.92095911
Enthalpy 0K = -2685.627686
Enthalpy 298K = -2685.551828
Free Energy 298K = -2685.736497
Lowest Frequency = 15.5827 cm^{-1}
Second Frequency = 17.5628 cm^{-1}
SCF (BP86-D3BJ) Energy = -2687.36667721

```
SCF (tol) Energy = -2686.93410979
SCF (BS2) Energy = -4070.88321280

Si -2.60104  1.91408  2.44572
Si -3.42916  2.32742 -0.55999
Al -1.18999  0.50875  0.22186
O  -2.57832  2.63570  0.89122
N  -1.76809  0.41155  2.05858
N  -2.57411  0.87713 -1.09332
N   3.54851 -0.53600 -1.31343
N   0.57816  1.35478 -0.13944
N   0.51789 -2.12975 -0.80118
C  -1.53944 -0.55213  3.10768
C  -0.36425 -0.49507  3.93183
C  -0.19939 -1.40986  4.99103
H   0.70542 -1.34568  5.60677
C  -1.15557 -2.38633  5.27830
H  -1.01350 -3.08276  6.11189
C  -2.29463 -2.45772  4.47350
H  -3.04869 -3.22611  4.67999
C  -2.50783 -1.57210  3.39791
C   0.76013  0.51369  3.70905
H   0.43539  1.18509  2.89635
C   2.05275 -0.19903  3.25283
H   2.37556 -0.94585  4.00035
H   2.87504  0.52741  3.12867
H   1.91113 -0.71397  2.28949
C   1.05360  1.36175  4.96800
H   0.14637  1.84676  5.36142
H   1.79176  2.15141  4.73832
H   1.47923  0.74430  5.77916
C  -3.77397 -1.79014  2.56983
H  -3.80899 -0.99611  1.80315
C  -3.71532 -3.15898  1.85660
H  -2.86731 -3.22166  1.15670
H  -4.64417 -3.34736  1.29335
H  -3.60157 -3.97749  2.58950
C  -5.06199 -1.71462  3.42314
H  -5.11498 -2.55224  4.14141
H  -5.95597 -1.78312  2.77759
H  -5.12741 -0.77920  4.00014
C  -1.72630  3.14510  3.59444
H  -0.67119  3.31153  3.32890
H  -1.76746  2.80005  4.64127
H  -2.25230  4.11468  3.54317
C  -4.35951  1.76751  3.14136
H  -4.81799  2.77031  3.19691
H  -4.31705  1.36709  4.16900
H  -5.01589  1.11616  2.54691
C  -3.23792  3.84718 -1.67803
H  -3.75631  4.70871 -1.22227
H  -3.70426  3.65824 -2.66039
H  -2.18621  4.12638 -1.84310
C  -5.29167  2.21844 -0.20570
H  -5.59559  1.29973  0.31666
H  -5.86465  2.28747 -1.14553
H  -5.57986  3.08346  0.41712
C  -2.86036  0.36706 -2.40902
C  -2.09004  0.76763 -3.55659
C  -2.44095  0.31335 -4.84422
H  -1.84269  0.64401 -5.70198
C  -3.53737 -0.52417 -5.05999
H  -3.80649 -0.85026 -6.07059
C  -4.28295 -0.93269 -3.95199
H  -5.14243 -1.59632 -4.10206
C  -3.97036 -0.51928 -2.64091
C  -0.85633  1.66538 -3.46583
H  -0.76060  1.99603 -2.41765
C   0.41389  0.85247 -3.81524
H   0.55593  0.07241 -3.04884
H   1.31521  1.48862 -3.81816
H   0.31576  0.38131 -4.81106
C  -0.94648  2.91056 -4.37620
H  -0.94838  2.63712 -5.44650
H  -0.07848  3.57385 -4.21013
H  -1.86112  3.49124 -4.17953
C  -4.84808 -1.08859 -1.52544
H  -4.49169 -0.66092 -0.57259
C  -4.70681 -2.62606 -1.45752
H  -4.99994 -3.08853 -2.41733
H  -5.36856 -3.04353 -0.67958
H  -3.67474 -2.93754 -1.23384
C  -6.34227 -0.73230 -1.71247
H  -6.50222  0.34712 -1.85472
H  -6.92863 -1.05304 -0.83310
H  -6.76605 -1.24887 -2.59233
C   2.55916 -0.13864 -0.62272
C   4.83148 -0.97552 -0.67928
C   1.24557  0.16592 -0.45179
C   1.31596  2.63511 -0.09066
C   2.37971  2.63859  1.04811
H   1.86929  2.44002  2.00644
H   3.10069  1.81418  0.89019
C   2.04404  2.95540 -1.43257
H   2.75665  2.14723 -1.67860
H   1.29225  2.99038 -2.24062
C   0.32443  3.78932  0.18581
H  -0.22055  3.59342  1.12370
H  -0.43717  3.81044 -0.61407
C   3.12435  3.99331  1.11864
H   3.87074  3.95519  1.93565
C   3.83756  4.26812 -0.22479
H   4.38825  5.22755 -0.17677
H   4.58535  3.47668 -0.42831
C   2.78867  4.31088 -1.35930
H   3.29423  4.49776 -2.32659
C   1.76882  5.43834 -1.07821
H   2.28213  6.41871 -1.03787
H   1.02903  5.49448 -1.90005
C   1.05450  5.15189  0.26262
H   0.30997  5.94494  0.46517
C   2.10189  5.11928  1.39994
H   1.60308  4.94385  2.37238
H   2.61787  6.09647  1.47310
C   0.20595 -0.93762 -0.37866
C  -0.31950 -3.27334 -0.72692
C  -1.03797 -3.73083 -1.86696
C  -0.25288 -4.08075  0.44793
C  -1.09661 -2.92946 -3.15200
H  -2.05992 -3.07422 -3.66610
H  -0.32179 -3.25491 -3.88287
H  -0.99631 -1.84601 -2.98569
C   0.53689 -3.62118  1.65106
H   1.54685 -3.28200  1.35813
H   0.63709 -4.43434  2.38770
H   0.05518 -2.76832  2.16008
C   5.21421 -2.35444 -1.27863
H   4.43808 -3.09942 -1.00708
H   5.24468 -2.27539 -2.38616
C   5.92141  0.05318 -1.07812
H   5.64511  1.04323 -0.66900
H   5.94220  0.15355 -2.18042
C   4.78253 -1.09779  0.85823
H   3.99371 -1.81803  1.14621
H   4.49027 -0.12445  1.29271
C   6.59323 -2.81220 -0.74401
```

H 6.84572 -3.79693 -1.18137
C 6.52824 -2.92444 0.79821
H 7.50426 -3.26626 1.19212
H 5.77784 -3.68253 1.09393
C 6.16081 -1.54727 1.40099
H 6.10965 -1.62670 2.50301
C 7.23681 -0.50826 1.00286
H 8.22374 -0.81020 1.40265
H 6.99666 0.47642 1.44684
C 7.30092 -0.39397 -0.53931
H 8.06414 0.35314 -0.82799
C 7.66620 -1.77109 -1.14346
H 7.73580 -1.69796 -2.24626
H 8.65942 -2.09576 -0.77953
C -1.69255 -4.97782 -1.80595
H -2.26167 -5.31472 -2.68076
C -1.63430 -5.77663 -0.65823
H -2.14852 -6.74263 -0.62899
C -0.91312 -5.32176 0.45583
H -0.85869 -5.93718 1.36135
K 2.12063 -2.08622 -3.00117

4·THF

SCF (BP86) Energy = -3151.84905112
Enthalpy 0K = -3150.326306
Enthalpy 298K = -3150.236319
Free Energy 298K = -3150.458199
Lowest Frequency = 12.2235 cm^{-1}
Second Frequency = 13.4223 cm^{-1}
SCF (BP86-D3BJ) Energy = -3152.35157957
SCF (tol) Energy = -3151.85720205
SCF (BS2) Energy = -4535.93075430

Si -4.54525 1.45901 0.87063
Si -4.43274 -0.41221 -1.64737
Al -1.97135 0.21660 -0.04500
O -4.39382 1.06443 -0.78819
N -3.01227 0.82357 1.46025
N -2.86030 -1.07976 -1.19307
N 2.87504 1.52497 -0.88271
N -0.83586 1.55794 -0.97171
N 1.01498 -0.88703 0.82461
C -2.70291 0.93240 2.86432
C -2.06161 2.10057 3.40121
C -1.83053 2.20535 4.78760
H -1.34100 3.10726 5.17366
C -2.21093 1.19815 5.67732
H -2.03078 1.30215 6.75296
C -2.82104 0.05224 5.16217
H -3.11452 -0.75197 5.84698
C -3.07191 -0.10670 3.78439
C -1.58228 3.25985 2.53072
H -1.90781 3.04504 1.49912
C -0.03975 3.34676 2.53508
H 0.34481 3.47736 3.56272
H 0.30206 4.21043 1.93756
H 0.41651 2.43974 2.10781
C -2.17869 4.61719 2.96875
H -3.27768 4.58481 3.03466
H -1.90226 5.40993 2.25027
H -1.79497 4.92568 3.95774
C -3.70366 -1.42833 3.34807
H -3.87436 -1.36895 2.25875
C -2.73665 -2.59919 3.63052
H -1.79681 -2.49979 3.06477
H -3.20038 -3.56359 3.36585
H -2.47479 -2.64035 4.70277
C -5.05580 -1.70099 4.04783
H -4.92028 -1.86086 5.13253
H -5.52208 -2.61600 3.64035
H -5.76651 -0.86949 3.92156
C -4.80859 3.33792 0.91742
H -3.94958 3.90525 0.52742
H -5.00996 3.68105 1.94607
H -5.69288 3.58682 0.30461
C -6.11641 0.72735 1.64242
H -7.00387 1.11233 1.11042
H -6.19561 1.04998 2.69501
H -6.14465 -0.37143 1.61793
C -4.63266 -0.00868 -3.48927
H -5.62914 0.43142 -3.66795
H -4.56030 -0.93111 -4.09097
H -3.87276 0.70007 -3.85156
C -6.01494 -1.36027 -1.18852
H -5.99455 -1.81822 -0.18889
H -6.21847 -2.15264 -1.92785
H -6.86137 -0.65211 -1.22730
C -2.40984 -2.26122 -1.88333
C -1.56225 -2.18137 -3.04370
C -1.20536 -3.35077 -3.74592
H -0.57334 -3.25638 -4.63738
C -1.65246 -4.61534 -3.35626
H -1.38283 -5.50990 -3.92866
C -2.45692 -4.70774 -2.21859
H -2.80786 -5.69328 -1.89102
C -2.83673 -3.57375 -1.47234
C -0.97978 -0.87019 -3.56959
H -1.38237 -0.05157 -2.94917
C 0.55901 -0.86794 -3.40765
H 0.82538 -0.98287 -2.34402
H 0.99970 0.07789 -3.76503
H 1.01606 -1.69680 -3.97819
C -1.34837 -0.60458 -5.04699
H -0.89924 -1.35646 -5.72071
H -0.97499 0.38458 -5.36825
H -2.43798 -0.62486 -5.20513
C -3.67088 -3.84140 -0.21832
H -3.91058 -2.86472 0.23539
C -2.85977 -4.66950 0.80417
H -2.55215 -5.63682 0.36724
H -3.47135 -4.89342 1.69541
H -1.95158 -4.14258 1.13532
C -4.98730 -4.59139 -0.53269
H -5.57073 -4.10121 -1.32654
H -5.62117 -4.65458 0.36968
H -4.78525 -5.62666 -0.86147
C 1.65442 1.54129 -0.54065
C 3.88801 2.41541 -0.22960
C 0.38616 1.06234 -0.49101
C -0.87730 2.78654 -1.79118
C -0.42664 4.03493 -0.97359
H -1.07632 4.12656 -0.08586
H 0.60577 3.88655 -0.60394
C 0.02361 2.69307 -3.06128
H 1.07255 2.50565 -2.76952
H -0.30799 1.83112 -3.66606
C -2.32738 3.03392 -2.26909
H -3.00105 3.09197 -1.39840
H -2.66358 2.16414 -2.86142
C -0.51106 5.32606 -1.82260
H -0.18164 6.18721 -1.20911
C 0.39800 5.19538 -3.06554
H 0.35560 6.12245 -3.66989
H 1.45256 5.06131 -2.75472
C -0.06321 3.98555 -3.91006

```
H   0.59163  3.87956 -4.79670
C  -1.52478  4.20193 -4.36462
H  -1.60226  5.11513 -4.98644
H  -1.85638  3.35327 -4.99362
C  -2.42952  4.32463 -3.11712
H  -3.48141  4.46320 -3.43158
C  -1.97447  5.53960 -2.27520
H  -2.63102  5.65820 -1.39186
H  -2.06044  6.47050 -2.86918
C   0.05211 -0.15166  0.34827
C   0.85351 -2.00783  1.67290
C   0.79573 -3.33220  1.14959
C   0.96294 -1.80070  3.08205
C   0.73145 -3.59662 -0.34015
H   0.26089 -4.57176 -0.54313
H   1.74006 -3.63686 -0.80214
H   0.14243 -2.83999 -0.87937
C   1.03540 -0.39939  3.64073
H   1.77077  0.21187  3.08699
H   1.31095 -0.41337  4.70785
H   0.06746  0.12478  3.55395
O   3.91790 -3.36130 -2.39760
C   3.21429 -3.99202 -3.50997
H   2.13384 -3.94278 -3.28751
H   3.40854 -3.41987 -4.43618
C   3.73613 -5.44562 -3.60018
H   4.47272 -5.54335 -4.41624
H   2.92609 -6.16696 -3.79116
C   4.42429 -5.65886 -2.23418
H   5.21333 -6.42771 -2.26380
H   3.68761 -5.94466 -1.46338
C   4.96517 -4.25578 -1.95002
H   5.14799 -4.02962 -0.88636
H   5.89968 -4.06896 -2.52189
C   5.18311  1.59209 -0.01188
H   4.97692  0.76290  0.69598
H   5.49624  1.14572 -0.97793
C   4.20035  3.56478 -1.22354
H   3.27509  4.14608 -1.39602
H   4.49755  3.13237 -2.19793
C   3.44576  3.01766  1.12111
H   3.20709  2.19990  1.82815
H   2.51134  3.58956  0.97806
C   6.30783  2.49270  0.55508
H   7.22237  1.88677  0.70528
C   5.84803  3.09023  1.90671
H   6.64960  3.72363  2.33250
H   5.65587  2.27946  2.63642
C   4.56297  3.92539  1.69158
H   4.23061  4.35008  2.65749
C   4.85805  5.07100  0.69332
H   5.64205  5.73800  1.10047
H   3.95215  5.68926  0.54744
C   5.31703  4.47429 -0.65885
H   5.52105  5.29010 -1.37792
C   6.60049  3.63708 -0.44414
H   6.95047  3.22108 -1.40870
H   7.41418  4.27858 -0.05531
C   0.83091 -4.41984  2.04720
H   0.76282 -5.43578  1.63970
C   0.93985 -4.22644  3.43053
H   0.96264 -5.08473  4.10992
C   1.00810 -2.91839  3.93429
H   1.08574 -2.75029  5.01497
K   3.26750 -1.24673 -0.79919
O   5.01329 -2.35031  1.05730
C   4.52616 -3.15544  2.16295
H   4.93023 -4.18546  2.06721
H   3.42802 -3.19211  2.10106
C   5.06515 -2.46369  3.43073
H   5.27888 -3.19168  4.22942
H   4.32170 -1.75029  3.81887
C   6.34487 -1.72302  2.93388
H   6.26778 -0.63971  3.11974
H   7.26197 -2.08095  3.42915
C   6.37320 -2.02099  1.41643
H   6.68689 -1.16708  0.79442
H   7.03511 -2.88477  1.19380
```

L

SCF (BP86) Energy = -2686.87719838
Enthalpy 0K = -2685.587316
Enthalpy 298K = -2685.509311
Free Energy 298K = -2685.707463
Lowest Frequency = 2.6922 cm^{-1}
Second Frequency = 4.8979 cm^{-1}
SCF (BP86-D3BJ) Energy = -2687.30910900
SCF (tol) Energy = -2686.88842451
SCF (BS2) Energy = -4070.84104751

```
K  -3.62622 -0.77744 -0.13145
Si  2.68738  2.59649 -1.53107
Si  2.89345  2.37462  1.64335
Al  1.26545  0.59814  0.02633
O   2.21080  2.80299  0.11458
N   2.22050  0.91111 -1.64197
N   2.23551  0.74818  1.70141
N  -7.66101 -1.18954  1.20567
N   0.35743 -1.19191 -0.06958
N  -1.96748  1.35625 -0.01823
C   2.30487  0.04849 -2.78178
C   1.17883 -0.14508 -3.65285
C   1.25877 -1.07968 -4.70445
H   0.38902 -1.21602 -5.35832
C   2.41913 -1.82132 -4.94682
H   2.45872 -2.54657 -5.76680
C   3.53773 -1.59567 -4.13883
H   4.46585 -2.14251 -4.34304
C   3.51256 -0.67464 -3.07287
C  -0.09602  0.69436 -3.54855
H  -0.02543  1.28534 -2.61900
C  -1.38427 -0.15315 -3.46706
H  -2.26228  0.51067 -3.35778
H  -1.35517 -0.83591 -2.60114
H  -1.53454 -0.75533 -4.38167
C  -0.19417  1.67240 -4.74402
H   0.71051  2.29442 -4.83655
H  -1.06381  2.34558 -4.63325
H  -0.31484  1.12103 -5.69398
C   4.82106 -0.43340 -2.31684
H   4.58719  0.24136 -1.47587
C   5.44534 -1.71976 -1.73467
H   4.76292 -2.22556 -1.03533
H   6.37531 -1.48086 -1.18878
H   5.70960 -2.43947 -2.52997
C   5.85377  0.25660 -3.24146
H   6.16267 -0.42417 -4.05493
H   6.76238  0.53896 -2.67952
H   5.44392  1.16462 -3.71152
C   1.67199  3.85294 -2.52608
H   0.58901  3.68207 -2.42759
H   1.92964  3.80953 -3.59806
H   1.89798  4.87393 -2.17108
C   4.49275  3.11524 -1.80987
H   4.62599  4.14188 -1.42498
```

```
H   4.71109  3.14383 -2.89101
H   5.23597  2.46481 -1.32789
C   2.24058  3.59974  2.93222
H   2.65432  4.60509  2.74035
H   2.55400  3.29850  3.94689
H   1.14222  3.66602  2.91608
C   4.77326  2.64555  1.57645
H   5.29804  1.92097  0.93541
H   5.21228  2.58726  2.58549
H   4.97269  3.65999  1.18920
C   2.20394 -0.14124  2.82580
C   3.34212 -0.94870  3.17363
C   3.25172 -1.87358  4.23276
H   4.13050 -2.48015  4.48060
C   2.08214 -2.02832  4.98216
H   2.03214 -2.75681  5.79864
C   0.98704 -1.21395  4.68175
H   0.07570 -1.29789  5.28567
C   1.02168 -0.27264  3.63349
C   4.70474 -0.79061  2.49736
H   4.55540 -0.14917  1.61246
C   5.32462 -2.12415  2.02529
H   5.55261 -2.79077  2.87591
H   6.27478 -1.93810  1.49464
H   4.65672 -2.67116  1.34177
C   5.69473 -0.09327  3.46339
H   5.27736  0.83822  3.87887
H   6.64542  0.14877  2.95532
H   5.92791 -0.75392  4.31778
C  -0.20536  0.62496  3.46395
H  -0.05568  1.22099  2.54727
C  -1.51735 -0.17335  3.29770
H  -1.45412 -0.86625  2.44173
H  -2.36112  0.51966  3.12237
H  -1.75265 -0.76063  4.20386
C  -0.34037  1.59859  4.65918
H  -0.53362  1.04780  5.59735
H  -1.18196  2.29814  4.50282
H   0.57547  2.19257  4.80647
C  -8.26868 -0.60713  2.05515
C  -0.90160 -0.77641 -0.09216
C   0.66486 -2.64685 -0.09903
C   2.19307 -2.83240 -0.16994
H   2.65103 -2.32547  0.69968
H   2.56866 -2.33893 -1.08507
C   0.02381 -3.33119 -1.33494
H   0.38852 -2.83667 -2.25466
H  -1.07134 -3.18100 -1.29138
C   0.13249 -3.33189  1.18811
H  -0.96332 -3.18193  1.23425
H   0.56933 -2.83718  2.07533
C   2.55556 -4.33633 -0.17997
H   3.65599 -4.44809 -0.22813
C   1.91314 -5.00620 -1.41806
H   2.17768 -6.08114 -1.45158
H   2.30332 -4.54327 -2.34399
C   0.37585 -4.83778 -1.35090
H  -0.08808 -5.31070 -2.23841
C  -0.15873 -5.50893 -0.06309
H   0.07168 -6.59208 -0.07337
H  -1.26220 -5.41456 -0.01535
C   0.48402 -4.83809  1.17431
H   0.09811 -5.31046  2.09837
C   2.02155 -5.00414  1.10962
H   2.48715 -4.53949  1.99912
H   2.29012 -6.07850  1.12234
C  -0.81067  0.75275 -0.01178
C  -2.15801  2.81437  0.07214
C  -3.40790  3.05030  0.96967
H  -4.25773  2.46423  0.56206
H  -3.20172  2.66032  1.98534
C  -2.49090  3.34644 -1.35165
H  -3.33182  2.75807 -1.77081
H  -1.61997  3.17494 -2.01141
C  -0.98292  3.63216  0.65289
H  -0.06925  3.47325  0.05788
H  -0.75970  3.25850  1.67054
C  -3.77671  4.55051  1.02983
H  -4.66492  4.68399  1.67675
C  -4.09147  5.05464 -0.39871
H  -4.36971  6.12567 -0.37177
H  -4.96066  4.50681 -0.81329
C  -2.85183  4.85023 -1.30155
H  -3.07470  5.20467 -2.32632
C  -1.65840  5.64605 -0.72102
H  -0.77110  5.52840 -1.37078
H  -1.90054  6.72627 -0.69664
C  -1.34382  5.13767  0.70630
H  -0.48517  5.70106  1.11800
C  -2.58281  5.34678  1.60793
H  -2.36488  5.00818  2.63912
H  -2.83750  6.42263  1.66848
C  -6.94301 -1.85579  0.22173
C  -6.96813 -1.34238 -1.10422
C  -6.21482 -3.02274  0.58225
C  -6.22037 -2.03052 -2.07955
C  -5.48523 -3.67029 -0.43384
C  -5.48596 -3.18281 -1.75180
H  -6.22900 -1.66166 -3.11076
H  -4.92023 -4.57480 -0.18547
C  -7.78386 -0.11703 -1.44036
H  -8.85731 -0.29406 -1.25201
H  -7.49834  0.74900 -0.81805
H  -7.66078  0.15921 -2.49855
C  -6.24326 -3.54185  1.99981
H  -5.90096 -2.78003  2.72207
H  -7.27015 -3.81485  2.29976
H  -5.60440 -4.43158  2.10553
H  -4.91834 -3.70704 -2.52700
```

TS(L-M)

SCF (BP86) Energy = -2686.85510104
Enthalpy 0K = -2685.564478
Enthalpy 298K = -2685.488468
Free Energy 298K = -2685.674019
Lowest Frequency = -177.2247 cm^{-1}
Second Frequency = 12.5955 cm^{-1}
SCF (BP86-D3BJ) Energy = -2687.29851294
SCF (tol) Energy = -2686.86541468
SCF (BS2) Energy = -4070.81653880

```
K  -3.68776 -0.67450 -1.46475
Si  2.30682  2.61120 -1.66898
Si  2.59387  2.58257  1.46599
Al  0.97281  0.57191  0.03063
O   1.87428  2.96823 -0.05081
N   1.95252  0.88703 -1.63719
N   1.92707  0.96483  1.66376
N  -3.64967 -2.21303  0.93836
N   0.12141 -1.26086 -0.00277
N  -2.28021  1.23804 -0.19539
C   2.21071  0.00887 -2.74422
C   1.19512 -0.28654 -3.71639
C   1.44660 -1.21655 -4.74465
H   0.65649 -1.42865 -5.47522
```

C	2.68143	-1.85728	-4.87248
H	2.85719	-2.58168	-5.67485
C	3.69680	-1.52838	-3.97049
H	4.68200	-1.99620	-4.08125
C	3.49913	-0.60629	-2.92355
C	-0.15351	0.43283	-3.74212
H	-0.20921	1.06213	-2.83720
C	-1.34363	-0.54959	-3.71171
H	-2.29439	0.01871	-3.76847
H	-1.31334	-1.16235	-2.79310
H	-1.33253	-1.23483	-4.57759
C	-0.27075	1.34058	-4.98937
H	0.57818	2.03759	-5.06344
H	-1.20095	1.93729	-4.96000
H	-0.28568	0.73872	-5.91559
C	4.71463	-0.27690	-2.05443
H	4.37802	0.43315	-1.27980
C	5.30134	-1.51581	-1.34490
H	4.56095	-2.00524	-0.69657
H	6.16507	-1.22706	-0.72033
H	5.66012	-2.26393	-2.07424
C	5.83114	0.38598	-2.89851
H	6.26044	-0.33745	-3.61448
H	6.65491	0.73694	-2.25114
H	5.46131	1.24431	-3.47987
C	1.19516	3.70988	-2.74858
H	0.12948	3.44678	-2.67224
H	1.48923	3.65062	-3.81002
H	1.31592	4.76053	-2.43054
C	4.05822	3.21390	-2.08331
H	4.10326	4.29439	-1.85734
H	4.25212	3.10111	-3.16356
H	4.86492	2.71232	-1.53235
C	2.02872	3.91212	2.68912
H	2.45787	4.88383	2.38798
H	2.38555	3.69238	3.70959
H	0.93306	4.01021	2.71866
C	4.47880	2.78378	1.31707
H	4.95200	2.00075	0.70429
H	4.95464	2.76872	2.31071
H	4.69989	3.76443	0.86098
C	1.91031	0.21030	2.89291
C	3.02662	-0.60817	3.28070
C	2.93664	-1.42044	4.42882
H	3.79540	-2.04294	4.70493
C	1.79305	-1.44116	5.23145
H	1.73891	-2.08971	6.11253
C	0.73505	-0.59086	4.90348
H	-0.14989	-0.56119	5.54945
C	0.77298	0.24540	3.77001
C	4.37811	-0.56048	2.56625
H	4.23036	-0.01285	1.61970
C	4.96148	-1.95074	2.23739
H	5.16781	-2.53291	3.15286
H	5.91780	-1.84924	1.69588
H	4.27943	-2.54639	1.61082
C	5.39995	0.21120	3.43934
H	5.00809	1.18791	3.76594
H	6.34519	0.38060	2.89304
H	5.63586	-0.36471	4.35218
C	-0.39290	1.21853	3.59106
H	-0.24696	1.72900	2.62369
C	-1.76723	0.51870	3.56347
H	-1.81994	-0.27206	2.79729
H	-2.56573	1.25560	3.36006
H	-1.99865	0.05480	4.53984
C	-0.38848	2.28100	4.71811
H	-0.59901	1.81130	5.69570

H	-1.17044	3.04212	4.54073
H	0.58040	2.79719	4.80135
C	-2.51660	-1.84899	1.22451
C	-1.15169	-0.87917	-0.12515
C	0.52203	-2.68860	0.02433
C	2.00107	-2.81359	-0.39846
H	2.61118	-2.18804	0.28060
H	2.13413	-2.41476	-1.42018
C	-0.33825	-3.54609	-0.93953
H	-0.22855	-3.14820	-1.96713
H	-1.40056	-3.44951	-0.65380
C	0.38226	-3.23781	1.47476
H	-0.66163	-3.11860	1.81267
H	1.01279	-2.63200	2.15072
C	2.45977	-4.29216	-0.33906
H	3.52153	-4.35287	-0.64488
C	1.59267	-5.13007	-1.30885
H	1.91926	-6.18791	-1.29509
H	1.71909	-4.76279	-2.34515
C	0.10810	-5.02625	-0.88487
H	-0.51853	-5.62080	-1.57813
C	-0.06155	-5.55824	0.55826
H	0.22982	-6.62547	0.60574
H	-1.12448	-5.49937	0.86244
C	0.81362	-4.72354	1.52240
H	0.69007	-5.09837	2.55610
C	2.29486	-4.84237	1.09744
H	2.93260	-4.27863	1.80297
H	2.62371	-5.89928	1.13749
C	-1.12119	0.65382	-0.06868
C	-2.49403	2.69857	-0.17283
C	-3.74928	2.95663	0.71081
H	-4.58760	2.33767	0.33443
H	-3.53755	2.61919	1.74266
C	-2.84163	3.15333	-1.62088
H	-3.68467	2.53959	-2.00418
H	-1.97489	2.96191	-2.28106
C	-1.33477	3.56759	0.36079
H	-0.41772	3.40117	-0.22545
H	-1.10085	3.25241	1.39491
C	-4.14340	4.45158	0.69681
H	-5.03319	4.59882	1.33833
C	-4.47147	4.87793	-0.75372
H	-4.76827	5.94378	-0.78121
H	-5.33180	4.29453	-1.13741
C	-3.22993	4.65145	-1.64818
H	-3.46032	4.94687	-2.68983
C	-2.05097	5.49765	-1.11162
H	-1.16334	5.36688	-1.75768
H	-2.31670	6.57204	-1.14037
C	-1.72445	5.06700	0.33814
H	-0.87450	5.66452	0.71775
C	-2.96487	5.29848	1.23174
H	-2.73887	5.01579	2.27768
H	-3.23943	6.37107	1.23884
C	-4.93006	-2.47964	0.52268
C	-5.95743	-1.52024	0.80866
C	-5.22810	-3.69190	-0.17879
C	-7.25153	-1.77060	0.32275
C	-6.54474	-3.88890	-0.62707
C	-7.55311	-2.93806	-0.39834
H	-8.04090	-1.04140	0.53896
H	-6.77996	-4.81614	-1.16218
C	-5.65082	-0.31880	1.67247
H	-5.45255	-0.63416	2.71361
H	-4.74696	0.22641	1.34977
H	-6.49840	0.38468	1.68558
C	-4.16126	-4.74039	-0.38553

H -3.30214 -4.36515 -0.97028
H -3.74691 -5.07974 0.58068
H -4.57080 -5.61799 -0.91004
H -8.57022 -3.11507 -0.76089

M

SCF (BP86) Energy = -2686.91742438
Enthalpy 0K = -2685.624253
Enthalpy 298K = -2685.548428
Free Energy 298K = -2685.732673
Lowest Frequency = 12.3787 cm^{-1}
Second Frequency = 20.0505 cm^{-1}
SCF (BP86-D3BJ) Energy = -2687.36821733
SCF (tol) Energy = -2686.92874402
SCF (BS2) Energy = -4070.87871909

K   4.26329 0.56605 -1.37158
Si -1.87593 -2.74268 -1.87617
Si -2.38851 -2.80850 1.22270
Al -0.88278 -0.58755 -0.05295
O -1.54139 -3.12830 -0.24087
N -1.68972 -0.99772 -1.76939
N -1.90800 -1.13101 1.48425
N   3.10529 2.24646 0.96276
N -0.25428 1.25700 0.01775
N   2.45686 -1.01427 -0.06312
C -1.94278 -0.04989 -2.81686
C -0.88540 0.41299 -3.67049
C -1.13386 1.42918 -4.61411
H -0.31377 1.77162 -5.25672
C -2.40181 1.99734 -4.76694
H -2.57418 2.79327 -5.49922
C -3.45248 1.50221 -3.98929
H -4.46108 1.90730 -4.13208
C -3.25961 0.48504 -3.03315
C   0.50069 -0.23301 -3.67676
H   0.54348 -0.92771 -2.82060
C   1.64786 0.78811 -3.51752
H   2.61931 0.26136 -3.62359
H   1.60434 1.29958 -2.54000
H   1.62556 1.55784 -4.30904
C   0.70677 -1.04091 -4.98106
H -0.10840 -1.76386 -5.14207
H   1.65987 -1.60116 -4.95655
H   0.73125 -0.37085 -5.85909
C -4.50526 -0.05674 -2.32740
H -4.16030 -0.79494 -1.58329
C -5.31685 1.02520 -1.58397
H -4.72150 1.52174 -0.80446
H -6.20102 0.57367 -1.10023
H -5.68512 1.80275 -2.27680
C -5.43274 -0.76259 -3.34772
H -5.86580 -0.02866 -4.05069
H -6.27217 -1.26552 -2.83450
H -4.89553 -1.51422 -3.94658
C -0.57984 -3.68636 -2.89622
H   0.44619 -3.33630 -2.70713
H -0.77971 -3.59102 -3.97683
H -0.63190 -4.76003 -2.64316
C -3.52450 -3.48356 -2.45748
H -3.49245 -4.57124 -2.26699
H -3.63702 -3.34866 -3.54679
H -4.41679 -3.07366 -1.96542
C -1.76571 -4.03563 2.52308
H -2.12423 -5.05234 2.28660
H -2.14650 -3.76874 3.52395
H -0.66590 -4.05533 2.56781
C -4.21798 -3.23165 0.92015
H -4.72923 -2.50523 0.26923
H -4.77958 -3.29483 1.86498
H -4.26860 -4.22333 0.43758
C -2.04790 -0.41962 2.73153
C -3.30447 0.15494 3.13252
C -3.38632 0.91261 4.31846
H -4.35341 1.34271 4.60280
C -2.27927 1.11969 5.14419
H -2.36409 1.71931 6.05684
C -1.06818 0.52147 4.79000
H -0.19832 0.64242 5.44576
C -0.92922 -0.24938 3.61845
C -4.61575 -0.08279 2.38122
H -4.36419 -0.56054 1.41949
C -5.40256 1.21291 2.08324
H -5.74329 1.70368 3.01197
H -6.30340 0.98611 1.48678
H -4.79900 1.94353 1.52307
C -5.52406 -1.03582 3.19851
H -4.99199 -1.95015 3.50793
H -6.41742 -1.33131 2.61963
H -5.87091 -0.53677 4.12111
C   0.42370 -0.92714 3.40221
H   0.40138 -1.39689 2.40418
C   1.60589 0.06427 3.43779
H   1.48384 0.88609 2.71624
H   2.54868 -0.45667 3.19495
H   1.72769 0.50953 4.44168
C   0.65381 -2.03748 4.45610
H   0.72830 -1.60586 5.47046
H   1.59790 -2.57714 4.25553
H -0.16603 -2.77268 4.46804
C   2.21138 1.80249 0.15461
C   1.09046 0.99389 0.13564
C -0.80985 2.60940 0.17456
C -2.34875 2.49570 0.11523
H -2.68467 1.81572 0.92070
H -2.62841 2.04215 -0.85342
C -0.35885 3.54451 -0.98136
H -0.65402 3.08884 -1.94509
H   0.74251 3.62576 -0.98081
C -0.41332 3.24659 1.53841
H   0.68583 3.33492 1.60928
H -0.74006 2.57627 2.35481
C -3.01022 3.88607 0.26812
H -4.11081 3.77324 0.22215
C -2.53930 4.80730 -0.88168
H -3.01575 5.80299 -0.79210
H -2.84524 4.38359 -1.85697
C -0.99962 4.94567 -0.82415
H -0.65368 5.59912 -1.64840
C -0.59117 5.56094 0.53591
H -1.03613 6.56933 0.64359
H   0.50790 5.69003 0.58411
C -1.06697 4.64234 1.68653
H -0.76981 5.07943 2.65887
C -2.60590 4.50035 1.62875
H -2.95616 3.85508 2.45637
H -3.08660 5.48966 1.75799
C   1.25426 -0.52397 0.03112
C   2.76848 -2.45878 -0.01765
C   3.95212 -2.62355 0.98189
H   4.78080 -1.94952 0.68071
H   3.62269 -2.29228 1.98430
C   3.27416 -2.90864 -1.41924
H   4.11692 -2.25638 -1.73983
H   2.46537 -2.77782 -2.16200

C   1.63001 -3.40334 0.42544
H   0.75948 -3.30819 -0.24155
H   1.28325 -3.09409 1.42879
C   4.44826 -4.08765 1.02529
H   5.28518 -4.16664 1.74502
C   4.93187 -4.50618 -0.38345
H   5.30259 -5.54865 -0.36606
H   5.78211 -3.86941 -0.70047
C   3.76268 -4.37727 -1.38827
H   4.10477 -4.66753 -2.40022
C   2.59977 -5.29581 -0.94426
H   1.76627 -5.23129 -1.66768
H   2.93837 -6.34970 -0.93311
C   2.11875 -4.87255 0.46356
H   1.27954 -5.52111 0.77705
C   3.28684 -5.00888 1.46710
H   2.94952 -4.73224 2.48417
H   3.63282 -6.05940 1.51602
C   4.35320 2.79130 0.56159
C   5.49992 2.23236 1.20867
C   4.51004 3.86192 -0.37054
C   6.78029 2.69900 0.85949
C   5.81376 4.30811 -0.67024
C   6.94624 3.72794 -0.08119
H   7.65487 2.26198 1.35579
H   5.93191 5.13948 -1.37573
C   5.32393 1.16248 2.26411
H   4.63843 1.51368 3.05427
H   4.85923 0.24253 1.86657
H   6.29207 0.89533 2.71867
C   3.32020 4.55888 -0.99314
H   2.79015 3.93066 -1.72918
H   2.57567 4.82865 -0.22519
H   3.63778 5.48265 -1.50412
H   7.94733 4.09482 -0.32975

I′

SCF (BP86) Energy = -2766.73137886
Enthalpy 0K = -2765.355373
Enthalpy 298K = -2765.278907
Free Energy 298K = -2765.465192
Lowest Frequency = 10.0967 cm^{-1}
Second Frequency = 14.5353 cm^{-1}
SCF (BP86-D3BJ) Energy = -2767.18704463
SCF (tol) Energy = -2766.74070260
SCF (BS2) Energy = -4150.70999803

K -2.25668 0.67897 -3.06189
Si 2.15186 3.23981 -0.25937
Si 1.50876 2.38447 2.63163
Al 0.40719 0.59710 0.28591
O   2.53357 2.71745 1.30348
N   0.80533 2.20257 -0.78101
N   0.57852 0.94928 2.17385
N -3.86280 -0.31978 -1.21301
N -0.31863 -0.95289 -0.50740
C   0.10527 2.60452 -1.95707
C -1.06596 3.44453 -1.88691
C -1.73706 3.81892 -3.07153
H -2.62039 4.46365 -2.99432
C -1.29243 3.41466 -4.33894
H -1.81386 3.74131 -5.24589
C -0.14425 2.61372 -4.42094
H   0.23013 2.31091 -5.40626
C   0.56305 2.20917 -3.26709
C -1.62695 3.95954 -0.56238
H -0.90281 3.66912 0.21728
C -1.79276 5.49699 -0.54930
H -2.59705 5.82287 -1.23341
H -0.86992 6.02131 -0.84268
H -2.07392 5.83740 0.46272
C -2.97638 3.29007 -0.22524
H -2.87369 2.19730 -0.14366
H -3.73661 3.52066 -0.99524
H -3.36148 3.66828 0.73761
C   1.80474 1.33831 -3.46071
H   2.30786 1.28928 -2.48208
C   2.78852 1.92250 -4.49783
H   3.03622 2.97500 -4.28324
H   2.37882 1.88018 -5.52312
H   3.72865 1.34344 -4.50227
C   1.41468 -0.10510 -3.84456
H   0.84855 -0.12865 -4.79586
H   0.80433 -0.56139 -3.04708
H   2.31195 -0.73490 -3.98344
C   1.74212 5.09328 -0.30081
H   2.65246 5.66531 -0.05040
H   0.95678 5.37074 0.41737
H   1.41807 5.40444 -1.30876
C   3.79767 3.09737 -1.20732
H   4.14491 2.05706 -1.30063
H   4.56207 3.66499 -0.64757
H   3.73295 3.53394 -2.21743
C   0.52602 3.97591 2.99161
H -0.25153 4.18938 2.24090
H   1.22916 4.82718 3.01438
H   0.03431 3.92529 3.97629
C   2.65450 2.13822 4.11958
H   3.06399 3.11574 4.42748
H   3.49852 1.47608 3.87206
H   2.11280 1.70957 4.97868
C   0.00924 0.16396 3.24764
C -1.27999 0.47280 3.79748
C -1.81587 -0.32400 4.82918
H -2.80746 -0.07545 5.22517
C -1.12069 -1.41075 5.36229
H -1.55731 -2.01898 6.16190
C   0.15125 -1.69544 4.86083
H   0.71755 -2.53419 5.28296
C   0.73496 -0.93204 3.83033
C -2.12502 1.65973 3.33726
H -1.55453 2.18502 2.55342
C -2.40490 2.64134 4.50052
H -1.48407 2.93042 5.03369
H -3.08396 2.19002 5.24602
H -2.89076 3.56067 4.12747
C -3.45952 1.19301 2.72061
H -4.05880 0.62267 3.45357
H -3.27389 0.55118 1.84403
H -4.06594 2.05977 2.40130
C   2.14266 -1.34043 3.39104
H   2.47828 -0.60130 2.64375
C   3.15231 -1.35237 4.56339
H   4.17336 -1.55720 4.19140
H   2.90966 -2.14460 5.29373
H   3.17210 -0.39515 5.10598
C   2.12565 -2.73448 2.72891
H   1.45758 -2.74999 1.85507
H   1.76672 -3.50264 3.43697
H   3.13637 -3.03753 2.40025
C -2.77852 -0.39871 -0.51216
C -5.18963 -0.71318 -0.67299
C -5.98400 -1.36657 -1.83433
H -6.02289 -0.65797 -2.68605
H -5.43827 -2.26465 -2.18483

C	-5.12608	-1.71811	0.50209
H	-4.54265	-1.27937	1.33126
H	-4.57995	-2.62357	0.17234
C	-5.95999	0.55400	-0.21303
H	-6.00195	1.27492	-1.05351
H	-5.39747	1.04056	0.60406
C	-7.41320	-1.74492	-1.37881
H	-7.95818	-2.21047	-2.22269
C	-7.32715	-2.74365	-0.19956
H	-8.34449	-3.03333	0.12827
H	-6.81631	-3.67102	-0.52374
C	-6.55272	-2.09303	0.97157
H	-6.48610	-2.80728	1.81471
C	-7.29686	-0.81624	1.43217
H	-8.31207	-1.07579	1.79050
H	-6.76201	-0.35185	2.28239
C	-7.38803	0.18345	0.25410
H	-7.91688	1.09892	0.58300
C	-8.15927	-0.46985	-0.91728
H	-9.18916	-0.72573	-0.60109
H	-8.24850	0.24441	-1.75903
C	-1.47956	-0.15302	-0.36205
C	-0.40552	-2.38893	-0.85318
C	-1.10658	-3.19082	0.28084
H	-0.55158	-3.03699	1.22366
H	-2.11864	-2.77658	0.44019
C	1.01452	-2.96745	-1.06594
H	1.61436	-2.80910	-0.15280
H	1.51730	-2.41226	-1.88094
C	-1.21303	-2.62223	-2.16928
H	-2.23583	-2.22314	-2.04555
H	-0.72001	-2.07008	-2.99657
C	-1.17741	-4.69685	-0.06576
H	-1.67531	-5.24102	0.75956
C	-1.97926	-4.89260	-1.37363
H	-3.01367	-4.52057	-1.24464
H	-2.05054	-5.96931	-1.62285
C	-1.28151	-4.12874	-2.52371
H	-1.85820	-4.25916	-3.46030
C	0.15097	-4.67832	-2.71491
H	0.65113	-4.15497	-3.55347
H	0.11557	-5.75285	-2.98000
C	0.95233	-4.47704	-1.40744
H	1.98197	-4.86297	-1.54190
C	0.25695	-5.24512	-0.25770
H	0.22704	-6.32779	-0.48969
H	0.83436	-5.13121	0.67944
C	2.50235	-0.09062	0.04250
N	3.60832	-0.47363	-0.12472
C	4.97113	-0.90301	-0.30932
C	5.91081	-0.00425	0.54231
C	5.35260	-0.78463	-1.81194
C	5.11645	-2.38176	0.14290
H	5.78386	1.04973	0.23836
H	5.61680	-0.07757	1.60469
C	7.37365	-0.46759	0.34005
H	5.22567	0.26278	-2.14074
H	4.66474	-1.40852	-2.41143
C	6.81914	-1.24221	-2.00227
H	4.82435	-2.46501	1.20451
H	4.42391	-3.01392	-0.44174
C	6.58255	-2.83517	-0.05616
H	8.03446	0.17744	0.94791
C	7.75321	-0.34458	-1.15533
C	7.51810	-1.94052	0.79197
H	7.08096	-1.15065	-3.07266
C	6.96345	-2.71517	-1.55082
H	6.67282	-3.88758	0.27030
H	8.80602	-0.64931	-1.30468
H	7.67218	0.70868	-1.48448
H	7.26813	-2.03667	1.86549
H	8.56693	-2.27171	0.67400
H	6.31429	-3.36842	-2.16436
H	8.00388	-3.05752	-1.70513

J’

SCF (BP86) Energy = -2766.75451006
Enthalpy 0K = -2765.379811
Enthalpy 298K = -2765.301862
Free Energy 298K = -2765.497513
Lowest Frequency = 4.1944 cm^{-1}
Second Frequency = 5.1651 cm^{-1}
SCF (BP86-D3BJ) Energy = -2767.18701365
SCF (tol) Energy = -2766.76189153
SCF (BS2) Energy = -4150.73406555

K	1.81032	0.68883	-1.08765
Si	-3.62311	3.24539	-1.50392
Si	-5.27298	0.62936	-1.35636
Al	-2.33754	0.62456	-0.17874
O	-4.95427	2.24109	-1.80683
N	-2.19218	2.22256	-1.18525
N	-4.15756	0.20029	-0.04103
N	-1.08505	0.03073	1.07389
C	-0.94084	2.60005	-1.76621
C	-0.54532	2.10417	-3.05807
C	0.71186	2.46470	-3.58780
H	0.99945	2.08309	-4.57456
C	1.58532	3.32240	-2.90204
H	2.54734	3.60737	-3.34254
C	1.19023	3.83394	-1.65779
H	1.85231	4.52844	-1.12659
C	-0.05297	3.49697	-1.07969
C	-1.45752	1.20826	-3.89642
H	-2.44206	1.20978	-3.39900
C	-1.64456	1.73870	-5.33595
H	-0.71392	1.65827	-5.92629
H	-1.95733	2.79573	-5.35017
H	-2.41389	1.14563	-5.85997
C	-0.94992	-0.25108	-3.93295
H	-0.86768	-0.67776	-2.92069
H	0.04289	-0.30988	-4.41733
H	-1.63897	-0.88578	-4.51826
C	-0.42805	4.13565	0.25779
H	-1.42905	3.75576	0.52049
C	-0.50615	5.67539	0.14541
H	-1.19516	5.99032	-0.65634
H	0.48259	6.11601	-0.07659
H	-0.85998	6.11525	1.09440
C	0.53721	3.72219	1.38901
H	1.57517	4.04063	1.17862
H	0.52494	2.62806	1.52801
H	0.23728	4.18469	2.34590
C	-3.36728	4.32578	-3.03835
H	-4.21362	5.02819	-3.12810
H	-3.33328	3.71918	-3.95650
H	-2.43826	4.91674	-2.97770
C	-4.08757	4.38539	-0.05812
H	-4.07982	3.85707	0.90781
H	-5.10236	4.78838	-0.21940
H	-3.39280	5.23909	0.01513
C	-5.03569	-0.40452	-2.93267
H	-3.97152	-0.48738	-3.20943
H	-5.57168	0.06765	-3.77411
H	-5.43087	-1.42630	-2.80632

```
C  -7.08177  0.51805 -0.81341
H  -7.73825  0.63116 -1.69292
H  -7.33293  1.31437 -0.09567
H  -7.30300 -0.45382 -0.34194
C  -4.61376 -0.57288  1.08848
C  -4.57739 -2.00314  1.06464
C  -4.92839 -2.72279  2.22452
H  -4.88955 -3.81827  2.20107
C  -5.32640 -2.07466  3.39868
H  -5.58188 -2.65192  4.29397
C  -5.42637 -0.67866  3.40054
H  -5.77973 -0.16578  4.30294
C  -5.10374  0.08650  2.26256
C  -4.23527 -2.78757 -0.20313
H  -3.90131 -2.05648 -0.95839
C  -5.49487 -3.50297 -0.74839
H  -6.32380 -2.79602 -0.92427
H  -5.85864 -4.26469 -0.03552
H  -5.27225 -4.01568 -1.70153
C  -3.08831 -3.79942 -0.00324
H  -3.32164 -4.53747  0.78510
H  -2.15214 -3.28299  0.26272
H  -2.91193 -4.36163 -0.93813
C  -5.34396  1.59740  2.30834
H  -5.20401  1.97121  1.28012
C  -6.78795  1.93590  2.74602
H  -6.97817  3.01850  2.63773
H  -6.96425  1.67644  3.80520
H  -7.53484  1.39260  2.14403
C  -4.33690  2.33795  3.21516
H  -3.30235  2.21993  2.85459
H  -4.37596  1.95252  4.25004
H  -4.56581  3.41857  3.25347
C   0.08079 -1.55287 -0.55922
C   1.35018 -3.38594 -1.47787
C   2.76142 -3.54364 -2.09324
H   2.84518 -2.88430 -2.97969
H   3.51671 -3.19828 -1.35985
C   1.25101 -4.29574 -0.22757
H   0.24765 -4.17380  0.22217
H   1.99037 -3.95867  0.52562
C   0.30083 -3.85125 -2.51874
H   0.36266 -3.19948 -3.41178
H  -0.70887 -3.71764 -2.08763
C   3.02008 -5.01755 -2.48679
H   4.03232 -5.11074 -2.92559
C   2.91547 -5.91083 -1.22679
H   3.11497 -6.96731 -1.49185
H   3.68279 -5.61362 -0.48559
C   1.50191 -5.77352 -0.61120
H   1.42678 -6.40791  0.29302
C   0.44395 -6.21995 -1.64906
H   0.60146 -7.28202 -1.92009
H  -0.57010 -6.14415 -1.21249
C   0.54654 -5.32825 -2.91000
H  -0.21221 -5.64459 -3.65159
C   1.96116 -5.46650 -3.52306
H   2.14580 -6.51587 -3.82461
H   2.04020 -4.84884 -4.43868
C  -0.73583 -0.57974 -0.16393
C  -0.53745 -0.42011  2.36524
C  -0.93862 -1.89043  2.67119
H  -2.04078 -1.96761  2.65606
H  -0.55249 -2.54698  1.86945
C  -1.10260  0.47428  3.49136
H  -2.20416  0.39370  3.48316
H  -0.84581  1.53163  3.28525
C   1.01714 -0.32821  2.38804
H   1.43188 -0.97343  1.59013
H   1.32045  0.71780  2.17699
C  -0.38188 -2.33810  4.04417
H  -0.68081 -3.38666  4.23545
C   1.16117 -2.23208  4.04066
H   1.58641 -2.89874  3.26583
H   1.57040 -2.56409  5.01483
C   1.57541 -0.76725  3.76436
H   2.68027 -0.68774  3.75522
C   0.99637  0.15067  4.86698
H   1.30391  1.19975  4.68860
H   1.40136 -0.14083  5.85563
C  -0.54695  0.04133  4.86980
H  -0.96420  0.70248  5.65341
C  -0.95839 -1.42368  5.15102
H  -0.58506 -1.74066  6.14459
H  -2.06117 -1.50896  5.17323
N   1.16405 -1.94940 -1.12899
N   5.81258  1.13967 -0.17039
C   4.67304  1.08791 -0.48831
C   7.19577  1.18851  0.22555
C   7.53882  2.62006  0.72349
H   7.33931  3.34529 -0.08640
H   6.87889  2.87862  1.57153
C   7.43779  0.16581  1.37067
H   7.16559 -0.84586  1.01891
H   6.77739  0.40988  2.22252
C   8.09278  0.83035 -0.99189
H   7.89652  1.54494 -1.81195
H   7.82483 -0.17748 -1.35777
C   9.02633  2.66487  1.14903
H   9.26218  3.68577  1.50154
C   9.26799  1.64704  2.28964
H  10.32376  1.68988  2.61612
H   8.64811  1.90432  3.16936
C   8.92579  0.22172  1.79296
H   9.08908 -0.50804  2.60703
C   9.81988 -0.13524  0.58119
H  10.88456 -0.12098  0.88010
H   9.59736 -1.16101  0.23105
C   9.57780  0.88317 -0.55876
H  10.20882  0.62765 -1.42975
C   9.92048  2.30873 -0.06304
H  10.98666  2.36173  0.22599
H   9.77084  3.04292 -0.87739
```

TS(I′-K′)

SCF (BP86) Energy = -2766.72222833
Enthalpy 0K = -2765.346594
Enthalpy 298K = -2765.271238
Free Energy 298K = -2765.454523
Lowest Frequency = -90.8213 cm^{-1}
Second Frequency = 8.6688 cm^{-1}
SCF (BP86-D3BJ) Energy = -2767.19184354
SCF (tol) Energy = -2766.73383484
SCF (BS2) Energy = -4150.70040786

```
K  -2.76478  0.69482 -2.69158
Si  2.70554  2.63271 -1.11546
Si  2.58991  2.12258  1.95064
Al  1.14439  0.28654  0.01954
O   2.15526  2.89833  0.47960
N   2.39699  0.90799 -1.31434
N   1.80296  0.54348  1.80294
N  -3.39537 -1.60299 -1.54096
N   0.24123 -1.31311 -0.42962
N  -1.65956  2.02696 -0.49419
```

C	2.95065	0.11915	-2.38330
C	2.21817	-0.11527	-3.59499
C	2.74748	-0.96084	-4.59019
H	2.17396	-1.12491	-5.51028
C	3.99003	-1.58254	-4.44508
H	4.37959	-2.24469	-5.22544
C	4.73395	-1.31859	-3.29275
H	5.72412	-1.77472	-3.17845
C	4.25575	-0.47447	-2.27078
C	0.89834	0.58990	-3.91412
H	0.60264	1.14583	-3.00591
C	-0.22786	-0.40468	-4.27798
H	-1.10074	0.13443	-4.70365
H	-0.53879	-1.00598	-3.40598
H	0.08678	-1.10684	-5.06789
C	1.09604	1.60313	-5.06777
H	1.92355	2.29873	-4.85623
H	0.18138	2.20239	-5.23759
H	1.33508	1.08448	-6.01298
C	5.20281	-0.20598	-1.10007
H	4.68330	0.48663	-0.41723
C	5.54943	-1.48287	-0.30576
H	4.65253	-1.95397	0.12052
H	6.23748	-1.24401	0.52454
H	6.05234	-2.22908	-0.94658
C	6.51780	0.45450	-1.58230
H	7.11990	-0.25550	-2.17687
H	7.13527	0.76525	-0.72023
H	6.33635	1.33990	-2.21087
C	1.64274	3.77834	-2.20192
H	0.60623	3.41570	-2.27816
H	2.05497	3.84956	-3.22241
H	1.62865	4.79721	-1.77671
C	4.47667	3.25834	-1.37495
H	4.47891	4.35262	-1.22539
H	4.80197	3.06765	-2.41224
H	5.21676	2.81590	-0.69399
C	1.92774	3.17009	3.38267
H	2.52508	4.09201	3.48713
H	2.00206	2.60865	4.32990
H	0.87411	3.45301	3.23620
C	4.48767	2.20503	2.07165
H	4.99842	1.50828	1.38902
H	4.83814	1.99353	3.09377
H	4.80333	3.23175	1.81603
C	1.47073	-0.27598	2.94524
C	2.44300	-1.14474	3.55253
C	2.05936	-2.00707	4.59921
H	2.81419	-2.66522	5.04406
C	0.75327	-2.03867	5.09284
H	0.47435	-2.72668	5.89808
C	-0.17909	-1.14989	4.55522
H	-1.19722	-1.13039	4.96098
C	0.15132	-0.25995	3.51351
C	3.92988	-1.12008	3.19272
H	4.03826	-0.52937	2.26696
C	4.53283	-2.52116	2.94783
H	4.51346	-3.13753	3.86357
H	5.58895	-2.43559	2.63861
H	3.99311	-3.07395	2.16310
C	4.72794	-0.42735	4.32622
H	4.30257	0.55518	4.58868
H	5.78545	-0.28409	4.04030
H	4.70680	-1.04382	5.24274
C	-0.93738	0.72955	3.09543
H	-0.52341	1.35393	2.28480
C	-2.19925	0.01818	2.56159
H	-1.98127	-0.59176	1.67018
H	-2.97187	0.75859	2.28389
H	-2.63985	-0.63760	3.33400
C	-1.32038	1.66015	4.27200
H	-1.79925	1.09135	5.08861
H	-2.04364	2.42869	3.94090
H	-0.44260	2.17380	4.69561
C	-2.26033	-1.45503	-0.92636
C	-4.60533	-1.98830	-0.76305
C	-4.30069	-2.93446	0.42409
H	-3.59021	-2.43806	1.11017
H	-3.79615	-3.84052	0.03688
C	-5.58106	-2.70276	-1.73059
H	-5.08533	-3.60202	-2.14229
H	-5.79412	-2.03445	-2.58901
C	-5.30853	-0.71591	-0.21145
H	-5.53977	-0.03286	-1.05775
H	-4.60993	-0.18627	0.46409
C	-5.60372	-3.31790	1.16444
H	-5.36284	-3.99363	2.00710
C	-6.56162	-4.03174	0.18079
H	-7.49313	-4.32838	0.70062
H	-6.09337	-4.96050	-0.19764
C	-6.88979	-3.08502	-0.99923
H	-7.57111	-3.59551	-1.70650
C	-7.57092	-1.80546	-0.45630
H	-7.83377	-1.12841	-1.29293
H	-8.51749	-2.06514	0.05563
C	-6.61562	-1.08932	0.52827
H	-7.09720	-0.16991	0.91442
C	-6.28324	-2.03686	1.70534
H	-7.20962	-2.29772	2.25252
H	-5.61451	-1.52764	2.42524
C	-1.05521	-0.98649	-0.74571
C	0.61966	-2.74976	-0.38972
C	2.12534	-2.84773	-0.07794
H	2.32316	-2.34406	0.88900
H	2.68936	-2.31150	-0.86467
C	0.35992	-3.44005	-1.75683
H	0.92318	-2.90054	-2.54166
H	-0.71538	-3.36690	-2.00592
C	-0.16306	-3.50680	0.71781
H	-1.24752	-3.42533	0.52055
H	0.03035	-3.01832	1.69124
C	2.57530	-4.32840	-0.01765
H	3.65817	-4.37152	0.20969
C	2.30975	-4.99756	-1.38669
H	2.64510	-6.05281	-1.36455
H	2.89125	-4.48609	-2.17721
C	0.79817	-4.92405	-1.70741
H	0.60266	-5.39651	-2.68945
C	-0.00195	-5.65939	-0.60624
H	0.28865	-6.72769	-0.57326
H	-1.08406	-5.62596	-0.83807
C	0.26886	-4.99260	0.76341
H	-0.30471	-5.51658	1.55210
C	1.77993	-5.06910	1.08373
H	1.97818	-4.61121	2.07157
H	2.10825	-6.12528	1.14214
C	-0.69887	1.27580	-0.30117
C	-2.00117	3.43803	-0.17169
C	-3.34628	3.43044	0.60432
H	-4.11604	2.90601	0.00533
H	-3.22007	2.85733	1.54026
C	-2.20940	4.21388	-1.49934
H	-2.99252	3.70821	-2.10389
H	-1.27409	4.21031	-2.08725
C	-0.92871	4.14656	0.68553
H	0.04941	4.12005	0.17824

H -0.80532 3.58468 1.62911
C -3.79051 4.88237 0.90962
H -4.74596 4.85342 1.46582
C -3.98174 5.65243 -0.41814
H -4.31163 6.68816 -0.21258
H -4.77643 5.17707 -1.02581
C -2.64744 5.66875 -1.19981
H -2.77893 6.20657 -2.15759
C -1.55664 6.36524 -0.35115
H -0.60198 6.39738 -0.90938
H -1.85015 7.41337 -0.15251
C -1.37131 5.60030 0.98075
H -0.58974 6.09486 1.58670
C -2.70705 5.58648 1.76053
H -2.57815 5.06027 2.72517
H -3.02458 6.61997 1.99628

TS(J'-K')

SCF (BP86) Energy = -2766.68943910
Enthalpy 0K = -2765.314913
Enthalpy 298K = -2765.239038
Free Energy 298K = -2765.424070
Lowest Frequency = -340.7802 cm^{-1}
Second Frequency = 11.8052 cm^{-1}
SCF (BP86-D3BJ) Energy = -2767.14863384
SCF (tol) Energy = -2766.69723897
SCF (BS2) Energy = -4150.66548696

K -2.20390 -1.18547 -1.90238
Si 3.29300 -3.10730 -0.97428
Si 4.33130 -0.36049 -1.81298
Al 1.65235 -0.53246 -0.02880
O   4.33905 -2.06338 -1.80752
N   1.71182 -2.28387 -0.77327
N   3.35615 0.20486 -0.42801
N   0.60555 0.21743 1.27182
C   0.56165 -3.04428 -1.16102
C   0.12816 -3.06827 -2.53402
C -1.02257 -3.80706 -2.89028
H -1.32982 -3.84265 -3.94302
C -1.75927 -4.52382 -1.93448
H -2.64322 -5.10007 -2.23074
C -1.30601 -4.54504 -0.60856
H -1.84530 -5.14522 0.13315
C -0.14768 -3.84918 -0.20597
C   0.88552 -2.32196 -3.63804
H   1.87615 -2.06621 -3.22801
C   1.09464 -3.17117 -4.91097
H   0.15001 -3.33612 -5.46056
H   1.51951 -4.16074 -4.67758
H   1.78460 -2.65503 -5.60035
C   0.19783 -0.98910 -4.01891
H   0.13621 -0.29457 -3.16046
H -0.81797 -1.16912 -4.42595
H   0.76063 -0.46775 -4.81215
C   0.38154 -4.07024 1.21238
H   1.21730 -3.36467 1.35370
C   0.93168 -5.51196 1.34787
H   1.69143 -5.74387 0.58269
H   0.11981 -6.25422 1.24257
H   1.39024 -5.65735 2.34186
C -0.65749 -3.79719 2.31751
H -1.50085 -4.51145 2.27009
H -1.05648 -2.77282 2.22291
H -0.19004 -3.91359 3.31130
C   3.15310 -4.67263 -2.03261
H   4.12059 -5.20256 -1.99255
H   2.94541 -4.43667 -3.08728
H   2.37246 -5.36084 -1.67025
C   4.08448 -3.59592 0.67839
H   3.96273 -2.82269 1.45122
H   5.16418 -3.77749 0.54006
H   3.63028 -4.52597 1.05924
C   3.65159 0.18267 -3.50391
H   2.55234 0.15369 -3.54154
H   4.04579 -0.47838 -4.29507
H   3.96632 1.21386 -3.73540
C   6.13618 0.20247 -1.72678
H   6.60482 0.03643 -2.71220
H   6.70133 -0.37806 -0.98233
H   6.22845 1.27208 -1.47799
C   3.89667 1.31574 0.32755
C   3.59138 2.66683 -0.03666
C   4.05428 3.72663 0.76888
H   3.80513 4.75624 0.48674
C   4.83079 3.49682 1.90876
H   5.17173 4.33246 2.52933
C   5.20240 2.18386 2.21655
H   5.85502 1.99903 3.07753
C   4.77863 1.08879 1.43708
C   2.85875 3.01988 -1.33263
H   2.51430 2.07322 -1.78250
C   3.83300 3.70146 -2.32461
H   4.73329 3.08928 -2.50283
H   4.17300 4.67801 -1.93589
H   3.34031 3.88162 -3.29710
C   1.62019 3.91210 -1.11485
H   1.87637 4.85298 -0.59606
H   0.85324 3.38760 -0.52293
H   1.17594 4.18727 -2.08848
C   5.35495 -0.29044 1.77563
H   5.07137 -0.96764 0.95305
C   6.90081 -0.25633 1.85486
H   7.30160 -1.28314 1.92413
H   7.24819 0.28976 2.74987
H   7.35047 0.23187 0.97545
C   4.79971 -0.88494 3.08911
H   3.71581 -1.06964 3.03485
H   4.97666 -0.20083 3.93844
H   5.29989 -1.84221 3.32110
C -1.09505 1.17824 -0.39099
C -2.59617 2.55537 -1.75086
C -4.05218 2.35581 -2.24450
H -4.11789 1.40815 -2.82015
H -4.71786 2.24417 -1.36791
C -2.53186 3.88031 -0.94401
H -1.49829 4.02132 -0.57825
H -3.18247 3.78768 -0.05415
C -1.67269 2.67785 -2.99055
H -1.71896 1.73688 -3.57815
H -0.62878 2.79368 -2.64867
C -4.49887 3.54244 -3.13055
H -5.53873 3.37518 -3.47044
C -4.42402 4.85095 -2.30710
H -4.75735 5.70670 -2.92511
H -5.10849 4.79333 -1.43911
C -2.97080 5.07359 -1.82508
H -2.91432 6.00617 -1.23205
C -2.03207 5.17938 -3.05074
H -2.32382 6.04092 -3.68168
H -0.99272 5.36023 -2.71746
C -2.10652 3.87154 -3.87613
H -1.43159 3.94401 -4.75052
C -3.56074 3.65134 -4.35680
H -3.87916 4.48961 -5.00567

```
H  -3.62480  2.72984 -4.96869
C  -0.39480  0.18832  0.26526
C   0.46174  1.01710  2.51578
C   0.49652  2.54056  2.22154
H   1.43412  2.77686  1.68829
H  -0.34374  2.80230  1.54960
C   1.64582  0.67617  3.44474
H   2.58914  0.89887  2.91628
H   1.62682 -0.41010  3.66034
C  -0.85503  0.70157  3.27793
H  -1.72102  0.94846  2.63843
H  -0.90537 -0.38284  3.48613
C   0.41014  3.36073  3.53239
H   0.43807  4.44098  3.29054
C  -0.90561  3.02571  4.27162
H  -1.77565  3.29642  3.64236
H  -0.98304  3.61830  5.20434
C  -0.93677  1.51371  4.59514
H  -1.88310  1.26439  5.11402
C   0.26184  1.15387  5.50286
H   0.23236  0.07756  5.76155
H   0.20476  1.71774  6.45454
C   1.57592  1.48770  4.76040
H   2.44277  1.22225  5.39582
C   1.61391  3.00004  4.43516
H   1.57985  3.59098  5.37166
H   2.56029  3.25234  3.92109
N  -2.24265  1.41191 -0.88782
N  -3.04049 -0.68931  0.67441
C  -1.86889 -0.92765  0.97554
C  -4.37244 -1.08799  1.12719
C  -4.31523 -2.16629  2.24097
H  -3.77778 -3.05427  1.86008
H  -3.72496 -1.77294  3.08842
C  -5.10505  0.16787  1.67052
H  -5.12042  0.94139  0.88115
H  -4.52469  0.58004  2.51618
C  -5.17816 -1.65125 -0.07461
H  -4.65870 -2.54736 -0.47231
H  -5.21427 -0.88537 -0.87516
C  -5.74791 -2.54003  2.69072
H  -5.68943 -3.30712  3.48582
C  -6.46509 -1.28019  3.23301
H  -7.48438 -1.54120  3.57654
H  -5.92083 -0.88161  4.11006
C  -6.53722 -0.20709  2.11993
H  -7.04277  0.69794  2.50583
C  -7.32615 -0.76851  0.91281
H  -8.35935 -1.02261  1.21685
H  -7.40287 -0.00231  0.11753
C  -6.61174 -2.02949  0.37057
H  -7.16721 -2.42969 -0.49884
C  -6.53666 -3.10220  1.48367
H  -7.55768 -3.39271  1.79628
H  -6.04494 -4.01672  1.09929
```

K'

SCF (BP86) Energy = -2766.76419918
Enthalpy 0K = -2765.386136
Enthalpy 298K = -2765.310971
Free Energy 298K = -2765.493301
Lowest Frequency = 10.9403 cm^{-1}
Second Frequency = 17.2415 cm^{-1}
SCF (BP86-D3BJ) Energy = -2767.23261919
SCF (tol) Energy = -2766.77622767
SCF (BS2) Energy = -4150.73972154

```
K  -3.03178  1.44388 -2.72098
Si  3.46884  2.21613 -0.48917
Si  2.86663  1.38970  2.45233
Al  1.22665  0.27350  0.17863
O   2.76795  2.38832  1.05788
N   2.71089  0.71039 -1.00084
N   1.76305  0.08092  2.02076
N  -3.50847 -0.79792 -1.36999
N   0.07468 -1.10364 -0.54518
N  -1.57944  2.02160 -0.49307
C   3.05992  0.01662 -2.20927
C   2.31713  0.21397 -3.42183
C   2.61416 -0.55000 -4.56761
H   2.03355 -0.38531 -5.48355
C   3.64301 -1.49575 -4.57484
H   3.85596 -2.08560 -5.47270
C   4.41378 -1.64868 -3.41915
H   5.24669 -2.36146 -3.42224
C   4.15914 -0.91026 -2.24615
C   1.25241  1.30220 -3.56074
H   1.10883  1.74968 -2.56234
C  -0.10783  0.74602 -4.03346
H  -0.79889  1.59190 -4.22971
H  -0.53560  0.05771 -3.28425
H  -0.01755  0.19398 -4.98517
C   1.72567  2.40502 -4.53752
H   2.71042  2.80409 -4.24868
H   1.01055  3.24829 -4.56252
H   1.81777  2.01062 -5.56534
C   5.12133 -1.11658 -1.07406
H   4.77559 -0.46586 -0.25311
C   5.13468 -2.56950 -0.55319
H   4.14274 -2.88626 -0.20186
H   5.84322 -2.66826  0.28841
H   5.45794 -3.27502 -1.33953
C   6.56439 -0.71408 -1.46535
H   6.97884 -1.41696 -2.21002
H   7.22942 -0.74005 -0.58337
H   6.61252  0.29529 -1.90197
C   2.98344  3.78042 -1.45431
H   1.90611  3.83460 -1.67072
H   3.52827  3.83528 -2.41199
H   3.26363  4.66981 -0.86282
C   5.36306  2.32782 -0.41524
H   5.62094  3.29895  0.04404
H   5.78646  2.32759 -1.43407
H   5.85257  1.53321  0.16364
C   2.34211  2.46123  3.92297
H   3.10365  3.24126  4.09669
H   2.26186  1.85972  4.84434
H   1.37440  2.95517  3.74627
C   4.70322  0.97605  2.73445
H   5.09906  0.24276  2.01401
H   4.87335  0.57927  3.74757
H   5.29067  1.90601  2.64096
C   1.18490 -0.84885  2.96200
C   1.88052 -2.04339  3.36177
C   1.24079 -2.98623  4.19130
H   1.78542 -3.89318  4.47690
C  -0.05544 -2.79046  4.67391
H  -0.53677 -3.54178  5.30930
C  -0.70770 -1.59838  4.35430
H  -1.70613 -1.40681  4.76459
C  -0.11592 -0.62095  3.52932
C   3.35149 -2.30355  3.03118
H   3.64641 -1.58776  2.24465
C   3.64078 -3.73343  2.52440
H   3.43013 -4.49019  3.30059
```

H	4.70619	-3.83579	2.25533
H	3.04083	-3.99111	1.63772
C	4.21387	-2.04905	4.29417
H	3.99553	-1.07127	4.75270
H	5.29229	-2.09021	4.05778
H	4.00553	-2.81863	5.05908
C	-0.89295	0.68352	3.34894
H	-0.32675	1.30422	2.63380
C	-2.30882	0.45978	2.77707
H	-2.27868	-0.06836	1.81120
H	-2.81962	1.42756	2.62298
H	-2.93121	-0.12914	3.47535
C	-1.00413	1.44855	4.69055
H	-1.62175	0.88523	5.41296
H	-1.48572	2.43225	4.53968
H	-0.02064	1.61621	5.15577
C	-2.39700	-0.72991	-0.75516
C	-4.78112	-1.16324	-0.66648
C	-4.58146	-2.10407	0.54365
H	-3.90275	-1.62729	1.27344
H	-4.08711	-3.03223	0.20032
C	-5.70057	-1.86113	-1.69825
H	-5.20532	-2.78034	-2.06406
H	-5.83385	-1.19757	-2.57650
C	-5.47323	0.13887	-0.18300
H	-5.62481	0.81940	-1.04886
H	-4.80828	0.65753	0.53232
C	-5.94690	-2.43541	1.19315
H	-5.78486	-3.10720	2.05699
C	-6.85530	-3.13398	0.15309
H	-7.83001	-3.39263	0.60944
H	-6.39262	-4.08287	-0.17915
C	-7.06970	-2.19455	-1.05838
H	-7.71241	-2.69301	-1.80851
C	-7.74584	-0.88711	-0.57970
H	-7.92710	-0.21382	-1.44066
H	-8.73399	-1.11095	-0.13459
C	-6.84129	-0.18716	0.46296
H	-7.31793	0.75252	0.80171
C	-6.62106	-1.12762	1.67213
H	-7.59051	-1.35319	2.15610
H	-5.98835	-0.62918	2.43063
C	-1.09859	-0.34986	-0.62538
C	0.07356	-2.54470	-0.82642
C	1.52305	-3.06078	-0.70786
H	1.89080	-2.82787	0.30973
H	2.16004	-2.51448	-1.42700
C	-0.42633	-2.85437	-2.26927
H	0.21204	-2.30527	-2.98718
H	-1.46301	-2.48964	-2.39364
C	-0.79974	-3.32774	0.19654
H	-1.84304	-2.96627	0.15474
H	-0.42770	-3.11747	1.21616
C	1.59538	-4.58278	-0.98001
H	2.64562	-4.91856	-0.88194
C	1.09295	-4.86551	-2.41592
H	1.15380	-5.94949	-2.63451
H	1.73647	-4.34626	-3.15123
C	-0.36806	-4.37530	-2.55081
H	-0.73211	-4.57011	-3.57831
C	-1.26220	-5.12113	-1.53184
H	-1.24904	-6.20908	-1.73858
H	-2.31311	-4.78619	-1.63347
C	-0.75232	-4.84684	-0.09695
H	-1.39200	-5.37924	0.63319
C	0.70671	-5.34184	0.03338
H	1.06861	-5.17211	1.06497
H	0.76124	-6.43196	-0.15529
C	-0.70616	1.08587	-0.25860
C	-1.39316	3.44753	-0.15220
C	-2.73552	3.92448	0.47816
H	-3.56693	3.68609	-0.21668
H	-2.92090	3.34660	1.40223
C	-1.17747	4.26197	-1.46089
H	-2.01091	4.05558	-2.16920
H	-0.24419	3.92946	-1.95269
C	-0.24974	3.78023	0.83253
H	0.72113	3.44221	0.43758
H	-0.41376	3.21539	1.76883
C	-2.70556	5.44049	0.77931
H	-3.67097	5.74239	1.22845
C	-2.47884	6.21907	-0.53836
H	-2.46748	7.30816	-0.34248
H	-3.31360	6.02880	-1.24250
C	-1.13574	5.78190	-1.16879
H	-0.97103	6.32935	-2.11679
C	0.01625	6.08470	-0.18178
H	0.98650	5.80568	-0.63109
H	0.05558	7.17178	0.02401
C	-0.21134	5.29994	1.13172
H	0.61879	5.50592	1.83279
C	-1.55118	5.74261	1.76411
H	-1.71770	5.20691	2.71811
H	-1.52617	6.82387	2.00079

5·toluene

SCF (BP86) Energy = -3038.33476310
Enthalpy 0K = -3036.831504
Enthalpy 298K = -3036.747657
Free Energy 298K = -3036.954491
Lowest Frequency = 7.1556 cm^{-1}
Second Frequency = 9.1801 cm^{-1}
SCF (BP86-D3BJ) Energy = -3038.83828388
SCF (tol) Energy = -3038.34368070
SCF (BS2) Energy = -4422.37300350

K	-3.49225	-0.73813	1.25668
Si	2.95740	-3.16464	0.19225
Si	3.51019	-1.47650	-2.36185
Al	1.64328	-0.44460	-0.21708
O	2.73731	-2.69724	-1.43428
N	2.54032	-1.66081	1.00894
N	2.73425	-0.01279	-1.74918
N	-2.84579	1.80626	0.65379
N	0.76499	1.02351	0.68209
N	-1.60593	-1.20345	-0.71584
C	2.69984	-1.45887	2.42219
C	1.61017	-1.67107	3.33224
C	1.76134	-1.37151	4.70056
H	0.91550	-1.53805	5.37882
C	2.96543	-0.88815	5.21979
H	3.06281	-0.65420	6.28531
C	4.05140	-0.73944	4.35273
H	5.01208	-0.39416	4.75252
C	3.95423	-1.02445	2.97605
C	0.28935	-2.30457	2.89478
H	0.32541	-2.41695	1.79773
C	-0.93484	-1.43194	3.24410
H	-1.86353	-1.99715	3.02779
H	-0.92077	-0.48354	2.68018
H	-0.96592	-1.18327	4.31950
C	0.12342	-3.70567	3.53098
H	0.99706	-4.34607	3.33238
H	-0.77188	-4.21868	3.13279
H	0.01078	-3.63220	4.62768

C	5.23385	-0.88957	2.14769
H	4.97522	-1.15468	1.10866
C	5.79935	0.54654	2.14320
H	5.08082	1.26610	1.72656
H	6.72159	0.59369	1.53705
H	6.05931	0.87853	3.16439
C	6.33052	-1.85575	2.65879
H	6.68943	-1.54848	3.65738
H	7.20263	-1.84963	1.98033
H	5.96828	-2.89187	2.74275
C	1.78198	-4.63374	0.47053
H	0.72055	-4.34542	0.44195
H	1.97985	-5.11515	1.44325
H	1.95756	-5.38970	-0.31505
C	4.67274	-3.92966	0.47457
H	4.76846	-4.79293	-0.20819
H	4.75332	-4.31880	1.50388
H	5.51843	-3.25271	0.29311
C	3.16908	-1.88065	-4.18025
H	3.71111	-2.80055	-4.46040
H	3.52011	-1.06929	-4.84028
H	2.09615	-2.04019	-4.36784
C	5.38683	-1.68674	-2.11937
H	5.73639	-1.35268	-1.12954
H	5.95688	-1.13521	-2.88312
H	5.63407	-2.75691	-2.23083
C	2.74716	1.25838	-2.43445
C	3.84051	2.18233	-2.28498
C	3.77383	3.45622	-2.88427
H	4.61496	4.14599	-2.75171
C	2.67982	3.85763	-3.65417
H	2.64551	4.85634	-4.10286
C	1.64715	2.94208	-3.86319
H	0.80234	3.22383	-4.50262
C	1.66158	1.65489	-3.28957
C	5.15556	1.81926	-1.59243
H	4.99117	0.87774	-1.04076
C	5.65618	2.88879	-0.59682
H	5.90455	3.83522	-1.10880
H	6.57561	2.54338	-0.09348
H	4.90960	3.11741	0.17962
C	6.25613	1.59344	-2.66064
H	5.92818	0.89906	-3.45071
H	7.17959	1.19259	-2.20553
H	6.50986	2.54859	-3.15472
C	0.51881	0.71963	-3.68578
H	0.64576	-0.21330	-3.11111
C	-0.86927	1.30463	-3.35032
H	-0.96701	1.52813	-2.27640
H	-1.66549	0.58905	-3.62450
H	-1.05700	2.23438	-3.91783
C	0.57665	0.38407	-5.19614
H	0.38538	1.28516	-5.80621
H	-0.19473	-0.36315	-5.45904
H	1.55746	-0.01638	-5.49530
C	-1.66919	1.49326	0.28966
C	-3.69586	2.74486	-0.15066
C	-2.89593	3.84536	-0.88538
H	-2.15839	3.37589	-1.56073
H	-2.32662	4.43447	-0.14191
C	-4.69683	3.41716	0.81995
H	-4.13607	3.99161	1.58136
H	-5.26119	2.63187	1.36181
C	-4.49310	1.92004	-1.19538
H	-5.07431	1.12969	-0.67405
H	-3.78189	1.41302	-1.87325
C	-3.85755	4.76791	-1.67393
H	-3.26884	5.54695	-2.19389
C	-4.85100	5.43452	-0.69245
H	-5.53399	6.11144	-1.24082
H	-4.30173	6.05515	0.04104
C	-5.66431	4.34044	0.04087
H	-6.37110	4.81262	0.74947
C	-6.44935	3.50403	-0.99830
H	-7.05540	2.73084	-0.48570
H	-7.15677	4.15110	-1.55122
C	-5.45875	2.83755	-1.98309
H	-6.01662	2.23190	-2.72291
C	-4.64088	3.93016	-2.71247
H	-5.31805	4.58268	-3.29629
H	-3.94077	3.46376	-3.43100
C	-0.54555	0.73234	0.28846
C	1.08116	2.24989	1.42350
C	2.58893	2.23952	1.75225
H	3.14961	2.16447	0.80095
H	2.82056	1.33902	2.34950
C	0.30061	2.33136	2.76934
H	0.53285	1.42808	3.36445
H	-0.78777	2.33300	2.57246
C	0.78333	3.52499	0.58227
H	-0.28703	3.55383	0.30991
H	1.35408	3.46824	-0.36297
C	2.99879	3.51418	2.52860
H	4.08424	3.47456	2.74236
C	2.21044	3.57342	3.85848
H	2.50382	4.47192	4.43585
H	2.45117	2.69073	4.48088
C	0.69467	3.60791	3.55122
H	0.12407	3.64545	4.49963
C	0.36605	4.85551	2.69713
H	0.61992	5.77750	3.25589
H	-0.72066	4.89465	2.48639
C	1.16362	4.80098	1.37214
H	0.92860	5.69289	0.75963
C	2.67758	4.77030	1.68496
H	3.25500	4.75709	0.74125
H	2.97517	5.68430	2.23549
C	-0.47985	-0.60811	-0.45021
C	-1.72149	-2.46036	-1.48455
C	-2.86297	-2.23346	-2.52063
H	-3.77310	-1.89215	-1.98676
H	-2.56868	-1.41313	-3.20076
C	-2.17765	-3.60293	-0.53141
H	-3.10073	-3.29344	0.00531
H	-1.39895	-3.77570	0.23522
C	-0.46185	-2.92551	-2.24834
H	0.38217	-3.07974	-1.55871
H	-0.14803	-2.12189	-2.93959
C	-3.15854	-3.52305	-3.32049
H	-3.96929	-3.32418	-4.04744
C	-3.59739	-4.64013	-2.34496
H	-3.82715	-5.56833	-2.90233
H	-4.52801	-4.34256	-1.82017
C	-2.46708	-4.90280	-1.32177
H	-2.77854	-5.69651	-0.61518
C	-1.18959	-5.34437	-2.07397
H	-0.38093	-5.56464	-1.35361
H	-1.38841	-6.27877	-2.63369
C	-0.75085	-4.22269	-3.04436
H	0.17045	-4.53159	-3.57244
C	-1.87930	-3.96553	-4.06984
H	-1.57091	-3.18099	-4.78691
H	-2.08045	-4.88111	-4.65919
C	-6.59867	-2.27692	1.59821
C	-6.79040	-0.99238	2.15397
H	-7.46776	-0.28879	1.65639

```
C  -6.14447 -0.61104  3.34449
H  -6.31982  0.38583  3.76243
C  -5.28588 -1.51032  4.00197
H  -4.79145 -1.22052  4.93459
C  -5.07763 -2.79078  3.45603
H  -4.41814 -3.50265  3.96345
C  -5.72448 -3.16577  2.26509
H  -5.56462 -4.16964  1.85497
C  -7.34006 -2.70737  0.35011
H  -7.66750 -1.84208 -0.24854
H  -8.24465 -3.28470  0.61455
H  -6.71937 -3.35545 -0.29083
```

L’

SCF (BP86) Energy = -2766.73339515
Enthalpy 0K = -2765.357885
Enthalpy 298K = -2765.280697
Free Energy 298K = -2765.474080
Lowest Frequency = 2.3889 cm^{-1}
Second Frequency = 5.8066 cm^{-1}
SCF (BP86-D3BJ) Energy = -2767.17816448
SCF (tol) Energy = -2766.74351081
SCF (BS2) Energy = -4150.70853235

```
K  -2.94268  0.44665 -0.85022
Si  4.23579  1.94178 -1.03775
Si  3.89783  1.44363  2.08205
Al  2.08153  0.31665  0.09068
O   3.61816  2.18674  0.55130
N   3.34928  0.46803 -1.37832
N   2.80396  0.08417  1.87714
N  -7.04189 -0.20077 -0.54860
N   0.74026 -1.11392 -0.31600
N  -0.75915  1.99221 -0.28659
C   3.37511 -0.30618 -2.58346
C   2.39601 -0.10941 -3.61644
C   2.38430 -0.95817 -4.74134
H   1.62754 -0.79585 -5.51820
C   3.31778 -1.98695 -4.90047
H   3.28720 -2.63920 -5.78003
C   4.30863 -2.14707 -3.92705
H   5.06925 -2.92577 -4.05894
C   4.36637 -1.32804 -2.78203
C   1.40066  1.05247 -3.59840
H   1.48359  1.54138 -2.61231
C  -0.06678  0.60670 -3.77806
H  -0.73479  1.48628 -3.72132
H  -0.36455 -0.10581 -2.99051
H  -0.23038  0.12851 -4.76094
C   1.76454  2.08800 -4.68976
H   2.80877  2.42750 -4.59795
H   1.10815  2.97502 -4.62649
H   1.64536  1.65377 -5.69886
C   5.54866 -1.52753 -1.83093
H   5.38076 -0.86437 -0.96513
C   5.67952 -2.97286 -1.30450
H   4.77842 -3.29309 -0.76063
H   6.53903 -3.05342 -0.61545
H   5.85154 -3.69044 -2.12660
C   6.87141 -1.12200 -2.52609
H   7.10417 -1.81627 -3.35348
H   7.71772 -1.15385 -1.81615
H   6.81888 -0.10815 -2.95328
C   3.75755  3.49470 -2.01844
H   2.66653  3.62810 -2.07850
H   4.15420  3.45124 -3.04704
H   4.19189  4.38565 -1.53148
C   6.13522  1.93764 -1.05632
H   6.49113  2.86284 -0.56908
H   6.50041  1.95807 -2.09728
H   6.59619  1.07901 -0.54838
C   3.42903  2.69421  3.42579
H   4.14385  3.53540  3.42707
H   3.46601  2.22212  4.42294
H   2.41640  3.09743  3.27272
C   5.76573  1.14562  2.27545
H   6.14952  0.34514  1.62457
H   6.01935  0.88899  3.31650
H   6.29913  2.07969  2.02668
C   2.35229 -0.82208  2.89331
C   3.15046 -1.94677  3.30260
C   2.64465 -2.86455  4.24444
H   3.26965 -3.71498  4.54068
C   1.37909 -2.71496  4.81800
H   1.00299 -3.44379  5.54416
C   0.61535 -1.60088  4.46058
H  -0.36473 -1.45081  4.92884
C   1.07167 -0.65072  3.52507
C   4.58717 -2.16032  2.82350
H   4.76203 -1.45344  1.99485
C   4.86303 -3.58848  2.30385
H   4.76194 -4.34020  3.10685
H   5.89449 -3.66287  1.91713
H   4.17567 -3.87262  1.49196
C   5.57884 -1.84748  3.97174
H   5.38722 -0.86128  4.42460
H   6.62392 -1.86784  3.61397
H   5.48362 -2.59841  4.77672
C   0.18225  0.57132  3.28874
H   0.63283  1.15609  2.46844
C  -1.25495  0.19549  2.86539
H  -1.25121 -0.43108  1.95775
H  -1.83735  1.11066  2.65115
H  -1.78251 -0.35510  3.66541
C   0.13249  1.46547  4.55088
H  -0.35156  0.93590  5.39138
H  -0.44858  2.38578  4.35679
H   1.14063  1.76101  4.88201
C  -5.89783  0.03248 -0.74418
C  -8.42896 -0.49198 -0.29175
C  -8.52708 -1.75887  0.60332
H  -7.97983 -1.58168  1.54702
H  -8.03844 -2.60676  0.08977
C  -9.16197 -0.74529 -1.63828
H  -8.67682 -1.58715 -2.16495
H  -9.06503  0.14902 -2.28046
C  -9.07986  0.71560  0.43868
H  -8.98226  1.61852 -0.19120
H  -8.53624  0.90732  1.38165
C  -10.01816 -2.06733  0.88016
H  -10.07938 -2.96888  1.51677
C  -10.74977 -2.32050 -0.46003
H  -11.81132 -2.56532 -0.27010
H  -10.30640 -3.19136 -0.97901
C  -10.65038 -1.05899 -1.35123
H  -11.16482 -1.23812 -2.31311
C  -11.30024  0.14298 -0.62432
H  -11.25336  1.04640 -1.26157
H  -12.37039 -0.06333 -0.43690
C  -10.56851  0.39523  0.71596
H  -11.02414  1.25829  1.23506
C  -10.66744 -0.86567  1.60792
H  -11.72751 -1.08768  1.83132
H  -10.16455 -0.68858  2.57748
C  -0.33411 -0.34788 -0.46072
```

C   0.62839 -2.58966 -0.46136
C   2.03228 -3.21161 -0.33208
H   2.46003 -2.91916 0.64505
H   2.68039 -2.79258 -1.12366
C   0.03994 -2.97369 -1.84440
H   0.67858 -2.54969 -2.64174
H -0.96011 -2.51127 -1.94155
C -0.28452 -3.16910 0.65220
H -1.28525 -2.70603 0.55746
H   0.11769 -2.88352 1.64204
C   1.95505 -4.75166 -0.45679
H   2.97175 -5.17909 -0.35862
C   1.36997 -5.12165 -1.84071
H   1.32460 -6.22250 -1.95434
H   2.02542 -4.73567 -2.64410
C -0.04680 -4.51289 -1.97682
H -0.46762 -4.76957 -2.96844
C -0.95855 -5.07801 -0.86147
H -1.04245 -6.17814 -0.95840
H -1.98227 -4.66593 -0.96260
C -0.37245 -4.70780 0.52199
H -1.02628 -5.10421 1.32293
C   1.04474 -5.31463 0.66072
H   1.46463 -5.06643 1.65378
H   0.99541 -6.41901 0.59218
C   0.16754 1.07799 -0.19996
C -0.54319 3.43178 -0.06005
C -1.78832 3.95794 0.71271
H -2.70383 3.67321 0.15453
H -1.83897 3.45589 1.69840
C -0.51635 4.13549 -1.44777
H -1.42227 3.84689 -2.01733
H   0.35393 3.76488 -2.02105
C   0.72212 3.83084 0.73129
H   1.62826 3.45441 0.23055
H   0.69078 3.34014 1.72288
C -1.72726 5.49279 0.88944
H -2.62256 5.83410 1.44433
C -1.69085 6.16448 -0.50395
H -1.65970 7.26576 -0.39501
H -2.61309 5.92220 -1.06794
C -0.44631 5.67195 -1.28005
H -0.41941 6.14607 -2.28006
C   0.83089 6.04458 -0.48996
H   1.72993 5.71940 -1.04604
H   0.89951 7.14427 -0.37853
C   0.79316 5.36892 0.90169
H   1.71045 5.62882 1.46324
C -0.44913 5.86644 1.67709
H -0.47716 5.40874 2.68477
H -0.39885 6.96322 1.82171

TS(L′-K′)
SCF (BP86) Energy = -2766.69426403
Enthalpy 0K = -2765.318908
Enthalpy 298K = -2765.243372
Free Energy 298K = -2765.427883
Lowest Frequency = -73.0719 cm^{-1}
Second Frequency = 9.6645 cm^{-1}
SCF (BP86-D3BJ) Energy = -2767.15206688
SCF (tol) Energy = -2766.70527059
SCF (BS2) Energy = -4150.67094316

K -2.87460 1.30140 -2.15297
Si 3.87717 1.62974 -0.90910
Si 3.43873 1.12431 2.16938
Al 1.48663 0.22250 0.14703
O   3.30092 1.95914 0.67036
N   2.84223 0.25186 -1.26506
N   2.14064 -0.04434 1.94285
N -4.17150 -0.37190 -0.45486
N -0.03077 -1.01742 -0.37327
N -1.02981 2.29483 -0.48397
C   2.92971 -0.52225 -2.47114
C   2.11122 -0.22250 -3.61328
C   2.15760 -1.04893 -4.75363
H   1.52356 -0.80335 -5.61424
C   3.00433 -2.15789 -4.82639
H   3.02283 -2.79172 -5.71942
C   3.84758 -2.42259 -3.74390
H   4.54113 -3.26968 -3.80113
C   3.84086 -1.63030 -2.57886
C   1.22778 1.02330 -3.69043
H   1.24822 1.50634 -2.69851
C -0.24201 0.68528 -4.02126
H -0.83195 1.62222 -4.08345
H -0.66620 0.01628 -3.25112
H -0.33699 0.18264 -5.00009
C   1.77358 2.01990 -4.74042
H   2.83050 2.26570 -4.55364
H   1.19658 2.96281 -4.73011
H   1.70822 1.59566 -5.75842
C   4.86228 -1.98287 -1.49548
H   4.69492 -1.28965 -0.65315
C   4.70511 -3.42483 -0.96717
H   3.70596 -3.59911 -0.54325
H   5.45139 -3.62830 -0.17898
H   4.86612 -4.16634 -1.77008
C   6.30739 -1.80243 -2.02196
H   6.53517 -2.55274 -2.80016
H   7.04115 -1.93941 -1.20720
H   6.46859 -0.80831 -2.46607
C   3.58120 3.22783 -1.89250
H   2.51262 3.46391 -2.00801
H   4.03215 3.16880 -2.89733
H   4.06489 4.06678 -1.36153
C   5.76387 1.42419 -0.94554
H   6.21198 2.33542 -0.51050
H   6.11723 1.35553 -1.98854
H   6.14479 0.55448 -0.39342
C   3.19864 2.40678 3.54079
H   4.05746 3.10042 3.54903
H   3.15022 1.92234 4.53086
H   2.27877 2.99364 3.39693
C   5.22317 0.49289 2.34952
H   5.45930 -0.34121 1.67059
H   5.41970 0.15621 3.38003
H   5.91857 1.32321 2.13572
C   1.54972 -0.83216 2.99519
C   2.13287 -2.07860 3.41016
C   1.48776 -2.86674 4.38419
H   1.94659 -3.81580 4.68468
C   0.29168 -2.46674 4.98520
H -0.19807 -3.09926 5.73357
C -0.25026 -1.22999 4.62881
H -1.16886 -0.88539 5.11770
C   0.35417 -0.39951 3.66438
C   3.49309 -2.56890 2.91169
H   3.79354 -1.91273 2.07710
C   3.47136 -4.02447 2.39872
H   3.20188 -4.73392 3.20109
H   4.46754 -4.31836 2.02510
H   2.74858 -4.15916 1.57880
C   4.54360 -2.45480 4.04464
H   4.55057 -1.45127 4.49985

H	5.55975	-2.67376	3.67041
H	4.31950	-3.17518	4.85179
C	-0.28479	0.97058	3.43577
H	0.26547	1.45420	2.61049
C	-1.77024	0.87687	3.02990
H	-1.92744	0.25022	2.13829
H	-2.17078	1.88442	2.81445
H	-2.38030	0.45478	3.84975
C	-0.15596	1.85490	4.70013
H	-0.74913	1.43569	5.53275
H	-0.53678	2.87401	4.50323
H	0.88606	1.93907	5.04574
C	-3.17856	-0.61151	0.23739
C	-5.61597	-0.58005	-0.21901
C	-5.88523	-1.30467	1.12590
H	-5.45421	-0.70818	1.95052
H	-5.35800	-2.27618	1.12261
C	-6.19324	-1.43016	-1.38012
H	-5.67147	-2.40430	-1.41046
H	-5.99109	-0.92217	-2.34406
C	-6.32747	0.79811	-0.20236
H	-6.13524	1.32496	-1.15969
H	-5.90172	1.41847	0.60796
C	-7.40675	-1.50238	1.32267
H	-7.58291	-2.02069	2.28375
C	-7.97102	-2.35356	0.15997
H	-9.05618	-2.51887	0.29993
H	-7.49135	-3.35066	0.15059
C	-7.71600	-1.62792	-1.18323
H	-8.11123	-2.23636	-2.01812
C	-8.41553	-0.24756	-1.16560
H	-8.25978	0.27219	-2.13117
H	-9.50749	-0.37708	-1.04392
C	-7.85086	0.60390	-0.00332
H	-8.34161	1.59517	0.00793
C	-8.10527	-0.12170	1.34014
H	-9.19304	-0.24585	1.50050
H	-7.72265	0.48798	2.18060
C	-0.93792	-0.08840	-0.64834
C	-0.30303	-2.46620	-0.54940
C	1.03063	-3.20539	-0.78980
H	1.70558	-2.99300	0.06129
H	1.51892	-2.81244	-1.69988
C	-1.24528	-2.74093	-1.75074
H	-0.77785	-2.33803	-2.67053
H	-2.19848	-2.20738	-1.59583
C	-0.94749	-3.02718	0.75209
H	-1.88466	-2.47971	0.95039
H	-0.26799	-2.83944	1.60362
C	0.78938	-4.72903	-0.92791
H	1.75937	-5.23447	-1.09644
C	-0.14629	-4.98595	-2.13301
H	-0.31479	-6.07310	-2.25955
H	0.32473	-4.61821	-3.06456
C	-1.49272	-4.26135	-1.89662
H	-2.16554	-4.43765	-2.75865
C	-2.15330	-4.79542	-0.60315
H	-2.36490	-5.87795	-0.70279
H	-3.12382	-4.28853	-0.43729
C	-1.21131	-4.54473	0.59812
H	-1.68218	-4.92182	1.52598
C	0.12712	-5.27976	0.35743
H	0.79433	-5.13714	1.22750
H	-0.04457	-6.36940	0.25660
C	-0.28033	1.24638	-0.28466
C	-0.65002	3.68187	-0.16758
C	-1.87356	4.32184	0.55414
H	-2.77728	4.17522	-0.07163
H	-2.05088	3.78398	1.50401
C	-0.44938	4.44144	-1.51171
H	-1.35129	4.30494	-2.14506
H	0.40029	3.99329	-2.06013
C	0.59980	3.88533	0.71651
H	1.48647	3.42576	0.25174
H	0.44733	3.35777	1.67659
C	-1.63830	5.82753	0.81491
H	-2.52107	6.25015	1.33195
C	-1.42827	6.55436	-0.53461
H	-1.27082	7.63676	-0.36507
H	-2.33376	6.45629	-1.16574
C	-0.20357	5.94815	-1.25980
H	-0.05350	6.45866	-2.23056
C	1.05465	6.12208	-0.37611
H	1.94238	5.71360	-0.89395
H	1.24714	7.19863	-0.20331
C	0.84246	5.39388	0.97244
H	1.74508	5.51191	1.60105
C	-0.37977	6.00415	1.69719
H	-0.52928	5.50723	2.67484
H	-0.20784	7.07833	1.90363
